# Interactive effects of cold spell and air pollution on outpatient visits for anxiety in three subtropical Chinese cities

**DOI:** 10.1016/j.scitotenv.2021.152789

**Published:** 2022-04-15

**Authors:** Huan Li, Min Li, Shiyu Zhang, Zhengmin (Min) Qian, Zilong Zhang, Kai Zhang, Chongjian Wang, Lauren D. Arnold, Stephen Edward McMillin, Shaowei Wu, Fei Tian, Hualiang Lin

**Affiliations:** a Department of Epidemiology, School of Public Health, Sun Yat-sen University, Guangzhou 510080, China; b Department of Preventive Medicine, The Third Affiliated Hospital of Guangzhou University of Chinese Medicine, The Third Clinical Medical Institute Affiliated to Guangzhou University of Chinese Medicine, Guangzhou, China; c Department of Epidemiology and Biostatistics, College for Public Health & Social Justice, Saint Louis University, Saint Louis, USA; d Department of Environmental Health Sciences, School of Public Health, University at Albany, State University of New York, USA; e Department of Epidemiology and Biostatistics, College of Public Health, Zhengzhou University, Zhengzhou, Henan 450001, China; f School of Social Work, College for Public Health & Social Justice, Saint Louis University, Saint Louis, USA; g Department of Occupational and Environmental Health, School of Public Health, Xi'an Jiaotong University Health Science Center, Xi'an, Shaanxi 710000, China

**Keywords:** Cold spell, Air pollution, Anxiety

## Abstract

**Background:**

Although low temperature and air pollution exposures have been associated with the risk of anxiety, their combined effects remain unclear.

**Objective:**

To investigate the independent and interactive effects of low temperature and air pollution exposures on anxiety.

**Method:**

Using a case-crossover study design, the authors collected data from 101,636 outpatient visits due to anxiety in three subtropical Chinese cities during the cold season (November to April in 2013 through 2018), and then built conditional logistic regression models based on individual exposure assessments [temperature, relative humidity, particulate matter (PM_2.5_, PM_10_), sulfur dioxide (SO_2_), and nitrogen dioxide (NO_2_)] and twelve cold spell definitions. Additive-scale interactions were assessed using the relative excess risk due to interaction (RERI).

**Results:**

Both cold spell and air pollution were significantly associated with outpatients for anxiety. The effects of cold spell increased with its intensity, ranging from 8.98% (95% CI: 2.02%, 16.41%) to 15.24% (95% CI: 6.75%, 24.39%) in Huizhou. Additionally, each 10 μg/m^3^ increase of PM_2.5_, PM_10_, NO_2_ and SO_2_ was associated with a 1.51% (95% CI: 0.61%, 2.43%), 1.58% (95% CI: 0.89%, 2.28%), 13.95% (9.98%, 18.05%) and 11.84% (95% CI: 8.25%, 15.55%) increase in outpatient visits for anxiety. Synergistic interactions (RERI >0) of cold spell with all four air pollutants on anxiety were observed, especially for more intense cold spells. For particulate matters, these interactions were found even under mild cold spell definitions [RERI: 0.11 (95% CI: 0.02, 0.21) for PM_2.5_, and 0.24 (95% CI: 0.14, 0.33) for PM_10_]. Stratified analyses yielded a pronounced results in people aged 18–65 years.

**Conclusions:**

These findings indicate that both cold spell and air pollution are important drivers of the occurrence of anxiety, and simultaneous exposure to these two factors might have synergistic effects on anxiety. These findings highlight the importance of controlling air pollution and improving cold-warning systems.

## Highlights

• Both cold spell and air pollution were significantly associated with anxiety.
• Simultaneous exposure to these two factors might have synergistic effect on anxiety.
• Interaction between particulates and cold spell was found even on mildly cold days.
• People aged 18–65 years were more vulnerable to cold spell and air pollution.

## Graphical abstract

**Figure:**
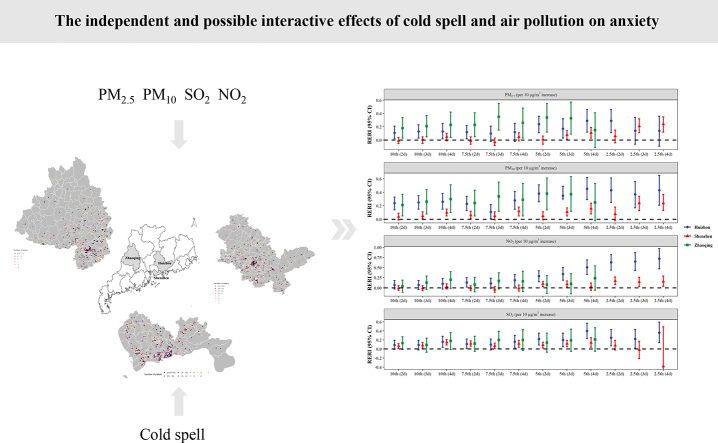

## 1 Introduction

Anxiety disorders are among the most common mental health disorders worldwide and include a range of conditions characterized by fear and worry (Schiele et al., 2020). Globally, an estimated 301 million individuals had an anxiety disorder in 2019 (GBD 2019 Diseases and Injuries Collaborators, 2020). In China, the lifetime prevalence of anxiety was estimated to be 4.1% (Guo et al., 2016). Anxiety substantially impacts both individuals and society, resulting in 28.7 million disability-adjusted life-years (DALYs), as well as decreased productivity, increased psychiatric and non-psychiatric healthcare cost, absenteeism, and risk of suicide (Lépine, 2002).

### 1.1 Related works

Previous studies suggested that air pollution might be one preventable risk factor associated with the onset of anxiety (Lu et al., 2020; Yue et al., 2020), with a more pronounced effect during the cold season (Song et al., 2018; Tong et al., 2016). The adverse impact of exposure to extreme temperatures, especially cold temperatures, on anxiety and other mental disorders has been reported as well (Muller et al., 2012; Zhang et al., 2020). While some studies have controlled for temperature as a confounder (Lu et al., 2020; Zhang et al., 2020), climate change concerns have paid recent attention to estimating the joint effects of air pollution and weather extremes (i.e., heatwaves, cold spells) (Chen et al., 2018; Liang et al., 2018). Most of these studies focused on the effects of heatwave and air pollution on anxiety (Yang et al., 2019), with less data on the interactive impact of cold spells and air pollution (Chen et al., 2019). Additionally, single definition of weather extremes was used in these studies (Diaz et al., 2020), which may raise concerns about biased associations. While the synergistic effects of air pollution and extreme temperature on all-cause mortality, cardiovascular mortality, and preterm birth (Chen et al., 2018) have been reported, it remains unclear whether air pollution and cold spell could work synergistically on anxiety. Furthermore, the ecological nature of many of these studies meant that exposure assessments were largely based on air pollution data from fixed monitoring stations and city-level meteorological data in previous studies, resulting in the lack of individual-level exposure information and the potential for ecological fallacy (Gao et al., 2017; Gu et al., 2020).

To address these research gaps, we collected data about outpatient visits due to anxiety in three subtropical Chinese cities, assessed air pollution and cold spell exposures for each participant, and then conducted a case-crossover study to explore the independent and possible interactive effects of cold spell and air pollution on anxiety.

## 2 Methods

### 2.1 Study population and outcome definition

Daily outpatient visit data were obtained from hospital information systems for three psychiatric specialty hospitals in the three subtropical Chinese cities of Huizhou, Shenzhen and Zhaoqing (Fig. S1). A total of 101,636 individuals with anxiety disorders (International Classification of Disease-10 codes: F40–F41) were identified in the cold season (November to April) during 2013–2018 for Huizhou; 2016–2018 for Shenzhen; and 2016–2018 for Zhaoqing. Variables of interest included age, sex, date of hospital visit, clinical diagnoses, and home address of each case. Patients who resided outside their city for more than six months prior to their outpatient visit were excluded. Detailed information about the study area and corresponding hospitals has been described elsewhere (Li et al., 2020; Zhang et al., 2020). The study protocol was approved by the Ethics Committee at the School of Public Health, Sun Yat-Sen University ([2019] No.149). Since the collected data is part of the outpatient registration information and is anonymous, informed consent was waived.

### 2.2 Study design

A time stratified case-crossover study was used to evaluate the independent and combined effects of air pollution (PM_10_, PM_2.5_, SO_2_, and NO_2_) and cold spell on outpatient visits for anxiety (Liu et al., 2021). Each case served as their own control: the day of the outpatient visit was defined as the case day, while days sharing the same year, month, and day of week with the case day were defined as the control days. For example, if a patient went to the hospital for anxiety on Wednesday, April 13, 2016, that date was assigned as the case day, with all other Wednesdays in April 2016 (i.e., April 6, 20, and 27) were assigned as control days. This design thus has the advantage of controlling for some individual factors such as age, sex, and genetics, as well as minimizing time-trend bias (Lu et al., 2020).

### 2.3 Exposure assessment

Exposure to PM_10_, PM_2.5_, SO_2_, and NO_2_ was assessed based on a six-year-long (2013–2018) Chinese air quality reanalysis dataset (CAQRA). The CAQRA was developed by assimilating over 1000 surface air quality monitoring sites operated by China National Environmental Monitoring Center (CNEMC). Using a post-processing mode, the CNEMC uses its own chemical data assimilation system (ChemDAS) to develop the CAQRA. This dataset contains measurements of PM_2.5_, PM_10_, SO_2_, NO_2_, CO, and O_3_ at high spatial (15 km × 15 km) and temporal (1 h) resolutions for the period 2013–2018 (Chen et al., 2021; Kong et al., 2021). The 5-fold cross-validation indicated its excellent performance in reproducing the magnitude and variability of air pollutants (Kong et al., 2021).

Temperature (°C) and dew point temperature (°C) were accessed from the ERA5-land dataset, which is a gridded reanalysis dataset produced by replaying the land component of the European Center for Medium-Range Weather Forecasts (ECMWF) atmospheric reanalysis dataset of the global climate (ERA5) (Yang et al., 2020). The spatial resolution was 9 km × 9 km, and the temporal resolution was one hour. Relative humidity was calculated using temperature and dew point temperature.

Daily average measurements were obtained by calculating the 24-hour average of the dataset. Based on patients' home addresses, environmental exposure was assessed for each case and control day (Liu et al., 2019). For those without a specific address, the values of the city center were assigned to represent the exposure (Xu et al., 2019).

### 2.4 Cold spell definition

The cold spell metrics and effects on health may vary from region to region (Kent et al., 2014). Previous studies have proposed a series of definitions based on a comprehensive consideration of both temperature thresholds (absolute or relative) and durations (number of consecutive days) (Chen et al., 2019). City-specific relative thresholds (10th, 7.5th, 5th and 2.5th) were calculated based on the distribution of daily mean temperature during the study period. These thresholds allow for a long-term acclimatization (Guo et al., 2017). Simultaneously, three durations of cold spell were considered: ≥2 days, ≥3 days, and ≥4 days. Based on these four city-specific thresholds and three heatwave durations, 12 cold spell definitions were created [denoted as 10th (2d), 10th (3d), 10th (4d), 7.5th (2d), 7.5th (3d), 7.5th (4d), 5th (2d), 5th (3d), 5th (4d), 2.5th (2d), 2.5th (3d), 2.5th (4d)].

### 2.5 Statistical analysis

#### 2.5.1 Basic models

Spearman's correlation was used to examine the correlation between air pollutants and meteorological conditions. And conditional logistic regression was used to build basic models that included all covariates other than air pollutants nor cold spells.

(1) $$\mathrm{logit}\left[P\right]=\mathrm{stratum}\left(i\right)+\mathrm{ns}\;\left(\mathrm{relative humidity},\mathrm{df}=3\right)+\mathrm{factor}\left(\mathrm{PH}\right)+\alpha$$

where P refers to the possibility of outpatient visit for anxiety; to control for the potential nonlinear effect, relative humidity is expressed as a natural cubic spline function with a degrees of freedom (df) of 3 (Lu et al., 2019); PH is referred to public holiday, a binary variable (Xu et al., 2019). By virtue of the case-crossover design, covariates that did not vary with a short period of time (e.g., age) were not included (Liu et al., 2019).

#### 2.5.2 Independent effects of pollutants and cold spell

Based on the basic model, cold spell and air pollution were added to the model to estimate their independent effects.

(2) $$\mathrm{logit}\left[P\right]=\mathrm{stratum}\left(i\right)+\beta *\mathrm{Zt}+\mathrm{CS}+\mathrm{ns}\left(\mathrm{relative humidity},\mathrm{df}=3\right)+\mathrm{factor}\;\left(\mathrm{PH}\right)+\alpha$$

where Z_t_ refers to air pollution concentrations and CS means cold spell. Because the effect of air pollution can last for several days, air pollution concentrations at lag03 were used in the model (Li et al., 2020; Qiu et al., 2019). A single pollutant model was fitted for PM_10_, PM_2.5_, SO_2_, and NO_2_, respectively to avoid the co-linearity. The percent change in odds of anxiety morbidity [defined as (odds ratio – 1) ∗ 100%] and 95% confidence intervals (CIs) associated with each 10 μg/m^3^ increase of pollutant concentrations were estimated. Cold spells were set as a binary value (yes/no) based on each of the 12 cold spell definitions described above, which indicated whether a certain outpatient experienced a cold spell during case or control days.

#### 2.5.3 Interaction effects of air pollution and cold spell

Additive-scale interactions were investigated because interactions estimated as a departure from additivity may be more informative than as departure from multiplicativity in terms of reflecting biological interaction and translating epidemiological results into public health actions (Rothman, 2002). Specifically, the relative excess risk due to interaction (RERI) was calculated, where RERI = 0 indicates the absence of an additive interaction, RERI >0 indicates that the joint effect of air pollution and cold spell is greater than the sum of the effect of each exposure alone (i.e., synergistic effect), and RERI <0 indicates an antagonistic effect (Wang et al., 2020).

The median value of air pollutant concentrations in a city was used as a cut- off point to classify air pollution into a binary variable (greater than the median/less than the median). Then, a new variable was created to represent the combination of air pollution and cold spell. Taking PM_2.5_ as an example, the four levels of the variable were as follows: (a) low PM_2.5_ and no cold spell day (reference); (b) low PM_2.5_ and cold spell day; (c) high PM_2.5_ and no cold spell day; (d) high PM_2.5_ and cold spell day. The RERI can be specified as: RERI = *OR*_11_ − *OR*_01_ − *OR*_10_ + 1, where OR_11_ represents the risk in the level of high PM_2.5_ and cold spell, OR_10_ represents the risk in the level of high PM_2.5_ and no cold spell, and OR_01_ represents the risk in the level of low PM_2.5_ and cold spell. The corresponding 95% CIs were calculated using the delta method (Knol and VanderWeele, 2012).

To identify possible susceptible subgroups, we conducted stratified analyses by sex (male, female) and age (<18, 18–65, and ≥65 years) under different cold spell definitions.

#### 2.5.4 Sensitivity analyses

We performed a series of sensitivity analyses. First, we estimated the additive interaction using a bootstrapping method, in which the pollutant was added in the model as a continuous variable (Knol et al., 2007). Second, air pollution concentrations at lag0 were employed in the models. Third, considering the lagged effect of meteorological factors, relative humidity at lag03 was included in the models. Lastly, effects were examined after excluding outpatients without detailed home addresses.

All analyses were conducted using R software (version 3.6.1). The statistical tests were two-side, and association was considered statistically significant when *p* < 0.05.

## 3 Results

A total of 101,636 outpatient visits for anxiety were identified: 22,039 in Huizhou, 69,446 in Shenzhen and 10,151 in Zhaoqing (Table 1). Almost half of visits were females, and those aged 18–64 years make up the majority (>80%) of visits. The mean exposure to PM_2.5_, PM_10_, NO_2_ and SO_2_ in Huizhou were 51.46 μg/m^3^, 71.39 μg/m^3^, 17.79 μg/m^3^, and 16.66 μg/m^3^, respectively (Table S1), which was slightly higher than in Shenzhen and Zhaoqing. Meteorological conditions were similar in the three cities.

**Table 1:** Characteristics of study population.

	Huizhou	Shenzhen	Zhaoqing
Case days	22,039	69,446	10,151
Control days	74,062	233,723	34,163
Sex, n (%)			
Male	10,168 (46.14)	32,282 (46.49)	4874 (48.01)
Female	11,871 (53.86)	37,164 (53.51)	5277 (51.99)
Age, n (%)			
<18 years	705 (3.20)	2364 (3.40)	252 (2.48)
18–64 years	18,616 (84.47)	61,743 (88.91)	8255 (81.32)
≥65 years	2718 (12.33)	5339 (7.69)	1644 (16.20)

During the cold season (November to April), daily outpatient visits for anxiety ranged across different cold spell definitions and cities. In general, the number of outpatients who experienced cold spells decreased with the intensity of cold spells definitions (Table S2). For example, 13,297 outpatients for anxiety experienced cold spell under the definition of 10th (2d) [daily mean temperature ≤ 14.38 °C (the 10th percentile) for at least 2 consecutive days] in Shenzhen, in comparison to 1985 outpatient visits when the definition was 2.5th (4d) [daily mean temperature ≤ 10.14 °C (the 2.5th percentile) for at least 4 consecutive days]. To allow for a sufficient sample size, only nine cold spell definitions [10th (2d), 10th (3d), 10th (4d), 7.5th (2d), 7.5th (3d), 7.5th (4d), 5th (2d), 5th (3d), and 5th (4d)] in Zhaoqing were used.

PM_2.5_, PM_10_, NO_2_ and SO_2_ were moderately to strongly correlated (correlation coefficient, r > 0.50) (Fig. S2). Daily mean temperature was moderately associated with daily relative humidity (r = 0.55), whereas these were both negatively associated with all four pollutants.

**Fig. 1:**
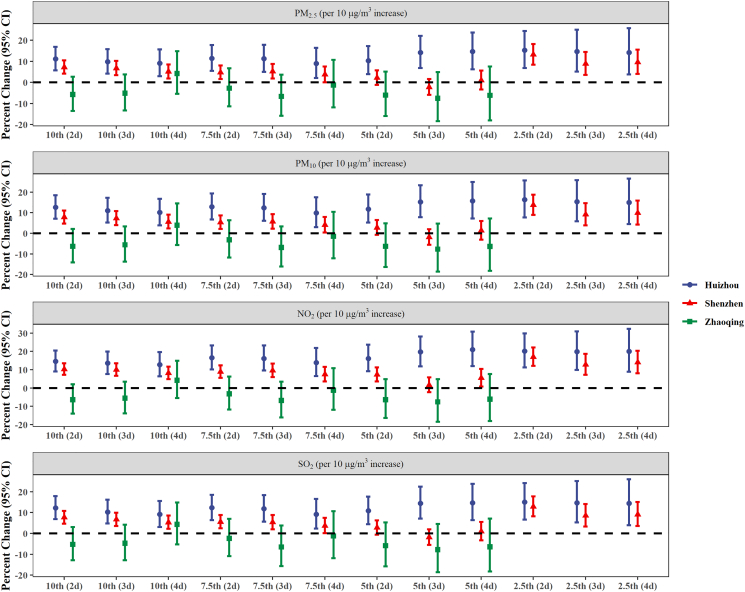
Percent change of outpatients visits for anxiety associated with cold spell exposure in three subtropical Chinese cities. Air pollutants (PM_2.5_, PM_10_, NO_2_ and SO_2_) at lag03 were included in each model separately. Cold spells were defined by percentile temperature thresholds (10th, 7.5th, 5th and 2.5th) and by the number of consecutive days below the thresholds (2–4 d); only nine definitions [10th (2d), 10th (3d), 10th (4d), 7.5th (2d), 7.5th (3d), 7.5th (4d), 5th (2d), 5th (3d), and 5th (4d)] were used in Zhaoqing. Note: PM, particulate matter; SO_2_, sulfur dioxide; NO_2_, nitrogen dioxide.

Fig. 1 shows that exposure to cold spell was significantly associated with outpatient visits for anxiety in Huizhou and Shenzhen when PM_2.5_, PM_10_, NO_2_ and SO_2_ at lag03 were included separately in the model. Under all 12 definitions, an adverse impact of cold spell on anxiety was found. In general, the risk of anxiety outpatient visits increased with the intensity of cold spell (i.e., the estimates increased with lower temperature threshold and longer duration) and was more pronounced in Huizhou. When PM_2.5_ was controlled, the estimates for cold spell ranged from 8.98% (95% CI: 2.02%, 16.41%) to 15.24% (95% CI: 6.75%, 24.39%) in Huizhou, and from −2.22% (95% CI: −5.92%, 1.61%) to 13.22% (95% CI: 8.43%, 18.21%) in Shenzhen. No significant effect of cold spell was found in Zhaoqing. When PM_10_, NO_2_ and SO_2_ were controlled for separately, the estimates showed a similar pattern (Table S3).

Table S4 presents the independent effects of four air pollutants when cold spells under different definitions were controlled. Generally, significant effects of all four pollutants were found, and the estimates were relatively stable when cold spell definition changed. The effect of SO_2_ was consistently significant in all three cities, while estimates for PM_2.5_, PM_10_, and NO_2_ varied across cities. For example, under the definition of 10th (2d), each 10 μg/m^3^ increase of PM_2.5_, PM_10_, NO_2_ and SO_2_ was associated with a 1.51% (95% CI: 0.61%, 2.43%), 1.58% (95% CI: 0.89%, 2.28%), 13.95% (9.98%, 18.05%) and 11.84% (95% CI: 8.25%, 15.55%) increase in outpatient visits for anxiety in Huizhou, whereas in Shenzhen, the estimates were 0.39% (95% CI: −0.11%, 0.89%), 0.55% (95% CI: 0.14%, 0.96%), 12.56% (95% CI: 9.64%, 15.56%), and 5.85% (95% CI: 3.02%, 8.74%), respectively. In Zhaoqing, a positive and significant result was only found for SO_2_ (percent change in odds: 8.86%; 95% CI: 0.23%, 18.24%).

**Fig. 2:**
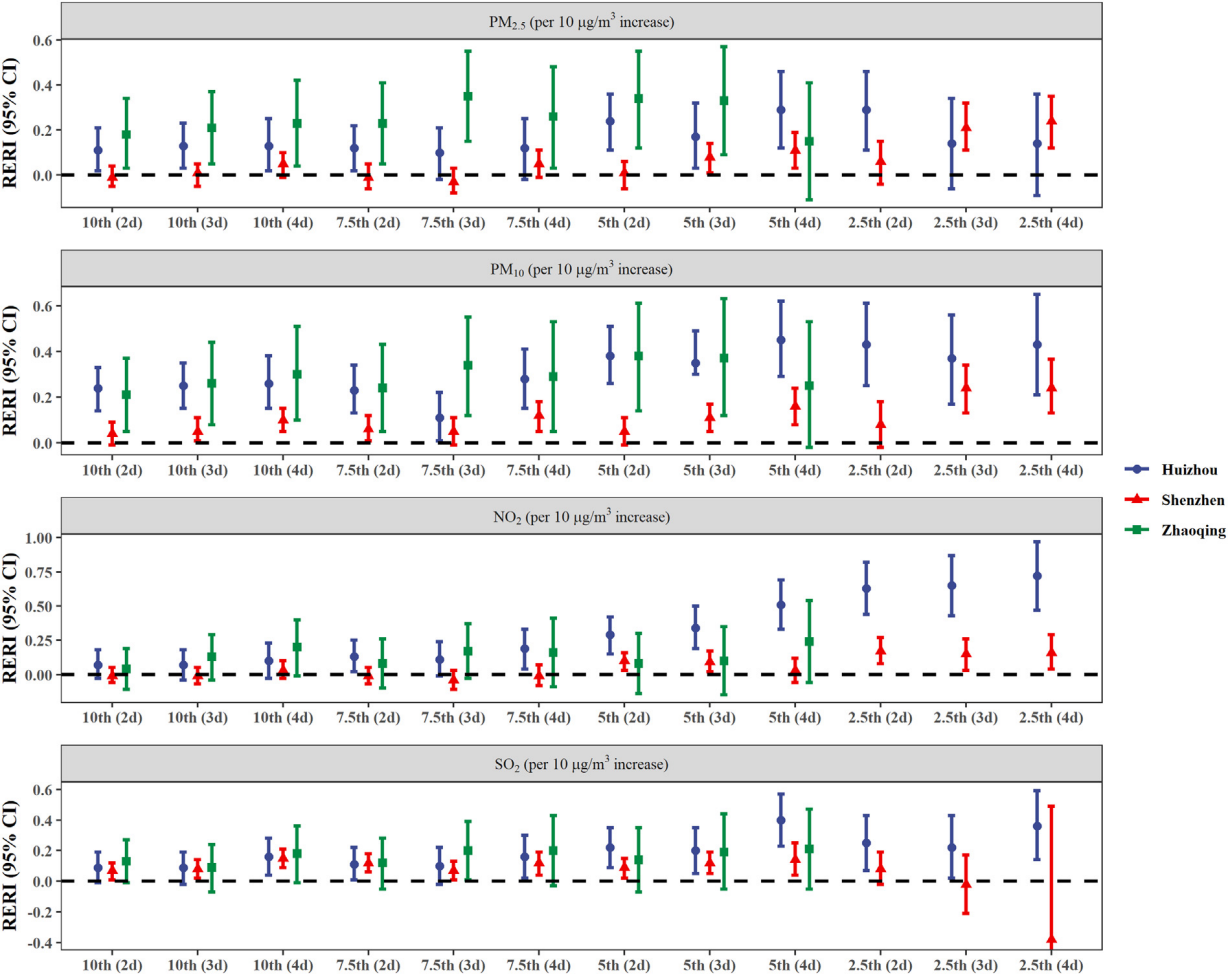
Relative excess risk due to interaction (RERI) of cold spells and air pollutants on anxiety outpatient visits in three subtropical cities in China. Air pollutants were classified as binary variables using the median of air pollutant (lag03) concentrations as a cut off. Cold spells were defined by percentile temperature thresholds (10th, 7.5th, 5th and 2.5th) and by the number of consecutive days below the thresholds (2–4 d); nine definitions [10th (2d), 10th (3d), 10th (4d), 7.5th (2d), 7.5th (3d), 7.5th (4d), 5th (2d), 5th (3d), and 5th (4d)] were used in Zhaoqing. Note: PM, particulate matter; SO_2_, sulfur dioxide; NO_2_, nitrogen dioxide.

We observed synergistic effects (indicated by RERI >0) of cold spell with all four air pollutants on anxiety outpatient visits (Fig. 2 and Table S5), especially for more intense cold spells [5th (2d), 5th (3d), 5th (4d), 2.5th (2d), 2.5th (3d), 2.5th (4d)]. More specifically, interactions between cold spell and PM_2.5_ or PM_10_ were found in all three cities and varied slightly under different cold spell definitions. Additive interactions for NO_2_ were significant only under more intense cold spells [7.5th (4d), 5th (2d), 5th (3d), 5th (4d), 2.5th (2d), 2.5th (3d), 2.5th (4d)] in Huizhou and Shenzhen. The estimates for SO_2_ were relatively consistent under different definitions in Huizhou and Shenzhen. In Zhaoqing, a positive and significant synergistic effect was only observed for 7.5th (3d) [RERI: 0.20 (95% CI: 0.01, 0.39)], suggesting a 20% excess risk compared to the expectation based on the estimated independent effects. When air pollutants were added to models as continuous variables, the analyses yielded similar findings (Table 2).

**Table 2:** Relative excess risk due to interaction (RERI) of cold spells and air pollution on anxiety in three subtropical cities in China.

	PM_2.5_^c^	PM_10_^c^	NO_2_^c^	SO_2_^c^
Huizhou				
10th (2d)^a^	1.95 (1.77, 2.15)	1.92 (1.73, 2.14)	2.35 (1.87, 2.59)	2.13 (1.85, 2.34)
10th (3d)^a^	1.88 (1.71, 2.09)	1.84 (1.63, 2.08)	2.29 (1.77, 2.55)	2.10 (1.83, 2.32)
10th (4d)^a^	1.90 (1.69, 2.13)	1.86 (1.63, 2.12)	2.26 (1.67, 2.55)	2.10 (1.80, 2.32)
7.5th (2d)^a^	1.94 (1.74, 2.15)	1.89 (1.67, 2.13)	2.30 (1.79, 2.55)	2.14 (1.84, 2.36)
7.5th (3d)^a^	1.92 (1.72, 2.15)	1.85 (1.62, 2.11)	2.20 (1.67, 2.54)	2.10 (1.81, 2.33)
7.5th (4d)^a^	1.95 (1.67, 2.24)	1.83 (1.54, 2.15)	2.00 (1.49, 2.44)	2.12 (1.77, 2.41)
5th (2d)^a^	1.86 (1.66, 2.12)	1.69 (1.51, 1.98)	1.87 (1.42, 2.30)	2.06 (1.75, 2.35)
5th (3d)^a^	1.93 (1.66, 2.24)	1.70 (1.47, 2.05)	1.75 (1.18, 2.31)	2.11 (1.74, 2.43)
5th (4d)^a^	1.93 (1.65, 2.27)	1.67 (1.43, 2.05)	1.11 (0.79, 1.99)	2.04 (1.68, 2.40)
2.5th (2d)^a^	1.82 (1.53, 2.15)	1.55 (1.33, 1.93)	1.22 (0.70, 2.17)	2.21 (1.76, 2.51)
2.5th (3d)^a^	1.84 (1.46, 2.27)	1.50 (1.23, 1.93)	0.82 (0.32, 2.01)	2.24 (1.75, 2.59)
2.5th (4d)^a^	1.81 (1.36, 2.31)	1.37 (1.11, 1.82)	0.50 (−0.14, 2.16)	2.00 (1.46, 2.52)

Shenzhen				
10th (2d)^a^	2.00 (1.90, 2.10)	1.97 (1.86, 2.09)	2.22 (1.94, 2.36)	1.73 (1.53, 2.09)
10th (3d)^a^	1.95 (1.84, 2.09)	1.90 (1.80, 2.07)	2.23 (1.89, 2.39)	1.60 (1.41, 1.99)
10th (4d)^a^	1.87 (1.77, 2.03)	1.80 (1.72, 1.98)	2.11 (1.79, 2.31)	1.38 (1.23, 1.87)
7.5th (2d)^a^	1.89 (1.80, 2.05)	1.84 (1.76, 2.01)	2.12 (1.84, 2.32)	1.42 (1.26, 1.91)
7.5th (3d)^a^	1.99 (1.83, 2.15)	1.91 (1.75, 2.10)	2.25 (1.88, 2.44)	1.54 (1.31, 2.03)
7.5th (4d)^a^	1.89 (1.76, 2.08)	1.81 (1.68, 2.03)	2.16 (1.79, 2.41)	1.22 (1.02, 1.87)
5th (2d)^a^	1.81 (1.70, 1.99)	1.77 (1.65, 1.98)	1.82 (1.59, 2.11)	1.34 (1.11, 1.89)
5th (3d)^a^	1.73 (1.64, 1.94)	1.68 (1.58, 1.90)	1.83 (1.54, 2.14)	1.12 (0.89, 1.77)
5th (4d)^a^	1.74 (1.62, 1.98)	1.68 (1.56, 1.94)	2.04 (1.58, 2.49)	0.73 (0.43, 1.76)
2.5th (2d)^a^	1.82 (1.68, 2.09)	1.78 (1.64, 2.05)	1.50 (1.20, 2.07)	1.75 (1.22, 2.42)
2.5th (3d)^a^	1.72 (1.58, 2.02)	1.63 (1.51, 1.93)	1.74 (1.25, 2.35)	1.65 (1.08, 2.44)
2.5th (4d)^a^	1.71 (1.56, 2.03)	1.60 (1.46, 1.94)	0.98 (0.44, 2.25)	1.16 (0.40, 2.58)

Zhaoqing				
10th (2d)^b^	1.49 (1.27, 1.89)	1.46 (1.19, 1.90)	1.44 (0.69, 2.46)	1.40 (0.53, 2.52)
10th (3d)^b^	1.43 (1.20, 1.88)	1.36 (1.11, 1.82)	1.18 (0.42, 2.39)	1.39 (0.43, 2.64)
10th (4d)^b^	1.54 (1.17, 2.04)	1.38 (1.06, 1.89)	0.97 (0.08, 2.64)	1.29 (0.19, 2.81)
7.5th (2d)^b^	1.52 (1.22, 1.98)	1.45 (1.15, 1.91)	1.59 (0.65, 2.69)	1.37 (0.30, 2.75)
7.5th (3d)^b^	1.28 (1.01, 1.80)	1.12 (0.90, 1.61)	1.13 (0.16, 2.51)	0.62 (−0.68, 2.87)
7.5th (4d)^b^	1.48 (1.07, 2.06)	1.28 (0.92, 1.86)	0.92 (−0.28, 2.86)	0.79 (−0.86, 3.32)
5th (2d)^b^	1.46 (1.12, 2.00)	1.35 (0.99, 1.89)	1.70 (0.26, 3.24)	0.54 (−1.14, 3.01)
5th (3d)^b^	1.41 (1.01, 2.01)	1.21 (0.86, 1.78)	1.22 (−0.36, 3.29)	−0.83 (−3.71, 5.04)
5th (4d)^b^	1.69 (0.99, 2.45)	1.52 (0.79, 2.31)	0.24 (−2.02, 4.14)	0.29 (−2.54, 4.63)

Note: PM, particulate matter; SO_2_, sulfur dioxide; NO_2_, nitrogen dioxide.

a Cold spells were defined by percentile temperature thresholds (10th, 7.5th, 5th and 2.5th) and by the number of consecutive days below the thresholds (2–4 d).

b Nine definitions [10th (2d), 10th (3d), 10th (4d), 7.5th (2d), 7.5th (3d), 7.5th (4d), 5th (2d), 5th (3d), and 5th (4d)] were used in Zhaoqing.

c Air pollutants at lag03 were included in each model separately as a continuous variable.

When stratified by age, the independent effects of cold spell, four air pollutants, and their synergistic interactions were relatively consistent across the 12 definitions and three cities for people aged 18–64 years (Tables S6–S9 and Tables S12–S15). Few significant RERIs were found in people aged <18 years and those aged ≥65 years. When stratified by sex, the independent effect estimates of air pollution for males were generally greater than those for females (Tables S10–S11). RERIs of cold spell and four air pollutants showed different patterns in different cities (Tables S12–S15). For example, in Zhaoqing, significant interactions of PM_2.5_ and PM_10_ with cold spell seems to be more pronounced in females, while in Huizhou and Shenzhen, they were similar in two subgroups.

In sensitivity analyses, independent effects of cold spell and air pollutants decreased slightly but remained significant when air pollutants at lag0 were added in models instead of those at lag03; exceptions were for PM_2.5_ in Shenzhen and SO_2_ in Shenzhen and Zhaoqing (Tables S16 and S17). However, the interactions of PM_2.5_ and PM_10_ with cold spell in Zhaoqing were then no longer significant (Table S18). Additionally, when relative humidity at lag03 was included in the models, estimates of independent effects showed similar patterns (Tables S19 and S20), while synergistic effects between NO_2_ and cold spell in Zhaoqing became significant under the definition of 10th (4d) [RERI: 0.22 (95% CI: 0.01, 0.43)] (Table S21). When outpatient visits without detailed home addresses were excluded, the estimated effects in Huizhou and Zhaoqing changed slightly, while most estimates in Shenzhen became insignificant because many cases were eliminated (about 88.95%) (Tables S22–S24).

## 4 Discussion

To our knowledge, this is the first study to simultaneously investigate both the independent effects of cold spell and air pollution exposure and their interactive effects on anxiety. Cold spell was consistently associated with anxiety outpatient visits, and the estimates generally increased with the intensity of cold spell. All four pollutants adversely impacted the onset of anxiety. Additionally, synergistic effects (i.e., RERI >0) of cold spell with four air pollutants on anxiety were observed, particularly for more intense cold spells. Stratified analyses yielded a pronounced results in people aged 18–65 years.

While the adverse impact of unfavorable temperatures on anxiety has been examined previously, most studies focused on heatwaves (Hansen et al., 2008; Wang et al., 2014); studies on the effects of extremely cold temperatures are limited. Our previous case-crossover study suggested that exposure to extremely cold temperature (2.5th) was associated with an increased risk of outpatient visits for anxiety (Zhang et al., 2020). While the association between cold spells and anxiety has been largely unclear. A time-series study in Spain reported a positive and significant impact of cold spell on emergency hospital admissions for anxiety [RR = 1.62 (95% CI: 1.18, 2.22)] (Diaz et al., 2020). This study was limited to its ecological design and single definition of cold wave, which may raise concerns about biased or spurious association. The current study suggests a consistent association and was derived from a large-sample case crossover study with individual-level exposure assessment and a series of cold spell definitions; these aspects of the study provide relatively accurate and convincing estimates.

It is biologically plausible that cold spells may trigger the onset of anxiety. First, it is widely reported that mental disorders are related to a 5-hydroxytryptamine (5-HT) deficiency (Sung et al., 2011). This neurotransmitter is also involved in thermoregulation, which could result in an increased physiological vulnerability to extreme temperatures in patients with anxiety (Hasegawa et al., 2005; Wang et al., 2014). In addition, people in low latitude areas (such as the three subtropical cities studied) are not often exposed to low temperatures. Thus, they would be more susceptible to low ambient temperature (Huang et al., 2015), and this physiological stress may lead to the onset of anxiety.

Building on previous studies (Lu et al., 2020; Song et al., 2018), our study not only found short-term effects of air pollution on anxiety but also found significant synergistic interactions, especially for more intense cold spells. The exact mechanisms of these interactions remain unclear, but their common pathophysiological pathways on mental health may play an important role, since both extreme low temperature and air pollution are associated with inflammatory response and oxidative stress, which could lead to mental disorders (Cai et al., 2016; Li et al., 2020). In addition, cold temperature exposure may reduce mucociliary clearance, which in turn can increase susceptibility to pollutants (Eccles, 2002). Notably, positive and significant interactions with particulate matter were observed under all cold spell definitions, while significant interactions with NO_2_ were only found under more intense definitions. This pattern suggests that even at relatively mild low temperatures, combined effects of cold spell and particulate matter are more than additive for anxiety, and protective measures including using air conditioning and wearing masks should be considered.

The stratified analyses indicate that both independent effects and synergistic interactions between four air pollutants and cold spell were relatively consistent and stable for people aged 18–65 years. This may be due in part to different lifestyles. Middle-aged individuals have more opportunities to be exposed to these environmental exposures (including extreme temperature and air pollution) on their way to and from work, while younger and older individuals are more likely to have decreased exposures due to avoiding outdoor activities and keeping warm under colder conditions. However, there also exists the possibility of limited statistical power to detect the interaction in people aged <18 years and those aged ≥65 years.

This study adds to the literature in several important ways. First, this was the first study to explore the independent and combined effects of cold spell and air pollution on anxiety in subtropical areas with a considerately large sample size. Second, we assessed exposure to air pollution and meteorological factors based on high-resolution gridded reanalysis datasets, which would lead to more accurate exposure estimations and reduce the misclassification. Finally, this study used a time-stratified case-crossover design, which had the ability to control some fixed factors such as age, sex and socioeconomic factors, as well as time-trend bias (Lu et al., 2020). Our sensitivity analyses underscore the robustness of the results.

Despite the above-mentioned strengths, the study also had limitations. Although the exposure assessment method provided more accurate estimates than using the average values, individual time-activity patterns were not considered in the analysis due to the unavailability of data. Additionally, residual confounding is possible even though time-variant confounding factors were controlled for using case crossover design and meteorological factors were included in model. Lastly, cold spell definitions used in this study were relevant to the local climate, the population exposure pattern and adaption capacity, caution should be taken in generalizing results to other regions or countries.

## 5 Conclusion

Study findings indicate that both cold spell and air pollution are important drivers of occurrence of anxiety, and simultaneous exposure to these two factors might have synergistic effects on anxiety. These effects were more pronounced under more intense cold spells definitions and in people aged 18–65 years. These findings provide new evidence on the association between environmental factors and anxiety and highlight the importance of improving cold-warning systems and controlling air pollution.

## CRediT authorship contribution statement

**Huan Li**: Formal analysis, Methodology, Writing - original draft, Writing - review & editing; **Min Li**: Formal analysis, Methodology, Writing - original draft, Writing - review & editing; **Shiyu Zhang**: Data curation, Validation, Writing - review & editing; **Zhengmin (Min) Qian**: Methodology, Writing - review & editing; **Zilong Zhang**: Data curation, Formal analysis, Writing - review & editing**; Kai Zhang**: Methodology, Writing - review & editing**; Chongjian Wang**: Data curation, Formal analysis, Writing - review & editing**; Lauren D. Arnold**: Methodology, Writing - review & editing; **Stephen Edward McMillin**: Methodology, Writing - review & editing**; Shaowei Wu**: Data curation, Formal analysis, Writing - review & editing; **Fei Tian**: Data curation, Validation, Writing - review & editing; **Hualiang Lin**: Conceptualization, Resource, Formal analysis, Writing - original draft, Writing - review & editing.

## Declaration of competing interest

The authors declare that they have no known competing financial interests or personal relationships that could have appeared to influence the work reported in this paper.

## Appendix A Supplementary data

Supplementary material Image 1
