# Supplementary material for: Interactive effects of cold spell and air pollution on outpatient visits for anxiety in three subtropical Chinese cities
Source: Sci Total Environ. 2022 Apr 15;817:152789. doi: 10.1016/j.scitotenv.2021.152789 (PMC8907861; doi:10.1016/j.scitotenv.2021.152789)
Supplement: Supplementary file 1 — Supplementary material [file mmc1.docx]

**Interactive effects of cold spell and air pollution on outpatient visits for anxiety in three subtropical Chinese cities**

Huan Li ^a,1^, Min Li ^b,1^, Shiyu Zhang ^a^, Zhengmin (Min) Qian ^c^, Zilong Zhang ^a^, Kai Zhang ^d^, Chongjian Wang ^e^, Lauren D. Arnold ^c^, Stephen Edward McMillin ^f^, Shaowei Wu ^g^, Fei Tian ^a^, Hualiang Lin ^a, *^

^a^ Department of Epidemiology, School of Public Health, Sun Yat-sen University, Guangzhou 510080, China;

^b^ Department of Preventive Medicine, The Third Affiliated Hospital of Guangzhou University of Chinese Medicine, The Third Clinical Medical Institute Affiliated to Guangzhou University of Chinese Medicine, Guangzhou, China

^c^ Department of Epidemiology and Biostatistics, College for Public Health & Social Justice, Saint Louis University, Saint Louis, USA;

^d^ Department of Environmental Health Sciences, School of Public Health, University at Albany, State University of New York;

^e^ Department of Epidemiology and Biostatistics, College of Public Health, Zhengzhou

University, Zhengzhou, Henan 450001, China;

^f^ School of Social Work, College for Public Health & Social Justice, Saint Louis University, Saint Louis, USA;

^g^ Department of Occupational and Environmental Health, School of Public Health, Xi'an Jiaotong University Health Science Center, Xi'an, Shaanxi 710000, China.

^1^ These authors contributed equally to this work

^*^ Corresponding author:

Hualiang Lin

E-mail: [linhualiang@mail.sysu.edu.cn](mailto:linhualiang@mail.sysu.edu.cn)

Department of Epidemiology, School of Public Health, Sun Yat-sen University, Guangzhou 510080, China.

## Table S1. Distribution of air pollutants and meteorologic conditions during case and control days in three subtropical cities in China.

|  |  |  | Percentile | | | | |
| --- | --- | --- | --- | --- | --- | --- | --- |
|  | Mean | SD | 5th | 25th | 50th | 75th | 95th |
| Huizhou |  |  |  |  |  |  |  |
| Air pollutants |  |  |  |  |  |  |  |
| PM_2.5_, μg/m^3^ | 51.46 | 26.88 | 19.32 | 31.88 | 45.91 | 65.29 | 103.23 |
| PM_10_, μg/m^3^ | 71.39 | 33.14 | 30.54 | 46.15 | 64.32 | 90.46 | 134.96 |
| NO_2_, μg/m^3^ | 17.79 | 8.21 | 7.02 | 11.58 | 16.54 | 22.50 | 32.47 |
| SO_2_, μg/m^3^ | 16.66 | 9.06 | 6.16 | 10.75 | 14.54 | 19.99 | 35.60 |
| Meteorological measures |  |  |  |  |  |  |  |
| Mean temperature (°C) | 21.49 | 5.73 | 10.85 | 17.29 | 22.95 | 26.29 | 28.21 |
| Relative humidity (%) | 78.83 | 11.45 | 56.28 | 72.66 | 81.30 | 87.17 | 93.55 |
| Shenzhen |  |  |  |  |  |  |  |
| Air pollutants |  |  |  |  |  |  |  |
| PM_2.5_, μg/m^3^ | 45.03 | 24.67 | 15.53 | 26.93 | 39.67 | 57.78 | 92.74 |
| PM_10_, μg/m^3^ | 64.35 | 30.03 | 28.60 | 41.65 | 58.60 | 80.60 | 121.59 |
| NO_2_, μg/m^3^ | 15.24 | 6.40 | 6.54 | 10.18 | 14.59 | 18.81 | 26.55 |
| SO_2_, μg/m^3^ | 13.88 | 5.29 | 5.71 | 10.68 | 13.33 | 16.73 | 23.18 |
| Meteorological measures |  |  |  |  |  |  |  |
| Mean temperature (°C) | 22.53 | 5.50 | 12.29 | 18.66 | 23.80 | 27.23 | 28.96 |
| Relative humidity (%) | 79.22 | 10.74 | 57.39 | 74.29 | 81.85 | 86.79 | 92.25 |
| Zhaoqing |  |  |  |  |  |  |  |
| Air pollutants |  |  |  |  |  |  |  |
| PM_2.5_, μg/m^3^ | 47.83 | 22.17 | 19.41 | 32.05 | 43.89 | 59.80 | 90.06 |
| PM_10_, μg/m^3^ | 62.87 | 25.40 | 29.69 | 44.20 | 57.99 | 77.20 | 112.96 |
| NO_2_, μg/m^3^ | 14.83 | 7.25 | 6.65 | 9.93 | 13.25 | 17.63 | 28.10 |
| SO_2_, μg/m^3^ | 14.66 | 4.43 | 8.38 | 11.55 | 14.03 | 17.32 | 22.87 |
| Meteorological measures |  |  |  |  |  |  |  |
| Mean temperature (°C) | 20.54 | 6.05 | 9.07 | 16.18 | 21.91 | 25.78 | 27.58 |
| Relative humidity (%) | 80.76 | 9.81 | 59.97 | 76.30 | 82.95 | 87.53 | 92.95 |

Note: PM, particulate matter; SO_2_, sulfur dioxide; NO_2_, nitrogen dioxide.

## Table S2. Characteristics of cold spell events and number of outpatients experienced cold spell during the study periods in three subtropical cities in China.

|  | Cut off temperature (°C) | Duration (d) | Outpatients who experienced cold spell (N) |
| --- | --- | --- | --- |
| Huizhou |  |  |  |
| 10^th^ (2d) | 12.82 | 2 | 3661 |
| 10^th^ (3d) | 12.82 | 3 | 2995 |
| 10^th^ (4d) | 12.82 | 4 | 2236 |
| 7.5^th^ (2d) | 12.04 | 2 | 2849 |
| 7.5^th^ (3d) | 12.04 | 3 | 2330 |
| 7.5^th^ (4d) | 12.04 | 4 | 1602 |
| 5^th^ (2d) | 10.85 | 2 | 1900 |
| 5^th^ (3d) | 10.85 | 3 | 1452 |
| 5^th^ (4d) | 10.85 | 4 | 1105 |
| 2.5^th^ (2d) | 9.21 | 2 | 1024 |
| 2.5^th^ (3d) | 9.21 | 3 | 753 |
| 2.5^th^ (4d) | 9.21 | 4 | 607 |
| Shenzhen |  |  |  |
| 10^th^ (2d) | 14.38 | 2 | 13297 |
| 10^th^ (3d) | 14.38 | 3 | 10693 |
| 10^th^ (4d) | 14.38 | 4 | 9102 |
| 7.5^th^ (2d) | 13.66 | 2 | 9644 |
| 7.5^th^ (3d) | 13.66 | 3 | 7957 |
| 7.5^th^ (4d) | 13.66 | 4 | 5990 |
| 5^th^ (2d) | 12.29 | 2 | 6740 |
| 5^th^ (3d) | 12.29 | 3 | 5189 |
| 5^th^ (4d) | 12.29 | 4 | 3747 |
| 2.5^th^ (2d) | 10.14 | 2 | 3246 |
| 2.5^th^ (3d) | 10.14 | 3 | 2260 |
| 2.5^th^ (4d) | 10.14 | 4 | 1985 |
| Zhaoqing |  |  |  |
| 10^th^ (2d) | 11.71 | 2 | 1093 |
| 10^th^ (3d) | 11.71 | 3 | 905 |
| 10^th^ (4d) | 11.71 | 4 | 756 |
| 7.5^th^ (2d) | 10.65 | 2 | 806 |
| 7.5^th^ (3d) | 10.65 | 3 | 541 |
| 7.5^th^ (4d) | 10.65 | 4 | 443 |
| 5^th^ (2d) | 9.07 | 2 | 465 |
| 5^th^ (3d) | 9.07 | 3 | 343 |
| 5^th^ (4d) | 9.07 | 4 | 295 |
| 2.5^th^ (2d) | 7.48 | 2 | 150 |
| 2.5^th^ (3d) | 7.48 | 3 | 116 |
| 2.5^th^ (4d) | 7.48 | 4 | 51 |

Note: Cold spells were defined by percentile temperature thresholds (10^th^, 7.5^th^, 5^th^ and 2.5^th^) and by the number of consecutive days below the thresholds (2-4 d).

## Table S3. Independent effects of cold spells on anxiety when PM_2.5_, PM_10_, NO_2_ and SO_2_ at lag03 were included in each model separately.

|  | PM_2.5_ | PM_10_ | NO_2_ | SO_2_ |
| --- | --- | --- | --- | --- |
| Huizhou |  |  |  |  |
| 10^th^ (2d) ^a^ | 11.11 (5.68, 16.82) | 12.57 (7.01, 18.42) | 14.60 (8.98, 20.52) | 12.28 (6.89, 17.93) |
| 10^th^ (3d) ^a^ | 9.77 (4.08, 15.78) | 11.04 (5.24, 17.16) | 13.61 (7.67, 19.87) | 10.31 (4.72, 16.20) |
| 10^th^ (4d) ^a^ | 9.11 (2.94, 15.64) | 10.09 (3.84, 16.71) | 12.83 (6.39, 19.66) | 9.17 (3.11, 15.59) |
| 7.5^th^ (2d) ^a^ | 11.40 (5.43, 17.71) | 12.83 (6.73, 19.28) | 16.59 (10.22, 23.33) | 12.31 (6.41, 18.53) |
| 7.5^th^ (3d) ^a^ | 11.26 (5.03, 17.86) | 12.39 (6.07, 19.08) | 16.19 (9.57, 23.22) | 11.84 (5.68, 18.35) |
| 7.5^th^ (4d) ^a^ | 8.98 (2.02, 16.41) | 9.96 (2.92, 17.48) | 13.96 (6.54, 21.90) | 9.17 (2.29, 16.53) |
| 5^th^ (2d) ^a^ | 10.33 (3.85, 17.21) | 11.75 (5.13, 18.78) | 16.19 (9.17, 23.65) | 10.86 (4.44, 17.67) |
| 5^th^ (3d) ^a^ | 14.16 (6.77, 22.06) | 15.29 (7.79, 23.32) | 19.74 (11.81, 28.22) | 14.50 (7.14, 22.37) |
| 5^th^ (4d) ^a^ | 14.63 (6.23, 23.68) | 15.74 (7.23, 24.92) | 21.00 (11.93, 30.80) | 14.74 (6.38, 23.76) |
| 2.5^th^ (2d) ^a^ | 15.24 (6.75, 24.39) | 16.31 (7.72, 25.59) | 20.15 (11.20, 29.83) | 15.04 (6.62, 24.12) |
| 2.5^th^ (3d) ^a^ | 14.62 (5.16, 24.94) | 15.41 (5.85, 25.82) | 19.96 (9.90, 30.94) | 14.72 (5.25, 25.04) |
| 2.5^th^ (4d) ^a^ | 14.20 (3.75, 25.70) | 14.96 (4.42, 26.56) | 20.03 (8.88, 32.32) | 14.45 (3.98, 25.98) |
| Shenzhen |  |  |  |  |
| 10^th^ (2d) ^a^ | 7.21 (4.13, 10.38) | 7.86 (4.74, 11.07) | 10.34 (7.21, 13.57) | 7.69 (4.69, 10.77) |
| 10^th^ (3d) ^a^ | 6.74 (3.40, 10.17) | 7.34 (3.98, 10.81) | 10.05 (6.67, 13.52) | 6.69 (3.53, 9.94) |
| 10^th^ (4d) ^a^ | 5.15 (1.87, 8.53) | 5.59 (2.29, 8.99) | 8.22 (4.87, 11.68) | 5.33 (2.17, 8.59) |
| 7.5^th^ (2d) ^a^ | 4.76 (1.56, 8.06) | 5.32 (2.08, 8.66) | 8.87 (5.50, 12.34) | 5.57 (2.41, 8.84) |
| 7.5^th^ (3d) ^a^ | 5.22 (1.80, 8.76) | 5.68 (2.22, 9.24) | 9.63 (5.97, 13.42) | 5.35 (2.01, 8.80) |
| 7.5^th^ (4d) ^a^ | 3.73 (0.07, 7.52) | 4.05 (0.37, 7.87) | 7.46 (3.59, 11.48) | 3.71 (0.12, 7.43) |
| 5^th^ (2d) ^a^ | 2.20 (-1.24, 5.77) | 2.76 (-0.73, 6.37) | 7.35 (3.63, 11.20) | 2.81 (-0.57, 6.30) |
| 5^th^ (3d) ^a^ | -2.22 (-5.92, 1.61) | -1.88 (-5.60, 1.99) | 1.68 (-2.27, 5.78) | -1.80 (-5.45, 1.99) |
| 5^th^ (4d) ^a^ | 0.98 (-3.41, 5.56) | 1.31 (-3.09, 5.92) | 5.52 (0.82, 10.44) | 0.96 (-3.36, 5.47) |
| 2.5^th^ (2d) ^a^ | 13.22 (8.43, 18.21) | 13.70 (8.89, 18.73) | 17.03 (12.07, 22.22) | 12.88 (8.18, 17.79) |
| 2.5^th^ (3d) ^a^ | 8.86 (3.58, 14.40) | 9.11 (3.81, 14.67) | 12.80 (7.24, 18.65) | 8.58 (3.34, 14.08) |
| 2.5^th^ (4d) ^a^ | 9.63 (4.00, 15.57) | 9.85 (4.21, 15.81) | 14.03 (8.05, 20.33) | 9.12 (3.53, 15.01) |
| Zhaoqing |  |  |  |  |
| 10^th^ (2d) ^b^ | -5.75 (-13.54, 2.73) | -6.35 (-14.14, 2.14) | -6.34 (-14.06, 2.06) | -5.27 (-12.89, 3.02) |
| 10^th^ (3d) ^b^ | -5.15 (-13.36, 3.83) | -5.61 (-13.82, 3.39) | -5.59 (-13.80, 3.41) | -4.72 (-12.86, 4.18) |
| 10^th^ (4d) ^b^ | 4.20 (-5.40, 14.78) | 3.88 (-5.74, 14.48) | 4.18 (-5.51, 14.87) | 4.35 (-5.20, 14.86) |
| 7.5^th^ (2d) ^b^ | -2.78 (-11.38, 6.65) | -3.19 (-11.80, 6.27) | -3.20 (-11.81, 6.26) | -2.41 (-10.94, 6.93) |
| 7.5^th^ (3d) ^b^ | -6.64 (-15.87, 3.60) | -6.86 (-16.09, 3.39) | -6.79 (-16.06, 3.51) | -6.48 (-15.70, 3.75) |
| 7.5^th^ (4d) ^b^ | -1.26 (-11.89, 10.66) | -1.51 (-12.13, 10.40) | -1.20 (-11.92, 10.83) | -1.22 (-11.83, 10.66) |
| 5^th^ (2d) ^b^ | -6.04 (-16.03, 5.14) | -6.35 (-16.34, 4.84) | -6.35 (-16.34, 4.84) | -5.87 (-15.82, 5.26) |
| 5^th^ (3d) ^b^ | -7.54 (-18.44, 4.81) | -7.71 (-18.60, 4.64) | -7.57 (-18.53, 4.86) | -7.76 (-18.61, 4.53) |
| 5^th^ (4d) ^b^ | -6.15 (-18.04, 7.47) | -6.41 (-18.28, 7.19) | -6.12 (-18.10, 7.62) | -6.38 (-18.21, 7.16) |

Note: PM, particulate matter; SO_2_, sulfur dioxide; NO_2_, nitrogen dioxide.

^a^ Cold spells were defined by percentile temperature thresholds (10^th^, 7.5^th^, 5^th^ and 2.5^th^) and by the number of consecutive days below the thresholds (2-4 d).

^b^ Nine definitions [10^th^ (2d), 10^th^ (3d), 10^th^ (4d), 7.5^th^ (2d), 7.5^th^ (3d), 7.5^th^ (4d), 5^th^ (2d), 5^th^ (3d), and 5^th^ (4d)] were used in Zhaoqing.

## Table S4. Independent effects of air pollution on anxiety when cold spells under different definitions were controlled.

|  | PM_2.5_ ^c^ | PM_10_ ^c^ | NO_2_ ^c^ | SO_2_ ^c^ |
| --- | --- | --- | --- | --- |
| Huizhou |  |  |  |  |
| 10^th^ (2d) ^a^ | 1.51 (0.61, 2.43) | 1.58 (0.89, 2.28) | 13.95 (9.98, 18.05) | 11.84 (8.25, 15.55) |
| 10^th^ (3d) ^a^ | 1.41 (0.51, 2.32) | 1.47 (0.79, 2.16) | 13.58 (9.62, 17.67) | 11.21 (7.67, 14.87) |
| 10^th^ (4d) ^a^ | 1.28 (0.39, 2.18) | 1.34 (0.67, 2.02) | 12.87 (8.99, 16.90) | 10.72 (7.22, 14.34) |
| 7.5^th^ (2d) ^a^ | 1.47 (0.57, 2.39) | 1.53 (0.84, 2.22) | 14.46 (10.42, 18.64) | 11.53 (7.97, 15.22) |
| 7.5^th^ (3d) ^a^ | 1.38 (0.48, 2.28) | 1.43 (0.75, 2.11) | 13.85 (9.88, 17.96) | 11.13 (7.60, 14.77) |
| 7.5^th^ (4d) ^a^ | 1.21 (0.33, 2.11) | 1.28 (0.61, 1.96) | 12.85 (8.95, 16.88) | 10.59 (7.09, 14.20) |
| 5^th^ (2d) ^a^ | 1.30 (0.41, 2.20) | 1.39 (0.71, 2.07) | 13.79 (9.81, 17.91) | 10.89 (7.38, 14.52) |
| 5^th^ (3d) ^a^ | 1.25 (0.37, 2.14) | 1.33 (0.67, 2.00) | 13.37 (9.48, 17.41) | 10.72 (7.23, 14.33) |
| 5^th^ (4d) ^a^ | 1.19 (0.32, 2.08) | 1.28 (0.62, 1.95) | 13.12 (9.24, 17.14) | 10.53 (7.05, 14.13) |
| 2.5^th^ (2d) ^a^ | 1.19 (0.31, 2.07) | 1.28 (0.61, 1.94) | 12.66 (8.83, 16.62) | 10.44 (6.97, 14.03) |
| 2.5^th^ (3d) ^a^ | 1.08 (0.20, 1.95) | 1.18 (0.52, 1.84) | 12.20 (8.41, 16.12) | 10.29 (6.83, 13.88) |
| 2.5^th^ (4d) ^a^ | 1.06 (0.19, 1.94) | 1.16 (0.50, 1.82) | 12.05 (8.26, 15.96) | 10.29 (6.82, 13.87) |
| Shenzhen |  |  |  |  |
| 10^th^ (2d) ^a^ | 0.39 (-0.11, 0.89) | 0.55 (0.14, 0.96) | 12.56 (9.64, 15.56) | 5.85 (3.02, 8.74) |
| 10^th^ (3d) ^a^ | 0.54 (0.03, 1.05) | 0.63 (0.21, 1.05) | 11.97 (9.01, 15.02) | 5.70 (2.87, 8.60) |
| 10^th^ (4d) ^a^ | 0.39 (-0.11, 0.89) | 0.48 (0.07, 0.89) | 10.98 (8.07, 13.96) | 5.10 (2.29, 7.99) |
| 7.5^th^ (2d) ^a^ | 0.21 (-0.28, 0.70) | 0.39 (-0.01, 0.80) | 12.22 (9.28, 15.24) | 5.35 (2.55, 8.23) |
| 7.5^th^ (3d) ^a^ | 0.34 (-0.15, 0.83) | 0.45 (0.05, 0.86) | 11.67 (8.71, 14.71) | 5.03 (2.25, 7.88) |
| 7.5^th^ (4d) ^a^ | 0.24 (-0.24, 0.73) | 0.35 (-0.05, 0.75) | 10.41 (7.53, 13.36) | 4.45 (1.69, 7.28) |
| 5^th^ (2d) ^a^ | 0.09 (-0.40, 0.58) | 0.29 (-0.12, 0.69) | 11.84 (8.87, 14.89) | 4.69 (1.93, 7.52) |
| 5^th^ (3d) ^a^ | 0.09 (-0.39, 0.57) | 0.23 (-0.17, 0.63) | 9.34 (6.48, 12.29) | 4.36 (1.61, 7.19) |
| 5^th^ (4d) ^a^ | 0.16 (-0.32, 0.64) | 0.28 (-0.12, 0.67) | 9.78 (6.92, 12.73) | 4.30 (1.55, 7.12) |
| 2.5^th^ (2d) ^a^ | 0.24 (-0.24, 0.72) | 0.40 (0.01, 0.79) | 11.77 (8.92, 14.68) | 4.45 (1.72, 7.25) |
| 2.5^th^ (3d) ^a^ | 0.22 (-0.25, 0.70) | 0.34 (-0.05, 0.73) | 10.28 (7.47, 13.16) | 4.46 (1.72, 7.28) |
| 2.5^th^ (4d) ^a^ | 0.21 (-0.27, 0.69) | 0.32 (-0.07, 0.71) | 10.19 (7.37, 13.07) | 4.12 (1.38, 6.94) |
| Zhaoqing |  |  |  |  |
| 10^th^ (2d) ^b^ | 0.60 (-0.81, 2.04) | 0.20 (-1.00, 1.41) | 0.85 (-3.68, 5.60) | 8.86 (0.23, 18.24) |
| 10^th^ (3d) ^b^ | 0.69 (-0.70, 2.09) | 0.27 (-0.90, 1.46) | 1.10 (-3.40, 5.81) | 8.84 (0.24, 18.19) |
| 10^th^ (4d) ^b^ | 0.84 (-0.54, 2.23) | 0.42 (-0.74, 1.60) | 2.10 (-2.40, 6.81) | 8.71 (0.15, 17.99) |
| 7.5^th^ (2d) ^b^ | 0.80 (-0.58, 2.21) | 0.39 (-0.79, 1.57) | 1.48 (-3.03, 6.19) | 9.58 (0.94, 18.96) |
| 7.5^th^ (3d) ^b^ | 0.77 (-0.59, 2.15) | 0.35 (-0.80, 1.51) | 1.33 (-3.10, 5.95) | 9.18 (0.63, 18.45) |
| 7.5^th^ (4d) ^b^ | 0.72 (-0.63, 2.10) | 0.32 (-0.83, 1.48) | 1.61 (-2.83, 6.26) | 8.20 (-0.28, 17.40) |
| 5^th^ (2d) ^b^ | 0.78 (-0.58, 2.17) | 0.37 (-0.78, 1.54) | 1.43 (-2.99, 6.06) | 9.51 (0.94, 18.81) |
| 5^th^ (3d) ^b^ | 0.80 (-0.55, 2.17) | 0.38 (-0.76, 1.54) | 1.42 (-2.99, 6.04) | 9.46 (0.92, 18.72) |
| 5^th^ (4d) ^b^ | 0.68 (-0.68, 2.06) | 0.28 (-0.87, 1.43) | 1.36 (-3.06, 5.99) | 8.13 (-0.33, 17.31) |

Note: PM, particulate matter; SO_2_, sulfur dioxide; NO_2_, nitrogen dioxide.

^a^ Cold spells were defined by percentile temperature thresholds (10^th^, 7.5^th^, 5^th^ and 2.5^th^) and by the number of consecutive days below the thresholds (2-4 d).

^b^ Nine definitions [10^th^ (2d), 10^th^ (3d), 10^th^ (4d), 7.5^th^ (2d), 7.5^th^ (3d), 7.5^th^ (4d), 5^th^ (2d), 5^th^ (3d), and 5^th^ (4d)] were used in Zhaoqing.

^c^ Air pollutants at lag03 were included in each model separately.

## Table S5. Percent changes and relative excess risk due to interaction (RERI) of cold spells and air pollution (lag03) on anxiety in three subtropical cities in China.

| Cold spells | PM_2.5_ ^c^ (μg/m^3^) | Percent Change  (95% CI) | PM_10_ ^c^ (μg/m^3^) | Percent Change  (95% CI) | NO_2_ ^c^ (μg/m^3^) | Percent Change  (95% CI) | SO_2_ ^c^ (μg/m^3^) | Percent Change  (95% CI) |
| --- | --- | --- | --- | --- | --- | --- | --- | --- |
| Huizhou |  |  |  |  |  |  |  |  |
| 10^th^ (2d) ^a^ |  |  |  |  |  |  |  |  |
| No | < 46.03 | 1.00 | < 64.71 | 1.00 | < 16.87 | 1.00 | < 14.48 | 1.00 |
| No | ≥ 46.03 | 4.36 (0.13, 8.77) | ≥ 64.71 | 3.55 (-0.34, 7.60) | ≥ 16.87 | 14.42 (8.75, 20.38) | ≥ 14.48 | 6.96 (2.73, 11.36) |
| Yes | < 46.03 | 4.02 (-3.09, 11.65) | < 64.71 | -1.82 (-8.54, 5.40) | < 16.87 | 7.39 (-0.94, 16.41) | < 14.48 | 4.54 (-2.98, 12.63) |
| Yes | ≥ 46.03 | 19.78 (11.83, 28.30) | ≥ 64.71 | 25.46 (17.20, 34.30) | ≥ 16.87 | 29.05 (20.15, 38.61) | ≥ 14.48 | 20.78 (12.97, 29.12) |
| RERI |  | 0.11 (0.02, 0.21) |  | 0.24 (0.14, 0.33) |  | 0.07 (-0.03, 0.18) |  | 0.09 (-0.01, 0.19) |
| 10^th^ (3d) ^a^ |  |  |  |  |  |  |  |  |
| No | < 46.03 | 1.00 | < 64.71 | 1.00 | < 16.87 | 1.00 | < 14.48 | 1.00 |
| No | ≥ 46.03 | 4.04 (-0.08, 8.32) | ≥ 64.71 | 3.35 (-0.44, 7.29) | ≥ 16.87 | 14.12 (8.54, 19.99) | ≥ 14.48 | 6.87 (2.71, 11.20) |
| Yes | < 46.03 | 1.66 (-5.82, 9.73) | < 64.71 | -5.02 (-12.10, 2.62) | < 16.87 | 5.75 (-3.30, 15.65) | < 14.48 | 3.00 (-5.09, 11.77) |
| Yes | ≥ 46.03 | 18.38 (10.23, 27.13) | ≥ 64.71 | 23.54 (15.23, 32.45) | ≥ 16.87 | 26.85 (17.90, 36.48) | ≥ 14.48 | 18.60 (10.74, 27.02) |
| RERI |  | 0.13 (0.03, 0.23) |  | 0.25 (0.15, 0.35) |  | 0.07 (-0.04, 0.18) |  | 0.09 (-0.02, 0.19) |
| 10^th^ (4d) ^a^ |  |  |  |  |  |  |  |  |
| No | < 46.03 | 1.00 | < 64.71 | 1.00 | < 16.87 | 1.00 | < 14.48 | 1.00 |
| No | ≥ 46.03 | 4.11 (0.11, 8.27) | ≥ 64.71 | 3.67 (-0.02, 7.49) | ≥ 16.87 | 13.63 (8.19, 19.34) | ≥ 14.48 | 6.16 (2.09, 10.40) |
| Yes | < 46.03 | 0.64 (-7.60, 9.63) | < 64.71 | -7.32 (-15.24, 1.34) | < 16.87 | 3.66 (-6.23, 14.60) | < 14.48 | -2.03 (-10.76, 7.56) |
| Yes | ≥ 46.03 | 18.02 (9.32, 27.40) | ≥ 64.71 | 22.83 (14.10, 32.22) | ≥ 16.87 | 27.16 (17.54, 37.56) | ≥ 14.48 | 19.91 (11.40, 29.07) |
| RERI |  | 0.13 (0.02, 0.25) |  | 0.26 (0.15, 0.38) |  | 0.10 (-0.03, 0.23) |  | 0.16 (0.04, 0.28) |
| 7.5^th^ (2d) ^a^ |  |  |  |  |  |  |  |  |
| No | < 46.03 | 1.00 | < 64.71 | 1.00 | < 16.87 | 1.00 | < 14.48 | 1.00 |
| No | ≥ 46.03 | 4.31 (0.21, 8.58) | ≥ 64.71 | 4.02 (0.23, 7.94) | ≥ 16.87 | 13.33 (7.80, 19.16) | ≥ 14.48 | 7.01 (2.85, 11.34) |
| Yes | < 46.03 | 3.26 (-4.51, 11.68) | < 64.71 | -2.61 (-10.05, 5.45) | < 16.87 | 3.86 (-5.06, 13.62) | < 14.48 | 3.92 (-4.05, 12.54) |
| Yes | ≥ 46.03 | 19.77 (11.38, 28.79) | ≥ 64.71 | 24.79 (16.18, 34.04) | ≥ 16.87 | 30.58 (21.04, 40.88) | ≥ 14.48 | 22.38 (13.73, 31.69) |
| RERI |  | 0.12 (0.02, 0.22) |  | 0.23 (0.13, 0.34) |  | 0.13 (0.02, 0.25) |  | 0.11 (0.01, 0.22) |
| 7.5^th^ (3d) ^a^ |  |  |  |  |  |  |  |  |
| No | < 46.03 | 1.00 | < 64.71 | 1.00 | < 16.87 | 1.00 | < 14.48 | 1.00 |
| No | ≥ 46.03 | 4.70 (0.66, 8.89) | ≥ 64.71 | 4.37 (0.62, 8.26) | ≥ 16.87 | 13.76 (8.29, 19.51) | ≥ 14.48 | 7.11 (2.99, 11.40) |
| Yes | < 46.03 | 4.78 (-3.60, 13.88) | < 64.71 | 2.94 (-5.58, 12.23) | < 16.87 | 5.13 (-4.53, 15.77) | < 14.48 | 4.38 (-4.34, 13.90) |
| Yes | ≥ 46.03 | 19.10 (10.40, 28.49) | ≥ 64.71 | 18.49 (10.24, 27.35) | ≥ 16.87 | 30.27 (20.48, 40.87) | ≥ 14.48 | 21.66 (12.81, 31.21) |
| RERI |  | 0.10 (-0.02, 0.21) |  | 0.11 (0.01, 0.22) |  | 0.11 (-0.01, 0.24) |  | 0.10 (-0.02, 0.22) |
| 7.5^th^ (4d) ^a^ |  |  |  | lag03 |  |  |  |  |
| No | < 46.03 | 1.00 | < 64.71 | 1.00 | < 16.87 | 1.00 | < 14.48 | 1.00 |
| No | ≥ 46.03 | 4.50 (0.56, 8.61) | ≥ 64.71 | 4.15 (0.52, 7.92) | ≥ 16.87 | 12.68 (7.37, 18.24) | ≥ 14.48 | 6.58 (2.52, 10.80) |
| Yes | < 46.03 | 1.45 (-8.09, 11.98) | < 64.71 | -9.13 (-18.27, 1.03) | < 16.87 | -1.58 (-12.17, 10.28) | < 14.48 | -1.39 (-11.16, 9.45) |
| Yes | ≥ 46.03 | 17.48 (7.86, 27.95) | ≥ 64.71 | 23.27 (13.59, 33.77) | ≥ 16.87 | 29.69 (18.88, 41.48) | ≥ 14.48 | 21.29 (11.45, 32.00) |
| RERI |  | 0.12 (-0.02, 0.25) |  | 0.28 (0.15, 0.41) |  | 0.19 (0.04, 0.33) |  | 0.16 (0.02, 0.30) |
| 5^th^ (2d) ^a^ |  |  |  |  |  |  |  |  |
| No | < 46.03 | 1.00 | < 64.71 | 1.00 | < 16.87 | 1.00 | < 14.48 | 1.00 |
| No | ≥ 46.03 | 3.27 (-0.69, 7.38) | ≥ 64.71 | 3.29 (-0.37, 7.10) | ≥ 16.87 | 11.39 (6.04, 17.01) | ≥ 14.48 | 6.22 (2.15, 10.45) |
| Yes | < 46.03 | -2.42 (-10.46, 6.35) | < 64.71 | -9.02 (-16.65, -0.70) | < 16.87 | -3.90 (-12.74, 5.85) | < 14.48 | -1.17 (-9.36, 7.76) |
| Yes | ≥ 46.03 | 24.76 (14.82, 35.57) | ≥ 64.71 | 32.76 (22.34, 44.07) | ≥ 16.87 | 36.20 (25.20, 48.17) | ≥ 14.48 | 27.42 (17.13, 38.62) |
| RERI |  | 0.24 (0.11, 0.36) |  | 0.38 (0.26, 0.51) |  | 0.29 (0.15, 0.42) |  | 0.22 (0.09, 0.35) |
| 5^th^ (3d) ^a^ |  |  |  |  |  |  |  |  |
| No | < 46.03 | 1.00 | < 64.71 | 1.00 | < 16.87 | 1.00 | < 14.48 | 1.00 |
| No | ≥ 46.03 | 4.25 (0.34, 8.31) | ≥ 64.71 | 4.27 (0.65, 8.02) | ≥ 16.87 | 11.47 (6.24, 16.96) | ≥ 14.48 | 6.75 (2.69, 10.96) |
| Yes | < 46.03 | 4.06 (-5.85, 15.02) | < 64.71 | -5.39 (-14.71, 4.94) | < 16.87 | -4.65 (-15.07, 7.04) | < 14.48 | 3.35 (-6.63, 14.38) |
| Yes | ≥ 46.03 | 25.57 (14.86, 37.27) | ≥ 64.71 | 33.78 (22.63, 45.95) | ≥ 16.87 | 41.26 (29.20, 54.44) | ≥ 14.48 | 30.30 (18.95, 42.72) |
| RERI |  | 0.17 (0.03, 0.32) |  | 0.35 (0.30, 0.49) |  | 0.34 (0.19, 0.50) |  | 0.20 (0.05, 0.35) |
| 5^th^ (4d) ^a^ |  |  |  |  |  |  |  |  |
| No | < 46.03 | 1.00 | < 64.71 | 1.00 | < 16.87 | 1.00 | < 14.48 | 1.00 |
| No | ≥ 46.03 | 4.00 (0.13, 8.03) | ≥ 64.71 | 4.07 (0.48, 7.78) | ≥ 16.87 | 10.63 (5.48, 16.05) | ≥ 14.48 | 5.82 (1.83, 9.97) |
| Yes | < 46.03 | -0.31 (-10.82, 11.44) | < 64.71 | -10.50 (-20.68, 0.98) | < 16.87 | -10.80 (-21.46, 1.30) | < 14.48 | -6.13 (-16.57, 5.63) |
| Yes | ≥ 46.03 | 32.65 (19.72, 46.98) | ≥ 64.71 | 38.73 (25.86, 52.91) | ≥ 16.87 | 51.14 (36.50, 67.34) | ≥ 14.48 | 39.69 (26.20, 54.62) |
| RERI |  | 0.29 (0.12, 0.46) |  | 0.45 (0.29, 0.62) |  | 0.51 (0.33, 0.69) |  | 0.40 (0.23, 0.57) |
| 2.5^th^ (2d) ^a^ |  |  |  |  |  |  |  |  |
| No | < 46.03 | 1.00 | < 64.71 | 1.00 | < 16.87 | 1.00 | < 14.48 | 1.00 |
| No | ≥ 46.03 | 3.97 (0.11, 7.98) | ≥ 64.71 | 4.50 (0.91, 8.22) | ≥ 16.87 | 9.66 (4.54, 15.04) | ≥ 14.48 | 6.80 (2.80, 10.95) |
| Yes | < 46.03 | 1.44 (-8.99, 13.07) | < 64.71 | -6.57 (-16.85, 4.98) | < 16.87 | -12.82 (-22.79, -1.55) | < 14.48 | 2.23 (-8.94, 14.77) |
| Yes | ≥ 46.03 | 34.19 (20.52, 49.41) | ≥ 64.71 | 40.79 (27.07, 55.99) | ≥ 16.87 | 59.81 (43.88, 77.51) | ≥ 14.48 | 34.10 (20.75, 48.92) |
| RERI |  | 0.29 (0.11, 0.46) |  | 0.43 (0.25, 0.61) |  | 0.63 (0.44, 0.82) |  | 0.25 (0.07, 0.43) |
| 2.5^th^ (3d) ^a^ |  |  |  |  |  |  |  |  |
| No | < 46.03 | 1.00 | < 64.71 | 1.00 | < 16.87 | 1.00 | < 14.48 | 1.00 |
| No | ≥ 46.03 | 4.64 (0.81, 8.62) | ≥ 64.71 | 5.03 (1.46, 8.72) | ≥ 16.87 | 10.81 (5.73, 16.13) | ≥ 14.48 | 7.11 (3.13, 11.24) |
| Yes | < 46.03 | 6.67 (-6.73, 21.99) | < 64.71 | -7.70 (-20.46, 7.11) | < 16.87 | -12.44 (-23.59, 0.35) | < 14.48 | 3.64 (-9.20, 18.30) |
| Yes | ≥ 46.03 | 24.91 (11.37, 40.09) | ≥ 64.71 | 33.95 (20.20, 49.29) | ≥ 16.87 | 63.49 (45.01, 84.33) | ≥ 14.48 | 32.78 (18.04, 49.37) |
| RERI |  | 0.14 (-0.06, 0.34) |  | 0.37 (0.17, 0.56) |  | 0.65 (0.43, 0.87) |  | 0.22 (0.02, 0.43) |
| 2.5^th^ (4d) ^a^ |  |  |  |  |  |  |  |  |
| No | < 46.03 | 1.00 | < 64.71 | 1.00 | < 16.87 | 1.00 | < 14.48 | 1.00 |
| No | ≥ 46.03 | 4.78 (0.95, 8.75) | ≥ 64.71 | 4.99 (1.44, 8.68) | ≥ 16.87 | 11.02 (5.97, 16.30) | ≥ 14.48 | 6.66 (2.71, 10.77) |
| Yes | < 46.03 | 6.19 (-8.64, 23.44) | < 64.71 | -12.88 (-26.76, 3.63) | < 16.87 | -16.36 (-28.33, -2.38) | < 14.48 | -5.90 (-19.75, 10.33) |
| Yes | ≥ 46.03 | 24.61 (9.79, 41.44) | ≥ 64.71 | 34.98 (19.97, 51.86) | ≥ 16.87 | 67.07 (46.40, 90.66) | ≥ 14.48 | 37.06 (20.99, 55.27) |
| RERI |  | 0.14 (-0.09, 0.36) |  | 0.43 (0.21, 0.65) |  | 0.72 (0.47, 0.97) |  | 0.36 (0.14, 0.59) |
| Shenzhen |  |  |  |  |  |  |  |  |
| 10^th^ (2d) ^a^ |  |  |  |  |  |  |  |  |
| No | < 39.46 | 1.00 | < 58.15 | 1.00 | < 14.71 | 1.00 | < 13.27 | 1.00 |
| No | ≥ 39.46 | 1.70 (-0.40, 3.85) | ≥ 58.15 | 1.67 (-0.49, 3.87) | ≥ 14.71 | 11.30 (8.31, 14.36) | ≥ 13.27 | 1.54 (-0.76, 3.89) |
| Yes | < 39.46 | 7.36 (3.56, 11.30) | < 58.15 | 5.70 (1.94, 9.61) | < 14.71 | 8.82 (4.01, 13.87) | < 13.27 | 2.40 (-2.03, 7.03) |
| Yes | ≥ 39.46 | 8.45 (4.30, 12.77) | ≥ 58.15 | 11.24 (6.83, 15.84) | ≥ 14.71 | 19.71 (15.11, 24.51) | ≥ 13.27 | 10.75 (6.72, 14.94) |
| RERI |  | -0.01 (-0.05, 0.04) |  | 0.04 (-0.01, 0.09) |  | -0.01 (-0.06, 0.05) |  | 0.07 (0.01, 0.12) |
| 10^th^ (3d) ^a^ |  |  |  |  |  |  |  |  |
| No | < 39.46 | 1.00 | < 58.15 | 1.00 | < 14.71 | 1.00 | < 13.27 | 1.00 |
| No | ≥ 39.46 | 2.10 (0.02, 4.22) | ≥ 58.15 | 1.90 (-0.23, 4.07) | ≥ 14.71 | 10.98 (8.00, 14.05) | ≥ 13.27 | 1.34 (-0.92, 3.66) |
| Yes | < 39.46 | 6.04 (1.93, 10.33) | < 58.15 | 3.92 (-0.12, 8.12) | < 14.71 | 8.22 (2.60, 14.15) | < 13.27 | 0.75 (-3.88, 5.60) |
| Yes | ≥ 39.46 | 8.37 (4.10, 12.81) | ≥ 58.15 | 11.29 (6.73, 16.05) | ≥ 14.71 | 18.11 (13.42, 22.99) | ≥ 13.27 | 10.45 (6.06, 15.02) |
| RERI |  | 0.01 (-0.05, 0.05) |  | 0.05 (0.01, 0.11) |  | -0.01 (-0.07, 0.05) |  | 0.08 (0.02, 0.14) |
| 10^th^ (4d) ^a^ |  |  |  |  |  |  |  |  |
| No | < 39.46 | 1.00 | < 58.15 | 1.00 | < 14.71 | 1.00 | < 13.27 | 1.00 |
| No | ≥ 39.46 | 0.93 (-1.07, 2.97) | ≥ 58.15 | 0.76 (-1.29, 2.85) | ≥ 14.71 | 9.39 (6.53, 12.34) | ≥ 13.27 | 0.52 (-1.71, 2.80) |
| Yes | < 39.46 | 2.03 (-2.10, 6.33) | < 58.15 | -0.00 (-4.04, 4.20) | < 14.71 | 3.28 (-2.35, 9.23) | < 13.27 | -4.45 (-9.08, 0.41) |
| Yes | ≥ 39.46 | 7.88 (3.66, 12.28) | ≥ 58.15 | 10.70 (6.18, 15.42) | ≥ 14.71 | 15.97 (11.37, 20.76) | ≥ 13.27 | 10.75 (6.26, 15.42) |
| RERI |  | 0.05 (-0.01, 0.10) |  | 0.10 (0.05, 0.15) |  | 0.03 (-0.03, 0.10) |  | 0.15 (0.09, 0.21) |
| 7.5^th^ (2d) ^a^ |  |  |  |  |  |  |  |  |
| No | < 39.46 | 1.00 | < 58.15 | 1.00 | < 14.71 | 1.00 | < 13.27 | 1.00 |
| No | ≥ 39.46 | 1.17 (-0.86, 3.24) | ≥ 58.15 | 0.76 (-1.31, 2.87) | ≥ 14.71 | 11.60 (8.58, 14.69) | ≥ 13.27 | 0.96 (-1.26, 3.24) |
| Yes | < 39.46 | 5.11 (0.88, 9.52) | < 58.15 | 1.78 (-2.33, 6.06) | < 14.71 | 8.11 (2.90, 13.59) | < 13.27 | -2.83 (-7.41, 1.97) |
| Yes | ≥ 39.46 | 5.52 (1.32, 9.89) | ≥ 58.15 | 8.96 (4.46, 13.66) | ≥ 14.71 | 18.72 (13.73, 23.94) | ≥ 13.27 | 10.22 (5.88, 14.74) |
| RERI |  | -0.01 (-0.06, 0.05) |  | 0.06 (0.01, 0.12) |  | -0.01 (-0.07, 0.05) |  | 0.12 (0.06, 0.18) |
| 7.5^th^ (3d) ^a^ |  |  |  |  |  |  |  |  |
| No | < 39.46 | 1.00 | < 58.15 | 1.00 | < 14.71 | 1.00 | < 13.27 | 1.00 |
| No | ≥ 39.46 | 1.96 (-0.02, 3.97) | ≥ 58.15 | 1.54 (-0.47, 3.59) | ≥ 14.71 | 11.70 (8.73, 14.74) | ≥ 13.27 | 1.55 (-0.66, 3.82) |
| Yes | < 39.46 | 6.41 (1.70, 11.35) | < 58.15 | 2.22 (-2.26, 6.90) | < 14.71 | 10.08 (4.50, 15.96) | < 13.27 | 0.67 (-4.54, 6.16) |
| Yes | ≥ 39.46 | 5.76 (1.51, 10.18) | ≥ 58.15 | 9.20 (4.65, 13.94) | ≥ 14.71 | 17.88 (12.69, 23.30) | ≥ 13.27 | 8.81 (4.31, 13.51) |
| RERI |  | -0.03 (-0.08, 0.03) |  | 0.05 (-0.01, 0.11) |  | -0.04 (-0.11, 0.03) |  | 0.07 (0.01, 0.13) |
| 7.5^th^ (4d) ^a^ |  |  |  |  |  |  |  |  |
| No | < 39.46 | 1.00 | < 58.15 | 1.00 | < 14.71 | 1.00 | < 13.27 | 1.00 |
| No | ≥ 39.46 | 0.86 (-1.06, 2.81) | ≥ 58.15 | 0.67 (-1.28, 2.66) | ≥ 14.71 | 10.01 (7.18, 12.92) | ≥ 13.27 | 1.14 (-1.04, 3.36) |
| Yes | < 39.46 | 0.11 (-5.19, 5.71) | < 58.15 | -3.21 (-8.11, 1.95) | < 14.71 | 5.57 (-0.41, 11.90) | < 13.27 | -5.29 (-11.61, 1.48) |
| Yes | ≥ 39.46 | 6.00 (1.58, 10.60) | ≥ 58.15 | 9.22 (4.44, 14.23) | ≥ 14.71 | 14.80 (9.54, 20.32) | ≥ 13.27 | 7.72 (3.10, 12.55) |
| RERI |  | 0.05 (-0.01, 0.11) |  | 0.12 (0.05, 0.18) |  | -0.01 (-0.08, 0.07) |  | 0.12 (0.04, 0.19) |
| 5^th^ (2d) ^a^ |  |  |  | lag03 |  |  |  | lag03 |
| No | < 39.46 | 1.00 | < 58.15 | 1.00 | < 14.71 | 1.00 | < 13.27 | 1.00 |
| No | ≥ 39.46 | 0.70 (-1.24, 2.68) | ≥ 58.15 | 0.80 (-1.20, 2.83) | ≥ 14.71 | 9.09 (6.25, 12.00) | ≥ 13.27 | 1.45 (-0.75, 3.70) |
| Yes | < 39.46 | 2.16 (-2.49, 7.04) | < 58.15 | 0.05 (-4.37, 4.67) | < 14.71 | -2.25 (-7.60, 3.41) | < 13.27 | -3.62 (-8.91, 1.98) |
| Yes | ≥ 39.46 | 2.88 (-1.49, 7.45) | ≥ 58.15 | 5.81 (1.00, 10.84) | ≥ 14.71 | 16.49 (11.38, 21.83) | ≥ 13.27 | 6.63 (2.17, 11.29) |
| RERI |  | 0.01 (-0.06, 0.06) |  | 0.05 (-0.01, 0.11) |  | 0.10 (0.03, 0.16) |  | 0.09 (0.02, 0.15) |
| 5^th^ (3d) ^a^ |  | lag03 |  |  |  |  |  |  |
| No | < 39.46 | 1.00 | < 58.15 | 1.00 | < 14.71 | 1.00 | < 13.27 | 1.00 |
| No | ≥ 39.46 | 0.72 (-1.17, 2.65) | ≥ 58.15 | 0.68 (-1.26, 2.65) | ≥ 14.71 | 8.67 (5.88, 11.53) | ≥ 13.27 | 0.90 (-1.26, 3.11) |
| Yes | < 39.46 | -7.54 (-12.82, -1.93) | < 58.15 | -8.32 (-13.20, -3.17) | < 14.71 | -7.83 (-13.77, -1.47) | < 13.27 | -10.84 (-16.77, -4.48) |
| Yes | ≥ 39.46 | 1.01 (-3.41, 5.64) | ≥ 58.15 | 3.25 (-1.58, 8.32) | ≥ 14.71 | 10.24 (5.07, 15.66) | ≥ 13.27 | 2.05 (-2.59, 6.92) |
| RERI |  | 0.08 (0.01, 0.14) |  | 0.11 (0.05, 0.17) |  | 0.09 (0.02, 0.17) |  | 0.12 (0.05, 0.19) |
| 5^th^ (4d) ^a^ |  |  |  |  |  |  |  |  |
| No | < 39.46 | 1.00 | < 58.15 | 1.00 | < 14.71 | 1.00 | < 13.27 | 1.00 |
| No | ≥ 39.46 | 0.66 (-1.22, 2.57) | ≥ 58.15 | 0.65 (-1.27, 2.60) | ≥ 14.71 | 9.30 (6.54, 12.13) | ≥ 13.27 | 1.34 (-0.81, 3.53) |
| Yes | < 39.46 | -7.39 (-14.04, -0.23) | < 58.15 | -9.38 (-15.45, -2.87) | < 14.71 | -0.23 (-7.57, 7.69) | < 13.27 | -11.44 (-20.81, -0.96) |
| Yes | ≥ 39.46 | 4.46 (-0.62, 9.79) | ≥ 58.15 | 7.22 (1.76, 12.97) | ≥ 14.71 | 12.30 (6.37, 18.56) | ≥ 13.27 | 3.93 (-1.10, 9.22) |
| RERI |  | 0.11 (0.03, 0.19) |  | 0.16 (0.08, 0.24) |  | 0.03 (-0.06, 0.12) |  | 0.14 (0.04, 0.25) |
| 2.5^th^ (2d) ^a^ |  |  |  |  |  |  |  |  |
| No | < 39.46 | 1.00 | < 58.15 | 1.00 | < 14.71 | 1.00 | < 13.27 | 1.00 |
| No | ≥ 39.46 | 0.67 (-1.19, 2.58) | ≥ 58.15 | 1.26 (-0.64, 3.20) | ≥ 14.71 | 9.26 (6.49, 12.09) | ≥ 13.27 | 1.94 (-0.20, 4.14) |
| Yes | < 39.46 | 10.26 (3.77, 17.14) | < 58.15 | 9.63 (3.35, 16.29) | < 14.71 | 4.40 (-2.80, 12.13) | < 13.27 | 6.66 (-2.34, 16.48) |
| Yes | ≥ 39.46 | 16.43 (9.73, 23.54) | ≥ 58.15 | 18.99 (11.73, 26.72) | ≥ 14.71 | 31.06 (24.08, 38.43) | ≥ 13.27 | 16.74 (11.07, 22.71) |
| RERI |  | 0.06 (-0.04, 0.15) |  | 0.08 (-0.02, 0.18) |  | 0.17 (0.08, 0.27) |  | 0.08 (-0.02, 0.19) |
| 2.5^th^ (3d) ^a^ |  |  |  |  |  |  |  |  |
| No | < 39.46 | 1.00 | < 58.15 | 1.00 | < 14.71 | 1.00 | < 13.27 | 1.00 |
| No | ≥ 39.46 | 0.71 (-1.13, 2.59) | ≥ 58.15 | 1.11 (-0.76, 3.02) | ≥ 14.71 | 9.53 (6.73, 12.41) | ≥ 13.27 | 1.82 (-0.30, 3.98) |
| Yes | < 39.46 | -3.97 (-11.45, 4.14) | < 58.15 | -3.17 (-10.05, 4.23) | < 14.71 | 3.40 (-4.68, 12.17) | < 13.27 | 10.25 (-6.58, 30.11) |
| Yes | ≥ 39.46 | 17.90 (10.75, 25.53) | ≥ 58.15 | 21.65 (13.74, 30.11) | ≥ 14.71 | 27.62 (19.41, 36.40) | ≥ 13.27 | 10.21 (4.38, 16.37) |
| RERI |  | 0.21 (0.11, 0.32) |  | 0.24 (0.13, 0.34) |  | 0.15 (0.03, 0.26) |  | -0.02 (-0.21, 0.17) |
| 2.5^th^ (4d) ^a^ |  |  |  |  |  |  |  |  |
| No | < 39.46 | 1.00 | < 58.15 | 1.00 | < 14.71 | 1.00 | < 13.27 | 1.00 |
| No | ≥ 39.46 | 0.62 (-1.22, 2.50) | ≥ 58.15 | 1.03 (-0.84, 2.93) | ≥ 14.71 | 8.96 (6.22, 11.78) | ≥ 13.27 | 1.66 (-0.46, 3.82) |
| Yes | < 39.46 | -6.62 (-15.31, 2.95) | < 58.15 | -4.64 (-12.49, 3.91) | < 14.71 | 2.94 (-5.55, 12.19) | < 13.27 | 46.79 (-18.84, 165.49) |
| Yes | ≥ 39.46 | 17.73 (10.53, 25.40) | ≥ 58.15 | 20.85 (12.95, 29.30) | ≥ 14.71 | 28.19 (19.44, 37.57) | ≥ 13.27 | 10.62 (4.66, 16.92) |
| RERI |  | 0.24 (0.12, 0.35) |  | 0.24 (0.13, 0.366) |  | 0.16 (0.04, 0.29) |  | -0.38 (-1.25, 0.49) |
| Zhaoqing |  |  |  |  |  |  |  |  |
| 10^th^ (2d) ^b^ |  |  |  |  |  |  |  |  |
| No | < 44.50 | 1.00 | < 59.50 | 1.00 | < 13.41 | 1.00 | < 14.58 | 1.00 |
| No | ≥ 44.50 | 0.66 (-4.26, 5.83) | ≥ 59.50 | 0.54 (-4.42, 5.75) | ≥ 13.41 | 0.57 (-4.95, 6.42) | ≥ 14.58 | -0.37 (-5.64, 5.20) |
| Yes | < 44.50 | -12.49 (-21.14, -2.88) | < 59.50 | -12.79 (-21.53, -3.09) | < 13.41 | -8.08 (-17.28, 2.15) | < 14.58 | -13.81 (-23.64, -2.71) |
| Yes | ≥ 44.50 | 6.61 (-6.12, 21.06) | ≥ 59.50 | 8.77 (-4.97, 24.49) | ≥ 13.41 | -3.27 (-15.34, 10.52) | ≥ 14.58 | -1.07 (-11.32, 10.37) |
| RERI |  | 0.18 (0.03, 0.34) |  | 0.21 (0.05, 0.37) |  | 0.04 (-0.11, 0.19) |  | 0.13 (-0.01, 0.27) |
| 10^th^ (3d) ^b^ |  |  |  |  |  |  |  |  |
| No | < 44.50 | 1.00 | < 59.50 | 1.00 | < 13.41 | 1.00 | < 14.58 | 1.00 |
| No | ≥ 44.50 | 0.81 (-4.01, 5.87) | ≥ 59.50 | 0.66 (-4.16, 5.73) | ≥ 13.41 | 0.20 (-5.23, 5.95) | ≥ 14.58 | 0.88 (-4.38, 6.43) |
| Yes | < 44.50 | -13.59 (-22.91, -3.15) | < 59.50 | -14.16 (-23.34, -3.88) | < 13.41 | -10.85 (-20.62, 0.12) | < 14.58 | -11.22 (-22.79, 2.08) |
| Yes | ≥ 44.50 | 8.28 (-4.96, 23.36) | ≥ 59.50 | 12.43 (-2.26, 29.34) | ≥ 13.41 | 1.90 (-11.11, 16.81) | ≥ 14.58 | -1.75 (-12.17, 9.91) |
| RERI |  | 0.21 (0.05, 0.37) |  | 0.26 (0.08, 0.44) |  | 0.13 (-0.04, 0.29) |  | 0.09 (-0.07, 0.24) |
| 10^th^ (4d) ^b^ |  |  |  |  |  |  |  |  |
| No | < 44.50 | 1.00 | < 59.50 | 1.00 | < 13.41 | 1.00 | < 14.58 | 1.00 |
| No | ≥ 44.50 | 1.33 (-3.42, 6.30) | ≥ 59.50 | 1.33 (-3.42, 6.32) | ≥ 13.41 | 0.84 (-4.57, 6.56) | ≥ 14.58 | 2.14 (-3.19, 7.77) |
| Yes | < 44.50 | -6.55 (-17.86, 6.31) | < 59.50 | -7.73 (-18.68, 4.70) | < 13.41 | -3.42 (-14.56, 9.17) | < 14.58 | -7.10 (-20.13, 8.06) |
| Yes | ≥ 44.50 | 17.87 (3.18, 34.64) | ≥ 59.50 | 24.00 (7.43, 43.13) | ≥ 13.41 | 17.40 (0.83, 36.70) | ≥ 14.58 | 12.83 (0.15, 27.11) |
| RERI |  | 0.23 (0.04, 0.42) |  | 0.30 (0.10, 0.51) |  | 0.20 (-0.01, 0.40) |  | 0.18 (-0.01, 0.36) |
| 7.5^th^ (2d) ^b^ |  | lag03 |  |  |  |  |  |  |
| No | < 44.50 | 1.00 | < 59.50 | 1.00 | < 13.41 | 1.00 | < 14.58 | 1.00 |
| No | ≥ 44.50 | 1.49 (-3.29, 6.51) | ≥ 59.50 | 1.73 (-3.09, 6.79) | ≥ 13.41 | 0.99 (-4.44, 6.73) | ≥ 14.58 | 0.66 (-4.55, 6.14) |
| Yes | < 44.50 | -11.14 (-20.72, -0.40) | < 59.50 | -10.02 (-19.56, 0.66) | < 13.41 | -6.13 (-16.17, 5.10) | < 14.58 | -10.40 (-21.95, 2.86) |
| Yes | ≥ 44.50 | 13.42 (-1.36, 30.41) | ≥ 59.50 | 15.79 (-0.56, 34.83) | ≥ 13.41 | 2.90 (-11.72, 19.95) | ≥ 14.58 | 2.13 (-9.32, 15.02) |
| RERI |  | 0.23 (0.05, 0.41) |  | 0.24 (0.05, 0.43) |  | 0.08 (-0.10, 0.26) |  | 0.12 (-0.05, 0.28) |
| 7.5^th^ (3d) ^b^ |  |  |  | lag03 |  |  |  |  |
| No | < 44.50 | 1.00 | < 59.50 | 1.00 | < 13.41 | 1.00 | < 14.58 | 1.00 |
| No | ≥ 44.50 | 1.08 (-3.57, 5.95) | ≥ 59.50 | 1.44 (-3.23, 6.33) | ≥ 13.41 | 0.77 (-4.50, 6.34) | ≥ 14.58 | 0.89 (-4.16, 6.21) |
| Yes | < 44.50 | -21.73 (-32.16, -9.70) | < 59.50 | -18.33 (-28.61, -6.58) | < 13.41 | -13.57 (-24.62, -0.91) | < 14.58 | -20.33 (-34.05, -3.75) |
| Yes | ≥ 44.50 | 14.52 (-1.05, 32.54) | ≥ 59.50 | 16.76 (-0.66, 37.23) | ≥ 13.41 | 4.08 (-11.47, 22.37) | ≥ 14.58 | 0.18 (-11.71, 13.67) |
| RERI |  | 0.35 (0.15, 0.55) |  | 0.34 (0.12, 0.55) |  | 0.17 (-0.03, 0.37) |  | 0.20 (0.01, 0.39) |
| 7.5^th^ (4d) ^b^ |  |  |  |  |  |  |  |  |
| No | < 44.50 | 1.00 | < 59.50 | 1.00 | < 13.41 | 1.00 | < 14.58 | 1.00 |
| No | ≥ 44.50 | 1.81 (-2.83, 6.67) | ≥ 59.50 | 1.93 (-2.73, 6.80) | ≥ 13.41 | 1.24 (-4.04, 6.81) | ≥ 14.58 | 2.79 (-2.37, 8.23) |
| Yes | < 44.50 | -13.34 (-26.13, 1.67) | < 59.50 | -12.04 (-24.27, 2.17) | < 13.41 | -6.35 (-18.65, 7.81) | < 14.58 | -15.02 (-30.75, 4.28) |
| Yes | ≥ 44.50 | 14.20 (-2.56, 33.84) | ≥ 59.50 | 18.89 (-0.27, 41.73) | ≥ 13.41 | 10.69 (-9.05, 34.70) | ≥ 14.58 | 7.97 (-6.03, 24.06) |
| RERI |  | 0.26 (0.03, 0.48) |  | 0.29 (0.05, 0.53) |  | 0.16 (-0.09, 0.41) |  | 0.20 (-0.03, 0.43) |
| 5^th^ (2d) ^b^ |  | lag03 |  | lag03 |  |  |  |  |
| No | < 44.50 | 1.00 | < 59.50 | 1.00 | < 13.41 | 1.00 | < 14.58 | 1.00 |
| No | ≥ 44.50 | 1.65 (-3.01, 6.55) | ≥ 59.50 | 1.74 (-2.93, 6.63) | ≥ 13.41 | 1.21 (-4.11, 6.83) | ≥ 14.58 | 1.18 (-3.90, 6.53) |
| Yes | < 44.50 | -20.19 (-31.47, -7.06) | < 59.50 | -19.21 (-30.20, -6.50) | < 13.41 | -9.57 (-21.82, 4.60) | < 14.58 | -15.95 (-31.00, 2.39) |
| Yes | ≥ 44.50 | 15.14 (-1.93, 35.17) | ≥ 59.50 | 20.20 (0.95, 43.11) | ≥ 13.41 | -0.56 (-16.79, 18.83) | ≥ 14.58 | -1.00 (-13.62, 13.47) |
| RERI |  | 0.34 (0.12, 0.55) |  | 0.38 (0.14, 0.61) |  | 0.08 (-0.14, 0.30) |  | 0.14 (-0.07, 0.35) |
| 5^th^ (3d) ^b^ |  |  |  |  |  |  |  |  |
| No | < 44.50 | 1.00 | < 59.50 | 1.00 | < 13.41 | 1.00 | < 14.58 | 1.00 |
| No | ≥ 44.50 | 2.18 (-2.44, 7.03) | ≥ 59.50 | 2.18 (-2.44, 7.03) | ≥ 13.41 | 1.45 (-3.82, 7.00) | ≥ 14.58 | 1.47 (-3.54, 6.75) |
| Yes | < 44.50 | -23.20 (-36.02, -7.82) | < 59.50 | -21.88 (-34.12, -7.36) | < 13.41 | -11.01 (-23.96, 4.14) | < 14.58 | -21.83 (-39.06, 0.28) |
| Yes | ≥ 44.50 | 11.98 (-5.76, 33.06) | ≥ 59.50 | 17.40 (-2.64, 41.57) | ≥ 13.41 | 0.12 (-18.90, 23.59) | ≥ 14.58 | -1.20 (-14.77, 14.54) |
| RERI |  | 0.33 (0.09, 0.57) |  | 0.37 (0.12, 0.63) |  | 0.10 (-0.15, 0.35) |  | 0.19 (-0.05, 0.44) |
| 5^th^ (4d) ^b^ |  |  |  |  |  |  |  | lag03 (14.58) |
| No | < 44.50 | 1.00 | < 59.50 | 1.00 | < 13.41 | 1.00 | < 14.58 | 1.00 |
| No | ≥ 44.50 | 2.49 (-2.14, 7.33) | ≥ 59.50 | 2.30 (-2.32, 7.15) | ≥ 13.41 | 0.93 (-4.30, 6.45) | ≥ 14.58 | 3.14 (-1.98, 8.53) |
| Yes | < 44.50 | -13.67 (-28.67, 4.49) | < 59.50 | -16.36 (-30.26, 0.32) | < 13.41 | -13.09 (-26.33, 2.53) | < 14.58 | -21.13 (-39.04, 2.04) |
| Yes | ≥ 44.50 | 4.06 (-14.08, 26.02) | ≥ 59.50 | 11.39 (-9.13, 36.54) | ≥ 13.41 | 12.02 (-12.01, 42.61) | ≥ 14.58 | 3.01 (-12.38, 21.09) |
| RERI |  | 0.15 (-0.11, 0.41) |  | 0.25 (-0.02, 0.53) |  | 0.24 (-0.06, 0.54) |  | 0.21 (-0.05, 0.47) |

Note: PM, particulate matter; SO_2_, sulfur dioxide; NO_2_, nitrogen dioxide.

^a^ Cold spells were defined by percentile temperature thresholds (10^th^, 7.5^th^, 5^th^ and 2.5^th^) and by the number of consecutive days below the thresholds (2-4 d).

^b^ Nine definitions [10^th^ (2d), 10^th^ (3d), 10^th^ (4d), 7.5^th^ (2d), 7.5^th^ (3d), 7.5^th^ (4d), 5^th^ (2d), 5^th^ (3d), and 5^th^ (4d)] were used in Zhaoqing.

^c^ Air pollutants were classified as binary variables using the median of air pollutant (lag03) concentrations as a cut off.

## Table S6. Independent effects of cold spells on anxiety stratified by age when PM_2.5_ and PM_10_ at lag03 were included in each model separately.

| Cold spell | PM_2.5_ ^c^ | | |  | PM_10_ ^c^ | | |
| --- | --- | --- | --- | --- | --- | --- | --- |
|  | < 18 | 18-65 | ≥ 65 |  | < 18 | 18-65 | ≥ 65 |
| Huizhou |  |  |  |  |  |  |  |
| 10^th^ (2d) ^a^ | 5.93 (-20.27, 40.75) | 11.03 (5.13, 17.26) | 12.96 (-1.94, 30.11) |  | 6.78 (-19.86, 42.29) | 12.53 (6.48, 18.91) | 14.34 (-0.91, 31.93) |
| 10^th^ (3d) ^a^ | 3.74 (-24.31, 42.18) | 9.87 (3.69, 16.41) | 10.35 (-5.10, 28.32) |  | 4.98 (-23.56, 44.18) | 11.16 (4.86, 17.84) | 11.49 (-4.23, 29.81) |
| 10^th^ (4d) ^a^ | 7.01 (-24.40, 51.48) | 8.56 (1.90, 15.64) | 13.61 (-3.61, 33.89) |  | 8.39 (-23.50, 53.58) | 9.55 (2.81, 16.74) | 14.45 (-2.93, 34.95) |
| 7.5^th^ (2d) ^a^ | 5.33 (-23.54, 45.09) | 11.79 (5.28, 18.69) | 9.89 (-5.92, 28.37) |  | 6.52 (-22.86, 47.08) | 13.27 (6.62, 20.33) | 11.09 (-5.02, 29.94) |
| 7.5^th^ (3d) ^a^ | 10.34 (-21.61, 55.33) | 10.99 (4.25, 18.16) | 13.26 (-3.70, 33.22) |  | 11.80 (-20.70, 57.61) | 12.13 (5.29, 19.41) | 14.24 (-2.94, 34.46) |
| 7.5^th^ (4d) ^a^ | 4.64 (-29.63, 55.60) | 8.85 (1.33, 16.94) | 11.33 (-7.79, 34.41) |  | 5.79 (-28.92, 57.44) | 9.86 (2.24, 18.04) | 12.16 (-7.15, 35.48) |
| 5^th^ (2d) ^a^ | 5.13 (-26.14, 49.64) | 10.11 (3.10, 17.60) | 12.90 (-4.83, 33.94) |  | 5.85 (-25.83, 51.04) | 11.58 (4.41, 19.25) | 14.19 (-3.89, 35.67) |
| 5^th^ (3d) ^a^ | 2.52 (-31.19, 52.75) | 13.35 (5.38, 21.92) | 22.64 (1.78, 47.78) |  | 2.76 (-31.13, 53.31) | 14.53 (6.43, 23.23) | 23.75 (2.61, 49.25) |
| 5^th^ (4d) ^a^ | -9.40 (-42.58, 42.97) | 15.43 (6.27, 25.38) | 15.39 (-6.86, 42.96) |  | -9.30 (-42.59, 43.29) | 16.62 (7.33, 26.72) | 16.32 (-6.18, 44.21) |
| 2.5^th^ (2d) ^a^ | 25.96 (-20.07, 98.50) | 15.57 (6.38, 25.56) | 9.80 (-11.87, 36.80) |  | 26.37 (-19.90, 99.39) | 16.72 (7.40, 26.85) | 10.58 (-11.30, 37.85) |
| 2.5^th^ (3d) ^a^ | 29.65 (-22.53, 116.96) | 16.29 (5.92, 27.67) | 0.14 (-21.97, 28.53) |  | 29.11 (-22.92, 116.28) | 17.15 (6.68, 28.64) | 0.66 (-21.60, 29.25) |
| 2.5^th^ (4d) ^a^ | 22.37 (-30.66, 115.98) | 17.30 (5.74, 30.13) | -7.16 (-29.97, 23.08) |  | 21.66 (-31.13, 114.89) | 18.15 (6.48, 31.10) | -6.69 (-29.64, 23.76) |
| Shenzhen |  |  |  |  |  |  |  |
| 10^th^ (2d) ^a^ | -2.77 (-17.16, 14.13) | 7.29 (4.03, 10.65) | 10.77 (-0.42, 23.22) |  | -3.30 (-17.69, 13.61) | 7.94 (4.64, 11.35) | 11.96 (0.57, 24.65) |
| 10^th^ (3d) ^a^ | -2.52 (-17.94, 15.79) | 6.89 (3.36, 10.55) | 9.11 (-2.82, 22.50) |  | -3.32 (-18.66, 14.93) | 7.50 (3.94, 11.19) | 10.29 (-1.81, 23.88) |
| 10^th^ (4d) ^a^ | 1.18 (-14.91, 20.31) | 5.12 (1.65, 8.71) | 6.88 (-4.68, 19.85) |  | 0.38 (-15.62, 19.41) | 5.56 (2.07, 9.18) | 7.76 (-3.92, 20.87) |
| 7.5^th^ (2d) ^a^ | -3.13 (-18.32, 14.90) | 5.05 (1.65, 8.56) | 4.46 (-6.71, 16.96) |  | -3.70 (-18.89, 14.33) | 5.61 (2.17, 9.16) | 5.40 (-5.94, 18.10) |
| 7.5^th^ (3d) ^a^ | -0.16 (-16.65, 19.58) | 5.07 (1.45, 8.82) | 9.19 (-3.08, 23.01) |  | -0.93 (-17.38, 18.79) | 5.53 (1.88, 9.31) | 10.10 (-2.32, 24.10) |
| 7.5^th^ (4d) ^a^ | 7.29 (-12.13, 30.99) | 3.54 (-0.33, 7.56) | 4.07 (-8.64, 18.56) |  | 6.56 (-12.77, 30.17) | 3.86 (-0.02, 7.90) | 4.74 (-8.08, 19.35) |
| 5^th^ (2d) ^a^ | -1.48 (-18.49, 19.08) | 2.81 (-0.86, 6.61) | -3.67 (-14.93, 9.09) |  | -2.20 (-19.20, 18.38) | 3.38 (-0.34, 7.23) | -2.85 (-14.28, 10.11) |
| 5^th^ (3d) ^a^ | -6.12 (-24.04, 16.02) | -1.80 (-5.73, 2.29) | -5.75 (-17.93, 8.23) |  | -6.97 (-24.81, 15.11) | -1.44 (-5.40, 2.68) | -5.09 (-17.41, 9.06) |
| 5^th^ (4d) ^a^ | -1.43 (-23.56, 27.10) | 1.30 (-3.35, 6.18) | -2.06 (-16.51, 14.91) |  | -2.17 (-24.17, 26.22) | 1.65 (-3.03, 6.55) | -1.40 (-15.97, 15.71) |
| 2.5^th^ (2d) ^a^ | 1.51 (-20.83, 30.15) | 14.09 (9.01, 19.41) | 6.39 (-9.36, 24.86) |  | 1.10 (-21.17, 29.67) | 14.59 (9.47, 19.94) | 7.13 (-8.75, 25.77) |
| 2.5^th^ (3d) ^a^ | -10.15 (-33.34, 21.10) | 10.53 (4.88, 16.48) | -2.46 (-18.62, 16.91) |  | -10.55 (-33.64, 20.56) | 10.79 (5.12, 16.76) | -2.07 (-18.31, 17.41) |
| 2.5^th^ (4d) ^a^ | -9.98 (-35.22, 25.09) | 11.29 (5.26, 17.66) | -1.43 (-18.43, 19.13) |  | -10.41 (-35.53, 24.49) | 11.52 (5.48, 17.92) | -1.06 (-18.15, 19.60) |
| Zhaoqing |  |  |  |  |  |  |  |
| 10^th^ (2d) ^b^ | -15.55 (-54.60, 57.09) | -5.10 (-13.64, 4.29) | -6.81 (-25.72, 16.90) |  | -13.47 (-53.54, 61.17) | -5.74 (-14.28, 3.65) | -7.51 (-26.38, 16.18) |
| 10^th^ (3d) ^b^ | -18.26 (-57.43, 56.95) | -5.20 (-14.15, 4.68) | -1.52 (-22.20, 24.65) |  | -16.47 (-56.59, 60.71) | -5.71 (-14.66, 4.18) | -2.05 (-22.73, 24.15) |
| 10^th^ (4d) ^b^ | -1.17 (-50.77, 98.41) | 3.03 (-7.35, 14.57) | 13.63 (-11.43, 45.76) |  | 0.51 (-49.97, 101.94) | 2.65 (-7.74, 14.21) | 13.28 (-11.80, 45.51) |
| 7.5^th^ (2d) ^b^ | -20.01 (-59.20, 56.79) | -1.78 (-11.27, 8.73) | -4.61 (-24.86, 21.10) |  | -18.28 (-58.37, 60.41) | -2.24 (-11.75, 8.29) | -5.05 (-25.31, 20.69) |
| 7.5^th^ (3d) ^b^ | -9.58 (-56.54, 88.12) | -5.82 (-15.98, 5.56) | -10.47 (-31.76, 17.45) |  | -8.12 (-55.91, 91.46) | -6.09 (-16.25, 5.29) | -10.65 (-31.93, 17.28) |
| 7.5^th^ (4d) ^b^ | -12.44 (-61.43, 98.77) | 0.08 (-11.69, 13.41) | -6.69 (-30.46, 25.20) |  | -11.58 (-61.08, 100.87) | -0.22 (-11.97, 13.09) | -6.87 (-30.62, 25.01) |
| 5^th^ (2d) ^b^ | -26.12 (-66.10, 61.01) | -6.72 (-17.63, 5.64) | 1.63 (-23.13, 34.37) |  | -24.85 (-65.54, 63.87) | -7.08 (-17.99, 5.28) | 1.33 (-23.43, 34.11) |
| 5^th^ (3d) ^b^ | -23.92 (-68.81, 85.61) | -8.31 (-20.22, 5.37) | -0.65 (-26.93, 35.10) |  | -22.90 (-68.41, 88.21) | -8.53 (-20.43, 5.14) | -0.77 (-27.06, 34.99) |
| 5^th^ (4d) ^b^ | -26.89 (-71.85, 89.87) | -6.85 (-19.87, 8.30) | 0.91 (-27.34, 40.14) |  | -25.98 (-71.50, 92.27) | -7.16 (-20.15, 7.95) | 0.73 (-27.49, 39.94) |

Note: PM, particulate matter.

^a^ Cold spells were defined by percentile temperature thresholds (10^th^, 7.5^th^, 5^th^ and 2.5^th^) and by the number of consecutive days below the thresholds (2-4 d).

^b^ Nine definitions [10^th^ (2d), 10^th^ (3d), 10^th^ (4d), 7.5^th^ (2d), 7.5^th^ (3d), 7.5^th^ (4d), 5^th^ (2d), 5^th^ (3d), and 5^th^ (4d)] were used in Zhaoqing.

^c^ Air pollutants at lag03 were included in each model separately.

## Table S7. Independent effects of cold spells on anxiety stratified by age when NO_2_ and SO_2_ at lag03 were included in each model separately.

| Cold spell | NO_2_ ^c^ | | |  | SO_2_ ^c^ | | |
| --- | --- | --- | --- | --- | --- | --- | --- |
|  | < 18 | 18-65 | ≥ 65 |  | < 18 | 18-65 | ≥ 65 |
| Huizhou |  |  |  |  |  |  |  |
| 10^th^ (2d) ^a^ | 20.19 (-9.67, 59.92) | 14.00 (7.92, 20.42) | 17.21 (1.63, 35.18) |  | 17.85 (-10.85, 55.80) | 11.72 (5.90, 17.86) | 14.66 (-0.27, 31.83) |
| 10^th^ (3d) ^a^ | 21.11 (-12.03, 66.73) | 13.13 (6.73, 19.92) | 14.98 (-1.31, 33.95) |  | 16.21 (-14.48, 57.91) | 9.95 (3.91, 16.34) | 11.39 (-3.93, 29.16) |
| 10^th^ (4d) ^a^ | 24.41 (-12.66, 77.20) | 11.76 (4.85, 19.13) | 17.99 (-0.07, 39.31) |  | 18.60 (-15.61, 66.68) | 8.26 (1.74, 15.19) | 13.84 (-3.17, 33.84) |
| 7.5^th^ (2d) ^a^ | 23.58 (-10.84, 71.31) | 16.39 (9.49, 23.73) | 15.69 (-1.32, 35.62) |  | 18.72 (-13.26, 62.48) | 12.19 (5.79, 18.96) | 11.26 (-4.50, 29.61) |
| 7.5^th^ (3d) ^a^ | 30.42 (-8.12, 85.11) | 15.33 (8.20, 22.92) | 19.08 (0.88, 40.57) |  | 23.28 (-11.78, 72.28) | 11.12 (4.49, 18.18) | 14.28 (-2.64, 34.14) |
| 7.5^th^ (4d) ^a^ | 21.56 (-18.98, 82.40) | 13.32 (5.34, 21.90) | 17.05 (-3.44, 41.90) |  | 15.25 (-22.08, 70.48) | 8.66 (1.25, 16.62) | 11.78 (-7.26, 34.73) |
| 5^th^ (2d) ^a^ | 23.76 (-14.22, 78.54) | 15.37 (7.81, 23.45) | 19.81 (0.43, 42.93) |  | 16.77 (-17.55, 65.37) | 10.21 (3.29, 17.59) | 13.83 (-3.87, 34.80) |
| 5^th^ (3d) ^a^ | 16.49 (-22.75, 75.68) | 18.40 (9.89, 27.56) | 29.54 (7.02, 56.80) |  | 10.84 (-25.48, 64.87) | 13.34 (5.42, 21.84) | 23.40 (2.50, 48.57) |
| 5^th^ (4d) ^a^ | 2.73 (-35.72, 64.20) | 21.39 (11.54, 32.12) | 22.48 (-1.68, 52.56) |  | -2.07 (-37.85, 54.32) | 15.16 (6.07, 25.03) | 16.01 (-6.30, 43.62) |
| 2.5^th^ (2d) ^a^ | 42.10 (-10.36, 125.26) | 20.14 (10.46, 30.67) | 14.51 (-8.34, 43.05) |  | 35.40 (-13.82, 112.74) | 15.05 (5.96, 24.93) | 9.84 (-11.75, 36.72) |
| 2.5^th^ (3d) ^a^ | 43.61 (-15.20, 143.21) | 21.48 (10.49, 33.58) | 4.60 (-18.78, 34.72) |  | 36.15 (-18.81, 128.32) | 16.20 (5.84, 27.56) | 0.31 (-21.83, 28.72) |
| 2.5^th^ (4d) ^a^ | 35.57 (-24.20, 142.49) | 23.05 (10.74, 36.73) | -2.52 (-26.78, 29.78) |  | 28.36 (-27.48, 127.20) | 17.36 (5.79, 30.19) | -6.79 (-29.69, 23.57) |
| Shenzhen |  |  |  |  |  |  |  |
| 10^th^ (2d) ^a^ | -1.19 (-15.63, 15.71) | 10.45 (7.14, 13.87) | 14.39 (2.95, 27.10) |  | -0.04 (-14.51, 16.87) | 7.67 (4.50, 10.93) | 11.22 (0.31, 23.32) |
| 10^th^ (3d) ^a^ | -2.16 (-17.40, 15.87) | 10.28 (6.70, 13.98) | 13.00 (0.86, 26.61) |  | 0.39 (-14.83, 18.33) | 6.74 (3.40, 10.19) | 8.77 (-2.54, 21.39) |
| 10^th^ (4d) ^a^ | 1.25 (-14.71, 20.20) | 8.27 (4.72, 11.94) | 10.48 (-1.40, 23.78) |  | 3.47 (-12.44, 22.28) | 5.24 (1.89, 8.70) | 6.80 (-4.35, 19.25) |
| 7.5^th^ (2d) ^a^ | -2.26 (-17.63, 15.97) | 9.20 (5.63, 12.89) | 5.67 (-5.47, 18.13) |  | -0.63 (-15.96, 17.51) | 5.76 (2.41, 9.22) | 9.83 (-2.14, 23.28) |
| 7.5^th^ (3d) ^a^ | -1.63 (-18.22, 18.32) | 9.55 (5.68, 13.57) | 15.76 (2.33, 30.96) |  | 1.87 (-14.62, 21.55) | 5.14 (1.61, 8.79) | 9.13 (-2.87, 22.62) |
| 7.5^th^ (4d) ^a^ | 6.14 (-13.33, 29.99) | 7.34 (3.25, 11.60) | 9.22 (-4.42, 24.80) |  | 9.53 (-9.90, 33.15) | 3.48 (-0.31, 7.42) | 3.60 (-8.82, 17.70) |
| 5^th^ (2d) ^a^ | -1.21 (-18.56, 19.83) | 8.08 (4.12, 12.20) | 2.25 (-10.10, 16.29) |  | 1.49 (-15.65, 22.10) | 3.31 (-0.28, 7.03) | -2.97 (-14.07, 9.57) |
| 5^th^ (3d) ^a^ | -8.08 (-26.00, 14.17) | 2.25 (-1.95, 6.63) | -0.76 (-13.95, 14.45) |  | -3.81 (-21.85, 18.40) | -1.44 (-5.32, 2.60) | -5.48 (-17.52, 8.33) |
| 5^th^ (4d) ^a^ | -3.91 (-25.93, 24.66) | 6.00 (1.00, 11.24) | 3.76 (-11.95, 22.27) |  | 1.82 (-20.65, 30.65) | 1.24 (-3.34, 6.03) | -2.81 (-16.93, 13.71) |
| 2.5^th^ (2d) ^a^ | 2.83 (-19.72, 31.72) | 17.99 (12.70, 23.51) | 11.23 (-5.38, 30.76) |  | 5.11 (-17.70, 34.23) | 13.69 (8.69, 18.91) | 5.14 (-10.24, 23.17) |
| 2.5^th^ (3d) ^a^ | -11.83 (-34.74, 19.12) | 14.71 (8.75, 21.00) | 2.19 (-15.03, 22.89) |  | -8.01 (-31.68, 23.86) | 10.22 (4.62, 16.13) | -3.49 (-19.44, 15.62) |
| 2.5^th^ (4d) ^a^ | -12.52 (-37.25, 21.97) | 15.96 (9.55, 22.74) | 3.71 (-14.53, 25.84) |  | -7.36 (-33.30, 28.68) | 10.75 (4.77, 17.08) | -2.86 (-19.60, 17.36) |
| Zhaoqing |  |  |  |  |  |  |  |
| 10^th^ (2d) ^b^ | -20.94 (-56.83, 44.77) | -5.94 (-14.39, 3.34) | -5.96 (-24.91, 17.77) |  | -20.80 (-56.06, 42.75) | -4.28 (-12.67, 4.93) | -7.37 (-25.70, 15.49) |
| 10^th^ (3d) ^b^ | -22.04 (-59.33, 49.45) | -5.91 (-14.85, 3.97) | -0.52 (-21.44, 25.96) |  | -21.82 (-58.74, 48.15) | -4.50 (-13.41, 5.31) | -2.11 (-22.44, 23.53) |
| 10^th^ (4d) ^b^ | -4.33 (-52.44, 92.43) | 2.67 (-7.77, 14.30) | 15.35 (-10.22, 48.20) |  | -5.97 (-52.71, 86.96) | 3.44 (-6.92, 14.94) | 12.62 (-12.00, 44.13) |
| 7.5^th^ (2d) ^b^ | -24.10 (-61.08, 48.04) | -2.48 (-11.96, 8.03) | -3.56 (-24.13, 22.58) |  | -23.78 (-60.47, 46.97) | -1.12 (-10.57, 9.33) | -5.13 (-25.06, 20.10) |
| 7.5^th^ (3d) ^b^ | -11.24 (-57.43, 85.03) | -6.23 (-16.42, 5.19) | -9.52 (-31.15, 18.91) |  | -12.01 (-57.62, 82.65) | -5.46 (-15.63, 5.94) | -10.84 (-31.98, 16.88) |
| 7.5^th^ (4d) ^b^ | -12.82 (-61.76, 98.73) | -0.20 (-12.03, 13.21) | -5.28 (-29.58, 27.41) |  | -15.64 (-62.66, 90.60) | 0.34 (-11.42, 13.67) | -7.23 (-30.80, 24.38) |
| 5^th^ (2d) ^b^ | -29.32 (-67.46, 53.55) | -7.26 (-18.15, 5.08) | 2.60 (-22.47, 35.78) |  | -29.02 (-67.17, 53.43) | -6.27 (-17.18, 6.07) | 0.79 (-23.61, 32.99) |
| 5^th^ (3d) ^b^ | -25.01 (-69.28, 83.06) | -8.66 (-20.60, 5.06) | 0.76 (-26.05, 37.28) |  | -27.41 (-70.08, 76.13) | -8.31 (-20.19, 5.34) | -1.57 (-27.54, 33.72) |
| 5^th^ (4d) ^b^ | -27.51 (-72.18, 88.85) | -7.22 (-20.29, 7.99) | 2.81 (-26.18, 43.17) |  | -30.90 (-73.22, 78.31) | -6.81 (-19.80, 8.29) | -0.12 (-28.00, 38.57) |

Note: SO_2_, sulfur dioxide; NO_2_, nitrogen dioxide.

^a^ Cold spells were defined by percentile temperature thresholds (10^th^, 7.5^th^, 5^th^ and 2.5^th^) and by the number of consecutive days below the thresholds (2-4 d).

^b^ Nine definitions [10^th^ (2d), 10^th^ (3d), 10^th^ (4d), 7.5^th^ (2d), 7.5^th^ (3d), 7.5^th^ (4d), 5^th^ (2d), 5^th^ (3d), and 5^th^ (4d)] were used in Zhaoqing.

^c^ Air pollutants at lag03 were included in each model separately.

## Table S8. Independent effects of PM_2.5_ and PM_10_ on anxiety stratified by age when cold spells under different definitions were controlled.

| Cold spell | PM_2.5_ ^c^ | | |  | PM_10_ ^c^ | | |
| --- | --- | --- | --- | --- | --- | --- | --- |
|  | < 18 | 18-65 | ≥ 65 |  | < 18 | 18-65 | ≥ 65 |
| Huizhou |  |  |  |  |  |  |  |
| 10^th^ (2d) ^a^ | -4.40 (-9.21, 0.67) | 1.74 (0.75, 2.73) | 1.40 (-1.15, 4.02) |  | -2.56 (-6.26, 1.29) | 1.74 (0.99, 2.50) | 1.48 (-0.47, 3.46) |
| 10^th^ (3d) ^a^ | -4.50 (-9.32, 0.58) | 1.64 (0.66, 2.63) | 1.26 (-1.29, 3.87) |  | -2.64 (-6.34, 1.20) | 1.64 (0.89, 2.39) | 1.33 (-0.61, 3.30) |
| 10^th^ (4d) ^a^ | -4.44 (-9.21, 0.58) | 1.49 (0.52, 2.47) | 1.23 (-1.28, 3.81) |  | -2.61 (-6.26, 1.19) | 1.49 (0.76, 2.23) | 1.27 (-0.63, 3.21) |
| 7.5^th^ (2d) ^a^ | -4.44 (-9.26, 0.63) | 1.72 (0.73, 2.71) | 1.24 (-1.31, 3.85) |  | -2.60 (-6.30, 1.25) | 1.71 (0.96, 2.46) | 1.31 (-0.63, 3.29) |
| 7.5^th^ (3d) ^a^ | -4.31 (-9.11, 0.74) | 1.60 (0.62, 2.58) | 1.26 (-1.26, 3.85) |  | -2.49 (-6.17, 1.32) | 1.58 (0.85, 2.33) | 1.31 (-0.60, 3.25) |
| 7.5^th^ (4d) ^a^ | -4.55 (-9.28, 0.41) | 1.44 (0.47, 2.41) | 1.08 (-1.42, 3.64) |  | -2.71 (-6.32, 1.04) | 1.44 (0.72, 2.18) | 1.15 (-0.74, 3.07) |
| 5^th^ (2d) ^a^ | -4.51 (-9.26, 0.49) | 1.52 (0.55, 2.50) | 1.18 (-1.32, 3.75) |  | -2.67 (-6.32, 1.12) | 1.55 (0.81, 2.29) | 1.28 (-0.63, 3.22) |
| 5^th^ (3d) ^a^ | -4.62 (-9.30, 0.31) | 1.46 (0.50, 2.43) | 1.22 (-1.27, 3.77) |  | -2.77 (-6.35, 0.95) | 1.48 (0.76, 2.21) | 1.30 (-0.59, 3.22) |
| 5^th^ (4d) ^a^ | -4.78 (-9.44, 0.12) | 1.43 (0.48, 2.40) | 1.03 (-1.45, 3.57) |  | -2.92 (-6.49, 0.77) | 1.46 (0.73, 2.18) | 1.12 (-0.75, 3.03) |
| 2.5^th^ (2d) ^a^ | -4.37 (-9.06, 0.56) | 1.42 (0.47, 2.38) | 0.94 (-1.53, 3.47) |  | -2.55 (-6.13, 1.17) | 1.45 (0.72, 2.17) | 1.04 (-0.82, 2.95) |
| 2.5^th^ (3d) ^a^ | -4.53 (-9.19, 0.38) | 1.31 (0.36, 2.26) | 0.83 (-1.62, 3.35) |  | -2.67 (-6.23, 1.03) | 1.35 (0.63, 2.07) | 0.94 (-0.92, 2.83) |
| 2.5^th^ (4d) ^a^ | -4.59 (-9.24, 0.31) | 1.30 (0.35, 2.25) | 0.79 (-1.66, 3.31) |  | -2.73 (-6.28, 0.96) | 1.33 (0.62, 2.05) | 0.90 (-0.96, 2.79) |
| Shenzhen |  |  |  |  |  |  |  |
| 10^th^ (2d) ^a^ | -2.31 (-4.93, 0.38) | 0.42 (-0.11, 0.95) | 1.18 (-0.60, 3.00) |  | -2.04 (-4.23, 0.19) | 0.57 (0.14, 1.01) | 1.34 (-0.13, 2.83) |
| 10^th^ (3d) ^a^ | -2.03 (-4.72, 0.75) | 0.57 (0.03, 1.12) | 1.28 (-0.55, 3.14) |  | -1.92 (-4.16, 0.37) | 0.65 (0.21, 1.10) | 1.38 (-0.12, 2.90) |
| 10^th^ (4d) ^a^ | -1.90 (-4.56, 0.85) | 0.41 (-0.12, 0.94) | 1.09 (-0.69, 2.91) |  | -1.84 (-4.05, 0.42) | 0.50 (0.06, 0.93) | 1.19 (-0.27, 2.68) |
| 7.5^th^ (2d) ^a^ | -2.30 (-4.90, 0.37) | 0.25 (-0.27, 0.77) | 0.81 (-0.93, 2.59) |  | -2.04 (-4.20, 0.18) | 0.42 (-0.00, 0.85) | 1.01 (-0.43, 2.47) |
| 7.5^th^ (3d) ^a^ | -1.89 (-4.48, 0.78) | 0.35 (-0.17, 0.88) | 1.08 (-0.66, 2.86) |  | -1.78 (-3.94, 0.43) | 0.47 (0.04, 0.89) | 1.20 (-0.23, 2.66) |
| 7.5^th^ (4d) ^a^ | -1.76 (-4.35, 0.91) | 0.26 (-0.26, 0.77) | 0.87 (-0.86, 2.63) |  | -1.70 (-3.85, 0.49) | 0.36 (-0.06, 0.78) | 0.99 (-0.42, 2.43) |
| 5^th^ (2d) ^a^ | -2.22 (-4.81, 0.44) | 0.14 (-0.38, 0.66) | 0.50 (-1.23, 2.26) |  | -1.97 (-4.13, 0.24) | 0.33 (-0.10, 0.76) | 0.72 (-0.71, 2.17) |
| 5^th^ (3d) ^a^ | -2.04 (-4.61, 0.60) | 0.12 (-0.39, 0.64) | 0.61 (-1.10, 2.35) |  | -1.92 (-4.06, 0.26) | 0.26 (-0.16, 0.68) | 0.78 (-0.63, 2.20) |
| 5^th^ (4d) ^a^ | -1.98 (-4.55, 0.65) | 0.19 (-0.32, 0.70) | 0.71 (-1.00, 2.46) |  | -1.89 (-4.02, 0.28) | 0.30 (-0.12, 0.72) | 0.86 (-0.55, 2.28) |
| 2.5^th^ (2d) ^a^ | -2.14 (-4.68, 0.47) | 0.28 (-0.23, 0.79) | 0.74 (-0.97, 2.47) |  | -1.88 (-3.99, 0.28) | 0.43 (0.02, 0.85) | 0.92 (-0.48, 2.33) |
| 2.5^th^ (3d) ^a^ | -1.97 (-4.49, 0.63) | 0.26 (-0.25, 0.76) | 0.74 (-0.95, 2.45) |  | -1.83 (-3.92, 0.30) | 0.37 (-0.04, 0.78) | 0.88 (-0.50, 2.28) |
| 2.5^th^ (4d) ^a^ | -2.02 (-4.54, 0.57) | 0.24 (-0.26, 0.75) | 0.75 (-0.94, 2.47) |  | -1.92 (-4.01, 0.22) | 0.35 (-0.06, 0.76) | 0.87 (-0.51, 2.28) |
| Zhaoqing |  |  |  |  |  |  |  |
| 10^th^ (2d) ^b^ | 5.01 (-4.05, 14.92) | 0.38 (-1.18, 1.98) | 1.03 (-2.40, 4.58) |  | 5.03 (-2.77, 13.46) | -0.01 (-1.33, 1.34) | 0.52 (-2.39, 3.51) |
| 10^th^ (3d) ^b^ | 5.15 (-3.63, 14.73) | 0.44 (-1.10, 2.00) | 1.27 (-2.10, 4.76) |  | 5.10 (-2.47, 13.25) | 0.04 (-1.26, 1.36) | 0.74 (-2.13, 3.68) |
| 10^th^ (4d) ^b^ | 5.78 (-2.95, 15.30) | 0.55 (-0.98, 2.09) | 1.59 (-1.77, 5.07) |  | 5.62 (-1.88, 13.70) | 0.15 (-1.13, 1.46) | 1.05 (-1.79, 3.98) |
| 7.5^th^ (2d) ^b^ | 5.05 (-3.76, 14.66) | 0.58 (-0.96, 2.15) | 1.21 (-2.14, 4.68) |  | 5.02 (-2.57, 13.19) | 0.19 (-1.12, 1.51) | 0.69 (-2.15, 3.61) |
| 7.5^th^ (3d) ^b^ | 5.78 (-2.81, 15.13) | 0.53 (-0.98, 2.06) | 1.19 (-2.10, 4.58) |  | 5.60 (-1.78, 13.54) | 0.14 (-1.14, 1.43) | 0.66 (-2.12, 3.52) |
| 7.5^th^ (4d) ^b^ | 5.62 (-2.92, 14.92) | 0.48 (-1.03, 2.01) | 1.18 (-2.10, 4.58) |  | 5.45 (-1.88, 13.33) | 0.09 (-1.18, 1.38) | 0.67 (-2.10, 3.53) |
| 5^th^ (2d) ^b^ | 5.19 (-3.44, 14.59) | 0.52 (-1.00, 2.06) | 1.39 (-1.93, 4.82) |  | 5.10 (-2.31, 13.08) | 0.13 (-1.15, 1.43) | 0.86 (-1.94, 3.75) |
| 5^th^ (3d) ^b^ | 5.62 (-2.90, 14.88) | 0.54 (-0.96, 2.07) | 1.32 (-1.97, 4.72) |  | 5.44 (-1.87, 13.30) | 0.15 (-1.12, 1.44) | 0.80 (-1.98, 3.65) |
| 5^th^ (4d) ^b^ | 5.41 (-3.10, 14.67) | 0.41 (-1.10, 1.94) | 1.26 (-2.03, 4.66) |  | 5.27 (-2.04, 13.12) | 0.03 (-1.24, 1.32) | 0.75 (-2.03, 3.61) |

Note: PM, particulate matter.

^a^ Cold spells were defined by percentile temperature thresholds (10^th^, 7.5^th^, 5^th^ and 2.5^th^) and by the number of consecutive days below the thresholds (2-4 d).

^b^ Nine definitions [10^th^ (2d), 10^th^ (3d), 10^th^ (4d), 7.5^th^ (2d), 7.5^th^ (3d), 7.5^th^ (4d), 5^th^ (2d), 5^th^ (3d), and 5^th^ (4d)] were used in Zhaoqing.

^c^ Air pollutants at lag03 were included in each model separately.

## Table S9. Independent effects of NO_2_ and SO_2_ on anxiety stratified by age when cold spells under different definitions were controlled.

| Cold spell | NO_2_ ^c^ | | |  | SO_2_ ^c^ | | |
| --- | --- | --- | --- | --- | --- | --- | --- |
|  | < 18 | 18-65 | ≥ 65 |  | < 18 | 18-65 | ≥ 65 |
| Huizhou |  |  |  |  |  |  |  |
| Huizhou | 16.88 (-5.00, 43.80) | 13.63 (9.34, 18.08) | 15.05 (4.04, 27.22) |  | 16.84 (-4.18, 42.48) | 11.65 (7.75, 15.69) | 11.88 (2.19, 22.47) |
| 10^th^ (2d) ^a^ | 17.17 (-5.03, 44.55) | 13.27 (9.00, 17.72) | 14.47 (3.49, 26.61) |  | 16.02 (-4.72, 41.29) | 11.06 (7.21, 15.05) | 11.07 (1.53, 21.50) |
| 10^th^ (3d) ^a^ | 16.87 (-5.09, 43.93) | 12.51 (8.31, 16.88) | 14.15 (3.35, 26.08) |  | 15.65 (-4.94, 40.70) | 10.55 (6.75, 14.50) | 10.67 (1.24, 20.98) |
| 10^th^ (4d) ^a^ | 17.70 (-4.65, 45.29) | 14.22 (9.85, 18.76) | 14.87 (3.74, 27.18) |  | 16.50 (-4.40, 41.97) | 11.41 (7.54, 15.42) | 11.11 (1.56, 21.57) |
| 7.5^th^ (2d) ^a^ | 18.58 (-3.96, 46.41) | 13.49 (9.20, 17.94) | 14.94 (3.93, 27.13) |  | 16.31 (-4.44, 41.58) | 10.95 (7.12, 14.92) | 11.04 (1.54, 21.43) |
| 7.5^th^ (3d) ^a^ | 15.71 (-5.91, 42.28) | 12.57 (8.36, 16.95) | 13.76 (2.98, 25.66) |  | 15.07 (-5.35, 39.89) | 10.46 (6.66, 14.39) | 10.38 (1.00, 20.64) |
| 7.5^th^ (4d) ^a^ | 17.25 (-5.05, 44.79) | 13.45 (9.14, 17.92) | 10.79 (1.35, 21.12) |  | 15.66 (-4.95, 40.75) | 10.73 (6.92, 14.69) | 14.98 (3.93, 27.21) |
| 5^th^ (2d) ^a^ | 14.90 (-6.50, 41.20) | 13.01 (8.80, 17.38) | 15.14 (4.24, 27.17) |  | 14.82 (-5.54, 39.57) | 10.55 (6.76, 14.48) | 10.84 (1.41, 21.14) |
| 5^th^ (3d) ^a^ | 12.90 (-8.04, 38.60) | 12.96 (8.76, 17.32) | 13.88 (3.13, 25.75) |  | 14.21 (-6.01, 38.77) | 10.42 (6.63, 14.34) | 10.32 (0.96, 20.56) |
| 5^th^ (4d) ^a^ | 16.09 (-5.25, 42.24) | 12.48 (8.33, 16.79) | 12.66 (2.15, 24.26) |  | 15.27 (-5.16, 40.09) | 10.33 (6.55, 14.25) | 10.04 (0.71, 20.23) |
| 2.5^th^ (2d) ^a^ | 15.41 (-5.71, 41.27) | 12.10 (7.99, 16.37) | 11.75 (1.37, 23.20) |  | 14.91 (-5.42, 39.60) | 10.19 (6.42, 14.10) | 9.88 (0.58, 20.05) |
| 2.5^th^ (3d) ^a^ | 14.73 (-6.26, 40.41) | 12.01 (7.91, 16.27) | 11.25 (0.91, 22.65) |  | 14.75 (-5.54, 39.40) | 10.20 (6.42, 14.10) | 9.80 (0.50, 19.96) |
| 2.5^th^ (4d) ^a^ |  |  |  |  |  |  |  |
| Shenzhen | -8.83 (-21.14, 5.40) | 12.86 (9.76, 16.06) | 18.93 (8.19, 30.74) |  | -5.88 (-18.50, 8.69) | 5.73 (2.74, 8.80) | 13.00 (2.42, 24.69) |
| 10^th^ (2d) ^a^ | -11.09 (-23.33, 3.12) | 12.36 (9.21, 15.60) | 18.30 (7.44, 30.26) |  | -6.33 (-19.01, 8.34) | 5.58 (2.59, 8.67) | 12.73 (2.15, 24.40) |
| 10^th^ (3d) ^a^ | -10.62 (-22.89, 3.59) | 11.32 (8.23, 14.49) | 16.93 (6.31, 28.60) |  | -6.98 (-19.59, 7.60) | 5.01 (2.03, 8.07) | 11.97 (1.50, 23.52) |
| 10^th^ (4d) ^a^ | -9.08 (-21.45, 5.24) | 12.57 (9.45, 15.79) | 17.84 (7.08, 29.69) |  | -5.98 (-18.56, 8.56) | 5.27 (2.31, 8.33) | 11.74 (1.30, 23.25) |
| 7.5^th^ (2d) ^a^ | -10.93 (-23.23, 3.34) | 11.96 (8.81, 15.19) | 18.99 (7.99, 31.11) |  | -6.20 (-18.73, 8.26) | 4.89 (1.95, 7.91) | 12.08 (1.69, 23.53) |
| 7.5^th^ (3d) ^a^ | -9.84 (-22.16, 4.42) | 10.70 (7.65, 13.84) | 16.21 (5.67, 27.80) |  | -7.02 (-19.42, 7.29) | 4.36 (1.44, 7.36) | 11.07 (0.81, 22.37) |
| 7.5^th^ (4d) ^a^ | -8.85 (-21.38, 5.68) | 12.31 (9.15, 15.56) | 15.60 (4.92, 27.37) |  | -5.72 (-18.22, 8.69) | 4.63 (1.71, 7.64) | 10.29 (0.11, 21.51) |
| 5^th^ (2d) ^a^ | -12.06 (-24.11, 1.91) | 9.78 (6.73, 12.91) | 14.13 (3.75, 25.54) |  | -6.67 (-19.08, 7.65) | 4.28 (1.36, 7.28) | 10.62 (0.41, 21.86) |
| 5^th^ (3d) ^a^ | -11.47 (-23.58, 2.56) | 10.19 (7.15, 13.32) | 14.78 (4.39, 26.21) |  | -7.52 (-19.81, 6.67) | 4.23 (1.32, 7.23) | 10.81 (0.59, 22.07) |
| 5^th^ (4d) ^a^ | -8.37 (-20.50, 5.62) | 12.15 (9.13, 15.25) | 16.39 (6.08, 27.69) |  | -5.86 (-18.22, 8.37) | 4.36 (1.47, 7.33) | 10.63 (0.50, 21.78) |
| 2.5^th^ (2d) ^a^ | -11.41 (-23.18, 2.17) | 10.78 (7.79, 13.86) | 14.55 (4.44, 25.65) |  | -6.36 (-18.74, 7.92) | 4.36 (1.46, 7.35) | 11.05 (0.84, 22.29) |
| 2.5^th^ (3d) ^a^ | -11.78 (-23.53, 1.78) | 10.70 (7.71, 13.78) | 14.47 (4.34, 25.58) |  | -7.40 (-19.72, 6.80) | 4.03 (1.12, 7.02) | 10.94 (0.70, 22.21) |
| 2.5^th^ (4d) ^a^ |  |  |  |  |  |  |  |
| Zhaoqing | 9.14 (-17.87, 45.02) | -0.43 (-5.44, 4.86) | 5.19 (-5.53, 17.14) |  | 32.82 (-23.74, 131.32) | 8.70 (-0.78, 19.08) | 7.09 (-13.01, 31.83) |
| 10^th^ (2d) ^b^ | 9.62 (-17.28, 45.26) | -0.26 (-5.23, 4.97) | 5.87 (-4.84, 17.78) |  | 33.30 (-23.38, 131.92) | 8.56 (-0.87, 18.90) | 7.72 (-12.46, 32.55) |
| 10^th^ (3d) ^b^ | 12.55 (-14.77, 48.62) | 0.58 (-4.39, 5.82) | 7.48 (-3.30, 19.47) |  | 35.53 (-21.82, 134.92) | 8.15 (-1.22, 18.40) | 8.92 (-11.36, 33.84) |
| 10^th^ (4d) ^b^ | 9.37 (-17.43, 44.87) | 0.23 (-4.75, 5.48) | 5.66 (-5.00, 17.50) |  | 32.86 (-23.62, 131.11) | 9.40 (-0.09, 19.79) | 7.88 (-12.20, 32.56) |
| 7.5^th^ (2d) ^b^ | 12.02 (-14.91, 47.48) | 0.05 (-4.85, 5.20) | 5.30 (-5.14, 16.90) |  | 37.24 (-20.57, 137.11) | 8.89 (-0.49, 19.16) | 7.54 (-12.32, 31.89) |
| 7.5^th^ (3d) ^b^ | 12.07 (-14.82, 47.45) | 0.31 (-4.61, 5.48) | 5.74 (-4.75, 17.39) |  | 35.45 (-21.50, 133.72) | 7.82 (-1.48, 18.00) | 7.28 (-12.54, 31.59) |
| 7.5^th^ (4d) ^b^ | 10.15 (-16.36, 45.06) | 0.04 (-4.86, 5.18) | 6.23 (-4.32, 17.95) |  | 33.82 (-22.71, 131.71) | 9.10 (-0.30, 19.38) | 8.68 (-11.40, 33.32) |
| 5^th^ (2d) ^b^ | 11.36 (-15.26, 46.34) | 0.05 (-4.83, 5.18) | 5.97 (-4.52, 17.61) |  | 37.50 (-20.10, 136.62) | 9.08 (-0.29, 19.33) | 8.09 (-11.85, 32.54) |
| 5^th^ (3d) ^b^ | 11.13 (-15.48, 46.12) | -0.07 (-4.97, 5.07) | 6.20 (-4.33, 17.89) |  | 35.49 (-21.33, 133.34) | 7.64 (-1.63, 17.79) | 7.47 (-12.39, 31.82) |

Note: SO_2_, sulfur dioxide; NO_2_, nitrogen dioxide.

^a^ Cold spells were defined by percentile temperature thresholds (10^th^, 7.5^th^, 5^th^ and 2.5^th^) and by the number of consecutive days below the thresholds (2-4 d).

^b^ Nine definitions [10^th^ (2d), 10^th^ (3d), 10^th^ (4d), 7.5^th^ (2d), 7.5^th^ (3d), 7.5^th^ (4d), 5^th^ (2d), 5^th^ (3d), and 5^th^ (4d)] were used in Zhaoqing.

^c^ Air pollutants at lag03 were included in each model separately.

## Table S10. Independent effects of cold spells on anxiety stratified by sex when PM_2.5_, PM_10_, NO_2_ and SO_2_ at lag03 were included in each model separately.

| Cold spell | PM_2.5_ ^c^ | |  | PM_10_ ^c^ | |  | NO_2_ ^c^ | |  | SO_2_ ^c^ | |
| --- | --- | --- | --- | --- | --- | --- | --- | --- | --- | --- | --- |
|  | male | female |  | male | female |  | male | female |  | male | female |
| Huizhou |  |  |  |  |  |  |  |  |  |  |  |
| 10^th^ (2d) ^a^ | 12.55 (4.54, 21.17) | 9.89 (2.64, 17.65) |  | 14.44 (6.21, 23.31) | 10.98 (3.57, 18.91) |  | 16.25 (7.94, 25.19) | 13.20 (5.69, 21.25) |  | 13.81 (5.87, 22.34) | 10.94 (3.74, 18.63) |
| 10^th^ (3d) ^a^ | 9.74 (1.43, 18.73) | 9.80 (2.15, 18.04) |  | 11.34 (2.85, 20.54) | 10.77 (2.98, 19.14) |  | 13.74 (5.07, 23.13) | 13.49 (5.50, 22.07) |  | 10.32 (2.16, 19.14) | 10.28 (2.75, 18.35) |
| 10^th^ (4d) ^a^ | 8.04 (-0.87, 17.76) | 9.90 (1.56, 18.93) |  | 9.28 (0.24, 19.14) | 10.65 (2.22, 19.78) |  | 11.80 (2.50, 21.94) | 13.58 (4.87, 23.02) |  | 8.12 (-0.65, 17.67) | 9.94 (1.74, 18.80) |
| 7.5^th^ (2d) ^a^ | 13.56 (4.71, 23.15) | 9.56 (1.63, 18.11) |  | 15.46 (6.39, 25.30) | 10.59 (2.52, 19.30) |  | 19.02 (9.59, 29.26) | 14.49 (6.03, 23.62) |  | 14.45 (5.73, 23.90) | 10.44 (2.60, 18.88) |
| 7.5^th^ (3d) ^a^ | 11.49 (2.37, 21.43) | 11.00 (2.67, 20.01) |  | 12.93 (3.65, 23.04) | 11.85 (3.42, 20.98) |  | 16.53 (6.84, 27.10) | 15.83 (6.96, 25.43) |  | 12.11 (3.08, 21.92) | 11.52 (3.28, 20.41) |
| 7.5^th^ (4d) ^a^ | 7.92 (-2.21, 19.09) | 9.71 (0.37, 19.91) |  | 9.17 (-1.10, 20.51) | 10.44 (1.01, 20.74) |  | 12.99 (2.20, 24.91) | 14.59 (4.64, 25.49) |  | 8.19 (-1.84, 19.26) | 9.82 (0.59, 19.90) |
| 5^th^ (2d) ^a^ | 16.39 (6.44, 27.28) | 5.55 (-2.78, 14.59) |  | 18.41 (8.20, 29.59) | 6.50 (-1.98, 15.72) |  | 23.20 (12.38, 35.06) | 10.63 (1.64, 20.41) |  | 16.85 (7.01, 27.60) | 6.10 (-2.17, 15.05) |
| 5^th^ (3d) ^a^ | 15.57 (4.58, 27.72) | 12.93 (3.20, 23.58) |  | 17.03 (5.85, 29.39) | 13.79 (3.94, 24.58) |  | 21.46 (9.68, 34.51) | 18.21 (7.78, 29.64) |  | 15.97 (5.01, 28.08) | 13.21 (3.52, 23.81) |
| 5^th^ (4d) ^a^ | 14.48 (2.13, 28.33) | 14.74 (3.62, 27.06) |  | 15.88 (3.33, 29.94) | 15.61 (4.36, 28.07) |  | 21.03 (7.70, 36.01) | 20.92 (8.91, 34.26) |  | 14.63 (2.32, 28.41) | 14.82 (3.75, 27.08) |
| 2.5^th^ (2d) ^a^ | 18.85 (6.07, 33.18) | 12.23 (1.22, 24.44) |  | 20.28 (7.30, 34.83) | 13.01 (1.89, 25.35) |  | 24.25 (10.73, 39.43) | 16.72 (5.12, 29.60) |  | 18.69 (5.99, 32.91) | 12.01 (1.09, 24.11) |
| 2.5^th^ (3d) ^a^ | 13.95 (0.12, 29.69) | 15.01 (2.45, 29.10) |  | 14.93 (0.95, 30.85) | 15.62 (2.97, 29.82) |  | 19.50 (4.79, 36.27) | 20.12 (6.80, 35.10) |  | 14.10 (0.26, 29.85) | 15.06 (2.51, 29.15) |
| 2.5^th^ (4d) ^a^ | 12.03 (-2.91, 29.26) | 15.90 (1.83, 31.90) |  | 12.97 (-2.12, 30.38) | 16.51 (2.35, 32.63) |  | 17.84 (1.92, 36.26) | 21.68 (6.68, 38.79) |  | 12.25 (-2.71, 29.52) | 16.17 (2.07, 32.21) |
| Shenzhen | male | female |  | male | female |  | male | female |  | male | female |
| 10^th^ (2d) ^a^ | 5.68 (1.27, 10.29) | 8.60 (4.35, 13.03) |  | 6.60 (2.12, 11.28) | 9.02 (4.72, 13.49) |  | 8.69 (4.21, 13.36) | 11.84 (7.52, 16.35) |  | 5.72 (1.46, 10.16) | 9.47 (5.31, 13.80) |
| 10^th^ (3d) ^a^ | 5.61 (0.81, 10.63) | 7.78 (3.20, 12.56) |  | 6.51 (1.66, 11.60) | 8.13 (3.52, 12.94) |  | 8.71 (3.87, 13.78) | 11.26 (6.62, 16.10) |  | 5.01 (0.50, 9.73) | 8.21 (3.85, 12.75) |
| 10^th^ (4d) ^a^ | 3.02 (-1.66, 7.92) | 7.08 (2.55, 11.81) |  | 3.68 (-1.04, 8.63) | 7.33 (2.78, 12.08) |  | 5.89 (1.11, 10.89) | 10.33 (5.70, 15.18) |  | 2.79 (-1.70, 7.50) | 7.63 (3.23, 12.21) |
| 7.5^th^ (2d) ^a^ | 3.74 (-0.88, 8.57) | 5.71 (1.32, 10.30) |  | 4.56 (-0.12, 9.47) | 6.04 (1.60, 10.67) |  | 7.68 (2.85, 12.75) | 9.95 (5.32, 14.78) |  | 4.08 (-0.45, 8.82) | 6.92 (2.55, 11.48) |
| 7.5^th^ (3d) ^a^ | 5.03 (0.06, 10.24) | 5.45 (0.78, 10.32) |  | 5.72 (0.70, 11.00) | 5.70 (1.00, 10.61) |  | 9.32 (4.02, 14.90) | 9.94 (4.94, 15.17) |  | 4.67 (-0.15, 9.72) | 5.97 (1.40, 10.76) |
| 7.5^th^ (4d) ^a^ | 3.40 (-1.89, 8.98) | 4.02 (-0.98, 9.27) |  | 3.91 (-1.42, 9.54) | 4.18 (-0.83, 9.46) |  | 7.06 (1.47, 12.97) | 7.82 (2.53, 13.38) |  | 3.04 (-2.14, 8.49) | 4.29 (-0.63, 9.45) |
| 5^th^ (2d) ^a^ | 2.44 (-2.59, 7.74) | 2.10 (-2.58, 6.99) |  | 3.32 (-1.80, 8.70) | 2.38 (-2.34, 7.33) |  | 7.59 (2.17, 13.30) | 7.23 (2.19, 12.52) |  | 2.55 (-2.34, 7.70) | 3.10 (-1.50, 7.92) |
| 5^th^ (3d) ^a^ | -3.44 (-8.76, 2.19) | -1.11 (-6.17, 4.21) |  | -2.88 (-8.26, 2.81) | -0.95 (-6.03, 4.41) |  | 0.37 (-5.31, 6.38) | 2.87 (-2.53, 8.57) |  | -3.36 (-8.60, 2.18) | -0.42 (-5.43, 4.86) |
| 5^th^ (4d) ^a^ | -0.17 (-6.47, 6.55) | 2.03 (-3.98, 8.42) |  | 0.36 (-5.98, 7.13) | 2.20 (-3.83, 8.61) |  | 4.29 (-2.46, 11.50) | 6.65 (0.21, 13.51) |  | -0.51 (-6.68, 6.07) | 2.26 (-3.67, 8.55) |
| 2.5^th^ (2d) ^a^ | 14.38 (7.44, 21.78) | 12.27 (5.78, 19.16) |  | 15.15 (8.14, 22.61) | 12.54 (6.02, 19.46) |  | 18.23 (11.02, 25.91) | 16.05 (9.31, 23.21) |  | 13.67 (6.86, 20.90) | 12.22 (5.82, 19.02) |
| 2.5^th^ (3d) ^a^ | 9.06 (1.43, 17.26) | 8.73 (1.57, 16.39) |  | 9.47 (1.80, 17.72) | 8.85 (1.68, 16.53) |  | 13.17 (5.11, 21.84) | 12.53 (4.99, 20.60) |  | 8.60 (1.03, 16.73) | 8.57 (1.44, 16.20) |
| 2.5^th^ (4d) ^a^ | 9.53 (1.38, 18.35) | 9.74 (2.11, 17.94) |  | 9.91 (1.72, 18.77) | 9.85 (2.21, 18.07) |  | 14.11 (5.45, 23.49) | 13.99 (5.91, 22.68) |  | 8.87 (0.79, 17.60) | 9.34 (1.76, 17.49) |
| Zhaoqing | male | female |  | male | female |  | male | female |  | male | female |
| 10^th^ (2d) ^b^ | -6.86 (-17.77, 5.50) | -4.78 (-15.50, 7.30) |  | -7.47 (-18.39, 4.91) | -5.34 (-16.07, 6.75) |  | -8.44 (-19.15, 3.69) | -4.39 (-15.12, 7.68) |  | -8.01 (-18.50, 3.84) | -2.77 (-13.45, 9.23) |
| 10^th^ (3d) ^b^ | -9.96 (-21.08, 2.72) | -0.50 (-12.16, 12.71) |  | -10.44 (-21.56, 2.27) | -0.94 (-12.61, 12.28) |  | -11.11 (-22.15, 1.50) | -0.24 (-11.99, 13.08) |  | -10.76 (-21.63, 1.62) | 1.10 (-10.61, 14.35) |
| 10^th^ (4d) ^b^ | 0.15 (-13.02, 15.32) | 7.92 (-5.52, 23.28) |  | -0.14 (-13.33, 15.07) | 7.57 (-5.88, 22.95) |  | -0.34 (-13.57, 14.92) | 8.38 (-5.23, 23.95) |  | -0.87 (-13.82, 14.01) | 9.21 (-4.30, 24.62) |
| 7.5^th^ (2d) ^b^ | -6.72 (-18.52, 6.79) | 1.02 (-11.03, 14.70) |  | -7.09 (-18.92, 6.47) | 0.58 (-11.48, 14.30) |  | -7.87 (-19.59, 5.57) | 1.27 (-10.88, 15.09) |  | -7.62 (-19.16, 5.57) | 2.54 (-9.57, 16.28) |
| 7.5^th^ (3d) ^b^ | -10.12 (-22.90, 4.78) | -3.47 (-16.23, 11.24) |  | -10.25 (-23.05, 4.68) | -3.75 (-16.51, 10.94) |  | -10.50 (-23.30, 4.44) | -3.41 (-16.27, 11.42) |  | -10.77 (-23.42, 3.97) | -2.58 (-15.43, 12.23) |
| 7.5^th^ (4d) ^b^ | -8.03 (-22.34, 8.91) | 5.00 (-10.01, 22.52) |  | -8.24 (-22.54, 8.70) | 4.74 (-10.26, 22.24) |  | -8.18 (-22.57, 8.88) | 5.26 (-9.92, 22.99) |  | -8.80 (-22.95, 7.94) | 5.83 (-9.27, 23.44) |
| 5^th^ (2d) ^b^ | -15.26 (-28.41, 0.30) | 2.47 (-11.90, 19.18) |  | -15.53 (-28.68, 0.04) | 2.12 (-12.24, 18.84) |  | -16.09 (-29.15, -0.61) | 2.68 (-11.77, 19.49) |  | -16.01 (-28.97, -0.69) | 3.57 (-10.87, 20.35) |
| 5^th^ (3d) ^b^ | -21.45 (-35.19, -4.80) | 5.27 (-10.83, 24.29) |  | -21.52 (-35.27, -4.85) | 5.02 (-11.07, 24.02) |  | -21.61 (-35.40, -4.88) | 5.43 (-10.81, 24.61) |  | -22.25 (-35.82, -5.81) | 5.78 (-10.37, 24.83) |
| 5^th^ (4d) ^b^ | -18.61 (-33.83, 0.10) | 5.07 (-12.21, 25.75) |  | -18.80 (-34.00, -0.11) | 4.78 (-12.47, 25.43) |  | -18.71 (-34.01, 0.13) | 5.33 (-12.13, 26.26) |  | -19.56 (-34.56, -1.13) | 5.72 (-11.62, 26.45) |

Note: PM, particulate matter; SO_2_, sulfur dioxide; NO_2_, nitrogen dioxide.

^a^ Cold spells were defined by percentile temperature thresholds (10^th^, 7.5^th^, 5^th^ and 2.5^th^) and by the number of consecutive days below the thresholds (2-4 d).

^b^ Nine definitions [10^th^ (2d), 10^th^ (3d), 10^th^ (4d), 7.5^th^ (2d), 7.5^th^ (3d), 7.5^th^ (4d), 5^th^ (2d), 5^th^ (3d), and 5^th^ (4d)] were used in Zhaoqing.

^c^ Air pollutants at lag03 were included in each model separately.

## Table S11. Independent effects of air pollution on anxiety stratified by sex when cold spells under different definitions were controlled.

| Cold spell | PM_2.5_ ^c^ | |  | PM_10_ ^c^ | |  | NO_2_ ^c^ | |  | SO_2_ ^c^ | |
| --- | --- | --- | --- | --- | --- | --- | --- | --- | --- | --- | --- |
|  | male | female |  | male | female |  | male | female |  | male | female |
| Huizhou |  |  |  |  |  |  |  |  |  |  |  |
| 10^th^ (2d) ^a^ | 1.69 (0.35, 3.04) | 1.35 (0.12, 2.59) |  | 1.86 (0.84, 2.89) | 1.33 (0.40, 2.27) |  | 15.16 (9.32, 21.32) | 12.85 (7.53, 18.43) |  | 13.39 (8.15, 18.90) | 10.38 (5.52, 15.46) |
| 10^th^ (3d) ^a^ | 1.51 (0.18, 2.86) | 1.31 (0.09, 2.55) |  | 1.68 (0.67, 2.70) | 1.28 (0.35, 2.22) |  | 14.44 (8.64, 20.56) | 12.78 (7.46, 18.36) |  | 12.56 (7.39, 17.97) | 9.95 (5.14, 14.97) |
| 10^th^ (4d) ^a^ | 1.35 (0.04, 2.68) | 1.21 (0.00, 2.44) |  | 1.52 (0.53, 2.53) | 1.18 (0.26, 2.10) |  | 13.54 (7.85, 19.53) | 12.24 (7.00, 17.74) |  | 12.03 (6.92, 17.38) | 9.49 (4.74, 14.45) |
| 7.5^th^ (2d) ^a^ | 1.67 (0.33, 3.02) | 1.30 (0.07, 2.54) |  | 1.82 (0.81, 2.85) | 1.27 (0.34, 2.21) |  | 15.78 (9.84, 22.04) | 13.25 (7.83, 18.94) |  | 13.09 (7.88, 18.55) | 10.06 (5.24, 15.10) |
| 7.5^th^ (3d) ^a^ | 1.48 (0.16, 2.82) | 1.28 (0.06, 2.50) |  | 1.64 (0.64, 2.65) | 1.23 (0.31, 2.16) |  | 14.68 (8.87, 20.80) | 13.06 (7.71, 18.68) |  | 12.48 (7.33, 17.88) | 9.84 (5.06, 14.84) |
| 7.5^th^ (4d) ^a^ | 1.29 (-0.02, 2.61) | 1.13 (-0.07, 2.35) |  | 1.47 (0.48, 2.46) | 1.11 (0.20, 2.02) |  | 13.52 (7.82, 19.53) | 12.20 (6.94, 17.71) |  | 11.93 (6.83, 17.28) | 9.32 (4.59, 14.26) |
| 5^th^ (2d) ^a^ | 1.57 (0.26, 2.90) | 1.06 (-0.15, 2.27) |  | 1.76 (0.76, 2.77) | 1.07 (0.15, 1.99) |  | 15.81 (9.91, 22.04) | 12.02 (6.71, 17.59) |  | 12.65 (7.50, 18.04) | 9.26 (4.52, 14.21) |
| 5^th^ (3d) ^a^ | 1.37 (0.07, 2.69) | 1.13 (-0.06, 2.33) |  | 1.56 (0.58, 2.55) | 1.13 (0.22, 2.04) |  | 14.36 (8.64, 20.38) | 12.46 (7.22, 17.95) |  | 12.15 (7.05, 17.51) | 9.37 (4.64, 14.31) |
| 5^th^ (4d) ^a^ | 1.29 (0.00, 2.60) | 1.10 (-0.09, 2.30) |  | 1.48 (0.51, 2.47) | 1.10 (0.20, 2.00) |  | 13.90 (8.21, 19.89) | 12.40 (7.17, 17.88) |  | 11.92 (6.83, 17.25) | 9.23 (4.51, 14.16) |
| 2.5^th^ (2d) ^a^ | 1.33 (0.04, 2.64) | 1.05 (-0.14, 2.25) |  | 1.52 (0.54, 2.51) | 1.05 (0.16, 1.96) |  | 13.82 (8.18, 19.75) | 11.60 (6.46, 16.98) |  | 11.93 (6.84, 17.25) | 9.06 (4.36, 13.97) |
| 2.5^th^ (3d) ^a^ | 1.18 (-0.11, 2.48) | 0.97 (-0.21, 2.17) |  | 1.38 (0.41, 2.36) | 0.99 (0.10, 1.89) |  | 13.00 (7.43, 18.86) | 11.44 (6.34, 16.79) |  | 11.71 (6.63, 17.02) | 8.97 (4.27, 13.87) |
| 2.5^th^ (4d) ^a^ | 1.16 (-0.12, 2.46) | 0.96 (-0.22, 2.15) |  | 1.36 (0.39, 2.34) | 0.97 (0.08, 1.87) |  | 12.77 (7.21, 18.61) | 11.37 (6.27, 16.72) |  | 11.68 (6.61, 16.99) | 8.98 (4.29, 13.89) |
| Shenzhen | male | female |  | male | female |  | male | female |  | male | female |
| 10^th^ (2d) ^a^ | 0.52 (-0.21, 1.26) | 0.28 (-0.40, 0.96) |  | 0.76 (0.16, 1.37) | 0.38 (-0.18, 0.94) |  | 13.06 (8.78, 17.51) | 12.16 (8.19, 16.27) |  | 5.15 (1.05, 9.41) | 6.48 (2.63, 10.47) |
| 10^th^ (3d) ^a^ | 0.68 (-0.08, 1.44) | 0.43 (-0.26, 1.13) |  | 0.84 (0.22, 1.46) | 0.46 (-0.11, 1.03) |  | 12.39 (8.06, 16.90) | 11.65 (7.63, 15.83) |  | 4.83 (0.72, 9.10) | 6.48 (2.62, 10.48) |
| 10^th^ (4d) ^a^ | 0.49 (-0.25, 1.23) | 0.32 (-0.36, 1.00) |  | 0.65 (0.04, 1.25) | 0.35 (-0.21, 0.91) |  | 11.08 (6.85, 15.49) | 10.93 (6.97, 15.03) |  | 4.10 (0.02, 8.35) | 6.00 (2.16, 9.98) |
| 7.5^th^ (2d) ^a^ | 0.38 (-0.35, 1.11) | 0.08 (-0.59, 0.75) |  | 0.63 (0.03, 1.23) | 0.20 (-0.35, 0.75) |  | 12.80 (8.50, 17.27) | 11.76 (7.77, 15.89) |  | 4.77 (0.69, 9.02) | 5.88 (2.06, 9.84) |
| 7.5^th^ (3d) ^a^ | 0.53 (-0.19, 1.26) | 0.18 (-0.48, 0.85) |  | 0.70 (0.11, 1.30) | 0.25 (-0.30, 0.80) |  | 12.37 (8.03, 16.88) | 11.11 (7.10, 15.28) |  | 4.38 (0.34, 8.58) | 5.60 (1.82, 9.52) |
| 7.5^th^ (4d) ^a^ | 0.43 (-0.28, 1.15) | 0.09 (-0.57, 0.75) |  | 0.59 (0.00, 1.17) | 0.15 (-0.39, 0.69) |  | 11.04 (6.82, 15.42) | 9.90 (6.00, 13.94) |  | 3.81 (-0.20, 7.99) | 5.02 (1.26, 8.91) |
| 5^th^ (2d) ^a^ | 0.31 (-0.41, 1.03) | -0.08 (-0.74, 0.59) |  | 0.57 (-0.03, 1.16) | 0.05 (-0.49, 0.61) |  | 12.80 (8.44, 17.34) | 11.07 (7.06, 15.24) |  | 4.34 (0.32, 8.52) | 5.01 (1.27, 8.90) |
| 5^th^ (3d) ^a^ | 0.25 (-0.46, 0.97) | -0.04 (-0.70, 0.62) |  | 0.45 (-0.14, 1.03) | 0.05 (-0.49, 0.59) |  | 9.82 (5.63, 14.17) | 8.98 (5.09, 13.01) |  | 3.68 (-0.32, 7.85) | 4.96 (1.22, 8.85) |
| 5^th^ (4d) ^a^ | 0.33 (-0.38, 1.05) | 0.02 (-0.64, 0.68) |  | 0.50 (-0.08, 1.09) | 0.09 (-0.45, 0.63) |  | 10.28 (6.08, 14.64) | 9.40 (5.52, 13.43) |  | 3.66 (-0.35, 7.83) | 4.86 (1.12, 8.74) |
| 2.5^th^ (2d) ^a^ | 0.47 (-0.23, 1.18) | 0.04 (-0.61, 0.70) |  | 0.69 (0.11, 1.27) | 0.16 (-0.38, 0.69) |  | 12.86 (8.68, 17.20) | 10.87 (7.04, 14.85) |  | 4.18 (0.20, 8.33) | 4.70 (0.99, 8.53) |
| 2.5^th^ (3d) ^a^ | 0.43 (-0.28, 1.13) | 0.06 (-0.59, 0.71) |  | 0.59 (0.02, 1.17) | 0.13 (-0.40, 0.66) |  | 11.12 (7.00, 15.40) | 9.59 (5.80, 13.53) |  | 3.93 (-0.06, 8.09) | 4.94 (1.21, 8.81) |
| 2.5^th^ (4d) ^a^ | 0.41 (-0.29, 1.12) | 0.04 (-0.60, 0.70) |  | 0.56 (-0.01, 1.14) | 0.11 (-0.42, 0.65) |  | 10.93 (6.80, 15.21) | 9.59 (5.78, 13.53) |  | 3.54 (-0.47, 7.70) | 4.65 (0.91, 8.52) |
| Zhaoqing | male | female |  | male | female |  | male | female |  | male | female |
| 10^th^ (2d) ^b^ | 1.88 (-0.19, 3.99) | -0.54 (-2.47, 1.42) |  | 1.16 (-0.58, 2.94) | -0.69 (-2.32, 0.98) |  | 2.97 (-3.65, 10.05) | -1.10 (-7.20, 5.40) |  | 12.43 (-0.26, 26.73) | 5.59 (-5.82, 18.37) |
| 10^th^ (3d) ^b^ | 1.81 (-0.21, 3.88) | -0.33 (-2.22, 1.60) |  | 1.10 (-0.61, 2.84) | -0.49 (-2.09, 1.14) |  | 2.77 (-3.78, 9.76) | -0.45 (-6.52, 6.02) |  | 11.68 (-0.89, 25.85) | 6.27 (-5.15, 19.07) |
| 10^th^ (4d) ^b^ | 1.98 (-0.03, 4.03) | -0.19 (-2.07, 1.72) |  | 1.26 (-0.43, 2.98) | -0.35 (-1.94, 1.27) |  | 3.94 (-2.63, 10.95) | 0.42 (-5.66, 6.88) |  | 11.09 (-1.38, 25.13) | 6.54 (-4.86, 19.30) |
| 7.5^th^ (2d) ^b^ | 1.99 (-0.04, 4.06) | -0.27 (-2.16, 1.65) |  | 1.27 (-0.44, 3.02) | -0.43 (-2.03, 1.20) |  | 3.35 (-3.22, 10.37) | -0.26 (-6.34, 6.20) |  | 12.88 (0.20, 27.16) | 6.55 (-4.88, 19.34) |
| 7.5^th^ (3d) ^b^ | 2.02 (0.03, 4.05) | -0.36 (-2.22, 1.52) |  | 1.30 (-0.38, 3.00) | -0.51 (-2.08, 1.08) |  | 3.49 (-2.98, 10.39) | -0.67 (-6.62, 5.67) |  | 12.73 (0.15, 26.89) | 5.90 (-5.36, 18.51) |
| 7.5^th^ (4d) ^b^ | 1.87 (-0.12, 3.90) | -0.32 (-2.17, 1.57) |  | 1.16 (-0.51, 2.86) | -0.46 (-2.03, 1.13) |  | 3.42 (-3.06, 10.33) | -0.05 (-6.05, 6.32) |  | 10.79 (-1.60, 24.73) | 5.81 (-5.45, 18.41) |
| 5^th^ (2d) ^b^ | 1.93 (-0.07, 3.97) | -0.26 (-2.12, 1.64) |  | 1.22 (-0.46, 2.93) | -0.41 (-1.99, 1.19) |  | 3.19 (-3.26, 10.08) | -0.24 (-6.22, 6.12) |  | 12.83 (0.24, 26.99) | 6.42 (-4.90, 19.09) |
| 5^th^ (3d) ^b^ | 1.98 (-0.00, 4.00) | -0.26 (-2.11, 1.62) |  | 1.25 (-0.42, 2.94) | -0.41 (-1.98, 1.18) |  | 3.07 (-3.35, 9.91) | -0.14 (-6.11, 6.21) |  | 12.98 (0.42, 27.12) | 6.22 (-5.06, 18.83) |
| 5^th^ (4d) ^b^ | 1.80 (-0.18, 3.82) | -0.33 (-2.18, 1.56) |  | 1.09 (-0.57, 2.78) | -0.47 (-2.04, 1.12) |  | 2.93 (-3.51, 9.79) | -0.11 (-6.10, 6.25) |  | 10.86 (-1.50, 24.78) | 5.65 (-5.58, 18.22) |

Note: PM, particulate matter; SO_2_, sulfur dioxide; NO_2_, nitrogen dioxide.

^a^ Cold spells were defined by percentile temperature thresholds (10^th^, 7.5^th^, 5^th^ and 2.5^th^) and by the number of consecutive days below the thresholds (2-4 d).

^b^ Nine definitions [10^th^ (2d), 10^th^ (3d), 10^th^ (4d), 7.5^th^ (2d), 7.5^th^ (3d), 7.5^th^ (4d), 5^th^ (2d), 5^th^ (3d), and 5^th^ (4d)] were used in Zhaoqing.

^c^ Air pollutants at lag03 were included in each model separately.

## Table S12. Percent changes and relative excess risk due to interaction (RERI) of cold spells and PM_2.5_ exposure on anxiety stratified by sex and age in three subtropical cities in China.

| Cold spells | PM_2.5_ ^c^ (µg/m^3^) | male | female | < 18 | 18-65 | ≥ 65 |
| --- | --- | --- | --- | --- | --- | --- |
| Huizhou |  |  |  |  |  |  |
| 10^th^ (2d) ^a^ |  |  |  |  |  |  |
| No | < 46.03 | 1.00 | 1.00 | 1.00 | 1.00 | 1.00 |
| No | ≥ 46.03 | 4.94 (-1.25, 11.52) | 3.78 (-1.92, 9.82) | 5.33 (-16.07, 32.18) | 4.86 (0.23, 9.70) | 0.06 (-11.00, 12.50) |
| Yes | < 46.03 | 5.03 (-5.32, 16.53) | 3.21 (-6.31, 13.71) | 26.16 (-14.20, 85.51) | 2.37 (-5.22, 10.58) | 9.90 (-10.20, 34.51) |
| Yes | ≥ 46.03 | 22.08 (10.33, 35.08) | 17.69 (7.18, 29.24) | 9.75 (-26.67, 64.24) | 21.32 (12.58, 30.74) | 11.47 (-8.18, 35.33) |
| RERI |  | 0.12 (-0.02, 0.26) | 0.11 (-0.02, 0.23) | -0.22 (-0.79, 0.36) | 0.14 (0.04, 0.24) | 0.02 (-0.25, 0.28) |
| 10^th^ (3d) ^a^ |  |  |  |  |  |  |
| No | < 46.03 | 1.00 | 1.00 | 1.00 | 1.00 | 1.00 |
| No | ≥ 46.03 | 4.17 (-1.82, 10.53) | 3.84 (-1.73, 9.74) | 3.96 (-16.89, 30.06) | 4.62 (0.12, 9.33) | -0.41 (-11.18, 11.65) |
| Yes | < 46.03 | 0.39 (-10.33, 12.39) | 2.82 (-7.32, 14.07) | 24.17 (-19.44, 91.37) | 0.09 (-7.90, 8.76) | 7.30 (-13.73, 33.45) |
| Yes | ≥ 46.03 | 19.34 (7.44, 32.57) | 17.43 (6.56, 29.41) | 8.17 (-29.72, 66.49) | 20.19 (11.23, 29.86) | 8.43 (-11.47, 32.81) |
| RERI |  | 0.15 (0.01, 0.29) | 0.11 (-0.03, 0.24) | -0.20 (-0.84, 0.44) | 0.15 (0.05, 0.26) | 0.02 (-0.27, 0.30) |
| 10^th^ (4d) ^a^ |  |  |  |  |  |  |
| No | < 46.03 | 1.00 | 1.00 | 1.00 | 1.00 | 1.00 |
| No | ≥ 46.03 | 4.15 (-1.67, 10.32) | 3.97 (-1.44, 9.69) | -0.20 (-19.56, 23.83) | 4.76 (0.39, 9.33) | 0.29 (-10.24, 12.06) |
| Yes | < 46.03 | -1.99 (-13.65, 11.26) | 2.83 (-8.42, 15.47) | 10.07 (-33.16, 81.27) | -1.47 (-10.22, 8.13) | 14.46 (-10.18, 45.88) |
| Yes | ≥ 46.03 | 18.16 (5.55, 32.28) | 17.67 (6.03, 30.58) | 18.82 (-24.75, 87.63) | 19.19 (9.68, 29.53) | 10.30 (-11.23, 37.06) |
| RERI |  | 0.16 (-0.01, 0.32) | 0.11 (-0.05, 0.26) | 0.09 (-0.61, 0.79) | 0..16 (0.04, 0.28) | -0.04 (-0.38, 0.29) |
| 7.5^th^ (2d) ^a^ |  |  |  |  |  |  |
| No | < 46.03 | 1.00 | 1.00 | 1.00 | 1.00 | 1.00 |
| No | ≥ 46.03 | 4.83 (-1.17, 11.20) | 3.77 (-1.76, 9.61) | 5.01 (-15.91, 31.14) | 4.63 (0.16, 9.31) | 1.31 (-9.56, 13.49) |
| Yes | < 46.03 | 4.50 (-6.87, 17.27) | 2.21 (-8.14, 13.73) | 30.59 (-15.39, 101.55) | 0.74 (-7.51, 9.72) | 14.50 (-8.13, 42.71) |
| Yes | ≥ 46.03 | 22.96 (10.50, 36.82) | 16.98 (5.95, 29.16) | 7.35 (-31.07, 67.16) | 22.39 (13.11, 32.42) | 4.79 (-14.79, 28.87) |
| RERI |  | 0.14 (-0.02, 0.29) | 0.11 (-0.03, 0.25) | -0.28 (-0.95, 0.38) | 0.17 (0.06, 0.28) | -0.11 (-0.40, 0.18) |
| 7.5^th^ (3d) ^a^ |  |  |  |  |  |  |
| No | < 46.03 | 1.00 | 1.00 | 1.00 | 1.00 | 1.00 |
| No | ≥ 46.03 | 5.28 (-0.63, 11.54) | 4.09 (-1.34, 9.83) | 3.82 (-16.55, 29.15) | 5.10 (0.70, 9.70) | 1.54 (-9.15, 13.49) |
| Yes | < 46.03 | 5.38 (-6.80, 19.14) | 4.24 (-6.95, 16.78) | 32.94 (-16.24, 110.98) | 1.59 (-7.24, 11.27) | 21.20 (-4.07, 53.14) |
| Yes | ≥ 46.03 | 19.78 (7.00, 34.08) | 18.34 (6.80, 31.13) | 12.84 (-29.07, 79.54) | 21.21 (11.64, 31.61) | 6.42 (-14.35, 32.23) |
| RERI |  | 0.09 (-0.08, 0.26) | 0.10 (-0.05, 0.25) | -0.24 (-0.97, 0.49) | 0.15 (0.02, 0.27) | -0.16 (-0.50, 0.17) |
| 7.5^th^ (4d) ^a^ |  |  |  |  |  |  |
| No | < 46.03 | 1.00 | 1.00 | 1.00 | 1.00 | 1.00 |
| No | ≥ 46.03 | 4.88 (-0.88, 10.98) | 4.08 (-1.25, 9.70) | 0.13 (-19.04, 23.83) | 5.02 (0.71, 9.52) | 1.50 (-9.00, 13.21) |
| Yes | < 46.03 | 0.23 (-13.47, 16.10) | 2.34 (-10.44, 16.96) | 10.71 (-36.96, 94.43) | -1.94 (-11.98, 9.23) | 24.54 (-5.25, 63.69) |
| Yes | ≥ 46.03 | 16.83 (2.89, 32.66) | 17.73 (4.91, 32.12) | 13.66 (-32.89, 92.51) | 19.96 (9.40, 31.55) | 1.81 (-20.80, 30.88) |
| RERI |  | 0.12 (-0.08, 0.31) | 0.11 (-0.07, 0.29) | 0.03 (-0.77, 0.83) | 0.17 (0.03, 0.31) | -0.24 (-0.64, 0.16) |
| 5^th^ (2d) ^a^ |  |  |  |  |  |  |
| No | < 46.03 | 1.00 | 1.00 | 1.00 | 1.00 | 1.00 |
| No | ≥ 46.03 | 3.85 (-1.96, 10.00) | 2.66 (-2.67, 8.27) | 2.51 (-17.41, 27.24) | 3.70 (-0.63, 8.20) | -0.01 (-10.49, 11.70) |
| Yes | < 46.03 | 1.60 (-10.41, 15.22) | -5.70 (-16.20, 6.10) | 23.55 (-23.61, 99.81) | -5.33 (-13.83, 4.00) | 12.63 (-11.33, 43.06) |
| Yes | ≥ 46.03 | 33.86 (18.35, 51.40) | 17.72 (5.19, 31.75) | 5.56 (-36.78, 76.26) | 27.60 (16.62, 39.60) | 9.69 (-13.68, 39.39) |
| RERI |  | 0.28 (0.09, 0.48) | 0.21 (0.05, 0.37) | -0.20 (-0.98, 0.57) | 0.29 (0.16, 0.43) | -0.03 (-0.39, 0.33) |
| 5^th^ (3d) ^a^ |  |  |  |  |  |  |
| No | < 46.03 | 1.00 | 1.00 | 1.00 | 1.00 | 1.00 |
| No | ≥ 46.03 | 4.48 (-1.22, 10.52) | 3.94 (-1.34, 9.50) | 1.53 (-17.78, 25.37) | 4.81 (0.54, 9.27) | 0.59 (-9.76, 12.12) |
| Yes | < 46.03 | 3.19 (-11.06, 19.73) | 4.72 (-8.56, 19.93) | 21.79 (-31.08, 115.24) | 0.01 (-10.37, 11.59) | 30.55 (-0.84, 71.86) |
| Yes | ≥ 46.03 | 29.47 (13.33, 47.90) | 22.32 (8.48, 37.91) | -0.96 (-42.97, 72.00) | 28.02 (16.22, 41.01) | 14.86 (-10.88, 48.03) |
| RERI |  | 0.22 (-0.01, 0.44) | 0.14 (-0.06, 0.33) | -0.24 (-1.11, 0.62) | 0.23 (0.07, 0.39) | -0.16 (-0.61, 0.28) |
| 5^th^ (4d) ^a^ |  |  |  |  |  |  |
| No | < 46.03 | 1.00 | 1.00 | 1.00 | 1.00 | 1.00 |
| No | ≥ 46.03 | 4.64 (-1.03, 10.65) | 3.36 (-1.86, 8.86) | 0.12 (-18.78, 23.42) | 4.58 (0.34, 9.00) | 0.54 (-9.75, 11.99) |
| Yes | < 46.03 | 1.08 (-14.37, 19.31) | -1.39 (-15.17, 14.63) | 1.26 (-46.43, 91.41) | -3.49 (-14.56, 9.02) | 23.12 (-9.39, 67.29) |
| Yes | ≥ 46.03 | 31.84 (12.95, 53.90) | 33.04 (15.99, 52.58) | -9.48 (-52.72, 73.30) | 38.29 (23.75, 54.53) | 7.65 (-19.90, 44.68) |
| RERI |  | 0.26 (0.01, 0.51) | 0.31 (0.09, 0.53) | -0.11 (-0.97, 0.75) | 0.37 (0.19, 0.56) | -0.16 (-0.64, 0.32) |
| Shenzhen |  |  |  |  |  |  |
| 10^th^ (2d) ^a^ |  |  |  |  |  |  |
| No | < 39.46 | 1.00 | 1.00 | 1.00 | 1.00 | 1.00 |
| No | ≥ 39.46 | 2.10 (-0.99, 5.29) | 1.41 (-1.43, 4.34) | -11.30 (-20.51, -1.03) | 2.27 (0.03, 4.57) | 2.06 (-5.33, 10.04) |
| Yes | < 39.46 | 5.30 (-0.12, 11.01) | 9.29 (4.03, 14.81) | -10.12 (-26.51, 9.91) | 8.03 (3.99, 12.23) | 8.15 (-5.22, 23.41) |
| Yes | ≥ 39.46 | 7.75 (1.78, 14.08) | 9.09 (3.40, 15.09) | -3.70 (-21.84, 18.64) | 8.67 (4.26, 13.27) | 12.56 (-2.31, 29.69) |
| RERI |  | 0.01 (-0.06, 0.07) | -0.02 (-0.08, 0.05) | 0.18 (-0.05, 0.40) | -0.02 (-0.07, 0.03) | 0.02 (-0.15, 0.20) |
| 10^th^ (3d) ^a^ |  |  |  |  |  |  |
| No | < 39.46 | 1.00 | 1.00 | 1.00 | 1.00 | 1.00 |
| No | ≥ 39.46 | 2.28 (-0.77, 5.43) | 1.99 (-0.83, 4.88) | -8.30 (-17.80, 2.29) | 2.58 (0.36, 4.84) | 1.72 (-5.54, 9.53) |
| Yes | < 39.46 | 3.84 (-2.03, 10.05) | 8.10 (2.40, 14.10) | -7.39 (-25.58, 15.24) | 6.89 (2.50, 11.47) | 2.82 (-11.07, 18.89) |
| Yes | ≥ 39.46 | 8.03 (1.86, 14.56) | 8.69 (2.86, 14.84) | -1.48 (-20.83, 22.59) | 8.46 (3.93, 13.18) | 12.16 (-2.93, 29.59) |
| RERI |  | 0.02 (-0.05, 0.09) | -0.01 (-0.08, 0.06) | 0.14 (-0.11, 0.40) | -0.01 (-0.06, 0.04) | 0.08 (-0.11, 0.26) |
| 10^th^ (4d) ^a^ |  |  |  |  |  |  |
| No | < 39.46 | 1.00 | 1.00 | 1.00 | 1.00 | 1.00 |
| No | ≥ 39.46 | 1.27 (-1.66, 4.30) | 0.67 (-2.03, 3.45) | -9.46 (-18.65, 0.76) | 1.28 (-0.85, 3.45) | 1.96 (-5.11, 9.55) |
| Yes | < 39.46 | -0.69 (-6.56, 5.54) | 4.52 (-1.20, 10.56) | -7.02 (-25.91, 16.69) | 2.33 (-2.05, 6.91) | 2.57 (-11.64, 19.07) |
| Yes | ≥ 39.46 | 6.41 (0.36, 12.84) | 9.21 (3.40, 15.35) | 2.83 (-17.35, 27.95) | 7.97 (3.49, 12.65) | 9.27 (-5.36, 26.17) |
| RERI |  | 0.06 (-0.02, 0.13) | 0.04 (-0.03, 0.11) | 0.19 (-0.08, 0.46) | 0.04 (-0.01, 0.10) | 0.05 (-0.14, 0.24) |
| 7.5^th^ (2d) ^a^ |  |  |  |  |  |  |
| No | < 39.46 | 1.00 | 1.00 | 1.00 | 1.00 | 1.00 |
| No | ≥ 39.46 | 1.58 (-1.41, 4.65) | 0.86 (-1.89, 3.68) | -8.90 (-18.10, 1.35) | 1.58 (-0.58, 3.79) | 1.65 (-5.48, 9.32) |
| Yes | < 39.46 | 3.04 (-3.00, 9.46) | 7.05 (1.22, 13.23) | -5.09 (-24.37, 19.11) | 5.63 (1.13, 10.33) | 3.80 (-10.65, 20.58) |
| Yes | ≥ 39.46 | 5.59 (-0.49, 12.05) | 5.47 (-0.24, 11.50) | -6.84 (-25.43, 16.38) | 6.10 (1.63, 10.76) | 4.86 (-9.51, 21.50) |
| RERI |  | 0.01 (-0.07, 0.09) | -0.02 (-0.10, 0.05) | 0.07 (-0.20, 0.34) | -0.01 (-0.07, 0.05) | -0.01 (-0.20, 0.19) |
| 7.5^th^ (3d) ^a^ |  |  |  |  |  |  |
| No | < 39.46 | 1.00 | 1.00 | 1.00 | 1.00 | 1.00 |
| No | ≥ 39.46 | 2.41 (-0.50, 5.41) | 1.61 (-1.07, 4.35) | -6.98 (-16.20, 3.27) | 2.21 (0.11, 4.36) | 3.40 (-3.63, 10.94) |
| Yes | < 39.46 | 5.15 (-1.63, 12.40) | 7.64 (1.18, 14.51) | -3.44 (-25.02, 24.35) | 6.41 (1.41, 11.64) | 11.17 (-5.56, 30.85) |
| Yes | ≥ 39.46 | 6.31 (0.13, 12.88) | 5.29 (-0.45, 11.36) | -1.08 (-20.86, 23.65) | 5.78 (1.28, 10.47) | 8.63 (-6.28, 25.90) |
| RERI |  | -0.01 (-0.10, 0.07) | -0.04 (-0.12, 0.04) | 0.09 (-0.20, 0.39) | -0.03 (-0.09, 0.03) | -0.06 (-0.27, 0.16) |
| 7.5^th^ (4d) ^a^ |  |  |  |  |  |  |
| No | < 39.46 | 1.00 | 1.00 | 1.00 | 1.00 | 1.00 |
| No | ≥ 39.46 | 1.40 (-1.42, 4.30) | 0.43 (-2.16, 3.08) | -7.76 (-16.70, 2.14) | 1.15 (-0.89, 3.23) | 1.76 (-5.01, 9.00) |
| Yes | < 39.46 | -1.30 (-8.91, 6.95) | 1.39 (-5.84, 9.18) | 2.94 (-23.76, 38.98) | 0.12 (-5.50, 6.07) | -0.97 (-18.46, 20.27) |
| Yes | ≥ 39.46 | 6.32 (-0.07, 13.13) | 5.70 (-0.30, 12.06) | 5.52 (-16.65, 33.58) | 5.96 (1.29, 10.84) | 6.58 (-8.71, 24.44) |
| RERI |  | 0.06 (-0.03, 0.16) | 0.04 (-0.05, 0.13) | 0.10 (-0.25, 0.46) | 0.05 (-0.02, 0.12) | 0.06 (-0.17, 0.29) |
| 5^th^ (2d) ^a^ |  |  |  |  |  |  |
| No | < 39.46 | 1.00 | 1.00 | 1.00 | 1.00 | 1.00 |
| No | ≥ 39.46 | 1.34 (-1.52, 4.27) | 0.19 (-2.42, 2.88) | -8.79 (-17.67, 1.06) | 1.22 (-0.85, 3.33) | -0.38 (-7.10, 6.83) |
| Yes | < 39.46 | 1.37 (-5.34, 8.56) | 2.99 (-3.36, 9.77) | -4.81 (-26.68, 23.60) | 3.53 (-1.45, 8.75) | -10.77 (-25.11, 6.33) |
| Yes | ≥ 39.46 | 4.12 (-2.31, 10.96) | 1.89 (-3.99, 8.13) | -3.28 (-23.69, 22.58) | 3.39 (-1.27, 8.27) | -0.05 (-14.37, 16.65) |
| RERI |  | 0.01 (-0.07, 0.10) | -0.01 (-0.09, 0.07) | 0.10 (-0.20, 0.41) | -0.01 (-0.08, 0.05) | 0.11 (-0.09, 0.31) |
| 5^th^ (3d) ^a^ |  |  |  |  |  |  |
| No | < 39.46 | 1.00 | 1.00 | 1.00 | 1.00 | 1.00 |
| No | ≥ 39.46 | 1.30 (-1.49, 4.17) | 0.25 (-2.31, 2.88) | -7.71 (-16.63, 2.16) | 1.04 (-0.98, 3.09) | 1.19 (-5.48, 8.33) |
| Yes | < 39.46 | -9.30 (-16.83, -1.09) | -5.92 (-13.17, 1.94) | -17.16 (-40.26, 14.89) | -6.49 (-12.13, -0.49) | -15.09 (-31.76, 5.65) |
| Yes | ≥ 39.46 | 0.27 (-6.12, 7.10) | 1.70 (-4.33, 8.10) | -2.85 (-24.07, 24.30) | 1.36 (-3.34, 6.29) | -1.42 (-15.94, 15.61) |
| RERI |  | 0.08 (-0.01, 0.18) | 0.07 (-0.01, 0.16) | 0.22 (-0.11, 0.55) | 0.07 (-0.01, 0.14) | 0.12 (-0.10, 0.35) |
| 5^th^ (4d) ^a^ |  |  |  |  |  |  |
| No | < 39.46 | 1.00 | 1.00 | 1.00 | 1.00 | 1.00 |
| No | ≥ 39.46 | 1.10 (-1.66, 3.93) | 0.31 (-2.23, 2.91) | -7.91 (-16.69, 1.79) | 0.97 (-1.02, 3.01) | 1.20 (-5.41, 8.28) |
| Yes | < 39.46 | -11.20 (-20.39, -0.96) | -3.81 (-13.12, 6.51) | -12.07 (-43.27, 36.30) | -6.43 (-13.51, 1.22) | -15.98 (-36.17, 10.59) |
| Yes | ≥ 39.46 | 4.51 (-2.86, 12.44) | 4.46 (-2.42, 11.82) | -0.76 (-25.09, 31.49) | 4.80 (-0.59, 10.48) | 2.62 (-14.06, 22.55) |
| RERI |  | 0.15 (0.03, 0.26) | 0.08 (-0.03, 0.19) | 0.19 (-0.24, 0.62) | 0.10 (0.02, 0.19) | 0.17 (-0.09, 0.44) |
| Zhaoqing |  |  |  |  |  |  |
| 10^th^ (2d) ^b^ |  |  |  |  |  |  |
| No | < 44.50 | 1.00 | 1.00 | 1.00 | 1.00 | 1.00 |
| No | ≥ 44.50 | 3.52 (-3.70, 11.28) | -2.02 (-8.60, 5.04) | 36.94 (0.02, 87.49) | -1.55 (-6.88, 4.09) | 7.05 (-5.38, 21.11) |
| Yes | < 44.50 | -11.53 (-23.98, 2.95) | -13.31 (-24.90, 0.05) | 4.05 (-47.30, 105.43) | -13.39 (-22.75, -2.90) | -9.40 (-31.00, 18.96) |
| Yes | ≥ 44.50 | 0.16 (-16.65, 20.35) | 13.05 (-5.23, 34.85) | -25.56 (-74.14, 114.27) | 6.95 (-6.88, 22.83) | 8.13 (-22.90, 51.65) |
| RERI |  | 0.08 (-0.13, 0.29) | 0.28 (0.07, 0.50) | -0.67 (-1.73, 0.40) | 0.22 (0.05, 0.38) | 0.10 (-0.30, 0.51) |
| 10^th^ (3d) ^b^ |  |  |  |  |  |  |
| No | < 44.50 | 1.00 | 1.00 | 1.00 | 1.00 | 1.00 |
| No | ≥ 44.50 | 2.61 (-4.40, 10.14) | -0.92 (-7.42, 6.04) | 41.78 (4.19, 92.93) | -1.67 (-6.88, 3.83) | 8.27 (-4.08, 22.22) |
| Yes | < 44.50 | -18.16 (-30.82, -3.19) | -9.26 (-22.33, 6.01) | 12.41 (-46.22, 134.96) | -15.61 (-25.58, -4.31) | -5.04 (-29.15, 27.27) |
| Yes | ≥ 44.50 | 1.50 (-15.98, 22.62) | 14.97 (-4.00, 37.68) | -38.49 (-80.46, 93.63) | 8.14 (-6.17, 24.64) | 14.93 (-18.63, 62.33) |
| RERI |  | 0.17 (-0.06, 0.40) | 0.25 (0.02, 0.49) | -0.93 (-2.07, 0.21) | 0.25 (0.08, 0.43) | 0.12 (-0.34, 0.57) |
| 10^th^ (4d) ^b^ |  |  |  |  |  |  |
| No | < 44.50 | 1.00 | 1.00 | 1.00 | 1.00 | 1.00 |
| No | ≥ 44.50 | 2.95 (-3.94, 10.34) | -0.25 (-6.67, 6.61) | 40.25 (3.69, 89.68) | -1.04 (-6.17, 4.37) | 8.62 (-3.58, 22.37) |
| Yes | < 44.50 | -11.57 (-26.96, 7.05) | -1.98 (-17.71, 16.76) | 31.66 (-42.25, 200.17) | -9.59 (-21.64, 4.30) | 7.14 (-22.39, 47.91) |
| Yes | ≥ 44.50 | 13.96 (-6.05, 38.24) | 21.49 (1.09, 45.99) | -20.97 (-75.47, 154.58) | 16.29 (0.58, 34.46) | 31.85 (-6.96, 86.86) |
| RERI |  | 0.23 (-0.04, 0.49) | 0.24 (-0.03, 0.50) | -0.93 (-2.38, 0.52) | 0.27 (0.07, 0.47) | 0.16 (-0.38, 0.70) |
| 7.5^th^ (2d) ^b^ |  |  |  |  |  |  |
| No | < 44.50 | 1.00 | 1.00 | 1.00 | 1.00 | 1.00 |
| No | ≥ 44.50 | 4.07 (-2.94, 11.59) | -0.92 (-7.33, 5.93) | 33.80 (-1.33, 81.44) | -0.48 (-5.68, 5.00) | 7.12 (-4.90, 20.66) |
| Yes | < 44.50 | -11.74 (-25.23, 4.20) | -10.43 (-23.45, 4.80) | -5.17 (-55.47, 101.95) | -11.29 (-21.75, 0.56) | -10.84 (-33.59, 19.69) |
| Yes | ≥ 44.50 | 2.30 (-16.79, 25.77) | 24.04 (2.60, 49.95) | -25.77 (-76.92, 138.73) | 13.87 (-2.25, 32.66) | 15.84 (-19.32, 66.31) |
| RERI |  | 0.10 (-0.15, 0.35) | 0.35 (0.10, 0.61) | -0.54 (-1.67, 0.58) | 0.26 (0.06, 0.45) | 0.20 (-0.28, 0.67) |
| 7.5^th^ (3d) ^b^ |  |  |  |  |  |  |
| No | < 44.50 | 1.00 | 1.00 | 1.00 | 1.00 | 1.00 |
| No | ≥ 44.50 | 3.73 (-3.08, 11.03) | -1.37 (-7.61, 5.28) | 41.15 (4.82, 90.08) | -0.96 (-6.01, 4.36) | 6.20 (-5.43, 19.26) |
| Yes | < 44.50 | -20.88 (-35.77, -2.54) | -22.35 (-36.21, -5.47) | 32.22 (-43.49, 209.37) | -21.33 (-32.73, -8.00) | -32.33 (-54.23, 0.06) |
| Yes | ≥ 44.50 | 5.47 (-15.16, 31.12) | 22.50 (0.56, 49.24) | -39.42 (-83.59, 123.65) | 13.75 (-3.10, 33.52) | 26.32 (-12.85, 83.10) |
| RERI |  | 0.23 (-0.05, 0.50) | 0.46 (0.18, 0.74) | -1.13 (-2.55, 0.29) | 0.36 (0.15, 0.58) | 0.52 (-0.01, 1.05) |
| 7.5^th^ (4d) ^b^ |  |  |  |  |  |  |
| No | < 44.50 | 1.00 | 1.00 | 1.00 | 1.00 | 1.00 |
| No | ≥ 44.50 | 3.70 (-3.05, 10.93) | 0.01 (-6.25, 6.69) | 37.20 (2.27, 84.06) | -0.09 (-5.13, 5.23) | 6.80 (-4.83, 19.85) |
| Yes | < 44.50 | -18.69 (-35.81, 2.99) | -8.21 (-26.09, 14.01) | 20.34 (-55.92, 228.59) | -11.33 (-25.51, 5.54) | -29.50 (-54.59, 9.44) |
| Yes | ≥ 44.50 | 5.94 (-16.40, 34.24) | 21.45 (-1.96, 50.44) | -30.03 (-81.45, 163.98) | 12.91 (-5.23, 34.51) | 26.86 (-14.39, 87.97) |
| RERI |  | 0.21 (-0.10, 0.52) | 0.30 (-0.02, 0.62) | -0.88 (-2.42, 0.67) | 0.24 (-0.01, 0.49) | 0.50 (-0.08, 1.07) |
| 5^th^ (2d) ^b^ |  |  |  |  |  |  |
| No | < 44.50 | 1.00 | 1.00 | 1.00 | 1.00 | 1.00 |
| No | ≥ 44.50 | 4.02 (-2.80, 11.32) | -0.57 (-6.85, 6.14) | 32.51 (-1.59, 78.41) | -0.01 (-5.10, 5.34) | 5.98 (-5.67, 19.07) |
| Yes | < 44.50 | -24.56 (-39.79, -5.47) | -15.98 (-31.66, 3.29) | -14.48 (-65.99, 115.09) | -19.90 (-32.31, -5.20) | -22.30 (-47.24, 14.44) |
| Yes | ≥ 44.50 | -1.33 (-22.85, 26.19) | 29.69 (4.89, 60.36) | -28.26 (-81.19, 173.57) | 11.06 (-7.01, 32.64) | 45.43 (-1.65, 115.05) |
| RERI |  | 0.19 (-0.10, 0.49) | 0.46 (0.14, 0.78) | -0.46 (-1.74, 0.82) | 0.31 (0.07, 0.55) | 0.62 (-0.01, 1.25) |
| 5^th^ (3d) ^b^ |  |  |  |  |  |  |
| No | < 44.50 | 1.00 | 1.00 | 1.00 | 1.00 | 1.00 |
| No | ≥ 44.50 | 4.47 (-2.28, 11.68) | 0.02 (-6.21, 6.67) | 34.91 (0.73, 80.68) | 0.23 (-4.79, 5.52) | 7.76 (-3.94, 20.88) |
| Yes | < 44.50 | -32.62 (-48.92, -11.13) | -14.40 (-32.91, 9.23) | -14.22 (-72.71, 169.65) | -23.62 (-37.59, -6.51) | -22.15 (-50.83, 23.25) |
| Yes | ≥ 44.50 | -6.64 (-28.51, 21.92) | 28.57 (2.46, 61.34) | -17.19 (-78.74, 222.46) | 9.03 (-9.97, 32.05) | 31.77 (-13.05, 99.70) |
| RERI |  | 0.22 (-0.10, 0.53) | 0.43 (0.07, 0.79) | -0.38 (-1.89, 1.13) | 0.32 (0.06, 0.58) | 0.46 (-0.19, 1.11) |
| 5^th^ (4d) ^b^ |  |  |  |  |  |  |
| No | < 44.50 | 1.00 | 1.00 | 1.00 | 1.00 | 1.00 |
| No | ≥ 44.50 | 4.34 (-2.39, 11.53) | 0.72 (-5.53, 7.40) | 35.90 (1.47, 82.00) | 0.53 (-4.49, 5.82) | 8.06 (-3.65, 21.20) |
| Yes | < 44.50 | -24.44 (-43.51, 1.07) | -3.82 (-25.38, 23.98) | 5.36 (-67.27, 239.22) | -13.99 (-30.36, 6.23) | -14.41 (-47.24, 38.83) |
| Yes | ≥ 44.50 | -10.41 (-33.18, 20.13) | 16.85 (-9.31, 50.55) | -44.95 (-88.75, 169.29) | 1.29 (-18.22, 25.46) | 25.30 (-19.88, 95.97) |
| RERI |  | 0.10 (-0.25, 0.44) | 0.20 (-0.18, 0.58) | -0.86 (-2.42, 0.70) | 0.15 (-0.13, 0.43) | 0.32 (-0.37, 1.00) |

Note: PM, particulate matter.

^a^ Cold spells were defined by percentile temperature thresholds (10^th^, 7.5^th^, 5^th^ and 2.5^th^) and by the number of consecutive days below the thresholds (2-4 d).

^b^ Nine definitions [10^th^ (2d), 10^th^ (3d), 10^th^ (4d), 7.5^th^ (2d), 7.5^th^ (3d), 7.5^th^ (4d), 5^th^ (2d), 5^th^ (3d), and 5^th^ (4d)] were used in Zhaoqing.

^c^ Air pollutants were classified as binary variables using the median of air pollutant (lag03) concentrations as a cut off.

## Table S13. Percent changes and relative excess risk due to interaction (RERI) of cold spells and PM_10_ exposure on anxiety stratified by sex and age in three subtropical cities in China.

| Cold spells | PM_10_ ^c^ (µg/m^3^) | male | female | < 18 | 18-65 | ≥ 65 |
| --- | --- | --- | --- | --- | --- | --- |
| Huizhou |  |  |  |  |  |  |
| 10^th^ (2d) ^a^ |  |  |  |  |  |  |
| No | < 64.71 | 1.00 | 1.00 | 1.00 | 1.00 | 1.00 |
| No | ≥ 64.71 | 4.13 (-1.57, 10.15) | 2.98 (-2.28, 8.53) | -5.15 (-23.07, 16.93) | 4.39 (0.11, 8.85) | -0.01 (-10.27, 11.42) |
| Yes | < 64.71 | -2.52 (-12.17, 8.20) | -1.14 (-10.25, 8.89) | 5.21 (-28.37, 54.54) | -3.58 (-10.76, 4.18) | 8.68 (-11.01, 32.73) |
| Yes | ≥ 64.71 | 29.88 (17.50, 43.56) | 21.62 (10.82, 33.46) | 12.23 (-24.57, 66.98) | 28.06 (18.91, 37.93) | 12.51 (-7.09, 36.24) |
| RERI |  | 0.28 (0.14, 0.42) | 0.20 (0.07, 0.33) | 0.12 (-0.40, 0.64) | 0.27 (0.17, 0.38) | 0.04 (-0.23, 0.31) |
| 10^th^ (3d) ^a^ |  |  |  |  |  |  |
| No | < 64.71 | 1.00 | 1.00 | 1.00 | 1.00 | 1.00 |
| No | ≥ 64.71 | 3.62 (-1.90, 9.46) | 3.05 (-2.09, 8.46) | -6.06 (-23.56, 15.44) | 4.24 (0.08, 8.58) | -0.29 (-10.28, 10.82) |
| Yes | < 64.71 | -8.07 (-18.07, 3.14) | -2.35 (-12.06, 8.44) | 0.73 (-34.60, 55.15) | -6.91 (-14.45, 1.30) | 6.78 (-14.14, 32.81) |
| Yes | ≥ 64.71 | 26.43 (14.13, 40.06) | 20.96 (10.00, 33.01) | 12.70 (-26.03, 71.72) | 26.31 (17.11, 36.24) | 8.98 (-10.48, 32.67) |
| RERI |  | 0.31 (0.16, 0.45) | 0.20 (0.07, 0.34) | 0.18 (-0.39, 0.75) | 0.29 (0.18, 0.40) | 0.02 (-0.26, 0.31) |
| 10^th^ (4d) ^a^ |  |  |  |  |  |  |
| No | < 64.71 | 1.00 | 1.00 | 1.00 | 1.00 | 1.00 |
| No | ≥ 64.71 | 4.19 (-1.19, 9.87) | 3.12 (-1.88, 8.36) | -8.89 (-25.32, 11.16) | 4.65 (0.60, 8.87) | 0.28 (-9.48, 11.09) |
| Yes | < 64.71 | -11.15 (-22.27, 1.55) | -4.20 (-15.05, 8.04) | -12.01 (-47.28, 46.83) | -9.94 (-18.30, -0.73) | 14.86 (-10.67, 47.69) |
| Yes | ≥ 64.71 | 24.26 (11.50, 38.47) | 21.39 (9.78, 34.24) | 24.01 (-20.12, 92.51) | 24.99 (15.36, 35.42) | 10.31 (-10.37, 35.76) |
| RERI |  | 0.31 (0.15, 0.48) | 0.22 (0.07, 0.38) | 0.45 (-0.19, 1.08) | 0.30 (0.18, 0.42) | -0.05 (-0.39, 0.29) |
| 7.5^th^ (2d) ^a^ |  |  |  |  |  |  |
| No | < 64.71 | 1.00 | 1.00 | 1.00 | 1.00 | 1.00 |
| No | ≥ 64.71 | 4.79 (-0.76, 10.64) | 3.26 (-1.84, 8.62) | -5.63 (-23.01, 15.67) | 4.70 (0.55, 9.02) | 1.77 (-8.32, 12.99) |
| Yes | < 64.71 | -2.72 (-13.48, 9.38) | -2.50 (-12.49, 8.64) | 3.10 (-33.56, 59.98) | -5.39 (-13.27, 3.20) | 16.15 (-6.86, 44.85) |
| Yes | ≥ 64.71 | 29.74 (16.81, 44.09) | 20.52 (9.31, 32.88) | 14.20 (-25.56, 75.20) | 28.55 (18.93, 38.93) | 4.32 (-14.77, 27.68) |
| RERI |  | 0.28 (0.12, 0.43) | 0.20 (0.06, 0.34) | 0.17 (-0.42, 0.76) | 0.29 (0.18, 0.40) | -0.14 (-0.44, 0.16) |
| 7.5^th^ (3d) ^a^ |  |  |  |  |  |  |
| No | < 64.71 | 1.00 | 1.00 | 1.00 | 1.00 | 1.00 |
| No | ≥ 64.71 | 5.09 (-0.36, 10.85) | 3.63 (-1.39, 8.91) | -6.32 (-23.36, 14.50) | 5.06 (0.98, 9.30) | 2.24 (-7.71, 13.25) |
| Yes | < 64.71 | -4.37 (-15.90, 8.75) | -1.25 (-12.14, 10.99) | 2.32 (-36.46, 64.78) | -6.50 (-14.94, 2.77) | 26.30 (-0.69, 60.62) |
| Yes | ≥ 64.71 | 26.90 (13.81, 41.49) | 21.71 (10.13, 34.49) | 23.63 (-20.96, 93.39) | 27.38 (17.60, 37.98) | 5.17 (-14.66, 29.62) |
| RERI |  | 0.26 (0.10, 0.43) | 0.19 (0.04, 0.34) | 0.28 (-0.38, 0.94) | 0.29 (0.17, 0.41) | -0.23 (-0.58, 0.12) |
| 7.5^th^ (4d) ^a^ |  |  |  |  |  |  |
| No | < 64.71 | 1.00 | 1.00 | 1.00 | 1.00 | 1.00 |
| No | ≥ 64.71 | 5.10 (-0.23, 10.72) | 3.24 (-1.67, 8.38) | -8.99 (-25.12, 10.61) | 5.07 (1.07, 9.22) | 1.48 (-8.21, 12.19) |
| Yes | < 64.71 | -11.48 (-24.54, 3.83) | -7.39 (-19.64, 6.72) | -22.57 (-58.78, 45.44) | -13.01 (-22.51, -2.35) | 28.93 (-4.00, 73.15) |
| Yes | ≥ 64.71 | 23.77 (9.66, 39.71) | 22.55 (9.69, 36.92) | 25.78 (-22.56, 104.28) | 26.78 (16.03, 38.54) | 1.94 (-19.53, 29.13) |
| RERI |  | 0.30 (0.11, 0.49) | 0.27 (0.09, 0.44) | 0.57 (-0.15, 1.30) | 0.35 (0.21, 0.49) | -0.28 (-0.72, 0.15) |
| 5^th^ (2d) ^a^ |  |  |  |  |  |  |
| No | < 64.71 | 1.00 | 1.00 | 1.00 | 1.00 | 1.00 |
| No | ≥ 64.71 | 3.99 (-1.39, 9.66) | 2.59 (-2.35, 7.78) | -7.41 (-24.08, 12.92) | 4.07 (0.04, 8.25) | 0.73 (-9.04, 11.56) |
| Yes | < 64.71 | -7.75 (-18.91, 4.93) | -10.01 (-20.14, 1.39) | -8.94 (-44.41, 49.17) | -12.28 (-20.30, -3.46) | 15.39 (-9.24, 46.72) |
| Yes | ≥ 64.71 | 46.08 (29.47, 64.83) | 22.57 (9.68, 36.99) | 26.52 (-22.75, 107.19) | 37.05 (25.42, 49.76) | 8.39 (-14.29, 37.06) |
| RERI |  | 0.50 (0.30, 0.70) | 0.30 (0.14, 0.46) | 0.43 (-0.30, 1.16) | 0.45 (0.32, 0.59) | -0.08 (-0.43, 0.28) |
| 5^th^ (3d) ^a^ |  |  |  |  |  |  |
| No | < 64.71 | 1.00 | 1.00 | 1.00 | 1.00 | 1.00 |
| No | ≥ 64.71 | 4.67 (-0.62, 10.24) | 3.81 (-1.09, 8.95) | -7.66 (-23.93, 12.09) | 5.21 (1.23, 9.35) | 0.89 (-8.71, 11.51) |
| Yes | < 64.71 | -10.00 (-22.99, 5.19) | -1.59 (-14.36, 13.07) | -21.26 (-57.85, 47.09) | -9.64 (-19.35, 1.23) | 33.03 (0.60, 75.90) |
| Yes | ≥ 64.71 | 41.78 (24.57, 61.36) | 27.39 (13.24, 43.30) | 24.14 (-26.00, 108.27) | 37.43 (25.02, 51.08) | 14.27 (-10.64, 46.11) |
| RERI |  | 0.47 (0.25, 0.69) | 0.25 (0.06, 0.45) | 0.53 (-0.25, 1.31) | 0.42 (0.26, 0.58) | -0.20 (-0.65, 0.25) |
| 5^th^ (4d) ^a^ |  |  |  |  |  |  |
| No | < 64.71 | 1.00 | 1.00 | 1.00 | 1.00 | 1.00 |
| No | ≥ 64.71 | 4.90 (-0.36, 10.44) | 3.25 (-1.59, 8.33) | -7.58 (-23.74, 12.00) | 5.07 (1.12, 9.17) | 0.43 (-9.07, 10.92) |
| Yes | < 64.71 | -13.64 (-27.99, 3.57) | -7.89 (-21.63, 8.27) | -27.58 (-64.41, 47.35) | -14.26 (-24.88, -2.14) | 25.42 (-9.72, 74.23) |
| Yes | ≥ 64.71 | 42.96 (23.54, 65.44) | 35.23 (18.67, 54.10) | 7.30 (-40.53, 93.57) | 45.40 (30.82, 61.62) | 7.75 (-18.38, 42.25) |
| RERI |  | 0.52 (0.27, 0.77) | 0.40 (0.18, 0.62) | 0.42 (-0.37, 1.22) | 0.55 (0.36, 0.73) | -0.18 (-0.68, 0.32) |
| Shenzhen |  |  |  |  |  |  |
| 10^th^ (2d) ^a^ |  |  |  |  |  |  |
| No | < 58.15 | 1.00 | 1.00 | 1.00 | 1.00 | 1.00 |
| No | ≥ 58.15 | 2.65 (-0.53, 5.94) | 0.89 (-2.01, 3.87) | -12.25 (-21.49, -1.93) | 2.23 (-0.06, 4.59) | 2.30 (-5.32, 10.54) |
| Yes | < 58.15 | 4.69 (-0.72, 10.40) | 6.70 (1.52, 12.14) | -9.38 (-25.73, 10.57) | 6.50 (2.49, 10.67) | 3.93 (-9.07, 18.78) |
| Yes | ≥ 58.15 | 10.33 (3.98, 17.06) | 12.11 (6.06, 18.50) | -7.51 (-25.72, 15.15) | 11.29 (6.61, 16.17) | 20.51 (4.09, 39.53) |
| RERI |  | 0.03 (-0.04, 0.10) | 0.05 (-0.02, 0.11) | 0.14 (-0.08, 0.37) | 0.03 (-0.03, 0.08) | 0.14 (-0.04, 0.32) |
| 10^th^ (3d) ^a^ |  |  |  |  |  |  |
| No | < 58.15 | 1.00 | 1.00 | 1.00 | 1.00 | 1.00 |
| No | ≥ 58.15 | 2.80 (-0.35, 6.05) | 1.19 (-1.68, 4.15) | -9.67 (-19.16, 0.94) | 2.53 (0.25, 4.85) | 0.34 (-7.04, 8.30) |
| Yes | < 58.15 | 3.10 (-2.72, 9.26) | 4.75 (-0.78, 10.59) | -6.33 (-24.36, 16.00) | 5.03 (0.72, 9.54) | -3.57 (-16.72, 11.66) |
| Yes | ≥ 58.15 | 10.80 (4.21, 17.81) | 11.79 (5.56, 18.38) | -6.00 (-25.39, 18.43) | 11.31 (6.47, 16.36) | 19.16 (2.54, 38.46) |
| RERI |  | 0.05 (-0.03, 0.12) | 0.06 (-0.01, 0.13) | 0.10 (-0.15, 0.35) | 0.04 (-0.02, 0.09) | 0.22 (0.04, 0.41) |
| 10^th^ (4d) ^a^ |  |  |  |  |  |  |
| No | < 58.15 | 1.00 | 1.00 | 1.00 | 1.00 | 1.00 |
| No | ≥ 58.15 | 1.50 (-1.52, 4.61) | 0.19 (-2.58, 3.03) | -10.69 (-19.92, -0.39) | 1.23 (-0.95, 3.47) | 0.73 (-6.47, 8.48) |
| Yes | < 58.15 | -2.00 (-7.76, 4.12) | 1.85 (-3.72, 7.74) | -5.88 (-24.46, 17.28) | 0.61 (-3.69, 5.10) | -4.39 (-17.77, 11.16) |
| Yes | ≥ 58.15 | 9.25 (2.76, 16.16) | 12.04 (5.84, 18.61) | -0.78 (-21.33, 25.15) | 10.64 (5.85, 15.64) | 16.43 (0.26, 35.20) |
| RERI |  | 0.10 (0.02, 0.18) | 0.10 (0.03, 0.17) | 0.16 (-0.11, 0.43) | 0.09 (0.03, 0.15) | 0.20 (0.01, 0.40) |
| 7.5^th^ (2d) ^a^ |  |  |  |  |  |  |
| No | < 58.15 | 1.00 | 1.00 | 1.00 | 1.00 | 1.00 |
| No | ≥ 58.15 | 1.70 (-1.36, 4.86) | 0.01 (-2.77, 2.88) | -9.86 (-19.11, 0.44) | 1.18 (-1.03, 3.43) | 1.19 (-6.10, 9.04) |
| Yes | < 58.15 | 0.95 (-4.97, 7.24) | 2.64 (-2.98, 8.58) | -4.10 (-23.17, 19.70) | 2.49 (-1.88, 7.06) | -3.90 (-17.42, 11.83) |
| Yes | ≥ 58.15 | 8.81 (2.28, 15.76) | 9.14 (3.03, 15.63) | -11.22 (-29.89, 12.42) | 9.36 (4.58, 14.36) | 13.76 (-2.30, 32.46) |
| RERI |  | 0.06 (-0.02, 0.14) | 0.06 (-0.01, 0.14) | 0.03 (-0.24, 0.30) | 0.06 (-0.01, 0.12) | 0.16 (-0.03, 0.36) |
| 7.5^th^ (3d) ^a^ |  |  |  |  |  |  |
| No | < 58.15 | 1.00 | 1.00 | 1.00 | 1.00 | 1.00 |
| No | ≥ 58.15 | 2.63 (-0.35, 5.69) | 0.67 (-2.03, 3.45) | -8.31 (-17.50, 1.90) | 1.91 (-0.23, 4.09) | 1.95 (-5.13, 9.57) |
| Yes | < 58.15 | 2.49 (-4.02, 9.44) | 2.11 (-3.96, 8.56) | -1.73 (-22.82, 25.11) | 2.59 (-2.17, 7.57) | -0.08 (-15.14, 17.64) |
| Yes | ≥ 58.15 | 9.55 (2.92, 16.61) | 8.94 (2.79, 15.46) | -5.37 (-25.27, 19.85) | 9.08 (4.27, 14.11) | 17.23 (0.65, 36.54) |
| RERI |  | 0.04 (-0.04, 0.13) | 0.06 (-0.02, 0.14) | 0.05 (-0.25, 0.34) | 0.05 (-0.02, 0.11) | 0.15 (-0.06, 0.37) |
| 7.5^th^ (4d) ^a^ |  |  |  |  |  |  |
| No | < 58.15 | 1.00 | 1.00 | 1.00 | 1.00 | 1.00 |
| No | ≥ 58.15 | 1.50 (-1.38, 4.47) | 0.01 (-2.62, 2.71) | -8.86 (-17.85, 1.10) | 1.05 (-1.02, 3.18) | 0.73 (-6.13, 8.10) |
| Yes | < 58.15 | -4.28 (-11.33, 3.32) | -2.23 (-8.92, 4.94) | 4.70 (-20.45, 37.82) | -2.82 (-8.03, 2.69) | -11.00 (-26.36, 7.56) |
| Yes | ≥ 58.15 | 9.98 (3.01, 17.41) | 8.58 (2.11, 15.47) | 2.09 (-20.77, 31.54) | 9.00 (3.95, 14.29) | 14.92 (-2.27, 35.14) |
| RERI |  | 0.13 (0.03, 0.22) | 0.11 (0.02, 0.20) | 0.06 (-0.29, 0.41) | 0.11 (0.04, 0.18) | 0.25 (0.02, 0.48) |
| 5^th^ (2d) ^a^ |  |  |  |  |  |  |
| No | < 58.15 | 1.00 | 1.00 | 1.00 | 1.00 | 1.00 |
| No | ≥ 58.15 | 2.05 (-0.91, 5.10) | -0.22 (-2.90, 2.54) | -9.83 (-18.78, 0.10) | 1.27 (-0.86, 3.44) | 0.55 (-6.45, 8.06) |
| Yes | < 58.15 | 1.04 (-5.42, 7.95) | -0.68 (-6.65, 5.66) | -3.61 (-24.62, 23.26) | 1.28 (-3.44, 6.24) | -12.87 (-26.45, 3.20) |
| Yes | ≥ 58.15 | 6.54 (-0.50, 14.08) | 5.28 (-1.18, 12.17) | -7.87 (-28.89, 19.36) | 6.33 (1.21, 11.70) | 5.87 (-10.23, 24.86) |
| RERI |  | 0.03 (-0.05, 0.12) | 0.06 (-0.01, 0.14) | 0.06 (-0.25, 0.36) | 0.04 (-0.03, 0.10) | 0.18 (-0.02, 0.39) |
| 5^th^ (3d) ^a^ |  |  |  |  |  |  |
| No | < 58.15 | 1.00 | 1.00 | 1.00 | 1.00 | 1.00 |
| No | ≥ 58.15 | 1.67 (-1.19, 4.62) | -0.13 (-2.74, 2.55) | -9.37 (-18.23, 0.45) | 1.09 (-0.98, 3.19) | 0.65 (-6.15, 7.95) |
| Yes | < 58.15 | -8.96 (-15.97, -1.37) | -7.71 (-14.36, -0.54) | -13.09 (-35.22, 16.60) | -7.17 (-12.39, -1.64) | -19.20 (-34.07, -0.98) |
| Yes | ≥ 58.15 | 2.50 (-4.51, 10.01) | 3.97 (-2.59, 10.98) | -7.03 (-28.94, 21.63) | 3.55 (-1.58, 8.96) | 4.02 (-12.25, 23.30) |
| RERI |  | 0.10 (0.01, 0.19) | 0.12 (0.03, 0.21) | 0.15 (-0.17, 0.48) | 0.10 (0.03, 0.16) | 0.23 (0.01, 0.45) |
| 5^th^ (4d) ^a^ |  |  |  |  |  |  |
| No | < 58.15 | 1.00 | 1.00 | 1.00 | 1.00 | 1.00 |
| No | ≥ 58.15 | 1.39 (-1.43, 4.30) | 0.05 (-2.53, 2.70) | -9.59 (-18.33, 0.09) | 1.02 (-1.02, 3.09) | 1.25 (-5.52, 8.51) |
| Yes | < 58.15 | -12.24 (-20.76, -2.81) | -6.80 (-15.21, 2.45) | -10.29 (-39.62, 33.31) | -8.44 (-14.91, -1.47) | -19.07 (-37.16, 4.23) |
| Yes | ≥ 58.15 | 7.55 (-0.37, 16.11) | 7.01 (-0.38, 14.94) | -3.00 (-27.89, 30.50) | 7.56 (1.76, 13.68) | 7.57 (-10.75, 29.64) |
| RERI |  | 0.18 (0.07, 0.30) | 0.14 (0.03, 0.24) | 0.17 (-0.25, 0.59) | 0.15 (0.07, 0.23) | 0.25 (-0.01, 0.52) |
| Zhaoqing |  |  |  |  |  |  |
| 10^th^ (2d) ^b^ |  |  |  |  |  |  |
| No | < 59.50 | 1.00 | 1.00 | 1.00 | 1.00 | 1.00 |
| No | ≥ 59.50 | 3.67 (-3.64, 11.54) | -2.26 (-8.87, 4.83) | 36.49 (-1.27, 88.69) | -1.95 (-7.31, 3.73) | 8.69 (-4.02, 23.08) |
| Yes | < 59.50 | -7.52 (-20.60, 7.72) | -17.17 (-28.44, -4.12) | -4.16 (-52.31, 92.62) | -13.68 (-23.13, -3.06) | -9.31 (-30.96, 19.14) |
| Yes | ≥ 59.50 | -6.71 (-23.43, 13.67) | 25.34 (4.04, 50.99) | 1.28 (-64.19, 186.42) | 7.85 (-6.88, 24.90) | 15.97 (-19.48, 67.03) |
| RERI |  | -0.03 (-0.25, 0.20) | 0.45 (0.20, 0.69) | -0.31 (-1.52, 0.90) | 0.23 (0.06, 0.41) | 0.17 (-0.30, 0.63) |
| 10^th^ (3d) ^b^ |  |  |  |  |  |  |
| No | < 59.50 | 1.00 | 1.00 | 1.00 | 1.00 | 1.00 |
| No | ≥ 59.50 | 1.93 (-5.07, 9.43) | -0.51 (-7.04, 6.48) | 36.70 (-0.20, 87.26) | -1.92 (-7.12, 3.58) | 9.53 (-3.00, 23.67) |
| Yes | < 59.50 | -15.24 (-28.13, -0.05) | -12.96 (-25.49, 1.69) | -5.34 (-55.31, 100.49) | -15.92 (-25.78, -4.75) | -5.23 (-28.88, 26.30) |
| Yes | ≥ 59.50 | -2.88 (-20.91, 19.25) | 28.46 (5.96, 55.75) | -6.88 (-69.05, 180.22) | 11.11 (-4.58, 29.39) | 24.02 (-14.94, 80.81) |
| RERI |  | 0.10 (-0.13, 0.34) | 0.42 (0.15, 0.69) | -0.38 (-1.60, 0.84) | 0.29 (0.10, 0.48) | 0.20 (-0.32, 0.71) |
| 10^th^ (4d) ^b^ |  |  |  |  |  |  |
| No | < 59.50 | 1.00 | 1.00 | 1.00 | 1.00 | 1.00 |
| No | ≥ 59.50 | 2.59 (-4.29, 9.96) | 0.17 (-6.27, 7.06) | 36.53 (0.32, 85.80) | -1.15 (-6.27, 4.26) | 9.97 (-2.41, 23.92) |
| Yes | < 59.50 | -6.95 (-22.66, 11.95) | -8.17 (-22.77, 9.19) | 7.57 (-53.83, 150.59) | -10.34 (-22.06, 3.14) | 5.89 (-22.60, 44.87) |
| Yes | ≥ 59.50 | 9.80 (-11.05, 35.54) | 38.07 (13.43, 68.07) | 20.26 (-61.26, 273.39) | 20.99 (3.48, 41.46) | 44.64 (-1.27, 111.89) |
| RERI |  | 0.14 (-0.14, 0.42) | 0.46 (0.16, 0.76) | -0.24 (-1.85, 1.38) | 0.32 (0.11, 0.54) | 0.29 (-0.33, 0.90) |
| 7.5^th^ (2d) ^b^ |  |  |  |  |  |  |
| No | < 59.50 | 1.00 | 1.00 | 1.00 | 1.00 | 1.00 |
| No | ≥ 59.50 | 3.98 (-3.08, 11.56) | -0.30 (-6.78, 6.62) | 36.37 (-0.20, 86.35) | -0.63 (-5.84, 4.88) | 9.45 (-2.90, 23.36) |
| Yes | < 59.50 | -6.94 (-20.84, 9.41) | -12.54 (-25.15, 2.20) | -7.45 (-56.23, 95.67) | -10.49 (-20.89, 1.27) | -7.93 (-30.95, 22.77) |
| Yes | ≥ 59.50 | -6.42 (-25.71, 17.87) | 38.05 (12.56, 69.33) | -14.48 (-74.68, 188.85) | 15.69 (-2.02, 36.60) | 20.53 (-19.30, 80.03) |
| RERI |  | -0.03 (-0.29, 0.22) | 0.51 (0.21, 0.81) | -0.43 (-1.67, 0.80) | 0.27 (0.06, 0.48) | 0.19 (-0.34, 0.72) |
| 7.5^th^ (3d) ^b^ |  |  |  |  |  |  |
| No | < 59.50 | 1.00 | 1.00 | 1.00 | 1.00 | 1.00 |
| No | ≥ 59.50 | 3.54 (-3.28, 10.84) | -0.48 (-6.76, 6.23) | 41.14 (4.19, 91.21) | -0.81 (-5.86, 4.52) | 8.00 (-3.85, 21.31) |
| Yes | < 59.50 | -12.00 (-27.41, 6.69) | -23.64 (-36.74, -7.82) | 20.67 (-47.36, 176.62) | -18.18 (-29.40, -5.17) | -25.83 (-48.20, 6.21) |
| Yes | ≥ 59.50 | -5.71 (-26.36, 20.74) | 38.20 (11.47, 71.33) | -30.83 (-82.15, 168.00) | 15.38 (-3.31, 37.68) | 32.52 (-12.73, 101.24) |
| RERI |  | 0.03 (-0.26, 0.31) | 0.62 (0.30, 0.94) | -0.93 (-2.31, 0.46) | 0.34 (0.11, 0.58) | 0.50 (-0.10, 1.11) |
| 7.5^th^ (4d) ^b^ |  |  |  |  |  |  |
| No | < 59.50 | 1.00 | 1.00 | 1.00 | 1.00 | 1.00 |
| No | ≥ 59.50 | 3.17 (-3.58, 10.38) | 0.77 (-5.55, 7.50) | 37.33 (1.72, 85.41) | -0.14 (-5.19, 5.18) | 8.11 (-3.69, 21.36) |
| Yes | < 59.50 | -10.77 (-28.15, 10.82) | -12.91 (-29.21, 7.13) | 7.93 (-60.06, 191.63) | -10.32 (-23.87, 5.63) | -24.73 (-49.57, 12.33) |
| Yes | ≥ 59.50 | -2.80 (-25.78, 27.29) | 38.99 (10.14, 75.41) | -18.02 (-79.64, 230.15) | 16.66 (-3.86, 41.55) | 37.14 (-11.79, 113.22) |
| RERI |  | 0.05 (-0.28, 0.37) | 0.51 (0.15, 0.88) | -0.63 (-2.23, 0.97) | 0.27 (0.01, 0.54) | 0.54 (-0.13, 1.21) |
| 5^th^ (2d) ^b^ |  |  |  |  |  |  |
| No | < 59.50 | 1.00 | 1.00 | 1.00 | 1.00 | 1.00 |
| No | ≥ 59.50 | 3.32 (-3.46, 10.56) | 0.30 (-6.02, 7.05) | 34.23 (-0.94, 81.90) | -0.33 (-5.38, 5.00) | 8.27 (-3.64, 21.67) |
| Yes | < 59.50 | -20.07 (-35.49, -0.96) | -18.12 (-32.95, -0.00) | -20.41 (-68.15, 98.87) | -19.22 (-31.28, -5.04) | -18.70 (-43.76, 17.52) |
| Yes | ≥ 59.50 | -6.85 (-29.11, 22.40) | 45.42 (15.70, 82.76) | -8.29 (-76.80, 262.55) | 14.84 (-5.33, 39.30) | 57.54 (2.55, 142.03) |
| RERI |  | 0.10 (-0.21, 0.41) | 0.63 (0.27, 1.00) | -0.22 (-1.69, 1.24) | 0.34 (0.09, 0.60) | 0.68 (-0.05, 1.41) |
| 5^th^ (3d) ^b^ |  |  |  |  |  |  |
| No | < 59.50 | 1.00 | 1.00 | 1.00 | 1.00 | 1.00 |
| No | ≥ 59.50 | 3.39 (-3.30, 10.54) | 1.05 (-5.24, 7.75) | 34.50 (-0.16, 81.20) | 0.03 (-4.98, 5.31) | 9.20 (-2.67, 22.52) |
| Yes | < 59.50 | -26.62 (-43.09, -5.40) | -17.57 (-34.51, 3.76) | -25.42 (-76.04, 132.14) | -22.25 (-35.62, -6.10) | -19.37 (-47.24, 23.22) |
| Yes | ≥ 59.50 | -12.60 (-34.94, 17.42) | 46.42 (14.68, 86.95) | 7.03 (-73.67, 335.09) | 13.19 (-8.05, 39.33) | 43.04 (-9.25, 125.45) |
| RERI |  | 0.11 (-0.21, 0.43) | 0.63 (0.23, 1.03) | -0.02 (-1.74, 1.70) | 0.35 (0.08, 0.63) | 0.53 (-0.20, 1.26) |
| 5^th^ (4d) ^b^ |  |  |  |  |  |  |
| No | < 59.50 | 1.00 | 1.00 | 1.00 | 1.00 | 1.00 |
| No | ≥ 59.50 | 3.04 (-3.62, 10.16) | 1.58 (-4.73, 8.31) | 35.77 (0.74, 82.97) | 0.24 (-4.78, 5.52) | 8.80 (-3.02, 22.05) |
| Yes | < 59.50 | -23.51 (-41.87, 0.65) | -9.97 (-29.40, 14.81) | -11.20 (-71.95, 181.08) | -15.69 (-31.01, 3.03) | -20.37 (-49.97, 26.74) |
| Yes | ≥ 59.50 | -11.03 (-35.06, 21.88) | 32.68 (1.46, 73.49) | -29.38 (-86.08, 258.25) | 6.31 (-15.39, 33.58) | 44.34 (-9.90, 131.23) |
| RERI |  | 0.09 (-0.26, 0.45) | 0.41 (-0.01, 0.83) | -0.54 (-2.11, 1.03) | 0.22 (-0.08, 0.51) | 0.56 (-0.21, 1.33) |

Note: PM, particulate matter.

^a^ Cold spells were defined by percentile temperature thresholds (10^th^, 7.5^th^, 5^th^ and 2.5^th^) and by the number of consecutive days below the thresholds (2-4 d).

^b^ Nine definitions [10^th^ (2d), 10^th^ (3d), 10^th^ (4d), 7.5^th^ (2d), 7.5^th^ (3d), 7.5^th^ (4d), 5^th^ (2d), 5^th^ (3d), and 5^th^ (4d)] were used in Zhaoqing.

^c^ Air pollutants were classified as binary variables using the median of air pollutant (lag03) concentrations as a cut off.

## Table S14. Percent changes and relative excess risk due to interaction (RERI) of cold spells and NO_2_ exposure on anxiety stratified by sex and age in three subtropical cities in China.

| Cold spells | NO_2_ ^c^ (µg/m^3^) | male | female | < 18 | 18-65 | ≥ 65 |
| --- | --- | --- | --- | --- | --- | --- |
| Huizhou |  |  |  |  |  |  |
| 10^th^ (2d) ^a^ |  |  |  |  |  |  |
| No | < 16.87 | 1.00 | 1.00 | 1.00 | 1.00 | 1.00 |
| No | ≥ 16.87 | 13.94 (5.81, 22.69) | 14.86 (7.11, 23.17) | 17.04 (-11.85, 55.40) | 13.55 (7.44, 20.02) | 18.63 (2.82, 36.89) |
| Yes | < 16.87 | 7.57 (-4.40, 21.04) | 7.35 (-3.90, 19.92) | 52.81 (0.46, 132.45) | 4.48 (-4.33, 14.10) | 16.78 (-7.30, 47.12) |
| Yes | ≥ 16.87 | 30.42 (17.41, 44.86) | 27.89 (16.01, 41.00) | 9.80 (-28.73, 69.16) | 28.97 (19.32, 39.40) | 32.35 (8.31, 61.73) |
| RERI |  | 0.09 (-0.07, 0.25) | 0.06 (-0.09, 0.20) | -0.60 (-1.34, 0.14) | 0.11 (-0.01, 0.22) | -0.03 (-0.35, 0.29) |
| 10^th^ (3d) ^a^ |  |  |  |  |  |  |
| No | < 16.87 | 1.00 | 1.00 | 1.00 | 1.00 | 1.00 |
| No | ≥ 16.87 | 13.02 (5.05, 21.59) | 15.13 (7.45, 23.35) | 16.92 (-11.93, 55.23) | 13.31 (7.28, 19.68) | 18.19 (2.63, 36.12) |
| Yes | < 16.87 | 3.07 (-9.64, 17.56) | 8.28 (-4.17, 22.35) | 61.19 (-0.48, 161.06) | 2.67 (-6.88, 13.20) | 15.11 (-10.75, 48.47) |
| Yes | ≥ 16.87 | 26.80 (13.83, 41.24) | 26.95 (14.91, 40.25) | 8.45 (-31.26, 71.10) | 27.01 (17.31, 37.51) | 28.38 (4.46, 57.79) |
| RERI |  | 0.11 (-0.06, 0.27) | 0.04 (-0.12, 0.19) | -0.70 (-1.56, 0.16) | 0.11 (-0.01, 0.23) | -0.05 (-0.39, 0.29) |
| 10^th^ (4d) ^a^ |  |  |  |  |  |  |
| No | < 16.87 | 1.00 | 1.00 | 1.00 | 1.00 | 1.00 |
| No | ≥ 16.87 | 13.31 (5.49, 21.70) | 13.87 (6.44, 21.82) | 11.42 (-15.72, 47.30) | 13.10 (7.21, 19.31) | 17.21 (2.19, 34.43) |
| Yes | < 16.87 | 2.88 (-11.27, 19.30) | 4.20 (-9.09, 19.43) | 47.38 (-14.93, 155.34) | 0.46 (-9.96, 12.08) | 17.25 (-11.62, 55.54) |
| Yes | ≥ 16.87 | 25.22 (11.51, 40.63) | 28.71 (15.65, 43.25) | 13.67 (-30.39, 85.63) | 26.83 (16.45, 38.13) | 31.89 (5.66, 64.63) |
| RERI |  | 0.09 (-0.10, 0.28) | 0.11 (-0.07, 0.29) | -0.45 (-1.37, 0.47) | 0.13 (-0.01, 0.27) | -0.03 (-0.43, 0.38) |
| 7.5^th^ (2d) ^a^ |  |  |  |  |  |  |
| No | < 16.87 | 1.00 | 1.00 | 1.00 | 1.00 | 1.00 |
| No | ≥ 16.87 | 12.09 (4.20, 20.59) | 14.48 (6.86, 22.64) | 16.20 (-12.31, 53.97) | 12.70 (6.71, 19.02) | 15.96 (0.70, 33.53) |
| Yes | < 16.87 | 1.82 (-10.81, 16.24) | 5.82 (-6.36, 19.58) | 60.70 (-0.53, 159.61) | 1.45 (-8.04, 11.91) | 7.36 (-16.67, 38.31) |
| Yes | ≥ 16.87 | 33.35 (19.33, 49.03) | 28.21 (15.55, 42.25) | 8.43 (-32.15, 73.29) | 31.10 (20.72, 42.37) | 30.01 (5.00, 60.97) |
| RERI |  | 0.19 (0.02, 0.36) | 0.08 (-0.08, 0.24) | -0.68 (-1.55, 0.18) | 0.17 (0.04, 0.29) | 0.07 (-0.27, 0.40) |
| 7.5^th^ (3d) ^a^ |  |  |  |  |  |  |
| No | < 16.87 | 1.00 | 1.00 | 1.00 | 1.00 | 1.00 |
| No | ≥ 16.87 | 12.23 (4.45, 20.60) | 15.15 (7.60, 23.23) | 14.12 (-13.60, 50.72) | 13.13 (7.21, 19.38) | 17.10 (1.95, 34.49) |
| Yes | < 16.87 | 0.61 (-12.78, 16.06) | 9.25 (-4.14, 24.50) | 61.93 (-3.26, 171.03) | 1.99 (-8.21, 13.33) | 14.10 (-13.04, 49.72) |
| Yes | ≥ 16.87 | 31.28 (17.00, 47.31) | 29.42 (16.34, 43.97) | 13.79 (-30.28, 85.70) | 30.21 (19.61, 41.74) | 33.21 (6.85, 66.08) |
| RERI |  | 0.18 (0.01, 0.37) | 0.05 (-0.13, 0.23) | -0.62 (-1.56, 0.31) | 0.15 (0.01, 0.29) | 0.02 (-0.36, 0.40) |
| 7.5^th^ (4d) ^a^ |  |  |  |  |  |  |
| No | < 16.87 | 1.00 | 1.00 | 1.00 | 1.00 | 1.00 |
| No | ≥ 16.87 | 11.44 (3.88, 19.55) | 13.76 (6.46, 21.56) | 6.51 (-18.86, 39.82) | 12.45 (6.69, 18.52) | 15.16 (0.63, 31.77) |
| Yes | < 16.87 | -7.00 (-21.62, 10.34) | 2.97 (-11.61, 19.96) | 26.53 (-32.45, 137.00) | -3.68 (-14.94, 9.07) | 6.78 (-22.30, 46.75) |
| Yes | ≥ 16.87 | 29.29 (13.69, 47.04) | 29.91 (15.42, 46.21) | 13.27 (-33.73, 93.60) | 29.76 (18.09, 42.59) | 32.73 (3.33, 70.49) |
| RERI |  | 0.25 (0.04, 0.46) | 0.13 (-0.07, 0.34) | -0.20 (-1.13, 0.74) | 0.21 (0.05, 0.37) | 0.11 (-0.33, 0.55) |
| 5^th^ (2d) ^a^ |  |  |  |  |  |  |
| No | < 16.87 | 1.00 | 1.00 | 1.00 | 1.00 | 1.00 |
| No | ≥ 16.87 | 10.86 (3.17, 19.13) | 11.81 (4.51, 19.62) | 9.81 (-16.72, 44.80) | 10.90 (5.11, 17.01) | 14.27 (-0.52, 31.27) |
| Yes | < 16.87 | -1.92 (-14.86, 12.98) | -5.45 (-17.16, 7.92) | 36.20 (-18.81, 128.48) | -6.31 (-15.71, 4.13) | 3.22 (-21.10, 35.03) |
| Yes | ≥ 16.87 | 45.65 (28.65, 64.90) | 28.90 (14.93, 44.58) | 9.47 (-35.10, 84.66) | 36.29 (24.39, 49.33) | 40.79 (10.80, 78.88) |
| RERI |  | 0.37 (0.16, 0.57) | 0.23 (0.05, 0.40) | -0.37 (-1.22, 0.49) | 0.32 (0.17, 0.46) | 0.23 (-0.16, 0.63) |
| 5^th^ (3d) ^a^ |  |  |  |  |  |  |
| No | < 16.87 | 1.00 | 1.00 | 1.00 | 1.00 | 1.00 |
| No | ≥ 16.87 | 9.82 (2.38, 17.80) | 12.89 (5.68, 20.60) | 3.38 (-21.20, 35.64) | 11.19 (5.52, 17.17) | 14.75 (0.27, 31.32) |
| Yes | < 16.87 | -10.98 (-25.17, 5.89) | 0.77 (-13.72, 17.69) | 4.70 (-45.70, 101.88) | -7.17 (-18.20, 5.34) | 10.63 (-19.59, 52.22) |
| Yes | ≥ 16.87 | 46.65 (28.50, 67.37) | 37.04 (21.42, 54.67) | 15.18 (-33.13, 98.40) | 40.75 (27.73, 55.09) | 50.44 (17.17, 93.15) |
| RERI |  | 0.48 (0.25, 0.71) | 0.23 (0.02, 0.45) | 0.07 (-0.81, 0.95) | 0.37 (0.20, 0.54) | 0.25 (-0.24, 0.74) |
| 5^th^ (4d) ^a^ |  |  |  |  |  |  |
| No | < 16.87 | 1.00 | 1.00 | 1.00 | 1.00 | 1.00 |
| No | ≥ 16.87 | 9.07 (1.73, 16.94) | 11.97 (4.86, 19.56) | 2.13 (-21.99, 33.71) | 10.53 (4.92, 16.44) | 12.75 (-1.36, 28.88) |
| Yes | < 16.87 | -18.65 (-33.01, -1.20) | -4.28 (-19.13, 13.30) | -9.66 (-56.68, 88.37) | -11.83 (-23.29, 1.33) | -5.04 (-32.91, 34.40) |
| Yes | ≥ 16.87 | 55.83 (33.95, 81.28) | 47.66 (28.64, 69.48) | 2.40 (-44.66, 89.47) | 52.71 (36.74, 70.55) | 53.45 (14.53, 105.60) |
| RERI |  | 0.65 (0.39, 0.92) | 0.40 (0.15, 0.65) | 0.10 (-0.78, 0.98) | 0.54 (0.34, 0.74) | 0.46 (-0.07, 0.98) |
| Shenzhen |  |  |  |  |  |  |
| 10^th^ (2d) ^a^ |  |  |  |  |  |  |
| No | < 14.71 | 1.00 | 1.00 | 1.00 | 1.00 | 1.00 |
| No | ≥ 14.71 | 13.58 (9.15, 18.19) | 9.33 (5.33, 13.48) | -4.80 (-17.37, 9.69) | 11.68 (8.51, 14.95) | 14.58 (3.58, 26.74) |
| Yes | < 14.71 | 8.00 (1.16, 15.31) | 9.65 (2.98, 16.74) | -7.99 (-29.76, 20.52) | 9.35 (4.26, 14.70) | 10.00 (-7.15, 30.33) |
| Yes | ≥ 14.71 | 20.12 (13.41, 27.23) | 19.34 (13.09, 25.93) | -0.51 (-19.17, 22.46) | 19.98 (15.09, 25.08) | 26.83 (9.78, 46.53) |
| RERI |  | -0.01 (-0.10, 0.07) | 0.01 (-0.07, 0.08) | 0.12 (-0.15, 0.40) | -0.01 (-0.07, 0.05) | 0.02 (-0.19, 0.24) |
| 10^th^ (3d) ^a^ |  |  |  |  |  |  |
| No | < 14.71 | 1.00 | 1.00 | 1.00 | 1.00 | 1.00 |
| No | ≥ 14.71 | 13.26 (8.83, 17.87) | 9.02 (5.02, 13.18) | -2.44 (-15.50, 12.63) | 11.52 (8.34, 14.79) | 10.64 (0.01, 22.39) |
| Yes | < 14.71 | 7.97 (-0.04, 16.63) | 8.56 (0.82, 16.89) | 5.97 (-22.34, 44.61) | 9.11 (3.14, 15.41) | -1.43 (-19.63, 20.89) |
| Yes | ≥ 14.71 | 18.89 (12.04, 26.17) | 17.40 (11.07, 24.09) | -1.67 (-20.79, 22.07) | 18.60 (13.61, 23.80) | 21.69 (4.96, 41.09) |
| RERI |  | -0.02 (-0.11, 0.07) | -0.01 (-0.09, 0.08) | -0.05 (-0.40, 0.29) | -0.02 (-0.09, 0.05) | 0.12 (-0.09, 0.34) |
| 10^th^ (4d) ^a^ |  |  |  |  |  |  |
| No | < 14.71 | 1.00 | 1.00 | 1.00 | 1.00 | 1.00 |
| No | ≥ 14.71 | 11.34 (7.10, 15.76) | 7.73 (3.88, 11.71) | -4.36 (-16.86, 10.02) | 10.01 (6.96, 13.15) | 8.16 (-1.95, 19.32) |
| Yes | < 14.71 | 1.67 (-6.27, 10.28) | 4.86 (-2.94, 13.30) | 3.52 (-25.41, 43.67) | 4.77 (-1.23, 11.14) | -14.72 (-31.30, 5.86) |
| Yes | ≥ 14.71 | 15.55 (8.88, 22.64) | 16.32 (10.07, 22.93) | 0.75 (-18.80, 25.01) | 16.06 (11.18, 21.16) | 21.93 (5.29, 41.21) |
| RERI |  | 0.03 (-0.07, 0.12) | 0.04 (-0.05, 0.13) | 0.02 (-0.35, 0.38) | 0.01 (-0.06, 0.08) | 0.28 (0.06, 0.51) |
| 7.5^th^ (2d) ^a^ |  |  |  |  |  |  |
| No | < 14.71 | 1.00 | 1.00 | 1.00 | 1.00 | 1.00 |
| No | ≥ 14.71 | 13.36 (8.91, 18.00) | 10.08 (6.03, 14.28) | -2.41 (-15.32, 12.48) | 11.96 (8.76, 15.26) | 13.76 (2.79, 25.90) |
| Yes | < 14.71 | 6.35 (-1.02, 14.27) | 9.87 (2.63, 17.61) | 1.25 (-24.73, 36.20) | 8.74 (3.22, 14.56) | 3.91 (-13.58, 24.94) |
| Yes | ≥ 14.71 | 20.16 (12.81, 27.98) | 17.48 (10.78, 24.59) | -2.18 (-21.80, 22.37) | 19.28 (13.96, 24.84) | 22.51 (4.59, 43.51) |
| RERI |  | 0.01 (-0.09, 0.10) | -0.02 (-0.11, 0.06) | -0.01 (-0.34, 0.32) | -0.01 (-0.08, 0.05) | 0.05 (-0.18, 0.28) |
| 7.5^th^ (3d) ^a^ |  |  |  |  |  |  |
| No | < 14.71 | 1.00 | 1.00 | 1.00 | 1.00 | 1.00 |
| No | ≥ 14.71 | 13.61 (9.22, 18.17) | 10.04 (6.05, 14.18) | -2.50 (-15.43, 12.40) | 12.19 (9.03, 15.44) | 12.37 (1.72, 24.14) |
| Yes | < 14.71 | 9.20 (1.26, 17.75) | 11.01 (3.32, 19.29) | 6.05 (-22.22, 44.59) | 10.68 (4.77, 16.92) | 4.70 (-13.86, 27.26) |
| Yes | ≥ 14.71 | 20.21 (12.53, 28.42) | 15.87 (8.96, 23.22) | -1.24 (-22.07, 25.16) | 17.90 (12.40, 23.67) | 27.01 (7.91, 49.50) |
| RERI |  | -0.03 (-0.12, 0.07) | -0.05 (-0.14, 0.04) | -0.05 (-0.41, 0.31) | -0.05 (-0.12, 0.02) | 0.10 (-0.15, 0.35) |
| 7.5^th^ (4d) ^a^ |  |  |  |  |  |  |
| No | < 14.71 | 1.00 | 1.00 | 1.00 | 1.00 | 1.00 |
| No | ≥ 14.71 | 11.70 (7.51, 16.04) | 8.56 (4.74, 12.51) | -3.87 (-16.32, 10.44) | 10.51 (7.50, 13.61) | 10.35 (0.20, 21.54) |
| Yes | < 14.71 | 3.88 (-4.59, 13.09) | 7.16 (-1.08, 16.09) | 10.62 (-21.81, 56.50) | 6.60 (0.25, 13.34) | -8.33 (-26.45, 14.26) |
| Yes | ≥ 14.71 | 16.96 (9.19, 25.28) | 12.91 (5.87, 20.40) | 5.61 (-17.75, 35.61) | 14.48 (8.91, 20.32) | 23.03 (3.75, 45.90) |
| RERI |  | 0.01 (-0.10, 0.12) | -0.03 (-0.13, 0.08) | -0.01 (-0.44, 0.42) | -0.03 (-0.11, 0.05) | 0.21 (-0.05, 0.47) |
| 5^th^ (2d) ^a^ |  |  |  |  |  |  |
| No | < 14.71 | 1.00 | 1.00 | 1.00 | 1.00 | 1.00 |
| No | ≥ 14.71 | 11.29 (7.08, 15.67) | 7.20 (3.40, 11.14) | -3.83 (-16.30, 10.49) | 9.53 (6.51, 12.63) | 9.68 (-0.47, 20.85) |
| Yes | < 14.71 | -1.69 (-9.39, 6.66) | -2.59 (-9.88, 5.29) | -6.30 (-33.43, 31.88) | -0.92 (-6.62, 5.13) | -15.94 (-32.09, 4.03) |
| Yes | ≥ 14.71 | 19.18 (11.57, 27.32) | 14.24 (7.45, 21.45) | 1.02 (-20.32, 28.08) | 17.28 (11.83, 23.00) | 13.83 (-3.32, 34.01) |
| RERI |  | 0.10 (-0.01, 0.19) | 0.10 (0.01, 0.19) | 0.11 (-0.24, 0.47) | 0.09 (0.02, 0.16) | 0.20 (-0.02, 0.42) |
| 5^th^ (3d) ^a^ |  |  |  |  |  |  |
| No | < 14.71 | 1.00 | 1.00 | 1.00 | 1.00 | 1.00 |
| No | ≥ 14.71 | 10.33 (6.21, 14.60) | 7.22 (3.46, 11.11) | -3.17 (-15.74, 11.28) | 9.05 (6.09, 12.10) | 9.31 (-0.70, 20.33) |
| Yes | < 14.71 | -10.66 (-19.00, -1.46) | -5.20 (-13.45, 3.83) | -0.51 (-32.22, 46.04) | -7.08 (-13.40, -0.31) | -19.12 (-37.04, 3.89) |
| Yes | ≥ 14.71 | 11.29 (3.72, 19.41) | 9.31 (2.37, 16.72) | -6.95 (-28.22, 20.62) | 11.00 (5.49, 16.79) | 9.50 (-7.96, 30.26) |
| RERI |  | 0.12 (0.01, 0.22) | 0.07 (-0.03, 0.17) | -0.03 (-0.44, 0.38) | 0.09 (0.01, 0.17) | 0.19 (-0.05, 0.44) |
| 5^th^ (4d) ^a^ |  |  |  |  |  |  |
| No | < 14.71 | 1.00 | 1.00 | 1.00 | 1.00 | 1.00 |
| No | ≥ 14.71 | 11.02 (6.95, 15.24) | 7.81 (4.10, 11.65) | -2.81 (-15.22, 11.42) | 9.72 (6.79, 12.73) | 9.74 (-0.12, 20.56) |
| Yes | < 14.71 | -3.00 (-13.34, 8.58) | 2.27 (-7.83, 13.47) | 17.38 (-23.99, 81.28) | 1.26 (-6.59, 9.76) | -21.71 (-41.27, 4.37) |
| Yes | ≥ 14.71 | 13.36 (4.71, 22.72) | 11.36 (3.38, 19.96) | -6.17 (-30.81, 27.24) | 12.56 (6.27, 19.22) | 18.07 (-2.92, 43.60) |
| RERI |  | 0.05 (-0.08, 0.18) | 0.01 (-0.11, 0.14) | -0.21 (-0.76, 0.34) | 0.02 (-0.08, 0.11) | 0.30 (0.01, 0.60) |
| Zhaoqing |  |  |  |  |  |  |
| 10^th^ (2d) ^b^ |  |  |  |  |  |  |
| No | < 13.41 | 1.00 | 1.00 | 1.00 | 1.00 | 1.00 |
| No | ≥ 13.41 | 2.01 (-5.99, 10.69) | -0.86 (-8.32, 7.21) | -5.96 (-34.70, 35.44) | -0.16 (-6.23, 6.31) | 5.15 (-8.50, 20.84) |
| Yes | < 13.41 | -13.67 (-26.03, 0.76) | -2.89 (-15.96, 12.21) | -46.92 (-75.18, 13.52) | -7.76 (-17.81, 3.53) | -1.76 (-25.56, 29.65) |
| Yes | ≥ 13.41 | 0.86 (-16.55, 21.89) | -7.22 (-23.10, 11.93) | 18.12 (-52.15, 191.62) | -2.28 (-15.57, 13.11) | -10.64 (-36.98, 26.72) |
| RERI |  | 0.13 (-0.09, 0.34) | -0.03 (-0.24, 0.17) | 0.71 (-0.32, 1.74) | 0.06 (-0.11, 0.22) | -0.14 (-0.53, 0.25) |
| 10^th^ (3d) ^b^ |  |  |  |  |  |  |
| No | < 13.41 | 1.00 | 1.00 | 1.00 | 1.00 | 1.00 |
| No | ≥ 13.41 | 1.22 (-6.62, 9.72) | -0.87 (-8.24, 7.08) | -5.42 (-34.17, 35.87) | -0.44 (-6.42, 5.91) | 4.23 (-9.17, 19.61) |
| Yes | < 13.41 | -20.84 (-33.34, -5.99) | -1.25 (-15.65, 15.62) | -51.30 (-79.15, 13.78) | -10.77 (-21.42, 1.32) | -2.61 (-27.98, 31.68) |
| Yes | ≥ 13.41 | 3.47 (-14.80, 25.67) | 0.33 (-17.19, 21.57) | 21.91 (-52.06, 210.00) | 1.48 (-12.67, 17.92) | 2.84 (-27.66, 46.19) |
| RERI |  | 0.23 (0.01, 0.46) | 0.02 (-0.21, 0.26) | 0.79 (-0.33, 1.90) | 0.13 (-0.05, 0.31) | 0.01 (-0.42, 0.45) |
| 10^th^ (4d) ^b^ |  |  |  |  |  |  |
| No | < 13.41 | 1.00 | 1.00 | 1.00 | 1.00 | 1.00 |
| No | ≥ 13.41 | 1.61 (-6.18, 10.05) | -0.01 (-7.36, 7.93) | 2.88 (-27.85, 46.69) | 0.09 (-5.85, 6.41) | 4.33 (-8.96, 19.56) |
| Yes | < 13.41 | -14.43 (-28.69, 2.69) | 6.92 (-9.43, 26.21) | -20.93 (-65.91, 83.39) | -3.64 (-15.80, 10.28) | 2.37 (-25.15, 40.01) |
| Yes | ≥ 13.41 | 25.11 (0.73, 55.39) | 10.48 (-10.79, 36.83) | 24.63 (-60.69, 295.12) | 13.75 (-3.80, 34.51) | 39.61 (-5.01, 105.20) |
| RERI |  | 0.38 (0.08, 0.67) | 0.04 (-0.24, 0.32) | 0.43 (-1.07, 1.92) | 0.17 (-0.05, 0.39) | 0.33 (-0.25, 0.91) |
| 7.5^th^ (2d) ^b^ |  |  |  |  |  |  |
| No | < 13.41 | 1.00 | 1.00 | 1.00 | 1.00 | 1.00 |
| No | ≥ 13.41 | 2.52 (-5.36, 11.05) | -0.52 (-7.85, 7.39) | -1.62 (-31.21, 40.70) | 0.30 (-5.67, 6.64) | 4.59 (-8.72, 19.84) |
| Yes | < 13.41 | -13.60 (-26.83, 2.04) | 1.13 (-13.34, 18.01) | -40.03 (-73.11, 33.71) | -5.57 (-16.60, 6.91) | -2.51 (-27.16, 30.48) |
| Yes | ≥ 13.41 | 4.88 (-15.87, 30.76) | 0.90 (-18.48, 24.89) | 4.95 (-65.50, 219.25) | 4.44 (-11.71, 23.53) | -4.89 (-36.25, 41.90) |
| RERI |  | 0.16 (-0.10, 0.42) | 0.01 (-0.25, 0.26) | 0.47 (-0.73, 1.67) | 0.10 (-0.10, 0.30) | -0.07 (-0.53, 0.39) |
| 7.5^th^ (3d) ^b^ |  |  |  |  |  |  |
| No | < 13.41 | 1.00 | 1.00 | 1.00 | 1.00 | 1.00 |
| No | ≥ 13.41 | 2.45 (-5.21, 10.73) | -0.89 (-8.00, 6.78) | 4.52 (-26.01, 47.64) | 0.08 (-5.72, 6.25) | 3.33 (-9.43, 17.89) |
| Yes | < 13.41 | -23.59 (-37.77, -6.19) | -4.00 (-20.12, 15.37) | -20.33 (-67.90, 97.78) | -13.09 (-25.18, 0.95) | -15.71 (-41.34, 21.13) |
| Yes | ≥ 13.41 | 12.32 (-10.99, 41.75) | -3.19 (-22.74, 21.30) | -0.48 (-69.88, 228.90) | 5.04 (-12.07, 25.48) | -0.29 (-34.14, 50.95) |
| RERI |  | 0.33 (0.04, 0.63) | 0.02 (-0.26, 0.29) | 0.15 (-1.21, 1.51) | 0.18 (-0.04, 0.40) | 0.12 (-0.39, 0.63) |
| 7.5^th^ (4d) ^b^ |  |  |  |  |  |  |
| No | < 13.41 | 1.00 | 1.00 | 1.00 | 1.00 | 1.00 |
| No | ≥ 13.41 | 2.74 (-4.92, 11.01) | -0.25 (-7.38, 7.44) | 6.77 (-24.37, 50.73) | 0.65 (-5.17, 6.83) | 3.05 (-9.64, 17.53) |
| Yes | < 13.41 | -18.34 (-34.00, 1.04) | 4.91 (-13.08, 26.62) | -6.26 (-61.25, 126.76) | -3.95 (-17.69, 12.09) | -19.18 (-44.46, 17.59) |
| Yes | ≥ 13.41 | 16.19 (-12.35, 54.02) | 5.60 (-19.70, 38.85) | -51.02 (-94.56, 341.43) | 8.94 (-12.23, 35.21) | 25.55 (-22.68, 103.87) |
| RERI |  | 0.32 (-0.04, 0.68) | 0.01 (-0.33, 0.35) | -0.51 (-1.90, 0.87) | 0.12 (-0.15, 0.39) | 0.42 (-0.25, 1.08) |
| 5^th^ (2d) ^b^ |  |  |  |  |  |  |
| No | < 13.41 | 1.00 | 1.00 | 1.00 | 1.00 | 1.00 |
| No | ≥ 13.41 | 2.77 (-4.95, 11.12) | -0.38 (-7.56, 7.36) | 0.41 (-29.13, 42.25) | 0.70 (-5.16, 6.92) | 3.62 (-9.31, 18.39) |
| Yes | < 13.41 | -23.14 (-38.37, -4.15) | 3.43 (-14.82, 25.60) | -47.46 (-80.44, 41.13) | -8.80 (-22.34, 7.11) | -6.32 (-35.06, 35.14) |
| Yes | ≥ 13.41 | -2.81 (-25.24, 26.35) | 1.09 (-20.70, 28.87) | 4.11 (-68.87, 248.22) | -3.69 (-21.00, 17.41) | 16.95 (-24.24, 80.56) |
| RERI |  | 0.18 (-0.13, 0.48) | -0.02 (-0.33, 0.30) | 0.51 (-0.82, 1.84) | 0.04 (-0.20, 0.28) | 0.20 (-0.41, 0.80) |
| 5^th^ (3d) ^b^ |  |  |  |  |  |  |
| No | < 13.41 | 1.00 | 1.00 | 1.00 | 1.00 | 1.00 |
| No | ≥ 13.41 | 3.18 (-4.46, 11.43) | -0.31 (-7.41, 7.34) | 6.01 (-24.77, 49.37) | 0.88 (-4.92, 7.03) | 3.28 (-9.43, 17.78) |
| Yes | < 13.41 | -29.28 (-44.80, -9.39) | 5.82 (-13.76, 29.85) | -16.42 (-68.53, 122.00) | -10.78 (-25.04, 6.19) | -11.58 (-40.46, 31.29) |
| Yes | ≥ 13.41 | -4.72 (-30.09, 29.85) | 4.24 (-21.83, 39.00) | -57.56 (-95.11, 268.37) | -3.13 (-23.36, 22.44) | 25.12 (-23.99, 105.94) |
| RERI |  | 0.21 (-0.13, 0.56) | -0.01 (-0.38, 0.35) | -0.47 (-1.73, 0.79) | 0.07 (-0.21, 0.34) | 0.33 (-0.37, 1.04) |
| 5^th^ (4d) ^b^ |  |  |  |  |  |  |
| No | < 13.41 | 1.00 | 1.00 | 1.00 | 1.00 | 1.00 |
| No | ≥ 13.41 | 2.78 (-4.83, 10.98) | -0.84 (-7.90, 6.76) | 5.02 (-25.50, 48.05) | 0.25 (-5.51, 6.36) | 3.41 (-9.30, 17.90) |
| Yes | < 13.41 | -27.01 (-43.47, -5.76) | -0.33 (-19.81, 23.88) | -26.71 (-74.25, 108.61) | -12.33 (-27.02, 5.30) | -14.60 (-43.39, 28.82) |
| Yes | ≥ 13.41 | 4.32 (-27.25, 49.60) | 18.36 (-14.53, 63.92) | -42.67 (-93.88, 437.19) | 6.46 (-18.68, 39.37) | 50.31 (-14.83, 165.25) |
| RERI |  | 0.29 (-0.13, 0.70) | 0.20 (-0.24, 0.63) | -0.21 (-1.72, 1.30) | 0.19 (-0.14, 0.51) | 0.62 (-0.29, 1.52) |

Note: NO_2_, nitrogen dioxide.

^a^ Cold spells were defined by percentile temperature thresholds (10^th^, 7.5^th^, 5^th^ and 2.5^th^) and by the number of consecutive days below the thresholds (2-4 d).

^b^ Nine definitions [10^th^ (2d), 10^th^ (3d), 10^th^ (4d), 7.5^th^ (2d), 7.5^th^ (3d), 7.5^th^ (4d), 5^th^ (2d), 5^th^ (3d), and 5^th^ (4d)] were used in Zhaoqing.

^c^ Air pollutants were classified as binary variables using the median of air pollutant (lag03) concentrations as a cut off.

## Table S15. Percent changes and relative excess risk due to interaction (RERI) of cold spells and SO_2_ exposure on anxiety stratified by sex and age in three subtropical cities in China.

| Cold spells | SO_2_ ^c^ (µg/m^3^) | male | female | < 18 | 18-65 | ≥ 65 |
| --- | --- | --- | --- | --- | --- | --- |
| Huizhou |  |  |  |  |  |  |
| 10^th^ (2d) ^a^ |  |  |  |  |  |  |
| No | < 14.48 | 1.00 | 1.00 | 1.00 | 1.00 | 1.00 |
| No | ≥ 14.48 | 7.95 (1.75, 14.54) | 6.10 (0.40, 12.12) | 26.08 (0.85, 57.64) | 5.81 (1.26, 10.56) | 10.29 (-1.80, 23.87) |
| Yes | < 14.48 | 3.80 (-7.00, 15.86) | 5.24 (-4.93, 16.50) | 21.61 (-17.86, 80.04) | 2.91 (-5.10, 11.60) | 11.97 (-10.03, 39.36) |
| Yes | ≥ 14.48 | 24.78 (13.11, 37.64) | 17.41 (7.18, 28.62) | 43.14 (-3.49, 112.29) | 19.66 (11.27, 28.68) | 23.30 (2.22, 48.73) |
| RERI |  | 0.13 (-0.02, 0.28) | 0.06 (-0.07, 0.20) | -0.05 (-0.70, 0.61) | 0.11 (0.01, 0.22) | 0.01 (-0.29, 0.31) |
| 10^th^ (3d) ^a^ |  |  |  |  |  |  |
| No | < 14.48 | 1.00 | 1.00 | 1.00 | 1.00 | 1.00 |
| No | ≥ 14.48 | 7.75 (1.66, 14.20) | 6.12 (0.50, 12.05) | 28.75 (3.35, 60.40) | 5.74 (1.27, 10.41) | 9.42 (-2.41, 22.67) |
| Yes | < 14.48 | 0.37 (-11.07, 13.29) | 5.33 (-5.74, 17.70) | 35.73 (-12.20, 109.83) | 1.34 (-7.28, 10.75) | 6.88 (-15.98, 35.97) |
| Yes | ≥ 14.48 | 21.10 (9.46, 33.98) | 16.50 (6.10, 27.91) | 31.07 (-13.69, 99.03) | 17.82 (9.35, 26.94) | 20.54 (-0.66, 46.26) |
| RERI |  | 0.13 (-0.03, 0.29) | 0.05 (-0.09, 0.20) | -0.33 (-1.07, 0.40) | 0.11 (-0.01, 0.22) | 0.04 (-0.27, 0.35) |
| 10^th^ (4d) ^a^ |  |  |  |  |  |  |
| No | < 14.48 | 1.00 | 1.00 | 1.00 | 1.00 | 1.00 |
| No | ≥ 14.48 | 7.13 (1.16, 13.46) | 5.30 (-0.19, 11.10) | 23.60 (-0.54, 53.61) | 5.39 (1.00, 9.97) | 7.31 (-4.10, 20.09) |
| Yes | < 14.48 | -6.03 (-18.32, 8.11) | 1.18 (-10.73, 14.69) | 16.22 (-30.00, 92.95) | -2.57 (-11.92, 7.78) | -2.36 (-26.42, 29.57) |
| Yes | ≥ 14.48 | 21.43 (8.99, 35.30) | 18.50 (7.17, 31.04) | 48.42 (-5.66, 133.49) | 17.96 (8.87, 27.81) | 27.62 (4.03, 56.54) |
| RERI |  | 0.20 (0.03, 0.38) | 0.12 (-0.04, 0.28) | 0.09 (-0.73, 0.90) | 0.15 (0.02, 0.28) | 0.23 (-0.12, 0.58) |
| 7.5^th^ (2d) ^a^ |  |  |  |  |  |  |
| No | < 14.48 | 1.00 | 1.00 | 1.00 | 1.00 | 1.00 |
| No | ≥ 14.48 | 8.34 (2.22, 14.82) | 5.88 (0.29, 11.78) | 28.34 (3.05, 59.83) | 5.79 (1.33, 10.45) | 10.33 (-1.56, 23.65) |
| Yes | < 14.48 | 4.64 (-6.92, 17.63) | 3.42 (-7.26, 15.33) | 32.63 (-12.48, 100.99) | 2.02 (-6.45, 11.27) | 10.16 (-12.70, 39.01) |
| Yes | ≥ 14.48 | 27.18 (14.18, 41.65) | 18.32 (7.05, 30.77) | 37.79 (-12.73, 117.54) | 22.15 (12.79, 32.28) | 19.72 (-2.55, 47.08) |
| RERI |  | 0.14 (-0.02, 0.31) | 0.09 (-0.06, 0.24) | -0.23 (-0.99, 0.52) | 0.14 (0.03, 0.26) | -0.01 (-0.33, 0.31) |
| 7.5^th^ (3d) ^a^ |  |  |  |  |  |  |
| No | < 14.48 | 1.00 | 1.00 | 1.00 | 1.00 | 1.00 |
| No | ≥ 14.48 | 8.16 (2.11, 14.55) | 6.22 (0.66, 12.09) | 28.37 (3.21, 59.66) | 5.90 (1.47, 10.52) | 10.51 (-1.29, 23.72) |
| Yes | < 14.48 | 2.34 (-10.08, 16.48) | 6.21 (-5.64, 19.56) | 41.25 (-10.41, 122.71) | 1.78 (-7.43, 11.91) | 15.08 (-11.03, 48.85) |
| Yes | ≥ 14.48 | 24.81 (11.62, 39.56) | 18.93 (7.33, 31.79) | 41.16 (-12.06, 126.58) | 20.90 (11.36, 31.24) | 22.53 (-0.89, 51.49) |
| RERI |  | 0.14 (-0.03, 0.32) | 0.06 (-0.10, 0.23) | -0.28 (-1.13, 0.56) | 0.13 (0.01, 0.26) | -0.03 (-0.40, 0.34) |
| 7.5^th^ (4d) ^a^ |  |  |  |  |  |  |
| No | < 14.48 | 1.00 | 1.00 | 1.00 | 1.00 | 1.00 |
| No | ≥ 14.48 | 7.81 (1.84, 14.12) | 5.51 (0.05, 11.27) | 25.17 (0.91, 55.26) | 5.52 (1.15, 10.07) | 9.52 (-2.05, 22.46) |
| Yes | < 14.48 | -4.24 (-18.15, 12.03) | 0.86 (-12.29, 15.98) | 25.17 (-29.59, 122.52) | -3.51 (-13.78, 8.00) | 9.92 (-19.99, 51.00) |
| Yes | ≥ 14.48 | 22.66 (8.22, 39.03) | 19.91 (6.91, 34.49) | 39.21 (-17.73, 135.56) | 20.80 (10.18, 32.43) | 21.27 (-4.29, 53.66) |
| RERI |  | 0.19 (-0.01, 0.39) | 0.14 (-0.06, 0.32) | -0.11 (-1.07, 0.85) | 0.19 (0.04, 0.33) | 0.02 (-0.41, 0.45) |
| 5^th^ (2d) ^a^ |  |  |  |  |  |  |
| No | < 14.48 | 1.00 | 1.00 | 1.00 | 1.00 | 1.00 |
| No | ≥ 14.48 | 7.60 (1.60, 13.94) | 5.01 (-0.46, 10.77) | 26.38 (1.93, 56.69) | 5.06 (0.69, 9.62) | 9.41 (-2.21, 22.41) |
| Yes | < 14.48 | 2.06 (-10.07, 15.83) | -3.73 (-14.50, 8.38) | 28.98 (-19.23, 105.96) | -3.34 (-12.01, 6.17) | 8.09 (-16.10, 39.25) |
| Yes | ≥ 14.48 | 38.84 (22.57, 57.27) | 18.68 (5.86, 33.05) | 34.04 (-21.07, 127.64) | 27.07 (15.95, 39.26) | 27.00 (0.47, 60.54) |
| RERI |  | 0.29 (0.09, 0.49) | 0.17 (0.01, 0.34) | -0.21 (-1.11, 0.68) | 0.25 (0.11, 0.39) | 0.10 (-0.29, 0.48) |
| 5^th^ (3d) ^a^ |  |  |  |  |  |  |
| No | < 14.48 | 1.00 | 1.00 | 1.00 | 1.00 | 1.00 |
| No | ≥ 14.48 | 7.60 (1.67, 13.88) | 5.99 (0.52, 11.75) | 23.17 (-0.57, 52.58) | 5.69 (1.34, 10.23) | 10.20 (-1.42, 23.19) |
| Yes | < 14.48 | -0.14 (-14.20, 16.24) | 6.27 (-7.29, 21.81) | 8.67 (-39.58, 95.45) | 0.90 (-9.61, 12.63) | 22.57 (-8.77, 64.69) |
| Yes | ≥ 14.48 | 37.81 (20.36, 57.79) | 24.27 (9.87, 40.55) | 42.39 (-18.42, 148.53) | 29.20 (16.97, 42.70) | 34.37 (4.70, 72.44) |
| RERI |  | 0.30 (0.07, 0.53) | 0.12 (-0.08, 0.32) | 0.11 (-0.88, 1.09) | 0.23 (0.06, 0.39) | 0.02 (-0.46, 0.49) |
| 5^th^ (4d) ^a^ |  |  |  |  |  |  |
| No | < 14.48 | 1.00 | 1.00 | 1.00 | 1.00 | 1.00 |
| No | ≥ 14.48 | 6.67 (0.82, 12.85) | 5.06 (-0.33, 10.74) | 21.75 (-1.58, 50.59) | 4.97 (0.67, 9.45) | 7.77 (-3.52, 20.38) |
| Yes | < 14.48 | -12.16 (-26.60, 5.12) | -1.20 (-15.54, 15.56) | -9.96 (-54.81, 79.37) | -6.15 (-17.37, 6.60) | -4.67 (-33.03, 35.71) |
| Yes | ≥ 14.48 | 46.89 (26.28, 70.86) | 34.04 (16.86, 53.75) | 32.22 (-28.91, 145.90) | 39.86 (25.19, 56.25) | 39.71 (5.72, 84.61) |
| RERI |  | 0.53 (0.26, 0.79) | 0.30 (0.07, 0.53) | 0.20 (-0.79, 1.20) | 0.41 (0.22, 0.60) | 0.37 (-0.13, 0.86) |
| Shenzhen |  | male | female | 18 | 18-64 | 65 |
| 10^th^ (2d) ^a^ |  |  |  |  |  |  |
| No | < 13.27 | 1.00 | 1.00 | 1.00 | 1.00 | 1.00 |
| No | ≥ 13.27 | -0.18 (-3.46, 3.22) | 3.08 (-0.10, 6.36) | -4.32 (-15.53, 8.39) | 1.29 (-1.14, 3.77) | 7.42 (-1.15, 16.74) |
| Yes | < 13.27 | 2.47 (-3.92, 9.30) | 2.40 (-3.64, 8.81) | -4.66 (-23.75, 19.21) | 1.53 (-3.13, 6.42) | 17.87 (0.30, 38.52) |
| Yes | ≥ 13.27 | 5.66 (0.09, 11.54) | 15.47 (9.73, 21.51) | 0.37 (-18.77, 24.02) | 10.92 (6.65, 15.36) | 12.81 (-1.64, 29.38) |
| RERI |  | 0.03 (-0.04, 0.11) | 0.10 (0.03, 0.17) | 0.09 (-0.17, 0.36) | 0.08 (0.02, 0.14) | -0.12 (-0.34, 0.09) |
| 10^th^ (3d) ^a^ |  |  |  |  |  |  |
| No | < 13.27 | 1.00 | 1.00 | 1.00 | 1.00 | 1.00 |
| No | ≥ 13.27 | -0.62 (-3.86, 2.72) | 3.12 (-0.02, 6.37) | -5.81 (-16.77, 6.59) | 1.24 (-1.15, 3.70) | 5.81 (-2.55, 14.89) |
| Yes | < 13.27 | -0.78 (-7.40, 6.31) | 2.22 (-4.14, 9.01) | -5.58 (-25.13, 19.09) | 0.14 (-4.75, 5.28) | 12.02 (-5.69, 33.06) |
| Yes | ≥ 13.27 | 6.21 (0.12, 12.68) | 14.35 (8.15, 20.91) | 0.65 (-20.62, 27.61) | 10.81 (6.16, 15.67) | 10.16 (-5.09, 27.87) |
| RERI |  | 0.08 (-0.01, 0.16) | 0.09 (0.01, 0.17) | 0.12 (-0.17, 0.41) | 0.009 (0.03, 0.16) | -0.08 (-0.31, 0.15) |
| 10^th^ (4d) ^a^ |  |  |  |  |  |  |
| No | < 13.27 | 1.00 | 1.00 | 1.00 | 1.00 | 1.00 |
| No | ≥ 13.27 | -1.85 (-5.03, 1.43) | 2.67 (-0.44, 5.88) | -6.39 (-17.24, 5.88) | 0.43 (-1.93, 2.85) | 4.65 (-3.55, 13.55) |
| Yes | < 13.27 | -7.26 (-13.81, -0.22) | -1.82 (-8.23, 5.03) | -5.57 (-25.80, 20.17) | -5.16 (-10.05, -0.01) | 4.53 (-12.57, 24.98) |
| Yes | ≥ 13.27 | 5.27 (-0.90, 11.82) | 15.82 (9.43, 22.59) | 5.61 (-17.08, 34.51) | 10.87 (6.13, 15.83) | 11.07 (-4.47, 29.15) |
| RERI |  | 0.14 (0.06, 0.23) | 0.15 (0.07, 0.23) | 0.18 (-0.13, 0.49) | 0.16 (0.09, 0.22) | 0.02 (-0.21, 0.25) |
| 7.5^th^ (2d) ^a^ |  |  |  |  |  |  |
| No | < 13.27 | 1.00 | 1.00 | 1.00 | 1.00 | 1.00 |
| No | ≥ 13.27 | -1.33 (-4.50, 1.94) | 3.04 (-0.06, 6.24) | -5.97 (-16.73, 6.18) | 0.81 (-1.55, 3.22) | 6.05 (-2.20, 15.01) |
| Yes | < 13.27 | -4.78 (-11.28, 2.19) | -0.98 (-7.30, 5.78) | -13.54 (-32.47, 10.69) | -3.36 (-8.18, 1.73) | 10.06 (-7.60, 31.09) |
| Yes | ≥ 13.27 | 6.21 (0.16, 12.62) | 13.88 (7.76, 20.34) | 5.20 (-16.50, 32.55) | 10.64 (6.04, 15.44) | 6.58 (-8.14, 23.66) |
| RERI |  | 0.12 (0.04, 0.21) | 0.12 (0.04, 0.20) | 0.25 (-0.04, 0.54) | 0.13 (0.07, 0.19) | -0.10 (-0.33, 0.14) |
| 7.5^th^ (3d) ^a^ |  |  |  |  |  |  |
| No | < 13.27 | 1.00 | 1.00 | 1.00 | 1.00 | 1.00 |
| No | ≥ 13.27 | -0.44 (-3.61, 2.82) | 3.36 (0.29, 6.53) | -5.19 (-15.94, 6.93) | 1.47 (-0.87, 3.87) | 5.70 (-2.46, 14.54) |
| Yes | < 13.27 | -0.72 (-8.16, 7.34) | 2.06 (-5.08, 9.74) | -4.25 (-26.58, 24.89) | -0.29 (-5.78, 5.51) | 15.22 (-4.67, 39.26) |
| Yes | ≥ 13.27 | 6.06 (-0.28, 12.81) | 11.29 (5.01, 17.95) | 1.22 (-20.62, 29.06) | 8.91 (4.15, 13.89) | 10.45 (-5.42, 28.98) |
| RERI |  | 0.07 (-0.02, 0.17) | 0.06 (-0.03, 0.15) | 0.11 (-0.22, 0.43) | 0.08 (0.01, 0.15) | -0.10 (-0.36, 0.15) |
| 7.5^th^ (4d) ^a^ |  |  |  |  |  |  |
| No | < 13.27 | 1.00 | 1.00 | 1.00 | 1.00 | 1.00 |
| No | ≥ 13.27 | -1.05 (-4.16, 2.15) | 3.10 (0.08, 6.21) | -4.93 (-15.55, 7.02) | 1.05 (-1.25, 3.41) | 4.81 (-3.15, 13.42) |
| Yes | < 13.27 | -6.54 (-15.50, 3.36) | -4.09 (-12.77, 5.44) | 3.41 (-26.29, 45.09) | -6.72 (-13.34, 0.42) | 7.14 (-16.08, 36.78) |
| Yes | ≥ 13.27 | 4.72 (-1.79, 11.65) | 10.38 (3.95, 17.22) | 7.30 (-16.46, 37.81) | 7.80 (2.92, 12.92) | 6.78 (-9.02, 25.33) |
| RERI |  | 0.12 (0.01, 0.23) | 0.11 (0.01, 0.22) | 0.09 (-0.33, 0.50) | 0.13 (0.05, 0.21) | -0.05 (-0.35, 0.24) |
| 5^th^ (2d) ^a^ |  |  |  |  |  |  |
| No | < 13.27 | 1.00 | 1.00 | 1.00 | 1.00 | 1.00 |
| No | ≥ 13.27 | -0.95 (-4.08, 2.27) | 3.62 (0.56, 6.78) | -5.63 (-16.22, 6.31) | 1.41 (-0.91, 3.79) | 5.09 (-2.95, 13.79) |
| Yes | < 13.27 | -5.88 (-13.37, 2.26) | -1.43 (-8.75, 6.48) | -16.70 (-38.23, 12.33) | -3.46 (-9.07, 2.50) | 1.53 (-17.36, 24.74) |
| Yes | ≥ 13.27 | 4.52 (-1.82, 11.26) | 8.58 (2.40, 15.13) | 8.05 (-15.15, 37.60) | 7.25 (2.51, 12.21) | -1.47 (-15.74, 15.21) |
| RERI |  | 0.11 (0.02, 0.21) | 0.06 (-0.02, 0.15) | 0.30 (-0.02, 0.63) | 0.09 (0.02, 0.16) | -0.08 (-0.32, 0.16) |
| 5^th^ (3d) ^a^ |  |  |  |  |  |  |
| No | < 13.27 | 1.00 | 1.00 | 1.00 | 1.00 | 1.00 |
| No | ≥ 13.27 | -1.12 (-4.21, 2.08) | 2.70 (-0.30, 5.79) | -6.04 (-16.50, 5.72) | 0.90 (-1.39, 3.25) | 4.00 (-3.87, 12.52) |
| Yes | < 13.27 | -13.37 (-21.69, -4.15) | -8.49 (-16.72, 0.55) | -17.03 (-41.31, 17.30) | -11.17 (-17.45, -4.40) | -3.33 (-24.43, 23.66) |
| Yes | ≥ 13.27 | -1.33 (-7.86, 5.67) | 5.08 (-1.39, 11.96) | -2.23 (-24.86, 27.20) | 2.69 (-2.25, 7.88) | -3.46 (-18.46, 14.29) |
| RERI |  | 0.13 (0.03, 0.23) | 0.11 (0.01, 0.21) | 0.21 (-0.15, 0.56) | 0.13 (0.05, 0.21) | -0.04 (-0.31, 0.23) |
| 5^th^ (4d) ^a^ |  |  |  |  |  |  |
| No | < 13.27 | 1.00 | 1.00 | 1.00 | 1.00 | 1.00 |
| No | ≥ 13.27 | -0.87 (-3.94, 2.29) | 3.30 (0.33, 6.37) | -5.42 (-15.78, 6.22) | 1.31 (-0.96, 3.64) | 4.76 (-3.07, 13.21) |
| Yes | < 13.27 | -13.98 (-26.74, 1.01) | -9.08 (-22.19, 6.24) | -17.23 (-56.68, 58.12) | -12.69 (-22.45, -1.70) | 8.28 (-27.06, 60.76) |
| Yes | ≥ 13.27 | 0.39 (-6.68, 7.99) | 7.11 (0.10, 14.60) | -0.61 (-24.81, 31.37) | 4.45 (-0.90, 10.09) | 0.15 (-16.22, 19.73) |
| RERI |  | 0.15 (0.01, 0.30) | 0.13 (-0.02, 0.28) | 0.22 (-0.35, 0.79) | 0.16 (0.05, 0.27) | -0.13 (-0.57, 0.31) |
| Zhaoqing |  | male | female | 18 | 18-64 | 65 |
| 10^th^ (2d) ^b^ |  |  |  |  |  |  |
| No | < 14.13 | 1.00 | 1.00 | 1.00 | 1.00 | 1.00 |
| No | ≥ 14.13 | -1.76 (-9.14, 6.22) | 0.91 (-6.47, 8.86) | -4.56 (-32.38, 34.68) | 1.33 (-4.61, 7.63) | -7.30 (-18.99, 6.08) |
| Yes | < 14.13 | -17.86 (-31.13, -2.02) | -9.92 (-23.77, 6.44) | -38.36 (-74.47, 48.78) | -12.56 (-23.43, -0.13) | -15.28 (-38.16, 16.06) |
| Yes | ≥ 14.13 | -5.18 (-19.03, 11.05) | 2.89 (-11.60, 19.77) | -19.02 (-62.33, 74.08) | 2.07 (-9.46, 15.08) | -12.77 (-34.48, 16.14) |
| RERI |  | 0.14 (-0.05, 0.34) | 0.12 (-0.08, 0.32) | 0.24 (-0.54, 1.02) | 0.13 (-0.02, 0.29) | 0.10 (-0.24, 0.44) |
| 10^th^ (3d) ^b^ |  |  |  |  |  |  |
| No | < 14.13 | 1.00 | 1.00 | 1.00 | 1.00 | 1.00 |
| No | ≥ 14.13 | -1.49 (-8.79, 6.39) | 3.14 (-4.27, 11.13) | -0.99 (-29.63, 39.30) | 2.69 (-3.24, 8.97) | -7.01 (-18.61, 6.26) |
| Yes | < 14.13 | -22.12 (-36.63, -4.28) | -0.20 (-17.46, 20.69) | -25.37 (-71.56, 95.88) | -10.16 (-22.96, 4.76) | -13.33 (-39.38, 23.91) |
| Yes | ≥ 14.13 | -7.51 (-21.39, 8.81) | 4.00 (-10.94, 21.44) | -28.20 (-68.78, 65.14) | 0.12 (-11.47, 13.21) | -6.21 (-29.85, 25.39) |
| RERI |  | 0.16 (-0.05, 0.37) | 0.01 (-0.22, 0.24) | -0.02 (-0.94, 0.90) | 0.08 (-0.10, 0.25) | 0.14 (-0.24, 0.52) |
| 10^th^ (4d) ^b^ |  |  |  |  |  |  |
| No | < 14.13 | 1.00 | 1.00 | 1.00 | 1.00 | 1.00 |
| No | ≥ 14.13 | -1.27 (-8.56, 6.61) | 1.33 (-5.93, 9.15) | 3.37 (-26.47, 45.31) | 1.86 (-4.00, 8.07) | -8.38 (-19.79, 4.66) |
| Yes | < 14.13 | -13.30 (-31.25, 9.35) | -2.30 (-20.74, 20.43) | 18.46 (-57.73, 232.03) | -7.26 (-21.90, 10.12) | -10.72 (-39.53, 31.82) |
| Yes | ≥ 14.13 | 3.02 (-13.25, 22.35) | 16.96 (-0.73, 37.80) | -24.25 (-69.79, 89.94) | 10.67 (-2.85, 26.07) | 13.07 (-16.42, 52.95) |
| RERI |  | 0.18 (-0.08, 0.43) | 0.18 (-0.08, 0.44) | -0.46 (-1.86, 0.94) | 0.16 (-0.04, 0.36) | 0.32 (-0.12, 0.76) |
| 7.5^th^ (2d) ^b^ |  |  |  |  |  |  |
| No | < 14.13 | 1.00 | 1.00 | 1.00 | 1.00 | 1.00 |
| No | ≥ 14.13 | -0.47 (-7.78, 7.42) | 1.66 (-5.58, 9.45) | 0.12 (-28.79, 40.76) | 2.09 (-3.75, 8.28) | -5.68 (-17.31, 7.57) |
| Yes | < 14.13 | -15.21 (-30.68, 3.71) | -5.69 (-21.96, 13.98) | -20.24 (-69.35, 107.59) | -10.26 (-22.92, 4.48) | -8.07 (-35.27, 30.56) |
| Yes | ≥ 14.13 | -5.41 (-20.46, 12.49) | 9.47 (-7.06, 28.94) | -34.19 (-72.89, 59.75) | 6.33 (-6.67, 21.14) | -11.97 (-35.31, 19.80) |
| RERI |  | 0.10 (-0.12, 0.33) | 0.14 (-0.11, 0.38) | -0.14 (-1.11, 0.83) | 0.15 (-0.04, 0.33) | 0.02 (-0.39, 0.43) |
| 7.5^th^ (3d) ^b^ |  |  |  |  |  |  |
| No | < 14.13 | 1.00 | 1.00 | 1.00 | 1.00 | 1.00 |
| No | ≥ 14.13 | 0.43 (-6.74, 8.14) | 1.25 (-5.73, 8.76) | 5.00 (-24.52, 46.06) | 2.45 (-3.23, 8.47) | -6.73 (-17.83, 5.86) |
| Yes | < 14.13 | -18.69 (-38.16, 6.91) | -21.56 (-39.60, 1.88) | 43.60 (-51.70, 326.94) | -19.67 (-34.69, -1.18) | -30.75 (-58.64, 15.95) |
| Yes | ≥ 14.13 | -7.89 (-23.56, 11.01) | 7.69 (-9.31, 27.89) | -37.54 (-76.58, 66.58) | 2.96 (-10.36, 18.26) | -7.92 (-33.42, 27.36) |
| RERI |  | 0.10 (-0.18, 0.38) | 0.28 (0.01, 0.55) | -0.86 (-2.57, 0.85) | 0.20 (-0.02, 0.42) | 0.30 (-0.17, 0.76) |
| 7.5^th^ (4d) ^b^ |  |  |  |  |  |  |
| No | < 14.13 | 1.00 | 1.00 | 1.00 | 1.00 | 1.00 |
| No | ≥ 14.13 | 0.09 (-7.02, 7.75) | 1.53 (-5.44, 9.02) | 4.80 (-24.49, 45.46) | 2.62 (-3.05, 8.62) | -7.66 (-18.61, 4.76) |
| Yes | < 14.13 | -15.59 (-37.74, 14.45) | -11.23 (-33.20, 17.96) | 79.62 (-48.99, 532.53) | -10.36 (-28.44, 12.28) | -38.49 (-66.54, 13.06) |
| Yes | ≥ 14.13 | -6.46 (-23.85, 14.92) | 15.43 (-4.30, 39.24) | -44.99 (-81.41, 62.79) | 7.56 (-7.68, 25.31) | -0.54 (-29.47, 40.26) |
| RERI |  | 0.09 (-0.23, 0.41) | 0.25 (-0.08, 0.58) | -1.29 (-3.68, 1.09) | 0.15 (-0.11, 0.41) | 0.46 (-0.05, 0.96) |
| 5^th^ (2d) ^b^ |  |  |  |  |  |  |
| No | < 14.13 | 1.00 | 1.00 | 1.00 | 1.00 | 1.00 |
| No | ≥ 14.13 | 0.74 (-6.46, 8.50) | 1.54 (-5.48, 9.08) | -3.11 (-30.55, 35.17) | 2.99 (-2.73, 9.04) | -6.50 (-17.71, 6.23) |
| Yes | < 14.13 | -19.47 (-39.79, 7.69) | -12.49 (-33.11, 14.50) | -51.10 (-87.36, 89.19) | -14.67 (-31.39, 6.13) | -14.67 (-47.95, 39.89) |
| Yes | ≥ 14.13 | -15.39 (-31.17, 4.01) | 12.60 (-6.18, 35.13) | -23.43 (-70.99, 102.11) | -0.40 (-14.37, 15.83) | 0.31 (-28.36, 40.46) |
| RERI |  | 0.03 (-0.26, 0.33) | 0.24 (-0.07, 0.54) | 0.31 (-0.69, 1.30) | 0.11 (-0.12, 0.35) | 0.21 (-0.32, 0.75) |
| 5^th^ (3d) ^b^ |  |  |  |  |  |  |
| No | < 14.13 | 1.00 | 1.00 | 1.00 | 1.00 | 1.00 |
| No | ≥ 14.13 | 0.85 (-6.24, 8.48) | 1.98 (-4.97, 9.44) | 0.10 (-27.79, 38.76) | 3.16 (-2.48, 9.13) | -6.06 (-17.16, 6.52) |
| Yes | < 14.13 | -30.65 (-52.68, 1.63) | -14.01 (-38.13, 19.52) | -30.65 (-85.37, 228.72) | -21.78 (-40.66, 3.09) | -19.36 (-56.71, 50.21) |
| Yes | ≥ 14.13 | -18.86 (-35.25, 1.67) | 15.92 (-4.77, 41.10) | -27.92 (-76.04, 116.84) | -0.55 (-15.60, 17.18) | -0.10 (-30.19, 42.98) |
| RERI |  | 0.11 (-0.22, 0.43) | 0.28 (-0.08, 0.64) | 0.03 (-1.32, 1.37) | 0.18 (-0.09, 0.45) | 0.25 (-0.36, 0.87) |
| 5^th^ (4d) ^b^ |  |  |  |  |  |  |
| No | < 14.13 | 1.00 | 1.00 | 1.00 | 1.00 | 1.00 |
| No | ≥ 14.13 | 0.30 (-6.74, 7.87) | 1.99 (-4.94, 9.43) | 0.85 (-27.22, 39.74) | 2.90 (-2.72, 8.84) | -6.53 (-17.55, 5.95) |
| Yes | < 14.13 | -26.70 (-50.72, 9.01) | -10.52 (-36.45, 25.99) | -11.01 (-81.95, 338.73) | -17.43 (-37.89, 9.76) | -21.60 (-60.13, 54.15) |
| Yes | ≥ 14.13 | -16.74 (-34.87, 6.44) | 14.95 (-7.29, 42.53) | -39.53 (-81.56, 98.31) | 0.23 (-16.31, 20.04) | 2.08 (-30.25, 49.41) |
| RERI |  | 0.10 (-0.26, 0.45) | 0.23 (-0.16, 0.63) | -0.29 (-1.91, 1.32) | 0.15 (-0.15, 0.44) | 0.30 (-0.35, 0.96) |

Note: SO_2_, sulfur dioxide.

^a^ Cold spells were defined by percentile temperature thresholds (10^th^, 7.5^th^, 5^th^ and 2.5^th^) and by the number of consecutive days below the thresholds (2-4 d).

^b^ Nine definitions [10^th^ (2d), 10^th^ (3d), 10^th^ (4d), 7.5^th^ (2d), 7.5^th^ (3d), 7.5^th^ (4d), 5^th^ (2d), 5^th^ (3d), and 5^th^ (4d)] were used in Zhaoqing.

^c^ Air pollutants were classified as binary variables using the median of air pollutant (lag03) concentrations as a cut off.

## Table S16 Independent effects of cold spells on anxiety when PM_2.5_, PM_10_, NO_2_ and SO_2_ at lag0 were included in each model separately.

|  | PM_2.5_ | PM_10_ | NO_2_ | SO_2_ |
| --- | --- | --- | --- | --- |
| Huizhou |  |  |  |  |
| 10^th^ (2d) ^a^ | 9.39 (4.17, 14.87) | 10.02 (4.74, 15.57) | 12.47 (7.07, 18.15) | 9.06 (3.92, 14.46) |
| 10^th^ (3d) ^a^ | 8.10 (2.62, 13.86) | 8.69 (3.15, 14.52) | 11.10 (5.44, 17.07) | 7.70 (2.30, 13.37) |
| 10^th^ (4d) ^a^ | 7.69 (1.71, 14.03) | 8.17 (2.13, 14.56) | 10.07 (3.94, 16.57) | 7.32 (1.40, 13.59) |
| 7.5^th^ (2d) ^a^ | 9.56 (3.82, 15.61) | 10.19 (4.39, 16.31) | 13.13 (7.15, 19.44) | 9.06 (3.42, 15.00) |
| 7.5^th^ (3d) ^a^ | 9.71 (3.67, 16.10) | 10.28 (4.18, 16.73) | 13.01 (6.75, 19.64) | 9.34 (3.38, 15.65) |
| 7.5^th^ (4d) ^a^ | 7.64 (0.85, 14.89) | 8.07 (1.24, 15.37) | 10.34 (3.35, 17.81) | 7.34 (0.60, 14.53) |
| 5^th^ (2d) ^a^ | 8.82 (2.54, 15.49) | 9.40 (3.05, 16.14) | 12.36 (5.80, 19.33) | 8.55 (2.32, 15.17) |
| 5^th^ (3d) ^a^ | 13.04 (5.77, 20.80) | 13.55 (6.23, 21.37) | 16.09 (8.58, 24.12) | 12.80 (5.57, 20.53) |
| 5^th^ (4d) ^a^ | 13.53 (5.26, 22.45) | 14.02 (5.70, 23.00) | 16.75 (8.19, 25.98) | 13.26 (5.02, 22.13) |
| 2.5^th^ (2d) ^a^ | 14.39 (5.99, 23.45) | 15.03 (6.55, 24.17) | 18.00 (9.29, 27.41) | 14.16 (5.80, 23.17) |
| 2.5^th^ (3d) ^a^ | 14.15 (4.73, 24.41) | 14.55 (5.09, 24.87) | 17.39 (7.65, 28.00) | 14.11 (4.70, 24.36) |
| 2.5^th^ (4d) ^a^ | 13.73 (3.34, 25.17) | 14.15 (3.71, 25.65) | 16.77 (6.05, 28.57) | 13.55 (3.18, 24.96) |
| Shenzhen |  |  |  |  |
| 10^th^ (2d) ^a^ | 6.68 (3.72, 9.72) | 7.15 (4.16, 10.22) | 8.84 (5.79, 11.98) | 6.76 (3.83, 9.77) |
| 10^th^ (3d) ^a^ | 5.70 (2.59, 8.91) | 6.14 (3.00, 9.38) | 8.01 (4.78, 11.34) | 5.70 (2.62, 8.88) |
| 10^th^ (4d) ^a^ | 4.54 (1.41, 7.77) | 4.93 (1.77, 8.18) | 6.56 (3.33, 9.89) | 4.61 (1.50, 7.82) |
| 7.5^th^ (2d) ^a^ | 4.45 (1.36, 7.64) | 4.79 (1.67, 8.00) | 7.36 (4.08, 10.74) | 4.64 (1.55, 7.83) |
| 7.5^th^ (3d) ^a^ | 4.71 (1.40, 8.12) | 4.99 (1.67, 8.42) | 7.65 (4.13, 11.29) | 4.72 (1.42, 8.13) |
| 7.5^th^ (4d) ^a^ | 3.46 (-0.13, 7.19) | 3.77 (0.15, 7.52) | 5.88 (2.12, 9.77) | 3.47 (-0.11, 7.18) |
| 5^th^ (2d) ^a^ | 2.05 (-1.28, 5.49) | 2.32 (-1.04, 5.78) | 4.97 (1.43, 8.64) | 2.11 (-1.22, 5.54) |
| 5^th^ (3d) ^a^ | -2.32 (-5.95, 1.45) | -2.05 (-5.70, 1.74) | 0.05 (-3.76, 4.02) | -2.23 (-5.85, 1.54) |
| 5^th^ (4d) ^a^ | 0.80 (-3.53, 5.33) | 1.12 (-3.23, 5.67) | 3.28 (-1.21, 7.99) | 0.83 (-3.48, 5.33) |
| 2.5^th^ (2d) ^a^ | 13.02 (8.28, 17.97) | 13.51 (8.74, 18.49) | 15.44 (10.56, 20.53) | 12.89 (8.18, 17.80) |
| 2.5^th^ (3d) ^a^ | 8.77 (3.51, 14.31) | 9.07 (3.79, 14.63) | 11.21 (5.77, 16.92) | 8.68 (3.44, 14.19) |
| 2.5^th^ (4d) ^a^ | 9.54 (3.92, 15.47) | 9.83 (4.19, 15.78) | 11.85 (6.07, 17.95) | 9.35 (3.76, 15.25) |
| Zhaoqing |  |  |  |  |
| 10^th^ (2d) ^b^ | -6.10 (-13.67, 2.13) | -6.25 (-13.87, 2.04) | -5.70 (-13.40, 2.68) | -6.82 (-14.20, 1.21) |
| 10^th^ (3d) ^b^ | -5.49 (-13.56, 3.33) | -5.58 (-13.70, 3.30) | -5.02 (-13.24, 3.97) | -6.18 (-14.12, 2.50) |
| 10^th^ (4d) ^b^ | 3.78 (-5.73, 14.25) | 3.78 (-5.78, 14.31) | 4.60 (-5.10, 15.28) | 3.01 (-6.39, 13.36) |
| 7.5^th^ (2d) ^b^ | -3.17 (-11.64, 6.10) | -3.22 (-11.74, 6.13) | -2.60 (-11.23, 6.87) | -3.96 (-12.29, 5.16) |
| 7.5^th^ (3d) ^b^ | -6.81 (-16.00, 3.39) | -6.82 (-16.04, 3.41) | -6.30 (-15.63, 4.06) | -7.31 (-16.43, 2.81) |
| 7.5^th^ (4d) ^b^ | -1.41 (-12.02, 10.47) | -1.46 (-12.09, 10.46) | -0.74 (-11.51, 11.33) | -2.04 (-12.56, 9.74) |
| 5^th^ (2d) ^b^ | -6.30 (-16.23, 4.80) | -6.34 (-16.30, 4.80) | -5.78 (-15.84, 5.49) | -6.97 (-16.80, 4.02) |
| 5^th^ (3d) ^b^ | -7.65 (-18.52, 4.67) | -7.65 (-18.55, 4.70) | -7.08 (-18.10, 5.42) | -8.14 (-18.95, 4.10) |
| 5^th^ (4d) ^b^ | -6.27 (-18.14, 7.33) | -6.36 (-18.24, 7.25) | -5.68 (-17.70, 8.10) | -6.88 (-18.64, 6.59) |

Note: PM, particulate matter; SO_2_, sulfur dioxide; NO_2_, nitrogen dioxide.

^a^ Cold spells were defined by percentile temperature thresholds (10^th^, 7.5^th^, 5^th^ and 2.5^th^) and by the number of consecutive days below the thresholds (2-4 d).

^b^ Nine definitions [10^th^ (2d), 10^th^ (3d), 10^th^ (4d), 7.5^th^ (2d), 7.5^th^ (3d), 7.5^th^ (4d), 5^th^ (2d), 5^th^ (3d), and 5^th^ (4d)] were used in Zhaoqing.

## Table S17. Independent effects of air pollution (lag0) on anxiety when cold spells under different definitions were controlled.

|  | PM_2.5_ ^c^ | PM_10_ ^c^ | NO_2_ ^c^ | SO_2_ ^c^ |
| --- | --- | --- | --- | --- |
| Huizhou |  |  |  |  |
| 10^th^ (2d) ^a^ | 0.64 (-0.07, 1.36) | 0.73 (0.18, 1.29) | 9.98 (6.98, 13.06) | 3.13 (0.49, 5.85) |
| 10^th^ (3d) ^a^ | 0.58 (-0.13, 1.30) | 0.68 (0.13, 1.23) | 9.65 (6.67, 12.72) | 2.96 (0.32, 5.67) |
| 10^th^ (4d) ^a^ | 0.53 (-0.18, 1.24) | 0.62 (0.07, 1.18) | 9.21 (6.26, 12.23) | 2.87 (0.23, 5.57) |
| 7.5^th^ (2d) ^a^ | 0.62 (-0.09, 1.34) | 0.71 (0.15, 1.27) | 9.94 (6.95, 13.03) | 3.01 (0.36, 5.72) |
| 7.5^th^ (3d) ^a^ | 0.59 (-0.13, 1.30) | 0.67 (0.12, 1.23) | 9.69 (6.71, 12.75) | 2.99 (0.35, 5.70) |
| 7.5^th^ (4d) ^a^ | 0.50 (-0.21, 1.21) | 0.59 (0.05, 1.14) | 9.10 (6.17, 12.12) | 2.85 (0.22, 5.55) |
| 5^th^ (2d) ^a^ | 0.53 (-0.18, 1.24) | 0.63 (0.09, 1.19) | 9.53 (6.57, 12.58) | 2.91 (0.27, 5.61) |
| 5^th^ (3d) ^a^ | 0.53 (-0.18, 1.24) | 0.63 (0.08, 1.18) | 9.38 (6.44, 12.40) | 2.91 (0.27, 5.61) |
| 5^th^ (4d) ^a^ | 0.50 (-0.20, 1.21) | 0.60 (0.06, 1.15) | 9.22 (6.30, 12.23) | 2.83 (0.20, 5.54) |
| 2.5^th^ (2d) ^a^ | 0.53 (-0.18, 1.24) | 0.63 (0.09, 1.18) | 9.37 (6.43, 12.39) | 2.94 (0.30, 5.64) |
| 2.5^th^ (3d) ^a^ | 0.46 (-0.24, 1.17) | 0.57 (0.02, 1.11) | 9.05 (6.13, 12.05) | 2.85 (0.21, 5.55) |
| 2.5^th^ (4d) ^a^ | 0.45 (-0.25, 1.16) | 0.56 (0.01, 1.10) | 8.91 (6.00, 11.90) | 2.79 (0.16, 5.49) |
| Shenzhen |  |  |  |  |
| 10^th^ (2d) ^a^ | 0.17 (-0.22, 0.56) | 0.37 (0.04, 0.69) | 6.73 (4.69, 8.81) | 1.80 (-0.14, 3.78) |
| 10^th^ (3d) ^a^ | 0.20 (-0.18, 0.60) | 0.37 (0.05, 0.70) | 6.38 (4.33, 8.47) | 1.68 (-0.28, 3.69) |
| 10^th^ (4d) ^a^ | 0.16 (-0.23, 0.55) | 0.33 (0.00, 0.65) | 6.13 (4.10, 8.19) | 1.57 (-0.40, 3.58) |
| 7.5^th^ (2d) ^a^ | 0.07 (-0.31, 0.46) | 0.28 (-0.04, 0.60) | 6.62 (4.56, 8.72) | 1.57 (-0.36, 3.55) |
| 7.5^th^ (3d) ^a^ | 0.13 (-0.26, 0.51) | 0.30 (-0.02, 0.62) | 6.32 (4.26, 8.42) | 1.40 (-0.55, 3.39) |
| 7.5^th^ (4d) ^a^ | 0.11 (-0.28, 0.50) | 0.28 (-0.04, 0.60) | 5.91 (3.89, 7.98) | 1.32 (-0.63, 3.32) |
| 5^th^ (2d) ^a^ | 0.03 (-0.36, 0.41) | 0.23 (-0.09, 0.55) | 6.11 (4.06, 8.19) | 1.31 (-0.61, 3.27) |
| 5^th^ (3d) ^a^ | 0.06 (-0.33, 0.44) | 0.22 (-0.10, 0.54) | 5.07 (3.05, 7.12) | 1.20 (-0.75, 3.19) |
| 5^th^ (4d) ^a^ | 0.08 (-0.31, 0.46) | 0.24 (-0.08, 0.57) | 5.49 (3.48, 7.53) | 1.25 (-0.71, 3.24) |
| 2.5^th^ (2d) ^a^ | 0.14 (-0.25, 0.52) | 0.33 (0.01, 0.65) | 6.39 (4.38, 8.43) | 1.39 (-0.54, 3.34) |
| 2.5^th^ (3d) ^a^ | 0.13 (-0.25, 0.52) | 0.29 (-0.03, 0.62) | 5.73 (3.74, 7.76) | 1.33 (-0.62, 3.31) |
| 2.5^th^ (4d) ^a^ | 0.12 (-0.27, 0.50) | 0.28 (-0.04, 0.60) | 5.77 (3.78, 7.80) | 1.17 (-0.78, 3.16) |
| Zhaoqing |  |  |  |  |
| 10^th^ (2d) ^b^ | 0.46 (-0.62, 1.56) | 0.27 (-0.68, 1.22) | 1.76 (-1.54, 5.18) | -0.53 (-6.28, 5.58) |
| 10^th^ (3d) ^b^ | 0.51 (-0.57, 1.60) | 0.30 (-0.64, 1.25) | 1.85 (-1.44, 5.25) | -0.68 (-6.44, 5.43) |
| 10^th^ (4d) ^b^ | 0.53 (-0.55, 1.62) | 0.35 (-0.59, 1.30) | 2.36 (-0.93, 5.76) | -1.28 (-7.01, 4.81) |
| 7.5^th^ (2d) ^b^ | 0.57 (-0.51, 1.66) | 0.37 (-0.57, 1.32) | 2.14 (-1.16, 5.55) | -0.41 (-6.18, 5.72) |
| 7.5^th^ (3d) ^b^ | 0.55 (-0.53, 1.63) | 0.34 (-0.59, 1.28) | 1.94 (-1.32, 5.30) | -0.58 (-6.34, 5.53) |
| 7.5^th^ (4d) ^b^ | 0.46 (-0.61, 1.55) | 0.28 (-0.66, 1.22) | 2.02 (-1.24, 5.39) | -1.50 (-7.21, 4.57) |
| 5^th^ (2d) ^b^ | 0.56 (-0.52, 1.64) | 0.36 (-0.57, 1.30) | 2.06 (-1.20, 5.42) | -0.46 (-6.22, 5.66) |
| 5^th^ (3d) ^b^ | 0.56 (-0.51, 1.64) | 0.36 (-0.57, 1.29) | 2.01 (-1.24, 5.36) | -0.53 (-6.29, 5.57) |
| 5^th^ (4d) ^b^ | 0.43 (-0.64, 1.51) | 0.24 (-0.69, 1.18) | 1.85 (-1.40, 5.20) | -1.58 (-7.29, 4.48) |

Note: PM, particulate matter; SO_2_, sulfur dioxide; NO_2_, nitrogen dioxide.

^a^ Cold spells were defined by percentile temperature thresholds (10^th^, 7.5^th^, 5^th^ and 2.5^th^) and by the number of consecutive days below the thresholds (2-4 d).

^b^ Nine definitions [10^th^ (2d), 10^th^ (3d), 10^th^ (4d), 7.5^th^ (2d), 7.5^th^ (3d), 7.5^th^ (4d), 5^th^ (2d), 5^th^ (3d), and 5^th^ (4d)] were used in Zhaoqing.

^c^ Air pollutants at lag0 were included in each model separately.

## Table S18. Percent changes and relative excess risk due to interaction (RERI) of cold spells and air pollution at lag0 exposure on anxiety in three subtropical cities in China.

| Cold spells | PM_2.5_ ^c^ (μg/m^3^) | Percent Change  (95% CI) | PM_10_ ^c^ (μg/m^3^) | Percent Change  (95% CI) | NO_2_ ^c^ (μg/m^3^) | Percent Change  (95% CI) | SO_2_ ^c^ (μg/m^3^) | Percent Change  (95% CI) |
| --- | --- | --- | --- | --- | --- | --- | --- | --- |
| Huizhou |  |  |  |  |  |  |  |  |
| 10^th^ (2d) ^a^ |  |  |  |  |  |  |  |  |
| No | < 45.91 | 1.00 | < 64.32 | 1.00 | < 16.54 | 1.00 | < 14.54 | 1.00 |
| No | ≥ 45.91 | 1.82 (-2.07, 5.85) | ≥ 64.32 | 3.62 (-0.23, 7.62) | ≥16.54 | **8.88 (4.31, 13.65)** | ≥ 14.54 | **4.53 (0.47, 8.75)** |
| Yes | < 45.91 | 2.70 (-4.35, 10.27) | < 64.32 | 1.27 (-5.89, 8.97) | < 16.54 | 7.35 (-0.65, 16.00) | < 14.54 | 3.32 (-4.87, 12.22) |
| Yes | ≥ 45.91 | **15.44 (8.15, 23.22)** | ≥64.32 | **18.60 (11.21, 26.48)** | ≥ 16.54 | **20.44 (12.72, 28.69)** | ≥ 14.54 | **15.66 (8.74, 23.02)** |
| RERI |  | **0.11 (0.02, 0.20)** |  | **0.14 (0.05, 0.23)** |  | 0.04 (-0.06, 0.14) |  | 0.08 (-0.02, 0.18) |
| 10^th^ (3d) ^a^ |  |  |  |  |  |  |  |  |
| No | < 45.91 | 1.00 | < 64.32 | 1.00 | < 16.54 | 1.00 | < 14.54 | 1.00 |
| No | ≥ 45.91 | 2.01 (-1.80, 5.97) | ≥ 64.32 | 3.73 (-0.05, 7.66) | ≥16.54 | **8.50 (3.97, 13.22)** | ≥ 14.54 | **4.58 (0.57, 8.76)** |
| Yes | < 45.91 | 1.68 (-5.85, 9.81) | < 64.32 | -0.36 (-8.08, 8.00) | < 16.54 | 4.90 (-3.81, 14.39) | < 14.54 | 1.66 (-7.33, 11.52) |
| Yes | ≥ 45.91 | **14.00 (6.47, 22.06)** | ≥64.32 | **16.74 (9.24, 24.75)** | ≥ 16.54 | **18.93 (11.09, 27.32)** | ≥ 14.54 | **14.03 (6.99, 21.54)** |
| RERI |  | **0.10 (0.01, 0.20)** |  | **0.13 (0.04, 0.23)** |  | 0.06 (-0.05, 0.16) |  | 0.08 (-0.03, 0.19) |
| 10^th^ (4d) ^a^ |  |  |  |  |  |  |  |  |
| No | < 45.91 | 1.00 | < 64.32 | 1.00 | < 16.54 | 1.00 | < 14.54 | 1.00 |
| No | ≥ 45.91 | 2.40 (-1.36, 6.30) | ≥ 64.32 | **4.12 (0.41, 7.98)** | ≥16.54 | **8.51 (4.04, 13.16)** | ≥ 14.54 | **4.71 (0.73, 8.85)** |
| Yes | < 45.91 | 2.17 (-6.26, 11.37) | < 64.32 | -0.61 (-9.24, 8.85) | < 16.54 | 3.51 (-6.30, 14.35) | < 14.54 | -0.88 (-10.29, 9.51) |
| Yes | ≥ 45.91 | **13.52 (5.37, 22.30)** | ≥64.32 | **16.51 (8.42, 25.19)** | ≥ 16.54 | **19.01 (10.57, 28.08)** | ≥ 14.54 | **15.64 (7.73, 24.13)** |
| RERI |  | 0.09 (-0.02, 0.20) |  | **0.13 (0.02, 0.24)** |  | 0.07 (-0.05, 0.19) |  | 0.12 (-0.01, 0.24) |
| 7.5^th^ (2d) ^a^ |  |  |  |  |  |  |  |  |
| No | < 45.91 | 1.00 | < 64.32 | 1.00 | < 16.54 | 1.00 | < 14.54 | 1.00 |
| No | ≥ 45.91 | 1.86 (-1.95, 5.82) | ≥ 64.32 | 3.72 (-0.06, 7.64) | ≥16.54 | **8.72 (4.22, 13.42)** | ≥ 14.54 | **4.21 (0.23, 8.35)** |
| Yes | < 45.91 | 1.87 (-5.68, 10.02) | < 64.32 | 0.41 (-7.41, 8.88) | < 16.54 | 6.40 (-2.47, 16.06) | < 14.54 | 0.33 (-8.79, 10.38) |
| Yes | ≥ 45.91 | **16.69 (8.70, 25.27)** | ≥64.32 | **19.07 (11.17, 27.54)** | ≥ 16.54 | **21.15 (12.84, 30.07)** | ≥ 14.54 | **16.17 (8.79, 24.04)** |
| RERI |  | **0.13 (0.03, 0.23)** |  | **0.15 (0.05, 0.25)** |  | 0.06 (-0.05, 0.17) |  | **0.12 (0.01, 0.23)** |
| 7.5^th^ (3d) ^a^ |  |  |  |  |  |  |  |  |
| No | < 45.91 | 1.00 | < 64.32 | 1.00 | < 16.54 | 1.00 | < 14.54 | 1.00 |
| No | ≥ 45.91 | 2.66 (-1.13, 6.58) | ≥ 64.32 | **4.36 (0.64, 8.22)** | ≥16.54 | **8.44 (3.98, 13.10)** | ≥ 14.54 | **4.81 (0.83, 8.95)** |
| Yes | < 45.91 | 5.16 (-3.09, 14.10) | < 64.32 | -2.66 (-10.72, 6.13) | < 16.54 | 5.35 (-4.29, 15.95) | < 14.54 | 3.63 (-6.29, 14.61) |
| Yes | ≥ 45.91 | **15.53 (7.19, 24.52)** | ≥64.32 | **24.16 (15.35, 33.64)** | ≥ 16.54 | **21.28 (12.72, 30.49)** | ≥ 14.54 | **16.01 (8.21, 24.36)** |
| RERI |  | 0.08 (-0.03, 0.19) |  | **0.22 (0.11, 0.34)** |  | 0.07 (-0.05, 0.20) |  | 0.08 (-0.05, 0.20) |
| 7.5^th^ (4d) ^a^ |  |  |  | lag0 |  |  |  |  |
| No | < 45.91 | 1.00 | < 64.32 | 1.00 | < 16.54 | 1.00 | < 14.54 | 1.00 |
| No | ≥ 45.91 | 2.80 (-0.92, 6.66) | ≥ 64.32 | **4.45 (0.77, 8.26)** | ≥16.54 | **8.21 (3.82, 12.79)** | ≥ 14.54 | **4.82 (0.87, 8.93)** |
| Yes | < 45.91 | 4.03 (-5.43, 14.44) | < 64.32 | -0.08 (-9.94, 10.85) | < 16.54 | 0.49 (-10.48, 12.80) | < 14.54 | -2.01 (-12.45, 9.66) |
| Yes | ≥ 45.91 | **13.11 (3.80, 23.25)** | ≥64.32 | **16.59 (7.49, 26.45)** | ≥ 16.54 | **20.27 (10.79, 30.56)** | ≥ 14.54 | **16.88 (7.80, 26.73)** |
| RERI |  | 0.06 (-0.07, 0.19) |  | 0.12 (-0.01, 0.25) |  | 0.12 (-0.03, 0.26) |  | **0.14 (0.01, 0.28)** |
| 5^th^ (2d) ^a^ |  |  |  |  |  |  |  |  |
| No | < 45.91 | 1.00 | < 64.32 | 1.00 | < 16.54 | 1.00 | < 14.54 | 1.00 |
| No | ≥ 45.91 | 1.91 (-1.82, 5.78) | ≥ 64.32 | 3.65 (-0.06, 7.49) | ≥16.54 | **7.56 (3.17, 12.15)** | ≥ 14.54 | **4.37 (0.39, 8.51)** |
| Yes | < 45.91 | 0.60 (-7.52, 9.44) | < 64.32 | -2.02 (-10.40, 7.14) | < 16.54 | -0.36 (-9.33, 9.49) | < 14.54 | 0.33 (-9.27, 10.95) |
| Yes | ≥ 45.91 | **18.49 (9.21, 28.56)** | ≥64.32 | **21.40 (12.30, 31.23)** | ≥ 16.54 | **24.86 (15.26, 35.27)** | ≥ 14.54 | **16.67 (8.37, 25.61)** |
| RERI |  | **0.16 (0.04, 0.28)** |  | **0.20 (0.08, 0.32)** |  | **0.18 (0.05, 0.30)** |  | 0.12 (-0.01, 0.25) |
| 5^th^ (3d) ^a^ |  |  |  |  |  |  |  |  |
| No | < 45.91 | 1.00 | < 64.32 | 1.00 | < 16.54 | 1.00 | < 14.54 | 1.00 |
| No | ≥ 45.91 | 2.86 (-0.85, 6.71) | ≥ 64.32 | **4.69 (1.02, 8.51)** | ≥16.54 | **7.68 (3.32, 12.22)** | ≥ 14.54 | **4.74 (0.79, 8.85)** |
| Yes | < 45.91 | 8.77 (-1.09, 19.62) | < 64.32 | 6.27 (-4.00, 17.64) | < 16.54 | 1.19 (-9.91, 13.67) | < 14.54 | 4.82 (-6.19, 17.13) |
| Yes | ≥ 45.91 | **19.96 (9.51, 31.40)** | ≥64.32 | **23.30 (13.08, 34.45)** | ≥ 16.54 | **28.75 (18.29, 40.13)** | ≥ 14.54 | **22.40 (12.46, 33.22)** |
| RERI |  | 0.08 (-0.06, 0.22) |  | 0.12 (-0.02, 0.27) |  | **0.20 (0.05, 0.35)** |  | 0.13 (-0.02, 0.28) |
| 5^th^ (4d) ^a^ |  |  |  |  |  |  |  |  |
| No | < 45.91 | 1.00 | < 64.32 | 1.00 | < 16.54 | 1.00 | < 14.54 | 1.00 |
| No | ≥ 45.91 | 2.52 (-1.16, 6.34) | ≥ 64.32 | **4.25 (0.61, 8.02)** | ≥16.54 | **7.38 (3.05, 11.88)** | ≥ 14.54 | **4.50 (0.58, 8.58)** |
| Yes | < 45.91 | 4.84 (-5.88, 16.78) | < 64.32 | 0.15 (-10.92, 12.59) | < 16.54 | -4.09 (-15.64, 9.04) | < 14.54 | -1.30 (-13.07, 12.06) |
| Yes | ≥ 45.91 | **24.93 (12.69, 38.50)** | ≥64.32 | **28.52 (16.65, 41.59)** | ≥ 16.54 | **34.70 (22.33, 48.33)** | ≥ 14.54 | **27.44 (15.81, 40.23)** |
| RERI |  | **0.18 (0.01, 0.34)** |  | **0.24 (0.08, 0.40)** |  | **0.31 (0.14, 0.48)** |  | **0.24 (0.07, 0.41)** |
| 2.5^th^ (2d) ^a^ |  |  |  |  |  |  |  |  |
| No | < 45.91 | 1.00 | < 64.32 | 1.00 | < 16.54 | 1.00 | < 14.54 | 1.00 |
| No | ≥ 45.91 | 2.97 (-0.74, 6.82) | ≥ 64.32 | **4.84 (1.16, 8.66)** | ≥16.54 | **8.19 (3.83, 12.74)** | ≥ 14.54 | **5.01 (1.09, 9.08)** |
| Yes | < 45.91 | 11.08 (-0.01, 23.41) | < 64.32 | 8.63 (-2.89, 21.53) | < 16.54 | 4.25 (-6.95, 16.81) | < 14.54 | 5.49 (-6.56, 19.10) |
| Yes | ≥ 45.91 | **21.00 (8.66, 34.74)** | ≥64.32 | **24.91 (12.86, 38.24)** | ≥ 16.54 | **37.17 (23.39, 52.49)** | ≥ 14.54 | **25.96 (13.94, 39.25)** |
| RERI |  | 0.07 (-0.10, 0.24) |  | 0.11 (-0.05, 0.28) |  | **0.25 (0.07, 0.43)** |  | 0.15 (-0.02, 0.33) |
| 2.5^th^ (3d) ^a^ |  |  |  |  |  |  |  |  |
| No | < 45.91 | 1.00 | < 64.32 | 1.00 | < 16.54 | 1.00 | < 14.54 | 1.00 |
| No | ≥ 45.91 | 3.33 (-0.35, 7.14) | ≥ 64.32 | **5.15 (1.51, 8.91)** | ≥16.54 | **7.71 (3.43, 12.18)** | ≥ 14.54 | **5.50 (1.59, 9.56)** |
| Yes | < 45.91 | **21.25 (5.98, 38.73)** | < 64.32 | **17.26 (0.72, 36.52)** | < 16.54 | -2.27 (-14.99, 12.34) | < 14.54 | 11.36 (-2.84, 27.63) |
| Yes | ≥ 45.91 | **12.60 (0.42, 26.25)** | ≥64.32 | **17.47 (5.59, 30.69)** | ≥ 16.54 | **38.60 (23.64, 55.37)** | ≥ 14.54 | **21.80 (8.71, 36.46)** |
| RERI |  | -0.12 (-0.33, 0.09) |  | -0.05 (-0.27, 0.17) |  | **0.33 (0.13, 0.54)** |  | 0.05 (-0.16, 0.25) |
| 2.5^th^ (4d) ^a^ |  |  |  |  |  |  |  |  |
| No | < 45.91 | 1.00 | < 64.32 | 1.00 | < 16.54 | 1.00 | < 14.54 | 1.00 |
| No | ≥ 45.91 | 3.38 (-0.29, 7.19) | ≥ 64.32 | **5.13 (1.50, 8.89)** | ≥16.54 | **8.35 (4.07, 12.81)** | ≥ 14.54 | **5.41 (1.51, 9.46)** |
| Yes | < 45.91 | **20.19 (3.77, 39.21)** | < 64.32 | 14.32 (-3.68, 35.68) | < 16.54 | 0.54 (-13.87, 17.36) | < 14.54 | 5.53 (-10.41, 24.30) |
| Yes | ≥ 45.91 | 12.58 (-1.04, 28.07) | ≥ 64.32 | **18.39 (5.23, 33.19)** | ≥ 16.54 | **35.79 (19.67, 54.09)** | ≥ 14.54 | **23.66 (9.61, 39.52)** |
| RERI |  | -0.11 (-0.34, 0.12) |  | -0.01 (-0.25, 0.23) |  | **0.27 (0.04, 0.50)** |  | 0.13 (-0.10, 0.36) |
| Shenzhen |  |  |  |  |  |  |  |  |
| 10^th^ (2d) ^a^ |  |  |  |  |  |  |  |  |
| No | < 39.67 | 1.00 | < 58.60 | 1.00 | < 14.59 | 1.00 | < 13.33 | 1.00 |
| No | ≥ 39.67 | -1.19 (-3.18, 0.85) | ≥ 58.60 | 0.53 (-1.51, 2.61) | ≥ 14.59 | **9.12 (6.37, 11.93)** | ≥ 13.33 | 1.52 (-0.64, 3.73) |
| Yes | < 39.67 | 2.24 (-1.45, 6.07) | < 58.60 | 2.76 (-1.02, 6.68) | < 14.59 | **9.11 (4.66, 13.75)** | < 13.33 | 1.05 (-3.46, 5.78) |
| Yes | ≥ 39.67 | **8.96 (5.04, 13.03)** | ≥ 58.60 | **10.85 (6.77, 15.09)** | ≥ 14.59 | **17.41 (12.90, 22.10)** | ≥ 13.33 | **10.10 (6.45, 13.87)** |
| RERI |  | **0.08 (0.03, 0.13)** |  | **0.08 (0.03, 0.12)** |  | -0.01 (-0.06, 0.04) |  | **0.08 (0.02, 0.13)** |
| 10^th^ (3d) ^a^ |  |  |  |  |  |  |  |  |
| No | < 39.67 | 1.00 | < 58.60 | 1.00 | < 14.59 | 1.00 | < 13.33 | 1.00 |
| No | ≥ 39.67 | -0.53 (-2.48, 1.47) | ≥ 58.60 | 0.72 (-1.28, 2.77) | ≥ 14.59 | **8.13 (5.40, 10.93)** | ≥ 13.33 | 0.84 (-1.28, 3.00) |
| Yes | < 39.67 | 3.25 (-0.87, 7.54) | < 58.60 | 3.69 (-0.56, 8.11) | < 14.59 | **9.00 (3.88, 14.36)** | < 13.33 | -0.65 (-5.47, 4.43) |
| Yes | ≥ 39.67 | **6.43 (2.49, 10.51)** | ≥ 58.60 | **7.59 (3.55, 11.79)** | ≥ 14.59 | **14.80 (10.22, 19.58)** | ≥ 13.33 | **8.82 (4.98, 12.80)** |
| RERI |  | 0.04 (-0.01, 0.09) |  | 0.03 (-0.02, 0.08) |  | -0.02 (-0.08, 0.03) |  | **0.09 (0.03, 0.14)** |
| 10^th^ (4d) ^a^ |  |  |  |  |  |  |  |  |
| No | < 39.67 | 1.00 | < 58.60 | 1.00 | < 14.59 | 1.00 | < 13.33 | 1.00 |
| No | ≥ 39.67 | -1.12 (-3.04, 0.85) | ≥ 58.60 | 0.28 (-1.71, 2.30) | ≥ 14.59 | **7.92 (5.24, 10.68)** | ≥ 13.33 | 1.26 (-0.86, 3.43) |
| Yes | < 39.67 | 1.28 (-2.87, 5.61) | < 58.60 | 1.64 (-2.64, 6.11) | < 14.59 | **6.60 (1.55, 11.90)** | < 13.33 | -2.86 (-7.74, 2.28) |
| Yes | ≥ 39.67 | **5.45 (1.41, 9.65)** | ≥ 58.60 | **6.74 (2.60, 11.04)** | ≥ 14.59 | **13.97 (9.34, 18.81)** | ≥ 13.33 | **8.81 (4.85, 12.93)** |
| RERI |  | **0.05 (0.01, 0.11)** |  | 0.05 (-0.01, 0.10) |  | -0.01 (-0.06, 0.05) |  | **0.10 (0.04, 0.16)** |
| 7.5^th^ (2d) ^a^ |  |  |  |  |  |  |  |  |
| No | < 39.67 | 1.00 | < 58.60 | 1.00 | < 14.59 | 1.00 | < 13.33 | 1.00 |
| No | ≥ 39.67 | -1.18 (-3.10, 0.78) | ≥ 58.60 | 0.30 (-1.67, 2.31) | ≥ 14.59 | **7.81 (5.18, 10.51)** | ≥ 13.33 | 1.53 (-0.58, 3.69) |
| Yes | < 39.67 | -0.85 (-5.08, 3.57) | < 58.60 | -0.88 (-5.23, 3.66) | < 14.59 | 3.63 (-1.25, 8.76) | < 13.33 | -3.43 (-8.45, 1.87) |
| Yes | ≥ 39.67 | **6.45 (2.55, 10.49)** | ≥ 58.60 | **8.01 (4.00, 12.18)** | ≥ 14.59 | **15.38 (10.72, 20.24)** | ≥ 13.33 | **8.64 (4.82, 12.60)** |
| RERI |  | **0.08 (0.03, 0.14)** |  | **0.09 (0.03, 0.14)** |  | 0.04 (-0.02, 0.10) |  | **0.11 (0.05, 0.16)** |
| 7.5^th^ (3d) ^a^ |  |  |  |  |  |  |  |  |
| No | < 39.67 | 1.00 | < 58.60 | 1.00 | < 14.59 | 1.00 | < 13.33 | 1.00 |
| No | ≥ 39.67 | -0.29 (-2.21, 1.65) | ≥ 58.60 | 0.85 (-1.11, 2.85) | ≥ 14.59 | **7.94 (5.32, 10.63)** | ≥ 13.33 | 1.22 (-0.87, 3.35) |
| Yes | < 39.67 | 4.03 (-0.87, 9.18) | < 58.60 | 4.32 (-0.76, 9.66) | < 14.59 | **9.40 (3.73, 15.40)** | < 13.33 | -1.48 (-7.60, 5.04) |
| Yes | ≥ 39.67 | **4.63 (0.63, 8.80)** | ≥ 58.60 | **5.66 (1.59, 9.89)** | ≥ 14.59 | **12.86 (8.08, 17.86)** | ≥ 13.33 | **7.42 (3.46, 11.53)** |
| RERI |  | 0.01 (-0.05, 0.07) |  | 0.01 (-0.05, 0.06) |  | -0.04 (-0.11, 0.02) |  | **0.08 (0.01, 0.15)** |
| 7.5^th^ (4d) ^a^ |  |  |  |  |  |  |  |  |
| No | < 39.67 | 1.00 | < 58.60 | 1.00 | < 14.59 | 1.00 | < 13.33 | 1.00 |
| No | ≥ 39.67 | -0.82 (-2.70, 1.10) | ≥ 58.60 | 0.45 (-1.48, 2.42) | ≥ 14.59 | **7.74 (5.15, 10.38)** | ≥ 13.33 | 1.66 (-0.42, 3.79) |
| Yes | < 39.67 | 1.33 (-3.79, 6.72) | < 58.60 | 1.42 (-3.88, 7.02) | < 14.59 | **6.80 (0.92, 13.02)** | < 13.33 | -5.74 (-12.17, 1.16) |
| Yes | ≥ 39.67 | 3.80 (-0.68, 8.49) | ≥ 58.60 | **4.96 (0.45, 9.67)** | ≥ 14.59 | **11.34 (6.28, 16.64)** | ≥ 13.33 | **7.72 (3.34, 12.30)** |
| RERI |  | 0.03 (-0.03, 0.10) | . | **0.03 (-0.03, 0.10)** |  | -0.03 (-0.10, 0.04) |  | **0.12 (0.04, 0.19)** |
| 5^th^ (2d) ^a^ |  |  |  | lag0 |  |  |  | lag0 |
| No | < 39.67 | 1.00 | < 58.60 | 1.00 | < 14.59 | 1.00 | < 13.33 | 1.00 |
| No | ≥ 39.67 | -1.32 (-3.19, 0.59) | ≥ 58.60 | -0.03 (-1.95, 1.92) | ≥ 14.59 | **7.03 (4.49, 9.63)** | ≥ 13.33 | 1.95 (-0.14, 4.08) |
| Yes | < 39.67 | **-7.31 (-12.12, -2.25)** | < 58.60 | **-8.35 (-13.30, -3.11)** | < 14.59 | -2.17 (-7.42, 3.39) | < 13.33 | **-7.58 (-14.14, -0.51)** |
| Yes | ≥ 39.67 | **5.45 (1.30, 9.76)** | ≥ 58.60 | **6.70 (2.50, 11.08)** | ≥ 14.59 | **13.18 (8.31, 18.26)** | ≥ 13.33 | **5.54 (1.65, 9.58)** |
| RERI |  | **0.14 (0.08, 0.20)** |  | **0.15 (0.09, 0.21)** |  | **0.08 (0.02, 0.15)** |  | **0.11 (0.04, 0.19)** |
| 5^th^ (3d) ^a^ |  | lag0 |  |  |  |  |  |  |
| No | < 39.67 | 1.00 | < 58.60 | 1.00 | < 14.59 | 1.00 | < 13.33 | 1.00 |
| No | ≥ 39.67 | -0.64 (-2.51, 1.26) | ≥ 58.60 | 0.22 (-1.70, 2.18) | ≥ 14.59 | **5.81 (3.29, 8.40)** | ≥ 13.33 | 1.47 (-0.59, 3.58) |
| Yes | < 39.67 | **-7.74 (-13.11, -2.04)** | < 58.60 | **-8.88 (-14.45, -2.94)** | < 14.59 | **-6.59 (-12.46, -0.33)** | < 13.33 | **-11.32 (-18.53, -3.47)** |
| Yes | ≥ 39.67 | -0.29 (-4.73, 4.35) | ≥ 58.60 | 0.68 (-3.76, 5.33) | ≥ 14.59 | **6.51 (1.55, 11.72)** | ≥ 13.33 | 0.75 (-3.46, 5.14) |
| RERI |  | **0.08 (0.02, 0.15)** |  | **0.09 (0.03, 0.16)** |  | **0.07 (0.01, 0.14)** |  | **0.11 (0.02, 0.19)** |
| 5^th^ (4d) ^a^ |  |  |  |  |  |  |  |  |
| No | < 39.67 | 1.00 | < 58.60 | 1.00 | < 14.59 | 1.00 | < 13.33 | 1.00 |
| No | ≥ 39.67 | -0.92 (-2.78, 0.97) | ≥ 58.60 | 0.26 (-1.64, 2.20) | ≥ 14.59 | **6.69 (4.16, 9.28)** | ≥ 13.33 | **2.11 (0.03, 4.23)** |
| Yes | < 39.67 | -4.63 (-11.14, 2.36) | < 58.60 | -5.74 (-12.61, 1.67) | < 14.59 | -1.59 (-8.61, 5.97) | < 13.33 | -8.45 (-18.32, 2.62) |
| Yes | ≥ 39.67 | 2.28 (-2.95, 7.79) | ≥ 58.60 | 3.46 (-1.71, 8.91) | ≥ 14.59 | **10.10 (4.32, 16.21)** | ≥ 13.33 | 3.68 (-1.14, 8.74) |
| RERI |  | 0.08 (-0.01, 0.16) |  | **0.09 (0.01, 0.17)** |  | 0.05 (-0.04, 0.14) |  | 0.10 (-0.01, 0.21) |
| 2.5^th^ (2d) ^a^ |  |  |  |  |  |  |  |  |
| No | < 39.67 | 1.00 | < 58.60 | 1.00 | < 14.59 | 1.00 | < 13.33 | 1.00 |
| No | ≥ 39.67 | -0.50 (-2.36, 1.40) | ≥ 58.60 | 0.52 (-1.38, 2.45) | ≥ 14.59 | **7.54 (5.00, 10.13)** | ≥ 13.33 | **2.08 (0.03, 4.18)** |
| Yes | < 39.67 | 2.15 (-4.46, 9.22) | < 58.60 | -1.91 (-8.38, 5.03) | < 14.59 | **7.19 (0.28, 14.56)** | < 13.33 | 2.30 (-6.64, 12.09) |
| Yes | ≥ 39.67 | **19.89 (13.40, 26.75)** | ≥ 58.60 | **23.48 (17.01, 30.31)** | ≥ 14.59 | **28.75 (21.62, 36.31)** | ≥ 13.33 | **17.82 (12.22, 23.69)** |
| RERI |  | **0.18 (0.09, 0.27)** |  | **0.25 (0.16, 0.34)** |  | **0.14 (0.04, 0.24)** |  | **0.13 (0.03, 0.24)** |
| 2.5^th^ (3d) ^a^ |  |  |  |  |  |  |  |  |
| No | < 39.67 | 1.00 | < 58.60 | 1.00 | < 14.59 | 1.00 | < 13.33 | 1.00 |
| No | ≥ 39.67 | -0.48 (-2.33, 1.40) | ≥ 58.60 | 0.38 (-1.50, 2.30) | ≥ 14.59 | **6.55 (4.06, 9.10)** | ≥ 13.33 | 1.71 (-0.31, 3.78) |
| Yes | < 39.67 | -2.07 (-10.12, 6.70) | < 58.60 | -8.62 (-17.34, 1.02) | < 14.59 | 4.34 (-4.27, 13.74) | < 13.33 | 1.60 (-11.42, 16.54) |
| Yes | ≥ 39.67 | **13.89 (7.07, 21.14)** | ≥ 58.60 | **15.37 (8.84, 22.29)** | ≥ 14.59 | **19.54 (12.27, 27.28)** | ≥ 13.33 | **11.20 (5.33, 17.39)** |
| RERI |  | **0.16 (0.06, 0.27)** |  | **0.24 (0.12, 0.35)** |  | 0.09 (-0.03, 0.20) |  | 0.08 (-0.07, 0.23) |
| 2.5^th^ (4d) ^a^ |  |  |  |  |  |  |  |  |
| No | < 39.67 | 1.00 | < 58.60 | 1.00 | < 14.59 | 1.00 | < 13.33 | 1.00 |
| No | ≥ 39.67 | -0.93 (-2.77, 0.95) | ≥ 58.60 | 0.17 (-1.71, 2.09) | ≥ 14.59 | **6.84 (4.36, 9.37)** | ≥ 13.33 | 2.03 (-0.02, 4.13) |
| Yes | < 39.67 | -2.26 (-10.76, 7.05) | < 58.60 | -9.09 (-18.45, 1.35) | < 14.59 | 4.61 (-4.57, 14.69) | < 13.33 | -3.99 (-22.19, 18.47) |
| Yes | ≥ 39.67 | **14.86 (7.54, 22.66)** | ≥ 58.60 | **16.28 (9.33, 23.67)** | ≥ 14.59 | **20.70 (12.96, 28.98)** | ≥ 13.33 | **11.93 (5.89, 18.32)** |
| RERI |  | **0.18 (0.07, 0.30)** |  | **0.25 (0.13, 0.37)** |  | 0.09 (-0.03, 0.22) |  | 0.14 (-0.07, 0.35) |
| Zhaoqing |  |  |  |  |  |  |  |  |
| 10^th^ (2d) ^b^ |  |  |  |  |  |  |  |  |
| No | < 43.89 | 1.00 | < 57.99 | 1.00 | < 13.25 | 1.00 | < 14.03 | 1.00 |
| No | ≥ 43.89 | 0.32 (-4.58, 5.47) | ≥ 57.99 | 1.87 (-3.10, 7.09) | ≥ 13.25 | 2.79 (-2.42, 8.28) | ≥ 14.03 | -3.45 (-8.54, 1.92) |
| Yes | < 43.89 | -9.25 (-18.36, 0.88) | < 57.99 | -7.73 (-16.75, 2.27) | < 13.25 | -8.59 (-17.62, 1.44) | < 14.03 | **-14.22 (-24.92, -2.00)** |
| Yes | ≥ 43.89 | -2.55 (-13.60, 9.91) | ≥ 57.99 | -1.44 (-13.11, 11.79) | ≥ 13.25 | 2.14 (-10.46, 16.52) | ≥ 14.03 | -6.37 (-15.28, 3.48) |
| RERI |  | 0.06 (-0.07, 0.20) |  | 0.04 (-0.10, 0.19) |  | 0.08 (-0.07, 0.23) |  | 0.11 (-0.02, 0.25) |
| 10^th^ (3d) ^b^ |  |  |  |  |  |  |  |  |
| No | < 43.89 | 1.00 | < 57.99 | 1.00 | < 13.25 | 1.00 | < 14.03 | 1.00 |
| No | ≥ 43.89 | 0.55 (-4.28, 5.62) | ≥ 57.99 | 2.10 (-2.79, 7.25) | ≥ 13.25 | 2.24 (-2.90, 7.66) | ≥ 14.03 | -3.36 (-8.41, 1.97) |
| Yes | < 43.89 | -9.09 (-19.09, 2.15) | < 57.99 | -7.56 (-17.36, 3.41) | < 13.25 | **-11.07 (-20.60, -0.40)** | < 14.03 | -13.35 (-25.23, 0.42) |
| Yes | ≥ 43.89 | -1.50 (-13.12, 11.66) | ≥ 57.99 | -0.05 (-12.42, 14.06) | ≥ 13.25 | 7.32 (-6.51, 23.20) | ≥ 14.03 | -5.96 (-15.46, 4.61) |
| RERI |  | 0.07 (-0.08, 0.22) |  | 0.05 (-0.10, 0.21) |  | 0.16 (-0.01, 0.33) |  | 0.11 (-0.04, 0.26) |
| 10^th^ (4d) ^b^ |  |  |  |  |  |  |  |  |
| No | < 43.89 | 1.00 | < 57.99 | 1.00 | < 13.25 | 1.00 | < 14.03 | 1.00 |
| No | ≥ 43.89 | 0.93 (-3.84, 5.95) | ≥ 57.99 | 2.08 (-2.75, 7.15) | ≥ 13.25 | 3.75 (-1.43, 9.19) | ≥ 14.03 | -4.12 (-9.12, 1.15) |
| Yes | < 43.89 | -0.08 (-12.19, 13.71) | < 57.99 | 0.35 (-11.25, 13.48) | < 13.25 | 2.32 (-9.59, 15.80) | < 14.03 | -7.21 (-20.77, 8.68) |
| Yes | ≥ 43.89 | 8.25 (-5.36, 23.82) | ≥ 57.99 | 11.26 (-3.44, 28.20) | ≥ 13.25 | 11.36 (-3.63, 28.67) | ≥ 14.03 | 3.93 (-7.36, 16.60) |
| RERI |  | 0.07 (-0.11, 0.26) |  | 0.09 (-0.10, 0.27) |  | 0.05 (-0.14, 0.25) |  | 0.15 (-0.02, 0.33) |
| 7.5^th^ (2d) ^b^ |  | lag0 |  |  |  |  |  |  |
| No | < 43.89 | 1.00 | < 57.99 | 1.00 | < 13.25 | 1.00 | < 14.03 | 1.00 |
| No | ≥ 43.89 | 0.66 (-4.15, 5.72) | ≥ 57.99 | 2.03 (-2.84, 7.14) | ≥ 13.25 | 3.93 (-1.23, 9.36) | ≥ 14.03 | -3.17 (-8.21, 2.14) |
| Yes | < 43.89 | -7.34 (-17.56, 4.14) | < 57.99 | -6.39 (-16.29, 4.69) | < 13.25 | -3.52 (-13.80, 7.98) | < 14.03 | -11.30 (-23.39, 2.70) |
| Yes | ≥ 43.89 | 2.42 (-10.51, 17.21) | ≥ 57.99 | 5.36 (-8.74, 21.63) | ≥ 13.25 | 3.14 (-11.37, 20.03) | ≥ 14.03 | -3.15 (-13.42, 8.35) |
| RERI |  | 0.09 (-0.07, 0.26) |  | 0.10 (-0.07, 0.27) |  | 0.03 (-0.15, 0.21) |  | 0.11 (-0.05, 0.27) |
| 7.5^th^ (3d) ^b^ |  |  |  | lag0 |  |  |  |  |
| No | < 43.89 | 1.00 | < 57.99 | 1.00 | < 13.25 | 1.00 | < 14.03 | 1.00 |
| No | ≥ 43.89 | 0.77 (-3.97, 5.75) | ≥ 57.99 | 2.20 (-2.57, 7.20) | ≥ 13.25 | 3.25 (-1.80, 8.55) | ≥ 14.03 | -3.06 (-8.01, 2.16) |
| Yes | < 43.89 | -11.25 (-23.05, 2.35) | < 57.99 | -10.62 (-21.97, 2.39) | < 13.25 | -11.11 (-22.54, 2.02) | < 14.03 | -18.19 (-33.12, 0.09) |
| Yes | ≥ 43.89 | -1.45 (-15.10, 14.40) | ≥ 57.99 | 1.33 (-13.47, 18.67) | ≥ 13.25 | 3.81 (-11.60, 21.90) | ≥ 14.03 | -5.82 (-16.64, 6.42) |
| RERI |  | 0.09 (-0.10, 0.28) |  | 0.10 (-0.10, 0.29) |  | 0.12 (-0.09, 0.32) |  | 0.15 (-0.04, 0.35) |
| 7.5^th^ (4d) ^b^ |  |  |  |  |  |  |  |  |
| No | < 43.89 | 1.00 | < 57.99 | 1.00 | < 13.25 | 1.00 | < 14.03 | 1.00 |
| No | ≥ 43.89 | 1.00 (-3.70, 5.94) | ≥ 57.99 | 2.25 (-2.49, 7.22) | ≥ 13.25 | 3.48 (-1.57, 8.79) | ≥ 14.03 | -3.13 (-8.05, 2.06) |
| Yes | < 43.89 | -4.53 (-18.43, 11.73) | < 57.99 | -2.70 (-16.33, 13.14) | < 13.25 | -3.03 (-16.22, 12.24) | < 14.03 | -8.07 (-25.68, 13.73) |
| Yes | ≥ 43.89 | 2.56 (-12.97, 20.86) | ≥ 57.99 | 2.76 (-13.51, 22.09) | ≥ 13.25 | 6.34 (-11.42, 27.65) | ≥ 14.03 | -2.27 (-14.69, 11.96) |
| RERI |  | 0.06 (-0.16, 0.28) |  | 0.03 (-0.19, 0.26) |  | 0.06 (-0.18, 0.29) |  | 0.09 (-0.15, 0.32) |
| 5^th^ (2d) ^b^ |  |  |  |  |  |  |  |  |
| No | < 43.89 | 1.00 | < 57.99 | 1.00 | < 13.25 | 1.00 | < 14.03 | 1.00 |
| No | ≥ 43.89 | 1.07 (-3.66, 6.04) | ≥ 57.99 | 2.47 (-2.32, 7.49) | ≥ 13.25 | 4.19 (-0.90, 9.53) | ≥ 14.03 | -3.18 (-8.16, 2.07) |
| Yes | < 43.89 | -9.75 (-22.59, 5.21) | < 57.99 | -7.88 (-19.91, 5.95) | < 13.25 | -4.64 (-17.22, 9.84) | < 14.03 | **-18.52 (-33.36, -0.38)** |
| Yes | ≥ 43.89 | -2.06 (-16.57, 14.97) | ≥ 57.99 | -0.42 (-16.65, 18.97) | ≥ 13.25 | -3.63 (-19.64, 15.57) | ≥ 14.03 | -4.76 (-16.58, 8.73) |
| RERI |  | 0.07 (-0.14, 0.27) |  | 0.05 (-0.16, 0.26) |  | -0.03 (-0.25, 0.18) |  | 0.17 (-0.03, 0.37) |
| 5^th^ (3d) ^b^ |  |  |  |  |  |  |  |  |
| No | < 43.89 | 1.00 | < 57.99 | 1.00 | < 13.25 | 1.00 | < 14.03 | 1.00 |
| No | ≥ 43.89 | 1.20 (-3.49, 6.12) | ≥ 57.99 | 2.76 (-1.97, 7.72) | ≥ 13.25 | 3.64 (-1.38, 8.92) | ≥ 14.03 | -3.36 (-8.26, 1.80) |
| Yes | < 43.89 | -11.09 (-25.73, 6.43) | < 57.99 | -8.91 (-23.12, 7.91) | < 13.25 | -10.57 (-24.03, 5.26) | < 14.03 | **-27.68 (-44.35, -6.01)** |
| Yes | ≥ 43.89 | -3.55 (-19.23, 15.17) | ≥ 57.99 | -3.03 (-19.79, 17.25) | ≥ 13.25 | 1.86 (-16.55, 24.34) | ≥ 14.03 | -3.64 (-16.61, 11.35) |
| RERI |  | 0.06 (-0.17, 0.30) |  | 0.03 (-0.21, 0.27) |  | 0.09 (-0.16, 0.34) |  | **0.27 (0.04, 0.51)** |
| 5^th^ (4d) ^b^ |  |  |  |  |  |  |  |  |
| No | < 43.89 | 1.00 | < 57.99 | 1.00 | < 13.25 | 1.00 | < 14.03 | 1.00 |
| No | ≥ 43.89 | 1.37 (-3.33, 6.29) | ≥ 57.99 | 2.78 (-1.95, 7.74) | ≥ 13.25 | 3.29 (-1.72, 8.55) | ≥ 14.03 | -3.01 (-7.90, 2.15) |
| Yes | < 43.89 | -3.36 (-20.30, 17.18) | < 57.99 | 1.03 (-15.73, 21.14) | < 13.25 | -8.65 (-23.23, 8.71) | < 14.03 | -14.40 (-34.87, 12.49) |
| Yes | ≥ 43.89 | -8.22 (-24.33, 11.32) | ≥ 57.99 | -12.03 (-28.54, 8.28) | ≥ 13.25 | 2.29 (-17.82, 27.31) | ≥ 14.03 | -6.54 (-20.16, 9.41) |
| RERI |  | -0.06 (-0.32, 0.19) |  | -0.16 (-0.42, 0.10) |  | 0.08 (-0.20, 0.35) |  | 0.11 (-0.17, 0.39) |

Note: PM, particulate matter; SO_2_, sulfur dioxide; NO_2_, nitrogen dioxide.

^a^ Cold spells were defined by percentile temperature thresholds (10^th^, 7.5^th^, 5^th^ and 2.5^th^) and by the number of consecutive days below the thresholds (2-4 d).

^b^ Nine definitions [10^th^ (2d), 10^th^ (3d), 10^th^ (4d), 7.5^th^ (2d), 7.5^th^ (3d), 7.5^th^ (4d), 5^th^ (2d), 5^th^ (3d), and 5^th^ (4d)] were used in Zhaoqing.

^c^ Air pollutants were classified as binary variables using the median of air pollutant (lag0) concentrations as a cut off.

## Table S19. Independent effects of cold spells on anxiety when relative humidity at lag03 was included in models.

|  | PM_2.5_ ^c^ | PM_10_ ^c^ | NO_2_ ^c^ | SO_2_ ^c^ |
| --- | --- | --- | --- | --- |
| Huizhou |  |  |  |  |
| 10^th^ (2d) ^a^ | 10.42 (5.37, 15.72) | 11.57 (6.39, 16.99) | 13.45 (8.21, 18.93) | 11.11 (6.14, 16.32) |
| 10^th^ (3d) ^a^ | 8.27 (2.93, 13.89) | 9.30 (3.85, 15.03) | 11.92 (6.31, 17.83) | 8.77 (3.53, 14.27) |
| 10^th^ (4d) ^a^ | 7.22 (1.38, 13.40) | 8.05 (2.13, 14.32) | 10.98 (4.82, 17.49) | 7.48 (1.73, 13.56) |
| 7.5^th^ (2d) ^a^ | 9.96 (4.43, 15.78) | 11.09 (5.44, 17.03) | 14.57 (8.66, 20.80) | 10.67 (5.23, 16.39) |
| 7.5^th^ (3d) ^a^ | 9.00 (3.17, 15.16) | 9.94 (4.02, 16.20) | 13.78 (7.53, 20.40) | 9.71 (3.94, 15.80) |
| 7.5^th^ (4d) ^a^ | 7.07 (0.45, 14.12) | 7.91 (1.20, 15.05) | 11.95 (4.84, 19.55) | 7.46 (0.90, 14.44) |
| 5^th^ (2d) ^a^ | 10.38 (4.09, 17.04) | 11.58 (5.16, 18.39) | 15.64 (8.85, 22.86) | 10.63 (4.44, 17.18) |
| 5^th^ (3d) ^a^ | 13.56 (6.37, 21.25) | 14.57 (7.27, 22.38) | 18.83 (11.10, 27.09) | 13.81 (6.66, 21.45) |
| 5^th^ (4d) ^a^ | 13.18 (5.03, 21.97) | 14.19 (5.92, 23.10) | 19.28 (10.45, 28.83) | 13.27 (5.16, 22.00) |
| 2.5^th^ (2d) ^a^ | 15.31 (6.87, 24.42) | 16.21 (7.67, 25.44) | 19.93 (11.01, 29.56) | 14.93 (6.57, 23.96) |
| 2.5^th^ (3d) ^a^ | 14.16 (4.77, 24.39) | 14.87 (5.40, 25.20) | 19.30 (9.32, 30.20) | 14.27 (4.88, 24.50) |
| 2.5^th^ (4d) ^a^ | 13.15 (2.82, 24.52) | 13.87 (3.45, 25.34) | 18.83 (7.79, 31.01) | 13.49 (3.13, 24.89) |
| Shenzhen |  |  |  |  |
| 10^th^ (2d) ^a^ | 7.57 (4.72, 10.49) | 8.28 (5.38, 11.26) | 11.10 (8.21, 14.08) | 7.78 (5.06, 10.57) |
| 10^th^ (3d) ^a^ | 7.59 (4.34, 10.94) | 8.40 (5.10, 11.81) | 12.18 (8.82, 15.64) | 7.44 (4.40, 10.58) |
| 10^th^ (4d) ^a^ | 6.07 (2.84, 9.41) | 6.72 (3.44, 10.11) | 10.59 (7.19, 14.10) | 6.34 (3.23, 9.54) |
| 7.5^th^ (2d) ^a^ | 5.38 (2.38, 8.46) | 5.97 (2.93, 9.10) | 10.10 (6.92, 13.37) | 6.09 (3.16, 9.10) |
| 7.5^th^ (3d) ^a^ | 5.62 (2.39, 8.96) | 6.19 (2.91, 9.58) | 11.19 (7.64, 14.84) | 5.75 (2.61, 8.98) |
| 7.5^th^ (4d) ^a^ | 4.65 (1.10, 8.33) | 5.13 (1.54, 8.85) | 9.99 (6.11, 14.01) | 4.71 (1.24, 8.31) |
| 5^th^ (2d) ^a^ | 3.43 (0.15, 6.82) | 4.02 (0.69, 7.47) | 9.41 (5.83, 13.12) | 3.95 (0.77, 7.24) |
| 5^th^ (3d) ^a^ | -0.65 (-4.18, 3.01) | -0.21 (-3.78, 3.50) | 4.33 (0.48, 8.32) | -0.20 (-3.67, 3.40) |
| 5^th^ (4d) ^a^ | 2.35 (-1.95, 6.84) | 2.84 (-1.50, 7.37) | 8.87 (4.10, 13.87) | 2.41 (-1.80, 6.80) |
| 2.5^th^ (2d) ^a^ | 14.31 (9.54, 19.30) | 14.84 (10.03, 19.86) | 18.91 (13.91, 24.13) | 13.86 (9.19, 18.74) |
| 2.5^th^ (3d) ^a^ | 9.92 (4.62, 15.48) | 10.28 (4.95, 15.87) | 15.17 (9.50, 21.14) | 9.65 (4.40, 15.16) |
| 2.5^th^ (4d) ^a^ | 10.54 (4.86, 16.53) | 10.89 (5.18, 16.90) | 16.60 (10.44, 23.09) | 10.01 (4.39, 15.94) |
| Zhaoqing |  |  |  |  |
| 10^th^ (2d) ^b^ | -1.11 (-8.79, 7.22) | -1.78 (-9.47, 6.57) | -2.05 (-9.68, 6.24) | -0.16 (-7.68, 7.98) |
| 10^th^ (3d) ^b^ | -0.41 (-8.56, 8.47) | -0.95 (-9.12, 7.95) | -1.22 (-9.41, 7.71) | 0.52 (-7.57, 9.33) |
| 10^th^ (4d) ^b^ | 8.61 (-0.96, 19.11) | 8.21 (-1.39, 18.75) | 8.27 (-1.44, 18.93) | 9.36 (-0.19, 19.81) |
| 7.5^th^ (2d) ^b^ | 0.96 (-7.70, 10.43) | 0.41 (-8.28, 9.92) | 0.14 (-8.56, 9.66) | 1.85 (-6.75, 11.24) |
| 7.5^th^ (3d) ^b^ | -3.65 (-13.06, 6.77) | -4.04 (-13.45, 6.39) | -4.30 (-13.75, 6.17) | -2.98 (-12.40, 7.45) |
| 7.5^th^ (4d) ^b^ | 1.73 (-9.13, 13.89) | 1.33 (-9.52, 13.49) | 1.24 (-9.72, 13.52) | 2.45 (-8.43, 14.63) |
| 5^th^ (2d) ^b^ | -3.31 (-13.51, 8.09) | -3.77 (-13.96, 7.63) | -3.99 (-14.17, 7.40) | -2.73 (-12.90, 8.62) |
| 5^th^ (3d) ^b^ | -4.84 (-15.99, 7.79) | -5.18 (-16.33, 7.44) | -5.46 (-16.65, 7.23) | -4.56 (-15.70, 8.04) |
| 5^th^ (4d) ^b^ | -3.64 (-15.82, 10.30) | -4.06 (-16.21, 9.86) | -4.28 (-16.52, 9.76) | -3.20 (-15.37, 10.71) |

Note: PM, particulate matter; SO_2_, sulfur dioxide; NO_2_, nitrogen dioxide.

^a^ Cold spells were defined by percentile temperature thresholds (10^th^, 7.5^th^, 5^th^ and 2.5^th^) and by the number of consecutive days below the thresholds (2-4 d).

^b^ Nine definitions [10^th^ (2d), 10^th^ (3d), 10^th^ (4d), 7.5^th^ (2d), 7.5^th^ (3d), 7.5^th^ (4d), 5^th^ (2d), 5^th^ (3d), and 5^th^ (4d)] were used in Zhaoqing.

^c^ Air pollutants at lag03 were included in each model separately.

## Table S20. Independent effects of air pollution on anxiety when relative humidity at lag03 was included in models

|  | PM_2.5_ ^c^ | PM_10_ ^c^ | NO_2_ ^c^ | SO_2_ ^c^ |
| --- | --- | --- | --- | --- |
| Huizhou |  |  |  |  |
| 10^th^ (2d) ^a^ | 1.50 (0.58, 2.42) | 1.47 (0.77, 2.18) | 13.01 (8.97, 17.21) | 11.39 (7.74, 15.16) |
| 10^th^ (3d) ^a^ | 1.35 (0.43, 2.27) | 1.33 (0.63, 2.04) | 12.57 (8.52, 16.77) | 10.82 (7.21, 14.55) |
| 10^th^ (4d) ^a^ | 1.21 (0.31, 2.12) | 1.20 (0.51, 1.89) | 11.86 (7.87, 15.99) | 10.38 (6.80, 14.07) |
| 7.5^th^ (2d) ^a^ | 1.43 (0.51, 2.35) | 1.40 (0.70, 2.11) | 13.43 (9.31, 17.71) | 11.13 (7.50, 14.88) |
| 7.5^th^ (3d) ^a^ | 1.30 (0.40, 2.22) | 1.28 (0.59, 1.98) | 12.82 (8.75, 17.04) | 10.76 (7.16, 14.48) |
| 7.5^th^ (4d) ^a^ | 1.15 (0.25, 2.06) | 1.15 (0.46, 1.83) | 11.81 (7.81, 15.95) | 10.25 (6.69, 13.94) |
| 5^th^ (2d) ^a^ | 1.30 (0.40, 2.21) | 1.30 (0.61, 2.00) | 12.90 (8.84, 17.12) | 10.58 (7.00, 14.27) |
| 5^th^ (3d) ^a^ | 1.24 (0.35, 2.14) | 1.24 (0.56, 1.92) | 12.49 (8.51, 16.62) | 10.43 (6.86, 14.11) |
| 5^th^ (4d) ^a^ | 1.17 (0.28, 2.06) | 1.17 (0.49, 1.85) | 12.16 (8.19, 16.27) | 10.21 (6.66, 13.88) |
| 2.5^th^ (2d) ^a^ | 1.16 (0.28, 2.06) | 1.16 (0.49, 1.84) | 11.67 (7.78, 15.71) | 10.07 (6.53, 13.73) |
| 2.5^th^ (3d) ^a^ | 1.05 (0.17, 1.94) | 1.07 (0.40, 1.74) | 11.21 (7.35, 15.21) | 9.98 (6.44, 13.63) |
| 2.5^th^ (4d) ^a^ | 1.03 (0.15, 1.92) | 1.04 (0.38, 1.72) | 11.03 (7.18, 15.03) | 9.98 (6.44, 13.63) |
| Shenzhen |  |  |  |  |
| 10^th^ (2d) ^a^ | 0.50 (-0.00, 1.01) | 0.65 (0.23, 1.07) | 14.65 (11.51, 17.87) | 6.51 (3.57, 9.54) |
| 10^th^ (3d) ^a^ | 0.72 (0.20, 1.24) | 0.81 (0.38, 1.24) | 15.09 (11.84, 18.44) | 7.11 (4.13, 10.19) |
| 10^th^ (4d) ^a^ | 0.56 (0.05, 1.07) | 0.66 (0.24, 1.08) | 14.03 (10.85, 17.31) | 6.80 (3.82, 9.86) |
| 7.5^th^ (2d) ^a^ | 0.29 (-0.21, 0.79) | 0.45 (0.04, 0.87) | 14.25 (11.10, 17.49) | 6.03 (3.11, 9.05) |
| 7.5^th^ (3d) ^a^ | 0.46 (-0.04, 0.96) | 0.57 (0.16, 0.98) | 14.36 (11.14, 17.67) | 6.18 (3.27, 9.18) |
| 7.5^th^ (4d) ^a^ | 0.37 (-0.12, 0.86) | 0.48 (0.08, 0.89) | 13.17 (10.04, 16.38) | 5.88 (2.99, 8.86) |
| 5^th^ (2d) ^a^ | 0.17 (-0.33, 0.66) | 0.34 (-0.07, 0.76) | 13.97 (10.78, 17.26) | 5.30 (2.42, 8.25) |
| 5^th^ (3d) ^a^ | 0.19 (-0.30, 0.68) | 0.32 (-0.09, 0.72) | 11.51 (8.43, 14.69) | 5.37 (2.49, 8.34) |
| 5^th^ (4d) ^a^ | 0.28 (-0.21, 0.77) | 0.40 (-0.00, 0.80) | 12.37 (9.26, 15.58) | 5.62 (2.73, 8.58) |
| 2.5^th^ (2d) ^a^ | 0.29 (-0.19, 0.77) | 0.43 (0.03, 0.83) | 13.29 (10.28, 16.37) | 4.87 (2.03, 7.79) |
| 2.5^th^ (3d) ^a^ | 0.31 (-0.16, 0.79) | 0.43 (0.03, 0.82) | 12.19 (9.21, 15.26) | 5.47 (2.60, 8.41) |
| 2.5^th^ (4d) ^a^ | 0.31 (-0.17, 0.79) | 0.42 (0.03, 0.82) | 12.29 (9.29, 15.36) | 5.43 (2.56, 8.38) |
| Zhaoqing |  |  |  |  |
| 10^th^ (2d) ^b^ | 0.36 (-1.09, 1.82) | -0.03 (-1.28, 1.24) | -0.60 (-5.15, 4.17) | 9.80 (0.53, 19.92) |
| 10^th^ (3d) ^b^ | 0.40 (-1.02, 1.84) | 0.03 (-1.20, 1.27) | -0.41 (-4.93, 4.32) | 9.94 (0.69, 20.03) |
| 10^th^ (4d) ^b^ | 0.57 (-0.84, 1.99) | 0.21 (-1.01, 1.44) | 0.75 (-3.77, 5.48) | 10.37 (1.14, 20.44) |
| 7.5^th^ (2d) ^b^ | 0.45 (-0.96, 1.89) | 0.08 (-1.15, 1.32) | -0.20 (-4.71, 4.53) | 10.16 (0.90, 20.26) |
| 7.5^th^ (3d) ^b^ | 0.34 (-1.05, 1.75) | -0.02 (-1.22, 1.19) | -0.63 (-5.04, 3.99) | 9.55 (0.42, 19.52) |
| 7.5^th^ (4d) ^b^ | 0.35 (-1.04, 1.76) | -0.00 (-1.20, 1.21) | -0.18 (-4.61, 4.46) | 9.31 (0.20, 19.24) |
| 5^th^ (2d) ^b^ | 0.35 (-1.04, 1.76) | -0.01 (-1.21, 1.20) | -0.54 (-4.93, 4.06) | 9.62 (0.49, 19.58) |
| 5^th^ (3d) ^b^ | 0.36 (-1.02, 1.75) | -0.01 (-1.19, 1.20) | -0.59 (-4.97, 4.00) | 9.66 (0.55, 19.59) |
| 5^th^ (4d) ^b^ | 0.27 (-1.11, 1.68) | -0.07 (-1.26, 1.13) | -0.57 (-4.97, 4.03) | 8.98 (-0.08, 18.86) |

Note: PM, particulate matter; SO_2_, sulfur dioxide; NO_2_, nitrogen dioxide.

^a^ Cold spells were defined by percentile temperature thresholds (10^th^, 7.5^th^, 5^th^ and 2.5^th^) and by the number of consecutive days below the thresholds (2-4 d).

^b^ Nine definitions [10^th^ (2d), 10^th^ (3d), 10^th^ (4d), 7.5^th^ (2d), 7.5^th^ (3d), 7.5^th^ (4d), 5^th^ (2d), 5^th^ (3d), and 5^th^ (4d)] were used in Zhaoqing.

^c^ Air pollutants at lag03 were included in each model separately.

## Table S21. Percent changes and relative excess risk due to interaction (RERI) of cold spells and air pollution exposure on anxiety when relative humidity at lag03 was included in models in three subtropical cities in China.

| Cold spells | PM_2.5_ ^c^ (μg/m^3^) | Percent Change  (95% CI) | PM_10_ ^c^ (μg/m^3^) | Percent Change  (95% CI) | NO_2_ ^c^ (μg/m^3^) | Percent Change  (95% CI) | SO_2_ ^c^ (μg/m^3^) | Percent Change  (95% CI) |
| --- | --- | --- | --- | --- | --- | --- | --- | --- |
| Huizhou |  |  |  |  |  |  |  |  |
| 10^th^ (2d) ^a^ |  |  |  |  |  |  |  |  |
| No | < 46.03 | 1.00 | < 64.71 | 1.00 | < 16.87 | 1.00 | < 14.48 | 1.00 |
| No | ≥ 46.03 | 3.42 (-0.88, 7.89) | ≥ 64.71 | 2.41 (-1.56, 6.55) | ≥ 16.87 | 14.35 (8.64, 20.35) | ≥ 14.48 | 6.25 (1.96, 10.73) |
| Yes | < 46.03 | 3.08 (-3.80, 10.45) | < 64.71 | -2.29 (-8.82, 4.71) | < 16.87 | 7.39 (-0.84, 16.30) | < 14.48 | 5.06 (-2.30, 12.96) |
| Yes | ≥ 46.03 | 17.96 (10.37, 26.06) | ≥ 64.71 | 22.73 (14.84, 31.15) | ≥ 16.87 | 27.71 (19.17, 36.87) | ≥ 14.48 | 18.22 (10.78, 26.16) |
| RERI |  | 0.11 (0.02, 0.21) |  | 0.23 (0.13, 0.32) |  | 0.06 (-0.05, 0.17) |  | 0.07 (-0.03, 0.17) |
| 10^th^ (3d) ^a^ |  |  |  |  |  |  |  |  |
| No | < 46.03 | 1.00 | < 64.71 | 1.00 | < 16.87 | 1.00 | < 14.48 | 1.00 |
| No | ≥ 46.03 | 2.85 (-1.32, 7.20) | ≥ 64.71 | 2.01 (-1.86, 6.03) | ≥ 16.87 | 13.93 (8.29, 19.86) | ≥ 14.48 | 6.16 (1.93, 10.57) |
| Yes | < 46.03 | -0.39 (-7.61, 7.39) | < 64.71 | -6.17 (-13.04, 1.25) | < 16.87 | 5.12 (-3.79, 14.86) | < 14.48 | 2.99 (-4.89, 11.52) |
| Yes | ≥ 46.03 | 15.98 (8.16, 24.37) | ≥ 64.71 | 20.43 (12.41, 29.04) | ≥ 16.87 | 24.81 (16.16, 34.09) | ≥ 14.48 | 15.64 (8.02, 23.79) |
| RERI |  | 0.14 (0.04, 0.23) |  | 0.25 (0.15, 0.34) |  | 0.06 (-0.06, 0.17) |  | 0.06 (-0.04, 0.17) |
| 10^th^ (4d) ^a^ |  |  |  |  |  |  |  |  |
| No | < 46.03 | 1.00 | < 64.71 | 1.00 | < 16.87 | 1.00 | < 14.48 | 1.00 |
| No | ≥ 46.03 | 2.90 (-1.15, 7.12) | ≥ 64.71 | 2.37 (-1.40, 6.28) | ≥ 16.87 | 13.68 (8.17, 19.48) | ≥ 14.48 | 5.52 (1.37, 9.84) |
| Yes | < 46.03 | -1.98 (-9.95, 6.70) | < 64.71 | -8.69 (-16.40, -0.26) | < 16.87 | 3.66 (-6.21, 14.57) | < 14.48 | -1.82 (-10.40, 7.58) |
| Yes | ≥ 46.03 | 15.45 (7.06, 24.49) | ≥ 64.71 | 19.69 (11.19, 28.83) | ≥ 16.87 | 24.45 (15.15, 34.49) | ≥ 14.48 | 16.50 (8.21, 25.43) |
| RERI |  | 0.15 (0.03, 0.26) |  | 0.26 (0.15, 0.37) |  | 0.07 (-0.06, 0.20) |  | 0.13 (0.01, 0.25) |
| 7.5^th^ (2d) ^a^ |  |  |  |  |  |  |  |  |
| No | < 46.03 | 1.00 | < 64.71 | 1.00 | < 16.87 | 1.00 | < 14.48 | 1.00 |
| No | ≥ 46.03 | 3.20 (-0.97, 7.54) | ≥ 64.71 | 2.70 (-1.17, 6.72) | ≥ 16.87 | 13.14 (7.56, 19.02) | ≥ 14.48 | 6.28 (2.05, 10.68) |
| Yes | < 46.03 | 1.33 (-6.16, 9.41) | < 64.71 | -3.78 (-10.99, 4.01) | < 16.87 | 3.10 (-5.67, 12.68) | < 14.48 | 3.77 (-3.96, 12.13) |
| Yes | ≥ 46.03 | 17.48 (9.51, 26.04) | ≥ 64.71 | 21.70 (13.49, 30.50) | ≥ 16.87 | 28.40 (19.30, 38.21) | ≥ 14.48 | 19.03 (10.79, 27.89) |
| RERI |  | 0.13 (0.03, 0.23) |  | 0.23 (0.13, 0.33) |  | 0.12 (0.01, 0.24) |  | 0.09 (-0.02, 0.20) |
| 7.5^th^ (3d) ^a^ |  |  |  |  |  |  |  |  |
| No | < 46.03 | 1.00 | < 64.71 | 1.00 | < 16.87 | 1.00 | < 14.48 | 1.00 |
| No | ≥ 46.03 | 3.54 (-0.56, 7.81) | ≥ 64.71 | 3.02 (-0.79, 6.97) | ≥ 16.87 | 13.58 (8.06, 19.39) | ≥ 14.48 | 6.42 (2.23, 10.79) |
| Yes | < 46.03 | 1.83 (-6.22, 10.57) | < 64.71 | -4.45 (-12.24, 4.04) | < 16.87 | 4.07 (-5.43, 14.52) | < 14.48 | 3.87 (-4.60, 13.09) |
| Yes | ≥ 46.03 | 16.21 (7.88, 25.19) | ≥ 64.71 | 20.55 (12.06, 29.69) | ≥ 16.87 | 27.22 (17.82, 37.38) | ≥ 14.48 | 17.66 (9.09, 26.89) |
| RERI |  | 0.11 (-0.01, 0.22) |  | 0.22 (0.11, 0.33) |  | 0.10 (-0.03, 0.22) |  | 0.07 (-0.04, 0.19) |
| 7.5^th^ (4d) ^a^ |  |  |  |  |  |  |  |  |
| No | < 46.03 | 1.00 | < 64.71 | 1.00 | < 16.87 | 1.00 | < 14.48 | 1.00 |
| No | ≥ 46.03 | 3.31 (-0.69, 7.47) | ≥ 64.71 | 2.85 (-0.86, 6.70) | ≥ 16.87 | 12.59 (7.22, 18.22) | ≥ 14.48 | 5.85 (1.72, 10.16) |
| Yes | < 46.03 | -1.65 (-10.82, 8.46) | < 64.71 | -10.41 (-19.28, -0.57) | < 16.87 | -1.86 (-12.40, 9.95) | < 14.48 | -1.41 (-10.96, 9.17) |
| Yes | ≥ 46.03 | 15.28 (5.97, 25.41) | ≥ 64.71 | 20.30 (10.86, 30.54) | ≥ 16.87 | 26.78 (16.35, 38.15) | ≥ 14.48 | 17.61 (8.03, 28.04) |
| RERI |  | 0.14 (0.01, 0.27) |  | 0.28 (0.15, 0.41) |  | 0.16 (0.01, 0.31) |  | 0.13 (-0.01, 0.27) |
| 5^th^ (2d) ^a^ |  |  |  |  |  |  |  |  |
| No | < 46.03 | 1.00 | < 64.71 | 1.00 | < 16.87 | 1.00 | < 14.48 | 1.00 |
| No | ≥ 46.03 | 1.89 (-2.12, 6.07) | ≥ 64.71 | 1.78 (-1.96, 5.66) | ≥ 16.87 | 11.20 (5.81, 16.88) | ≥ 14.48 | 5.34 (1.20, 9.64) |
| Yes | < 46.03 | -4.11 (-11.92, 4.39) | < 64.71 | -9.45 (-16.91, -1.31) | < 16.87 | -3.52 (-12.33, 6.18) | < 14.48 | -1.01 (-9.03, 7.73) |
| Yes | ≥ 46.03 | 25.09 (15.23, 35.79) | ≥ 64.71 | 31.47 (21.23, 42.57) | ≥ 16.87 | 35.42 (24.63, 47.15) | ≥ 14.48 | 25.92 (15.79, 36.94) |
| RERI |  | 0.27 (0.15, 0.40) |  | 0.39 (0.27, 0.52) |  | 0.28 (0.14, 0.41) |  | 0.22 (0.09, 0.34) |
| 5^th^ (3d) ^a^ |  |  |  |  |  |  |  |  |
| No | < 46.03 | 1.00 | < 64.71 | 1.00 | < 16.87 | 1.00 | < 14.48 | 1.00 |
| No | ≥ 46.03 | 2.95 (-1.01, 7.07) | ≥ 64.71 | 2.71 (-0.98, 6.53) | ≥ 16.87 | 11.14 (5.87, 16.69) | ≥ 14.48 | 5.77 (1.65, 10.06) |
| Yes | < 46.03 | 0.84 (-8.74, 11.43) | < 64.71 | -7.03 (-16.11, 3.02) | < 16.87 | -4.96 (-15.33, 6.67) | < 14.48 | 2.53 (-7.21, 13.28) |
| Yes | ≥ 46.03 | 25.80 (15.17, 37.42) | ≥ 64.71 | 32.49 (21.51, 44.47) | ≥ 16.87 | 39.91 (28.08, 52.83) | ≥ 14.48 | 28.59 (17.39, 40.85) |
| RERI |  | 0.22 (0.07, 0.37) |  | 0.37 (0.22, 0.51) |  | 0.34 (0.18, 0.49) |  | 0.20 (0.05, 0.35) |
| 5^th^ (4d) ^a^ |  |  |  |  |  |  |  |  |
| No | < 46.03 | 1.00 | < 64.71 | 1.00 | < 16.87 | 1.00 | < 14.48 | 1.00 |
| No | ≥ 46.03 | 2.70 (-1.22, 6.77) | ≥ 64.71 | 2.57 (-1.08, 6.36) | ≥ 16.87 | 10.20 (4.99, 15.67) | ≥ 14.48 | 4.89 (0.82, 9.12) |
| Yes | < 46.03 | -3.86 (-14.00, 7.47) | < 64.71 | -12.70 (-22.56, -1.58) | < 16.87 | -11.71 (-22.25, 0.26) | < 14.48 | -7.09 (-17.28, 4.35) |
| Yes | ≥ 46.03 | 31.61 (18.89, 45.68) | ≥ 64.71 | 36.57 (23.95, 50.49) | ≥ 16.87 | 48.38 (34.11, 64.16) | ≥ 14.48 | 36.81 (23.55, 51.49) |
| RERI |  | 0.33 (0.16, 0.49) |  | 0.47 (0.30, 0.63) |  | 0.50 (0.32, 0.68) |  | 0.39 (0.22, 0.56) |
| 2.5^th^ (2d) ^a^ |  |  |  |  |  |  |  |  |
| No | < 46.03 | 1.00 | < 64.71 | 1.00 | < 16.87 | 1.00 | < 14.48 | 1.00 |
| No | ≥ 46.03 | 2.70 (-1.20, 6.75) | ≥ 64.71 | 2.95 (-0.69, 6.73) | ≥ 16.87 | 9.12 (3.97, 14.51) | ≥ 14.48 | 5.85 (1.79, 10.07) |
| Yes | < 46.03 | -0.66 (-10.91, 10.77) | < 64.71 | -7.80 (-17.94, 3.60) | < 16.87 | -13.24 (-23.18, -2.03) | < 14.48 | 2.16 (-9.02, 14.71) |
| Yes | ≥ 46.03 | 35.42 (21.66, 50.74) | ≥ 64.71 | 40.47 (26.77, 55.64) | ≥ 16.87 | 59.52 (43.65, 77.15) | ≥ 14.48 | 32.74 (19.47, 47.49) |
| RERI |  | 0.33 (0.16, 0.51) |  | 0.45 (0.28, 0.63) |  | 0.64 (0.45, 0.83) |  | 0.25 (0.07, 0.43) |
| 2.5^th^ (3d) ^a^ |  |  |  |  |  |  |  |  |
| No | < 46.03 | 1.00 | < 64.71 | 1.00 | < 16.87 | 1.00 | < 14.48 | 1.00 |
| No | ≥ 46.03 | 3.54 (-0.34, 7.56) | ≥ 64.71 | 3.54 (-0.07, 7.29) | ≥ 16.87 | 10.08 (4.98, 15.44) | ≥ 14.48 | 6.12 (2.06, 10.33) |
| Yes | < 46.03 | 3.12 (-9.94, 18.07) | < 64.71 | -9.69 (-22.19, 4.83) | < 16.87 | -13.58 (-24.61, -0.94) | < 14.48 | 2.86 (-9.86, 17.37) |
| Yes | ≥ 46.03 | 25.81 (12.22, 41.05) | ≥ 64.71 | 33.27 (19.58, 48.53) | ≥ 16.87 | 63.10 (44.69, 83.86) | ≥ 14.48 | 31.26 (16.62, 47.73) |
| RERI |  | 0.19 (-0.01, 0.39) |  | 0.39 (0.20, 0.59) |  | 0.67 (0.44, 0.89) |  | 0.22 (0.02, 0.43) |
| 2.5^th^ (4d) ^a^ |  |  |  |  |  |  |  |  |
| No | < 46.03 | 1.00 | < 64.71 | 1.00 | < 16.87 | 1.00 | < 14.48 | 1.00 |
| No | ≥ 46.03 | 3.69 (-0.17, 7.71) | ≥ 64.71 | 3.63 (0.03, 7.37) | ≥ 16.87 | 10.45 (5.38, 15.77) | ≥ 14.48 | 5.74 (1.70, 9.93) |
| Yes | < 46.03 | 1.91 (-12.49, 18.69) | < 64.71 | -15.01 (-28.59, 1.16) | < 16.87 | -17.65 (-29.47, -3.85) | < 14.48 | -6.56 (-20.29, 9.53) |
| Yes | ≥ 46.03 | 24.87 (10.09, 41.63) | ≥ 64.71 | 33.38 (18.56, 50.06) | ≥ 16.87 | 65.33 (44.94, 88.57) | ≥ 14.48 | 34.51 (18.66, 52.47) |
| RERI |  | 0.19 (-0.03, 0.41) |  | 0.45 (0.23, 0.66) |  | 0.73 (0.48, 0.97) |  | 0.35 (0.13, 0.58) |
| Shenzhen |  |  |  |  |  |  |  |  |
| 10^th^ (2d) ^a^ |  |  |  |  |  |  |  |  |
| No | < 39.46 | 1.00 | < 58.15 | 1.00 | < 14.71 | 1.00 | < 13.27 | 1.00 |
| No | ≥ 39.46 | 2.06 (-0.12, 4.27) | ≥ 58.15 | 2.10 (-0.14, 4.39) | ≥ 14.71 | 11.86 (8.82, 14.99) | ≥ 13.27 | 2.77 (0.34, 5.25) |
| Yes | < 39.46 | 7.69 (3.99, 11.53) | < 58.15 | 5.86 (2.21, 9.64) | < 14.71 | 9.31 (4.52, 14.33) | < 13.27 | 2.53 (-1.80, 7.04) |
| Yes | ≥ 39.46 | 8.96 (4.95, 13.14) | ≥ 58.15 | 12.11 (7.77, 16.62) | ≥ 14.71 | 19.98 (15.62, 24.52) | ≥ 13.27 | 12.08 (8.19, 16.12) |
| RERI |  | -0.01 (-0.06, 0.04) |  | 0.04 (-0.01, 0.09) |  | -0.01 (-0.07, 0.04) |  | 0.07 (0.01, 0.12) |
| 10^th^ (3d) ^a^ |  |  |  |  |  |  |  |  |
| No | < 39.46 | 1.00 | < 58.15 | 1.00 | < 14.71 | 1.00 | < 13.27 | 1.00 |
| No | ≥ 39.46 | 2.83 (0.66, 5.05) | ≥ 58.15 | 2.79 (0.55, 5.09) | ≥ 14.71 | 11.66 (8.62, 14.78) | ≥ 13.27 | 2.81 (0.41, 5.27) |
| Yes | < 39.46 | 6.86 (2.74, 11.14) | < 58.15 | 4.66 (0.64, 8.84) | < 14.71 | 9.62 (3.88, 15.68) | < 13.27 | 1.57 (-2.98, 6.34) |
| Yes | ≥ 39.46 | 9.75 (5.38, 14.29) | ≥ 58.15 | 13.63 (8.86, 18.62) | ≥ 14.71 | 18.89 (14.30, 23.66) | ≥ 13.27 | 12.57 (8.11, 17.22) |
| RERI |  | 0.01 (-0.05, 0.05) |  | 0.06 (0.01, 0.12) |  | -0.02 (-0.09, 0.04) |  | 0.08 (0.02, 0.14) |
| 10^th^ (4d) ^a^ |  |  |  |  |  |  |  |  |
| No | < 39.46 | 1.00 | < 58.15 | 1.00 | < 14.71 | 1.00 | < 13.27 | 1.00 |
| No | ≥ 39.46 | 1.59 (-0.49, 3.71) | ≥ 58.15 | 1.58 (-0.58, 3.78) | ≥ 14.71 | 10.53 (7.58, 13.56) | ≥ 13.27 | 2.15 (-0.23, 4.58) |
| Yes | < 39.46 | 2.54 (-1.60, 6.85) | < 58.15 | 0.48 (-3.56, 4.69) | < 14.71 | 5.07 (-0.73, 11.22) | < 13.27 | -3.52 (-8.15, 1.34) |
| Yes | ≥ 39.46 | 9.75 (5.35, 14.32) | ≥ 58.15 | 13.64 (8.82, 18.67) | ≥ 14.71 | 17.48 (12.88, 22.26) | ≥ 13.27 | 13.33 (8.70, 18.16) |
| RERI |  | 0.06 (0.01, 0.11) |  | 0.12 (0.06, 0.17) |  | 0.02 (-0.05, 0.09) |  | 0.15 (0.09, 0.21) |
| 7.5^th^ (2d) ^a^ |  |  |  |  |  |  |  |  |
| No | < 39.46 | 1.00 | < 58.15 | 1.00 | < 14.71 | 1.00 | < 13.27 | 1.00 |
| No | ≥ 39.46 | 1.39 (-0.71, 3.53) | ≥ 58.15 | 0.97 (-1.18, 3.17) | ≥ 14.71 | 12.30 (9.22, 15.46) | ≥ 13.27 | 2.22 (-0.13, 4.64) |
| Yes | < 39.46 | 5.73 (1.56, 10.07) | < 58.15 | 2.08 (-1.95, 6.26) | < 14.71 | 9.19 (3.92, 14.72) | < 13.27 | -2.45 (-6.98, 2.30) |
| Yes | ≥ 39.46 | 6.21 (2.13, 10.45) | ≥ 58.15 | 10.03 (5.59, 14.66) | ≥ 14.71 | 19.37 (14.63, 24.31) | ≥ 13.27 | 11.67 (7.49, 16.02) |
| RERI |  | -0.01 (-0.06, 0.05) |  | 0.07 (0.01, 0.13) |  | -0.02 (-0.08, 0.04) |  | 0.12 (0.06, 0.18) |
| 7.5^th^ (3d) ^a^ |  |  |  |  |  |  |  |  |
| No | < 39.46 | 1.00 | < 58.15 | 1.00 | < 14.71 | 1.00 | < 13.27 | 1.00 |
| No | ≥ 39.46 | 2.50 (0.45, 4.60) | ≥ 58.15 | 2.17 (0.07, 4.31) | ≥ 14.71 | 12.31 (9.31, 15.41) | ≥ 13.27 | 2.86 (0.51, 5.25) |
| Yes | < 39.46 | 6.95 (2.31, 11.82) | < 58.15 | 2.51 (-1.87, 7.08) | < 14.71 | 11.17 (5.51, 17.14) | < 13.27 | 1.02 (-4.10, 6.41) |
| Yes | ≥ 39.46 | 6.48 (2.27, 10.86) | ≥ 58.15 | 10.62 (6.02, 15.41) | ≥ 14.71 | 18.14 (13.23, 23.27) | ≥ 13.27 | 10.30 (5.88, 14.90) |
| RERI |  | -0.03 (-0.09, 0.03) |  | 0.06 (-0.01, 0.12) |  | -0.05 (-0.12, 0.01) |  | 0.06 (-0.01, 0.13) |
| 7.5^th^ (4d) ^a^ |  |  |  |  |  |  |  |  |
| No | < 39.46 | 1.00 | < 58.15 | 1.00 | < 14.71 | 1.00 | < 13.27 | 1.00 |
| No | ≥ 39.46 | 1.45 (-0.54, 3.47) | ≥ 58.15 | 1.38 (-0.66, 3.46) | ≥ 14.71 | 11.13 (8.22, 14.12) | ≥ 13.27 | 2.62 (0.31, 4.98) |
| Yes | < 39.46 | 1.08 (-4.18, 6.63) | < 58.15 | -2.45 (-7.27, 2.63) | < 14.71 | 7.55 (1.39, 14.08) | < 13.27 | -4.50 (-10.82, 2.26) |
| Yes | ≥ 39.46 | 7.49 (2.99, 12.19) | ≥ 58.15 | 11.75 (6.76, 16.97) | ≥ 14.71 | 16.40 (11.25, 21.78) | ≥ 13.27 | 10.19 (5.50, 15.09) |
| RERI |  | 0.05 (-0.02, 0.12) | . | 0.13 (0.06, 0.20) |  | -0.02 (-0.10, 0.05) |  | 0.12 (0.04, 0.20) |
| 5^th^ (2d) ^a^ |  |  |  |  |  |  |  |  |
| No | < 39.46 | 1.00 | < 58.15 | 1.00 | < 14.71 | 1.00 | < 13.27 | 1.00 |
| No | ≥ 39.46 | 0.86 (-1.14, 2.90) | ≥ 58.15 | 1.02 (-1.05, 3.13) | ≥ 14.71 | 9.58 (6.69, 12.55) | ≥ 13.27 | 2.71 (0.39, 5.08) |
| Yes | < 39.46 | 3.41 (-1.18, 8.21) | < 58.15 | 1.08 (-3.23, 5.58) | < 14.71 | -1.14 (-6.52, 4.55) | < 13.27 | -2.93 (-8.16, 2.60) |
| Yes | ≥ 39.46 | 4.06 (-0.27, 8.58) | ≥ 58.15 | 7.56 (2.70, 12.65) | ≥ 14.71 | 17.63 (12.72, 22.75) | ≥ 13.27 | 9.20 (4.80, 13.80) |
| RERI |  | -0.01 (-0.06, 0.06) |  | 0.05 (-0.01, 0.12) |  | 0.09 (0.03, 0.16) |  | 0.09 (0.03, 0.16) |
| 5^th^ (3d) ^a^ |  |  |  |  |  |  |  |  |
| No | < 39.46 | 1.00 | < 58.15 | 1.00 | < 14.71 | 1.00 | < 13.27 | 1.00 |
| No | ≥ 39.46 | 1.10 (-0.86, 3.09) | ≥ 58.15 | 1.23 (-0.79, 3.28) | ≥ 14.71 | 9.14 (6.32, 12.03) | ≥ 13.27 | 2.18 (-0.11, 4.53) |
| Yes | < 39.46 | -5.42 (-10.65, 0.12) | < 58.15 | -6.43 (-11.22, -1.39) | < 14.71 | -5.97 (-12.02, 0.49) | < 13.27 | -9.83 (-15.72, -3.54) |
| Yes | ≥ 39.46 | 2.83 (-1.62, 7.48) | ≥ 58.15 | 5.70 (0.76, 10.88) | ≥ 14.71 | 11.66 (6.71, 16.84) | ≥ 13.27 | 5.10 (0.46, 9.95) |
| RERI |  | 0.07 (0.01, 0.14) |  | 0.11 (0.04, 0.18) |  | 0.08 (0.01, 0.16) |  | 0.13 (0.05, 0.20) |
| 5^th^ (4d) ^a^ |  |  |  |  |  |  |  |  |
| No | < 39.46 | 1.00 | < 58.15 | 1.00 | < 14.71 | 1.00 | < 13.27 | 1.00 |
| No | ≥ 39.46 | 1.22 (-0.72, 3.19) | ≥ 58.15 | 1.40 (-0.59, 3.43) | ≥ 14.71 | 10.21 (7.40, 13.10) | ≥ 13.27 | 2.78 (0.51, 5.11) |
| Yes | < 39.46 | -5.45 (-11.98, 1.57) | < 58.15 | -7.36 (-13.30, -1.02) | < 14.71 | 1.71 (-5.82, 9.84) | < 13.27 | -11.42 (-20.70, -1.06) |
| Yes | ≥ 39.46 | 6.61 (1.36, 12.12) | ≥ 58.15 | 10.11 (4.39, 16.15) | ≥ 14.71 | 14.30 (8.48, 20.43) | ≥ 13.27 | 7.02 (1.89, 12.41) |
| RERI |  | 0.11 (0.03, 0.19) |  | 0.16 (0.08, 0.24) |  | 0.02 (-0.07, 0.11) |  | 0.16 (0.05, 0.26) |
| 2.5^th^ (2d) ^a^ |  |  |  |  |  |  |  |  |
| No | < 39.46 | 1.00 | < 58.15 | 1.00 | < 14.71 | 1.00 | < 13.27 | 1.00 |
| No | ≥ 39.46 | 0.85 (-1.07, 2.81) | ≥ 58.15 | 1.47 (-0.50, 3.47) | ≥ 14.71 | 9.63 (6.81, 12.52) | ≥ 13.27 | 3.08 (0.81, 5.40) |
| Yes | < 39.46 | 11.76 (5.22, 18.70) | < 58.15 | 10.74 (4.40, 17.47) | < 14.71 | 5.73 (-1.60, 13.60) | < 13.27 | 6.61 (-2.32, 16.35) |
| Yes | ≥ 39.46 | 17.19 (10.43, 24.36) | ≥ 58.15 | 20.23 (12.89, 28.05) | ≥ 14.71 | 31.91 (25.01, 39.20) | ≥ 13.27 | 19.29 (13.50, 25.38) |
| RERI |  | 0.05 (-0.05, 0.14) |  | 0.08 (-0.02, 0.18) |  | 0.16 (0.07, 0.26) |  | 0.10 (-0.01, 0.20) |
| 2.5^th^ (3d) ^a^ |  |  |  |  |  |  |  |  |
| No | < 39.46 | 1.00 | < 58.15 | 1.00 | < 14.71 | 1.00 | < 13.27 | 1.00 |
| No | ≥ 39.46 | 1.15 (-0.75, 3.10) | ≥ 58.15 | 1.70 (-0.25, 3.68) | ≥ 14.71 | 10.07 (7.25, 12.97) | ≥ 13.27 | 3.07 (0.81, 5.37) |
| Yes | < 39.46 | -1.42 (-9.15, 6.96) | < 58.15 | -1.35 (-8.42, 6.26) | < 14.71 | 5.50 (-2.81, 14.52) | < 13.27 | 11.74 (-5.29, 31.84) |
| Yes | ≥ 39.46 | 18.16 (10.96, 25.82) | ≥ 58.15 | 22.43 (14.46, 30.96) | ≥ 14.71 | 28.42 (20.27, 37.12) | ≥ 13.27 | 12.35 (6.38, 18.65) |
| RERI |  | 0.18 (0.08, 0.29) |  | 0.22 (0.11, 0.33) |  | 0.13 (0.01, 0.25) |  | -0.02 (-0.22, 0.17) |
| 2.5^th^ (4d) ^a^ |  |  |  |  |  |  |  |  |
| No | < 39.46 | 1.00 | < 58.15 | 1.00 | < 14.71 | 1.00 | < 13.27 | 1.00 |
| No | ≥ 39.46 | 1.18 (-0.73, 3.12) | ≥ 58.15 | 1.75 (-0.19, 3.74) | ≥ 14.71 | 9.87 (7.07, 12.75) | ≥ 13.27 | 3.04 (0.78, 5.34) |
| Yes | < 39.46 | -4.17 (-13.12, 5.70) | < 58.15 | -3.00 (-11.03, 5.75) | < 14.71 | 4.81 (-3.88, 14.29) | < 13.27 | 48.55 (-17.85, 168.61) |
| Yes | ≥ 39.46 | 18.18 (10.93, 25.91) | ≥ 58.15 | 21.86 (13.89, 30.39) | ≥ 14.71 | 29.54 (20.80, 38.91) | ≥ 13.27 | 12.68 (6.57, 19.15) |
| RERI |  | 0.21 (0.09, 0.33) |  | 0.23 (0.11, 0.35) |  | 0.15 (0.02, 0.28) |  | -0.39 (-1.27, 0.49) |
| Zhaoqing |  |  |  |  |  |  |  |  |
| 10^th^ (2d) ^b^ |  |  |  |  |  |  |  |  |
| No | < 44.50 | 1.00 | < 59.50 | 1.00 | < 13.41 | 1.00 | < 14.58 | 1.00 |
| No | ≥ 44.50 | -0.07 (-5.14, 5.27) | ≥ 59.50 | 0.04 (-5.21, 5.59) | ≥ 13.41 | -0.85 (-6.32, 4.94) | ≥ 14.58 | 3.02 (-2.70, 9.08) |
| Yes | < 44.50 | -7.02 (-15.82, 2.69) | < 59.50 | -7.13 (-15.99, 2.65) | < 13.41 | -3.87 (-13.13, 6.38) | < 14.58 | -5.71 (-15.56, 5.28) |
| Yes | ≥ 44.50 | 9.92 (-3.05, 24.62) | ≥ 59.50 | 12.36 (-1.72, 28.46) | ≥ 13.41 | 0.56 (-11.77, 14.61) | ≥ 14.58 | 6.77 (-4.31, 19.12) |
| RERI |  | 0.17 (0.01, 0.33) |  | 0.19 (0.02, 0.37) |  | 0.05 (-0.10, 0.21) |  | 0.09 (-0.05, 0.24) |
| 10^th^ (3d) ^b^ |  |  |  |  |  |  |  |  |
| No | < 44.50 | 1.00 | < 59.50 | 1.00 | < 13.41 | 1.00 | < 14.58 | 1.00 |
| No | ≥ 44.50 | -0.01 (-4.96, 5.20) | ≥ 59.50 | 0.07 (-5.01, 5.42) | ≥ 13.41 | -1.44 (-6.82, 4.25) | ≥ 14.58 | 3.22 (-2.48, 9.26) |
| Yes | < 44.50 | -8.27 (-17.80, 2.37) | < 59.50 | -8.72 (-18.07, 1.71) | < 13.41 | -6.46 (-16.35, 4.61) | < 14.58 | -6.01 (-17.47, 7.04) |
| Yes | ≥ 44.50 | 12.22 (-1.34, 27.65) | ≥ 59.50 | 16.67 (1.54, 34.06) | ≥ 13.41 | 5.31 (-7.97, 20.50) | ≥ 14.58 | 6.88 (-4.34, 19.40) |
| RERI |  | 0.21 (0.03, 0.38) |  | 0.25 (0.07, 0.44) |  | 0.13 (-0.04, 0.30) |  | 0.10 (-0.07, 0.26) |
| 10^th^ (4d) ^b^ |  |  |  |  |  |  |  |  |
| No | < 44.50 | 1.00 | < 59.50 | 1.00 | < 13.41 | 1.00 | < 14.58 | 1.00 |
| No | ≥ 44.50 | 0.50 (-4.37, 5.61) | ≥ 59.50 | 0.71 (-4.28, 5.96) | ≥ 13.41 | -0.56 (-5.92, 5.11) | ≥ 14.58 | 2.49 (-3.17, 8.49) |
| Yes | < 44.50 | -1.65 (-13.31, 11.58) | < 59.50 | -2.81 (-14.05, 9.90) | < 13.41 | 0.13 (-11.13, 12.81) | < 14.58 | -1.97 (-15.42, 13.60) |
| Yes | ≥ 44.50 | 21.45 (6.46, 38.54) | ≥ 59.50 | 27.81 (10.85, 47.37) | ≥ 13.41 | 21.63 (4.58, 41.45) | ≥ 14.58 | 18.27 (5.17, 32.99) |
| RERI |  | 0.23 (0.03, 0.42) |  | 0.30 (0.09, 0.51) |  | 0.22 (0.01, 0.43) |  | 0.18 (-0.02, 0.37) |
| 7.5^th^ (2d) ^b^ |  |  |  |  |  |  |  |  |
| No | < 44.50 | 1.00 | < 59.50 | 1.00 | < 13.41 | 1.00 | < 14.58 | 1.00 |
| No | ≥ 44.50 | 0.26 (-4.63, 5.40) | ≥ 59.50 | 0.84 (-4.22, 6.17) | ≥ 13.41 | -0.65 (-6.02, 5.03) | ≥ 14.58 | 3.82 (-1.83, 9.80) |
| Yes | < 44.50 | -7.34 (-17.18, 3.68) | < 59.50 | -5.95 (-15.77, 5.01) | < 13.41 | -2.94 (-13.18, 8.51) | < 14.58 | -2.68 (-14.40, 10.65) |
| Yes | ≥ 44.50 | 16.26 (1.20, 33.56) | ≥ 59.50 | 18.51 (1.85, 37.89) | ≥ 13.41 | 5.61 (-9.26, 22.91) | ≥ 14.58 | 8.23 (-4.14, 22.20) |
| RERI |  | 0.23 (0.05, 0.42) |  | 0.24 (0.04, 0.44) |  | 0.09 (-0.09, 0.28) |  | 0.07 (-0.11, 0.25) |
| 7.5^th^ (3d) ^b^ |  |  |  |  |  |  |  |  |
| No | < 44.50 | 1.00 | < 59.50 | 1.00 | < 13.41 | 1.00 | < 14.58 | 1.00 |
| No | ≥ 44.50 | -0.46 (-5.19, 4.50) | ≥ 59.50 | 0.12 (-4.75, 5.23) | ≥ 13.41 | -1.46 (-6.62, 3.99) | ≥ 14.58 | 2.40 (-3.06, 8.17) |
| Yes | < 44.50 | -19.69 (-30.44, -7.27) | < 59.50 | -16.13 (-26.71, -4.02) | < 13.41 | -12.21 (-23.46, 0.68) | < 14.58 | -20.41 (-33.88, -4.19) |
| Yes | ≥ 44.50 | 17.28 (1.39, 35.65) | ≥ 59.50 | 19.84 (2.02, 40.77) | ≥ 13.41 | 6.57 (-9.24, 25.13) | ≥ 14.58 | 7.63 (-5.10, 22.06) |
| RERI |  | 0.37 (017, 0.58) |  | 0.36 (0.14, 0.58) |  | 0.20 (-0.01, 0.41) |  | 0.26 (0.06, 0.46) |
| 7.5^th^ (4d) ^b^ |  |  |  |  |  |  |  |  |
| No | < 44.50 | 1.00 | < 59.50 | 1.00 | < 13.41 | 1.00 | < 14.58 | 1.00 |
| No | ≥ 44.50 | 0.51 (-4.20, 5.45) | ≥ 59.50 | 0.80 (-4.05, 5.88) | ≥ 13.41 | -0.58 (-5.77, 4.90) | ≥ 14.58 | 2.63 (-2.83, 8.40) |
| Yes | < 44.50 | -11.76 (-24.96, 3.76) | < 59.50 | -10.23 (-22.83, 4.42) | < 13.41 | -5.05 (-17.59, 9.39) | < 14.58 | -14.07 (-30.21, 5.80) |
| Yes | ≥ 44.50 | 17.60 (0.43, 37.71) | ≥ 59.50 | 22.69 (3.01, 46.13) | ≥ 13.41 | 14.16 (-6.02, 38.68) | ≥ 14.58 | 12.54 (-1.96, 29.19) |
| RERI |  | 0.29 (0.06, 0.52) |  | 0.32 (0.07, 0.57) |  | 0.20 (-0.06, 0.45) |  | 0.24 (0.01, 0.48) |
| 5^th^ (2d) ^b^ |  |  |  |  |  |  |  |  |
| No | < 44.50 | 1.00 | < 59.50 | 1.00 | < 13.41 | 1.00 | < 14.58 | 1.00 |
| No | ≥ 44.50 | 0.14 (-4.58, 5.09) | ≥ 59.50 | 0.45 (-4.38, 5.52) | ≥ 13.41 | -0.73 (-5.94, 4.77) | ≥ 14.58 | 2.87 (-2.60, 8.64) |
| Yes | < 44.50 | -17.79 (-29.43, -4.24) | < 59.50 | -17.10 (-28.39, -4.03) | < 13.41 | -7.65 (-20.16, 6.81) | < 14.58 | -17.51 (-31.98, 0.03) |
| Yes | ≥ 44.50 | 17.16 (-0.16, 37.48) | ≥ 59.50 | 22.76 (3.20, 46.03) | ≥ 13.41 | 1.38 (-15.11, 21.07) | ≥ 14.58 | 7.51 (-6.23, 23.25) |
| RERI |  | 0.35 (0.12, 0.57) |  | 0.39 (0.15, 0.64) |  | 0.10 (-0.13, 0.32) |  | 0.22 (0.01, 0.44) |
| 5^th^ (3d) ^b^ |  |  |  |  |  |  |  |  |
| No | < 44.50 | 1.00 | < 59.50 | 1.00 | < 13.41 | 1.00 | < 14.58 | 1.00 |
| No | ≥ 44.50 | 0.72 (-3.96, 5.63) | ≥ 59.50 | 0.87 (-3.92, 5.90) | ≥ 13.41 | -0.73 (-5.87, 4.70) | ≥ 14.58 | 2.86 (-2.55, 8.56) |
| Yes | < 44.50 | -21.37 (-34.64, -5.41) | < 59.50 | -20.58 (-33.14, -5.66) | < 13.41 | -9.65 (-22.82, 5.77) | < 14.58 | -26.22 (-42.35, -5.57) |
| Yes | ≥ 44.50 | 14.40 (-3.68, 35.89) | ≥ 59.50 | 21.05 (0.46, 45.86) | ≥ 13.41 | 2.36 (-17.03, 26.29) | ≥ 14.58 | 7.57 (-7.24, 24.75) |
| RERI |  | 0.35 (0.10, 0.60) |  | 0.41 (0.14, 0.67) |  | 0.13 (-0.13, 0.38) |  | 0.31 (0.07, 0.55) |
| 5^th^ (4d) ^b^ |  |  |  |  |  |  |  |  |
| No | < 44.50 | 1.00 | < 59.50 | 1.00 | < 13.41 | 1.00 | < 14.58 | 1.00 |
| No | ≥ 44.50 | 1.21 (-3.47, 6.13) | ≥ 59.50 | 1.11 (-3.67, 6.14) | ≥ 13.41 | -0.94 (-6.07, 4.47) | ≥ 14.58 | 3.02 (-2.37, 8.71) |
| Yes | < 44.50 | -12.57 (-28.01, 6.18) | < 59.50 | -15.63 (-29.86, 1.48) | < 13.41 | -12.18 (-25.61, 3.67) | < 14.58 | -20.79 (-38.94, 2.76) |
| Yes | ≥ 44.50 | 7.37 (-11.28, 29.93) | ≥ 59.50 | 15.63 (-5.55, 41.57) | ≥ 13.41 | 15.44 (-9.24, 46.83) | ≥ 14.58 | 7.32 (-8.69, 26.12) |
| RERI |  | 0.19 (-0.08, 0.45) |  | 0.30 (0.02, 0.58) |  | 0.29 (-0.02, 0.59) |  | 0.25 (-0.02, 0.52) |

Note: PM, particulate matter; SO_2_, sulfur dioxide; NO_2_, nitrogen dioxide.

^a^ Cold spells were defined by percentile temperature thresholds (10^th^, 7.5^th^, 5^th^ and 2.5^th^) and by the number of consecutive days below the thresholds (2-4 d).

^b^ Nine definitions [10^th^ (2d), 10^th^ (3d), 10^th^ (4d), 7.5^th^ (2d), 7.5^th^ (3d), 7.5^th^ (4d), 5^th^ (2d), 5^th^ (3d), and 5^th^ (4d)] were used in Zhaoqing.

^c^ Air pollutants were classified as binary variables using the median of air pollutant (lag03) concentrations as a cut off.

## Table S22. Independent effects of cold spells on anxiety when outpatients without detailed home addresses were excluded.

|  | PM_2.5_ ^c^ | PM_10_ ^c^ | NO_2_ ^c^ | SO_2_ ^c^ |
| --- | --- | --- | --- | --- |
| Huizhou |  |  |  |  |
| 10^th^ (2d) ^a^ | 10.84 (5.24, 16.73) | 12.29 (6.56, 18.34) | 14.63 (8.81, 20.77) | 12.06 (6.51, 17.91) |
| 10^th^ (3d) ^a^ | 10.46 (4.53, 16.72) | 11.76 (5.72, 18.15) | 14.73 (8.51, 21.31) | 10.94 (5.13, 17.08) |
| 10^th^ (4d) ^a^ | 9.82 (3.40, 16.64) | 10.83 (4.32, 17.74) | 14.00 (7.26, 21.17) | 9.78 (3.49, 16.47) |
| 7.5^th^ (2d) ^a^ | 10.79 (4.64, 17.30) | 12.21 (5.93, 18.87) | 16.40 (9.80, 23.40) | 11.76 (5.69, 18.18) |
| 7.5^th^ (3d) ^a^ | 11.48 (5.01, 18.34) | 12.62 (6.05, 19.59) | 16.91 (9.99, 24.27) | 12.00 (5.62, 18.77) |
| 7.5^th^ (4d) ^a^ | 9.11 (1.93, 16.79) | 10.09 (2.83, 17.87) | 14.54 (6.85, 22.79) | 9.20 (2.11, 16.78) |
| 5^th^ (2d) ^a^ | 10.80 (4.08, 17.94) | 12.26 (5.39, 19.57) | 17.30 (9.97, 25.12) | 11.33 (4.68, 18.40) |
| 5^th^ (3d) ^a^ | 14.81 (7.17, 22.99) | 15.99 (8.23, 24.30) | 21.05 (12.80, 29.91) | 15.14 (7.53, 23.29) |
| 5^th^ (4d) ^a^ | 14.86 (6.22, 24.20) | 15.99 (7.23, 25.47) | 21.81 (12.42, 31.99) | 14.82 (6.23, 24.10) |
| 2.5^th^ (2d) ^a^ | 16.03 (7.26, 25.52) | 17.16 (8.27, 26.79) | 21.58 (12.25, 31.69) | 15.83 (7.12, 25.25) |
| 2.5^th^ (3d) ^a^ | 15.65 (5.84, 26.37) | 16.50 (6.60, 27.33) | 21.74 (11.23, 33.23) | 15.81 (5.99, 26.53) |
| 2.5^th^ (4d) ^a^ | 13.44 (2.82, 25.16) | 14.23 (3.51, 26.05) | 19.72 (8.33, 32.31) | 13.64 (3.00, 25.39) |
| Shenzhen |  |  |  |  |
| 10^th^ (2d) ^a^ | 0.99 (-7.71, 10.52) | 1.20 (-7.58, 10.81) | 0.73 (-7.86, 10.11) | 1.16 (-7.33, 10.44) |
| 10^th^ (3d) ^a^ | 0.53 (-8.77, 10.77) | 0.56 (-8.78, 10.85) | -0.84 (-9.91, 9.14) | 0.01 (-8.86, 9.74) |
| 10^th^ (4d) ^a^ | 1.34 (-8.21, 11.89) | 1.34 (-8.23, 11.92) | 0.02 (-9.36, 10.38) | 0.80 (-8.41, 10.93) |
| 7.5^th^ (2d) ^a^ | 5.79 (-3.78, 16.32) | 6.09 (-3.58, 16.73) | 5.74 (-3.88, 16.33) | 5.73 (-3.62, 15.97) |
| 7.5^th^ (3d) ^a^ | 7.08 (-3.24, 18.50) | 7.23 (-3.16, 18.74) | 6.30 (-4.16, 17.90) | 6.24 (-3.74, 17.27) |
| 7.5^th^ (4d) ^a^ | 5.83 (-5.53, 18.55) | 5.87 (-5.52, 18.63) | 4.94 (-6.53, 17.81) | 4.86 (-6.15, 17.17) |
| 5^th^ (2d) ^a^ | 6.36 (-4.38, 18.30) | 6.69 (-4.16, 18.76) | 6.66 (-4.30, 18.88) | 5.86 (-4.55, 17.41) |
| 5^th^ (3d) ^a^ | 2.92 (-8.75, 16.09) | 3.05 (-8.69, 16.30) | 2.09 (-9.73, 15.45) | 2.06 (-9.29, 14.82) |
| 5^th^ (4d) ^a^ | 2.11 (-11.26, 17.50) | 2.14 (-11.26, 17.56) | 1.04 (-12.45, 16.62) | 0.72 (-12.22, 15.56) |
| 2.5^th^ (2d) ^a^ | 6.90 (-6.85, 22.69) | 7.09 (-6.71, 22.93) | 6.75 (-6.98, 22.51) | 5.84 (-7.56, 21.17) |
| 2.5^th^ (3d) ^a^ | 0.27 (-14.40, 17.46) | 0.31 (-14.38, 17.53) | -0.25 (-14.99, 17.04) | -0.74 (-15.19, 16.16) |
| 2.5^th^ (4d) ^a^ | -3.98 (-20.07, 15.36) | -3.95 (-20.06, 15.40) | -4.49 (-20.65, 14.97) | -5.05 (-20.93, 14.02) |
| Zhaoqing |  |  |  |  |
| 10^th^ (2d) ^b^ | -6.60 (-14.38, 1.90) | -7.22 (-15.01, 1.28) | -7.01 (-14.74, 1.41) | -6.03 (-13.66, 2.27) |
| 10^th^ (3d) ^b^ | -6.13 (-14.33, 2.86) | -6.61 (-14.82, 2.39) | -6.45 (-14.67, 2.57) | -5.66 (-13.80, 3.25) |
| 10^th^ (4d) ^b^ | 2.86 (-6.73, 13.42) | 2.51 (-7.09, 13.09) | 2.91 (-6.77, 13.59) | 3.04 (-6.49, 13.54) |
| 7.5^th^ (2d) ^b^ | -3.41 (-12.03, 6.04) | -3.85 (-12.48, 5.64) | -3.70 (-12.34, 5.80) | -3.01 (-11.57, 6.37) |
| 7.5^th^ (3d) ^b^ | -7.13 (-16.40, 3.17) | -7.36 (-16.63, 2.94) | -7.21 (-16.53, 3.16) | -6.95 (-16.21, 3.34) |
| 7.5^th^ (4d) ^b^ | -2.26 (-12.90, 9.67) | -2.53 (-13.15, 9.39) | -2.15 (-12.89, 9.90) | -2.21 (-12.82, 9.69) |
| 5^th^ (2d) ^b^ | -6.97 (-16.94, 4.21) | -7.29 (-17.27, 3.89) | -7.17 (-17.17, 4.02) | -6.77 (-16.71, 4.36) |
| 5^th^ (3d) ^b^ | -8.53 (-19.42, 3.83) | -8.72 (-19.60, 3.64) | -8.52 (-19.48, 3.93) | -8.72 (-19.56, 3.59) |
| 5^th^ (4d) ^b^ | -6.14 (-18.08, 7.53) | -6.41 (-18.32, 7.25) | -6.04 (-18.08, 7.77) | -6.35 (-18.23, 7.24) |

Note: PM, particulate matter; SO_2_, sulfur dioxide; NO_2_, nitrogen dioxide.

^a^ Cold spells were defined by percentile temperature thresholds (10^th^, 7.5^th^, 5^th^ and 2.5^th^) and by the number of consecutive days below the thresholds (2-4 d).

^b^ Nine definitions [10^th^ (2d), 10^th^ (3d), 10^th^ (4d), 7.5^th^ (2d), 7.5^th^ (3d), 7.5^th^ (4d), 5^th^ (2d), 5^th^ (3d), and 5^th^ (4d)] were used in Zhaoqing.

^c^ Air pollutants at lag03 were included in each model separately.

## Table S23. Independent effects of air pollution on anxiety when outpatients without detailed home addresses were excluded

|  | PM_2.5_ ^c^ | PM_10_ ^c^ | NO_2_ ^c^ | SO_2_ ^c^ |
| --- | --- | --- | --- | --- |
| Huizhou |  |  |  |  |
| 10^th^ (2d) ^a^ | 1.65 (0.72, 2.59) | 1.66 (0.95, 2.37) | 14.84 (10.72, 19.10) | 12.89 (9.11, 16.80) |
| 10^th^ (3d) ^a^ | 1.60 (0.67, 2.53) | 1.59 (0.89, 2.30) | 14.71 (10.59, 18.99) | 12.35 (8.62, 16.21) |
| 10^th^ (4d) ^a^ | 1.46 (0.55, 2.39) | 1.46 (0.76, 2.16) | 13.96 (9.91, 18.15) | 11.81 (8.12, 15.63) |
| 7.5^th^ (2d) ^a^ | 1.60 (0.67, 2.54) | 1.60 (0.90, 2.31) | 15.32 (11.13, 19.67) | 12.56 (8.80, 16.44) |
| 7.5^th^ (3d) ^a^ | 1.54 (0.62, 2.47) | 1.53 (0.83, 2.23) | 14.88 (10.75, 19.16) | 12.20 (8.49, 16.04) |
| 7.5^th^ (4d) ^a^ | 1.37 (0.46, 2.29) | 1.38 (0.69, 2.07) | 13.81 (9.76, 18.01) | 11.63 (7.95, 15.43) |
| 5^th^ (2d) ^a^ | 1.47 (0.55, 2.39) | 1.50 (0.80, 2.20) | 14.90 (10.75, 19.19) | 11.99 (8.29, 15.81) |
| 5^th^ (3d) ^a^ | 1.42 (0.52, 2.33) | 1.44 (0.76, 2.13) | 14.47 (10.41, 18.67) | 11.82 (8.14, 15.62) |
| 5^th^ (4d) ^a^ | 1.36 (0.46, 2.27) | 1.38 (0.70, 2.07) | 14.10 (10.08, 18.28) | 11.57 (7.91, 15.36) |
| 2.5^th^ (2d) ^a^ | 1.35 (0.45, 2.26) | 1.38 (0.70, 2.06) | 13.68 (9.70, 17.80) | 11.52 (7.86, 15.30) |
| 2.5^th^ (3d) ^a^ | 1.23 (0.34, 2.14) | 1.27 (0.60, 1.95) | 13.19 (9.25, 17.27) | 11.35 (7.70, 15.13) |
| 2.5^th^ (4d) ^a^ | 1.21 (0.31, 2.11) | 1.24 (0.57, 1.92) | 12.87 (8.95, 16.93) | 11.30 (7.65, 15.07) |
| Shenzhen |  |  |  |  |
| 10^th^ (2d) ^a^ | 0.52 (-0.96, 2.03) | 0.49 (-0.75, 1.74) | 2.34 (-5.46, 10.79) | 5.10 (-2.99, 13.87) |
| 10^th^ (3d) ^a^ | 0.72 (-0.79, 2.26) | 0.59 (-0.67, 1.86) | 0.63 (-7.16, 9.09) | 4.89 (-3.21, 13.68) |
| 10^th^ (4d) ^a^ | 0.77 (-0.72, 2.28) | 0.62 (-0.62, 1.87) | 0.68 (-7.06, 9.07) | 5.03 (-3.08, 13.82) |
| 7.5^th^ (2d) ^a^ | 0.74 (-0.75, 2.24) | 0.68 (-0.55, 1.93) | 3.69 (-4.33, 12.38) | 5.89 (-2.24, 14.71) |
| 7.5^th^ (3d) ^a^ | 0.97 (-0.51, 2.46) | 0.81 (-0.42, 2.05) | 2.49 (-5.51, 11.16) | 5.65 (-2.45, 14.41) |
| 7.5^th^ (4d) ^a^ | 0.88 (-0.59, 2.36) | 0.71 (-0.50, 1.93) | 1.68 (-6.16, 10.17) | 5.23 (-2.81, 13.93) |
| 5^th^ (2d) ^a^ | 0.69 (-0.77, 2.18) | 0.65 (-0.57, 1.88) | 3.75 (-4.29, 12.46) | 5.42 (-2.57, 14.07) |
| 5^th^ (3d) ^a^ | 0.77 (-0.68, 2.25) | 0.63 (-0.57, 1.85) | 1.26 (-6.53, 9.70) | 5.00 (-2.98, 13.64) |
| 5^th^ (4d) ^a^ | 0.75 (-0.70, 2.22) | 0.60 (-0.60, 1.82) | 0.84 (-6.90, 9.22) | 4.91 (-3.07, 13.55) |
| 2.5^th^ (2d) ^a^ | 0.60 (-0.84, 2.06) | 0.55 (-0.64, 1.76) | 2.86 (-4.83, 11.17) | 4.90 (-3.00, 13.44) |
| 2.5^th^ (3d) ^a^ | 0.70 (-0.73, 2.14) | 0.57 (-0.61, 1.76) | 0.84 (-6.71, 9.00) | 4.90 (-3.05, 13.51) |
| 2.5^th^ (4d) ^a^ | 0.68 (-0.74, 2.13) | 0.54 (-0.63, 1.73) | 0.35 (-7.14, 8.45) | 5.00 (-2.99, 13.66) |
| Zhaoqing |  |  |  |  |
| 10^th^ (2d) ^b^ | 0.46 (-0.95, 1.90) | 0.07 (-1.13, 1.29) | 0.73 (-3.80, 5.48) | 7.81 (-0.79, 17.14) |
| 10^th^ (3d) ^b^ | 0.56 (-0.83, 1.97) | 0.16 (-1.01, 1.35) | 0.98 (-3.52, 5.68) | 7.85 (-0.71, 17.16) |
| 10^th^ (4d) ^b^ | 0.72 (-0.65, 2.12) | 0.32 (-0.84, 1.50) | 1.97 (-2.53, 6.68) | 7.80 (-0.72, 17.05) |
| 7.5^th^ (2d) ^b^ | 0.68 (-0.70, 2.09) | 0.28 (-0.89, 1.47) | 1.41 (-3.10, 6.12) | 8.60 (0.00, 17.94) |
| 7.5^th^ (3d) ^b^ | 0.67 (-0.69, 2.05) | 0.27 (-0.88, 1.43) | 1.28 (-3.14, 5.91) | 8.32 (-0.20, 17.55) |
| 7.5^th^ (4d) ^b^ | 0.63 (-0.74, 2.01) | 0.24 (-0.91, 1.40) | 1.54 (-2.90, 6.19) | 7.37 (-1.07, 16.54) |
| 5^th^ (2d) ^b^ | 0.67 (-0.70, 2.06) | 0.28 (-0.88, 1.44) | 1.36 (-3.07, 6.00) | 8.56 (0.03, 17.82) |
| 5^th^ (3d) ^b^ | 0.70 (-0.66, 2.08) | 0.30 (-0.85, 1.46) | 1.36 (-3.06, 5.98) | 8.61 (0.10, 17.85) |
| 5^th^ (4d) ^b^ | 0.60 (-0.76, 1.98) | 0.21 (-0.94, 1.37) | 1.36 (-3.07, 5.99) | 7.36 (-1.07, 16.51) |

Note: PM, particulate matter; SO_2_, sulfur dioxide; NO_2_, nitrogen dioxide.

^a^ Cold spells were defined by percentile temperature thresholds (10^th^, 7.5^th^, 5^th^ and 2.5^th^) and by the number of consecutive days below the thresholds (2-4 d).

^b^ Nine definitions [10^th^ (2d), 10^th^ (3d), 10^th^ (4d), 7.5^th^ (2d), 7.5^th^ (3d), 7.5^th^ (4d), 5^th^ (2d), 5^th^ (3d), and 5^th^ (4d)] were used in Zhaoqing.

^c^ Air pollutants at lag03 were included in each model separately.

## Table S24. Percent changes and relative excess risk due to interaction (RERI) of cold spells and air pollution exposure on anxiety when outpatients without detailed home addresses were excluded

| Cold spells | PM_2.5_ ^c^ (μg/m^3^) | Percent Change  (95% CI) | PM_10_ ^c^ (μg/m^3^) | Percent Change  (95% CI) | NO_2_ ^c^ (μg/m^3^) | Percent Change  (95% CI) | SO_2_ ^c^ (μg/m^3^) | Percent Change  (95% CI) |
| --- | --- | --- | --- | --- | --- | --- | --- | --- |
| Huizhou |  |  |  |  |  |  |  |  |
| 10^th^ (2d) ^a^ |  |  |  |  |  |  |  |  |
| No | < 46.03 | 1.00 | < 64.71 | 1.00 | < 16.87 | 1.00 | < 14.48 | 1.00 |
| No | ≥ 46.03 | 5.05 (0.67, 9.62) | ≥ 64.71 | 3.80 (-0.23, 7.99) | ≥ 16.87 | 15.83 (9.89, 22.09) | ≥ 14.48 | 7.08 (2.72, 11.63) |
| Yes | < 46.03 | 4.05 (-3.26, 11.92) | < 64.71 | -2.54 (-9.41, 4.85) | < 16.87 | 6.72 (-1.85, 16.03) | < 14.48 | 3.93 (-3.70, 12.16) |
| Yes | ≥ 46.03 | 19.89 (11.68, 28.70) | ≥ 64.71 | 25.83 (17.27, 35.02) | ≥ 16.87 | 30.67 (21.34, 40.71) | ≥ 14.48 | 20.66 (12.60, 29.30) |
| RERI |  | 0.11 (0.02, 0.20) |  | 0.25 (0.15, 0.34) |  | 0.08 (-0.03, 0.19) |  | 0.10 (-0.01, 0.20) |
| 10^th^ (3d) ^a^ |  |  |  |  |  |  |  |  |
| No | < 46.03 | 1.00 | < 64.71 | 1.00 | < 16.87 | 1.00 | < 14.48 | 1.00 |
| No | ≥ 46.03 | 4.67 (0.40, 9.11) | ≥ 64.71 | 3.64 (-0.29, 7.73) | ≥ 16.87 | 15.29 (9.45, 21.45) | ≥ 14.48 | 6.94 (2.65, 11.40) |
| Yes | < 46.03 | 2.07 (-5.64, 10.40) | < 64.71 | -5.19 (-12.45, 2.67) | < 16.87 | 4.81 (-4.49, 15.01) | < 14.48 | 2.67 (-5.56, 11.61) |
| Yes | ≥ 46.03 | 19.73 (11.22, 28.90) | ≥ 64.71 | 25.09 (16.39, 34.44) | ≥ 16.87 | 29.63 (20.17, 39.83) | ≥ 14.48 | 19.59 (11.39, 28.39) |
| RERI |  | 0.13 (0.03, 0.23) |  | 0.27 (0.16, 0.37) |  | 0.10 (-0.02, 0.21) |  | 0.10 (-0.01, 0.21) |
| 10^th^ (4d) ^a^ |  |  |  |  |  |  |  |  |
| No | < 46.03 | 1.00 | < 64.71 | 1.00 | < 16.87 | 1.00 | < 14.48 | 1.00 |
| No | ≥ 46.03 | 4.63 (0.49, 8.94) | ≥ 64.71 | 3.94 (0.12, 7.91) | ≥ 16.87 | 15.16 (9.44, 21.17) | ≥ 14.48 | 6.22 (2.02, 10.59) |
| Yes | < 46.03 | 0.81 (-7.65, 10.04) | < 64.71 | -7.93 (-16.00, 0.92) | < 16.87 | 3.54 (-6.68, 14.87) | < 14.48 | -2.27 (-11.12, 7.46) |
| Yes | ≥ 46.03 | 19.49 (10.40, 29.33) | ≥ 64.71 | 24.67 (15.53, 34.53) | ≥ 16.87 | 30.16 (19.98, 41.20) | ≥ 14.48 | 21.12 (12.21, 30.72) |
| RERI |  | 0.14 (0.02, 0.26) |  | 0.29 (0.17, 0.40) |  | 0.11 (-0.02, 0.25) |  | 0.17 (0.05, 0.29) |
| 7.5^th^ (2d) ^a^ |  |  |  |  |  |  |  |  |
| No | < 46.03 | 1.00 | < 64.71 | 1.00 | < 16.87 | 1.00 | < 14.48 | 1.00 |
| No | ≥ 46.03 | 4.89 (0.65, 9.31) | ≥ 64.71 | 4.23 (0.31, 8.29) | ≥ 16.87 | 14.64 (8.84, 20.75) | ≥ 14.48 | 7.13 (2.84, 11.60) |
| Yes | < 46.03 | 2.70 (-5.24, 11.30) | < 64.71 | -3.90 (-11.44, 4.28) | < 16.87 | 2.67 (-6.48, 12.70) | < 14.48 | 3.04 (-5.03, 11.79) |
| Yes | ≥ 46.03 | 19.67 (10.99, 29.02) | ≥ 64.71 | 25.02 (16.09, 34.64) | ≥ 16.87 | 31.82 (21.85, 42.61) | ≥ 14.48 | 21.88 (12.95, 31.51) |
| RERI |  | 0.12 (0.02, 0.23) |  | 0.25 (0.14, 0.35) |  | 0.15 (0.02, 0.27) |  | 0.12 (0.01, 0.23) |
| 7.5^th^ (3d) ^a^ |  |  |  |  |  |  |  |  |
| No | < 46.03 | 1.00 | < 64.71 | 1.00 | < 16.87 | 1.00 | < 14.48 | 1.00 |
| No | ≥ 46.03 | 5.19 (1.02, 9.54) | ≥ 64.71 | 4.54 (0.69, 8.54) | ≥ 16.87 | 14.86 (9.14, 20.89) | ≥ 14.48 | 7.20 (2.95, 11.62) |
| Yes | < 46.03 | 4.26 (-4.28, 13.56) | < 64.71 | -3.89 (-12.05, 5.03) | < 16.87 | 3.51 (-6.37, 14.44) | < 14.48 | 3.72 (-5.15, 13.41) |
| Yes | ≥ 46.03 | 20.26 (11.15, 30.11) | ≥ 64.71 | 25.67 (16.43, 35.64) | ≥ 16.87 | 32.70 (22.39, 43.88) | ≥ 14.48 | 22.27 (13.05, 32.25) |
| RERI |  | 0.11 (-0.01, 0.22) |  | 0.25 (0.14, 0.37) |  | 0.14 (0.01, 0.27) |  | 0.11 (-0.01, 0.24) |
| 7.5^th^ (4d) ^a^ |  |  |  |  |  |  |  |  |
| No | < 46.03 | 1.00 | < 64.71 | 1.00 | < 16.87 | 1.00 | < 14.48 | 1.00 |
| No | ≥ 46.03 | 5.04 (0.96, 9.29) | ≥ 64.71 | 4.55 (0.78, 8.46) | ≥ 16.87 | 14.55 (8.96, 20.42) | ≥ 14.48 | 6.80 (2.61, 11.16) |
| Yes | < 46.03 | 1.27 (-8.41, 11.97) | < 64.71 | -9.53 (-18.78, 0.77) | < 16.87 | -0.29 (-11.32, 12.12) | < 14.48 | -1.15 (-11.06, 9.87) |
| Yes | ≥ 46.03 | 18.30 (8.31, 29.21) | ≥ 64.71 | 24.25 (14.19, 35.19) | ≥ 16.87 | 31.27 (20.00, 43.61) | ≥ 14.48 | 21.41 (11.24, 32.52) |
| RERI |  | 0.12 (-0.01, 0.25) |  | 0.29 (0.16, 0.43) |  | 0.17 (0.02, 0.32) |  | 0.16 (0.02, 0.30) |
| 5^th^ (2d) ^a^ |  |  |  |  |  |  |  |  |
| No | < 46.03 | 1.00 | < 64.71 | 1.00 | < 16.87 | 1.00 | < 14.48 | 1.00 |
| No | ≥ 46.03 | 3.88 (-0.22, 8.14) | ≥ 64.71 | 3.61 (-0.19, 7.55) | ≥ 16.87 | 12.92 (7.29, 18.83) | ≥ 14.48 | 6.22 (2.03, 10.57) |
| Yes | < 46.03 | -2.01 (-10.27, 7.01) | < 64.71 | -9.25 (-17.04, -0.72) | < 16.87 | -3.65 (-12.83, 6.50) | < 14.48 | -1.98 (-10.29, 7.11) |
| Yes | ≥ 46.03 | 26.02 (15.64, 37.33) | ≥ 64.71 | 34.61 (23.70, 46.49) | ≥ 16.87 | 38.88 (27.31, 51.51) | ≥ 14.48 | 29.10 (18.33, 40.85) |
| RERI |  | 0.24 (0.11, 0.37) |  | 0.40 (0.27, 0.53) |  | 0.30 (0.16, 0.43) |  | 0.25 (0.12, 0.38) |
| 5^th^ (3d) ^a^ |  |  |  |  |  |  |  |  |
| No | < 46.03 | 1.00 | < 64.71 | 1.00 | < 16.87 | 1.00 | < 14.48 | 1.00 |
| No | ≥ 46.03 | 4.92 (0.87, 9.13) | ≥ 64.71 | 4.66 (0.91, 8.55) | ≥ 16.87 | 13.07 (7.58, 18.85) | ≥ 14.48 | 6.89 (2.71, 11.23) |
| Yes | < 46.03 | 4.92 (-5.25, 16.17) | < 64.71 | -5.29 (-14.80, 5.27) | < 16.87 | -3.91 (-14.75, 8.30) | < 14.48 | 3.33 (-6.79, 14.55) |
| Yes | ≥ 46.03 | 26.70 (15.57, 38.90) | ≥ 64.71 | 35.50 (23.87, 48.22) | ≥ 16.87 | 43.80 (31.19, 57.62) | ≥ 14.48 | 31.73 (19.89, 44.73) |
| RERI |  | 0.17 (0.02, 0.32) |  | 0.36 (0.21, 0.51) |  | 0.35 (0.18, 0.51) |  | 0.22 (0.06, 0.37) |
| 5^th^ (4d) ^a^ |  |  |  |  |  |  |  |  |
| No | < 46.03 | 1.00 | < 64.71 | 1.00 | < 16.87 | 1.00 | < 14.48 | 1.00 |
| No | ≥ 46.03 | 4.56 (0.55, 8.72) | ≥ 64.71 | 4.50 (0.78, 8.36) | ≥ 16.87 | 12.54 (7.10, 18.25) | ≥ 14.48 | 6.03 (1.91, 10.31) |
| Yes | < 46.03 | -0.11 (-10.81, 11.88) | < 64.71 | -10.64 (-20.97, 1.05) | < 16.87 | -8.72 (-19.92, 4.05) | < 14.48 | -5.48 (-16.12, 6.50) |
| Yes | ≥ 46.03 | 33.76 (20.32, 48.71) | ≥ 64.71 | 40.10 (26.72, 54.90) | ≥ 16.87 | 52.10 (36.97, 68.91) | ≥ 14.48 | 40.23 (26.23, 55.79) |
| RERI |  | 0.29 (0.12, 0.47) |  | 0.46 (0.29, 0.63) |  | 0.48 (0.29, 0.67) |  | 0.40 (0.22, 0.58) |
| 2.5^th^ (2d) ^a^ |  |  |  |  |  |  |  |  |
| No | < 46.03 | 1.00 | < 64.71 | 1.00 | < 16.87 | 1.00 | < 14.48 | 1.00 |
| No | ≥ 46.03 | 4.61 (0.62, 8.77) | ≥ 64.71 | 4.95 (1.23, 8.81) | ≥ 16.87 | 11.22 (5.84, 16.88) | ≥ 14.48 | 6.97 (2.86, 11.25) |
| Yes | < 46.03 | 2.43 (-8.31, 14.43) | < 64.71 | -5.97 (-16.53, 5.93) | < 16.87 | -11.70 (-22.07, 0.06) | < 14.48 | 2.40 (-8.95, 15.17) |
| Yes | ≥ 46.03 | 35.64 (21.38, 51.57) | ≥ 64.71 | 42.49 (28.18, 58.39) | ≥ 16.87 | 62.85 (46.14, 81.47) | ≥ 14.48 | 36.01 (21.99, 51.65) |
| RERI |  | 0.29 (0.10, 0.47) |  | 0.44 (0.25, 0.62) |  | 0.63 (0.43, 0.83) |  | 0.27 (0.08, 0.45) |
| 2.5^th^ (3d) ^a^ |  |  |  |  |  |  |  |  |
| No | < 46.03 | 1.00 | < 64.71 | 1.00 | < 16.87 | 1.00 | < 14.48 | 1.00 |
| No | ≥ 46.03 | 5.25 (1.29, 9.37) | ≥ 64.71 | 5.44 (1.75, 9.27) | ≥ 16.87 | 12.52 (7.19, 18.12) | ≥ 14.48 | 7.35 (3.25, 11.61) |
| Yes | < 46.03 | 7.56 (-6.19, 23.31) | < 64.71 | -7.38 (-20.43, 7.81) | < 16.87 | -10.14 (-21.89, 3.37) | < 14.48 | 5.00 (-8.17, 20.07) |
| Yes | ≥ 46.03 | 26.76 (12.59, 42.72) | ≥ 64.71 | 36.24 (21.83, 52.37) | ≥ 16.87 | 65.62 (46.37, 87.41) | ≥ 14.48 | 34.14 (18.70, 51.59) |
| RERI |  | 0.14 (-0.07, 0.35) |  | 0.38 (0.18, 0.59) |  | 0.63 (0.40, 0.87) |  | 0.22 (0.01, 0.43) |
| 2.5^th^ (4d) ^a^ |  |  |  |  |  |  |  |  |
| No | < 46.03 | 1.00 | < 64.71 | 1.00 | < 16.87 | 1.00 | < 14.48 | 1.00 |
| No | ≥ 46.03 | 5.41 (1.45, 9.52) | ≥ 64.71 | 5.52 (1.83, 9.33) | ≥ 16.87 | 12.83 (7.53, 18.39) | ≥ 14.48 | 6.92 (2.85, 11.15) |
| Yes | < 46.03 | 6.39 (-8.75, 24.03) | < 64.71 | -13.45 (-27.54, 3.38) | < 16.87 | -14.63 (-27.20, 0.11) | < 14.48 | -5.18 (-19.28, 11.37) |
| Yes | ≥ 46.03 | 23.63 (8.54, 40.81) | ≥ 64.71 | 34.50 (19.19, 51.77) | ≥ 16.87 | 64.16 (43.47, 87.84) | ≥ 14.48 | 35.60 (19.23, 54.22) |
| RERI |  | 0.12 (-0.11, 0.35) |  | 0.42 (0.20, 0.65) |  | 0.66 (0.41, 0.91) |  | 0.34 (0.11, 0.57) |
| Shenzhen |  |  |  |  |  |  |  |  |
| 10^th^ (2d) ^a^ |  |  |  |  |  |  |  |  |
| No | < 39.46 | 1.00 | < 58.15 | 1.00 | < 14.71 | 1.00 | < 13.27 | 1.00 |
| No | ≥ 39.46 | -0.53 (-6.37, 5.68) | ≥ 58.15 | 0.26 (-5.75, 6.66) | ≥ 14.71 | 1.76 (-5.82, 9.96) | ≥ 13.27 | 0.17 (-6.94, 7.81) |
| Yes | < 39.46 | -1.68 (-12.28, 10.21) | < 58.15 | -0.92 (-11.52, 10.95) | < 14.71 | -3.88 (-16.82, 11.07) | < 13.27 | -3.71 (-23.13, 20.61) |
| Yes | ≥ 39.46 | 1.14 (-10.11, 13.80) | ≥ 58.15 | 1.84 (-9.98, 15.21) | ≥ 14.71 | 3.97 (-7.60, 16.98) | ≥ 13.27 | 0.65 (-9.97, 12.51) |
| RERI |  | 0.03 (-0.11, 0.17) |  | 0.02 (-0.12, 0.17) |  | 0.06 (-0.10, 0.22) |  | 0.04 (-0.18, 0.27) |
| 10^th^ (3d) ^a^ |  |  |  |  |  |  |  |  |
| No | < 39.46 | 1.00 | < 58.15 | 1.00 | < 14.71 | 1.00 | < 13.27 | 1.00 |
| No | ≥ 39.46 | 0.78 (-5.06, 6.99) | ≥ 58.15 | 1.42 (-4.61, 7.83) | ≥ 14.71 | 1.45 (-6.08, 9.57) | ≥ 13.27 | 3.87 (-3.04, 11.27) |
| Yes | < 39.46 | -2.49 (-13.96, 10.50) | < 58.15 | -1.54 (-12.91, 11.32) | < 14.71 | -4.07 (-18.75, 13.26) | < 13.27 | -4.23 (-16.21, 9.46) |
| Yes | ≥ 39.46 | 1.51 (-10.17, 14.72) | ≥ 58.15 | 2.08 (-10.23, 16.07) | ≥ 14.71 | 1.65 (-9.95, 14.76) | ≥ 13.27 | 7.84 (-5.44, 22.98) |
| RERI |  | 0.03 (-0.12, 0.19) |  | 0.02 (-0.13, 0.18) |  | 0.04 (-0.13, 0.22) |  | 0.08 (-0.09, 0.26) |
| 10^th^ (4d) ^a^ |  |  |  |  |  |  |  |  |
| No | < 39.46 | 1.00 | < 58.15 | 1.00 | < 14.71 | 1.00 | < 13.27 | 1.00 |
| No | ≥ 39.46 | 1.17 (-4.60, 7.28) | ≥ 58.15 | 1.55 (-4.37, 7.84) | ≥ 14.71 | 0.43 (-6.91, 8.34) | ≥ 13.27 | 3.99 (-2.88, 11.36) |
| Yes | < 39.46 | -1.06 (-13.30, 12.92) | < 58.15 | -1.01 (-12.97, 12.60) | < 14.71 | -9.02 (-24.09, 9.05) | < 13.27 | -4.77 (-17.31, 9.66) |
| Yes | ≥ 39.46 | 2.08 (-9.83, 15.57) | ≥ 58.15 | 3.19 (-9.44, 17.58) | ≥ 14.71 | 3.18 (-8.74, 16.65) | ≥ 13.27 | 10.14 (-3.83, 26.12) |
| RERI |  | 0.02 (-0.14, 0.18) |  | 0.03 (-0.14, 0.19) |  | 0.12 (-0.07, 0.30) |  | 0.11 (-0.07, 0.29) |
| 7.5^th^ (2d) ^a^ |  |  |  |  |  |  |  |  |
| No | < 39.46 | 1.00 | < 58.15 | 1.00 | < 14.71 | 1.00 | < 13.27 | 1.00 |
| No | ≥ 39.46 | 1.59 (-4.23, 7.78) | ≥ 58.15 | 2.19 (-3.80, 8.57) | ≥ 14.71 | 2.83 (-4.83, 11.12) | ≥ 13.27 | 3.95 (-2.88, 11.27) |
| Yes | < 39.46 | 8.52 (-4.19, 22.91) | < 58.15 | 7.79 (-4.68, 21.90) | < 14.71 | 2.18 (-12.50, 19.31) | < 13.27 | -3.01 (-15.33, 11.09) |
| Yes | ≥ 39.46 | 2.68 (-9.29, 16.24) | ≥ 58.15 | 4.21 (-8.53, 18.72) | ≥ 14.71 | 9.25 (-3.68, 23.93) | ≥ 13.27 | 16.93 (2.91, 32.85) |
| RERI |  | -0.07 (-0.24, 0.09) |  | -0.06 (-0.23, 0.11) |  | 0.04 (-0.14, 0.23) |  | 0.16 (-0.02, 0.34) |
| 7.5^th^ (3d) ^a^ |  |  |  |  |  |  |  |  |
| No | < 39.46 | 1.00 | < 58.15 | 1.00 | < 14.71 | 1.00 | < 13.27 | 1.00 |
| No | ≥ 39.46 | 2.86 (-2.91, 8.98) | ≥ 58.15 | 3.45 (-2.48, 9.74) | ≥ 14.71 | 2.61 (-4.94, 10.75) | ≥ 13.27 | 5.24 (-1.61, 12.57) |
| Yes | < 39.46 | 11.84 (-2.70, 28.55) | < 58.15 | 10.80 (-3.19, 26.82) | < 14.71 | 4.69 (-11.25, 23.48) | < 13.27 | 4.39 (-10.00, 21.09) |
| Yes | ≥ 39.46 | 4.10 (-8.20, 18.05) | ≥ 58.15 | 5.39 (-7.65, 20.27) | ≥ 14.71 | 8.98 (-4.53, 24.40) | ≥ 13.27 | 13.36 (-0.90, 29.68) |
| RERI |  | -0.11 (-0.29, 0.08) |  | -0.09 (-0.27, 0.09) |  | 0.02 (-0.18, 0.22) |  | 0.04 (-0.16, 0.24) |
| 7.5^th^ (4d) ^a^ |  |  |  |  |  |  |  |  |
| No | < 39.46 | 1.00 | < 58.15 | 1.00 | < 14.71 | 1.00 | < 13.27 | 1.00 |
| No | ≥ 39.46 | 2.24 (-3.38, 8.19) | ≥ 58.15 | 2.23 (-3.51, 8.31) | ≥ 14.71 | 2.01 (-5.36, 9.97) | ≥ 13.27 | 5.34 (-1.42, 12.55) |
| Yes | < 39.46 | 10.16 (-6.75, 30.14) | < 58.15 | 5.35 (-10.07, 23.41) | < 14.71 | 2.07 (-15.75, 23.65) | < 13.27 | 3.38 (-13.97, 24.22) |
| Yes | ≥ 39.46 | 3.45 (-9.62, 18.41) | ≥ 58.15 | 6.36 (-7.74, 22.63) | ≥ 14.71 | 7.74 (-6.60, 24.28) | ≥ 13.27 | 11.57 (-3.24, 28.64) |
| RERI |  | -0.09 (-0.30, 0.12) |  | -0.01 (-0.22, 0.19) |  | 0.04 (-0.19, 0.26) |  | 0.03 (-0.20, 0.26) |
| 5^th^ (2d) ^a^ |  |  |  |  |  |  |  |  |
| No | < 39.46 | 1.00 | < 58.15 | 1.00 | < 14.71 | 1.00 | < 13.27 | 1.00 |
| No | ≥ 39.46 | 1.38 (-4.24, 7.31) | ≥ 58.15 | 1.84 (-3.93, 7.96) | ≥ 14.71 | 0.47 (-6.85, 8.37) | ≥ 13.27 | 5.33 (-1.44, 12.56) |
| Yes | < 39.46 | 11.02 (-4.06, 28.47) | < 58.15 | 8.53 (-5.61, 24.80) | < 14.71 | -9.99 (-24.73, 7.65) | < 13.27 | 2.32 (-12.93, 20.23) |
| Yes | ≥ 39.46 | 2.09 (-10.81, 16.86) | ≥ 58.15 | 4.22 (-9.94, 20.62) | ≥ 14.71 | 13.44 (-1.00, 29.97) | ≥ 13.27 | 14.10 (-0.50, 30.84) |
| RERI |  | -0.10 (-0.30, 0.09) |  | -0.06 (-0.26, 0.14) |  | 0.23 (0.03, 0.43) |  | 0.06 (-0.14, 0.27) |
| 5^th^ (3d) ^a^ |  |  |  |  |  |  |  |  |
| No | < 39.46 | 1.00 | < 58.15 | 1.00 | < 14.71 | 1.00 | < 13.27 | 1.00 |
| No | ≥ 39.46 | 1.87 (-3.69, 7.75) | ≥ 58.15 | 2.36 (-3.34, 8.39) | ≥ 14.71 | 0.80 (-6.47, 8.64) | ≥ 13.27 | 5.35 (-1.35, 12.51) |
| Yes | < 39.46 | 5.85 (-11.71, 26.90) | < 58.15 | 4.86 (-11.52, 24.27) | < 14.71 | -8.17 (-25.07, 12.54) | < 13.27 | 3.84 (-15.05, 26.93) |
| Yes | ≥ 39.46 | 1.20 (-12.28, 16.74) | ≥ 58.15 | 2.16 (-12.30, 19.02) | ≥ 14.71 | 7.21 (-7.65, 24.46) | ≥ 13.27 | 6.83 (-7.71, 23.66) |
| RERI |  | -0.07 (-0.29, 0.16) |  | -0.05 (-0.27, 0.17) |  | 0.15 (-0.08, 0.37) |  | -0.02 (-0.27, 0.22) |
| 5^th^ (4d) ^a^ |  |  |  |  |  |  |  |  |
| No | < 39.46 | 1.00 | < 58.15 | 1.00 | < 14.71 | 1.00 | < 13.27 | 1.00 |
| No | ≥ 39.46 | 1.58 (-3.92, 7.39) | ≥ 58.15 | 1.74 (-3.86, 7.68) | ≥ 14.71 | 1.29 (-5.97, 9.11) | ≥ 13.27 | 5.02 (-1.58, 12.07) |
| Yes | < 39.46 | 2.58 (-18.39, 28.93) | < 58.15 | -0.89 (-19.83, 22.52) | < 14.71 | -5.85 (-25.22, 18.55) | < 13.27 | -4.16 (-29.77, 30.81) |
| Yes | ≥ 39.46 | 1.41 (-13.67, 19.13) | ≥ 58.15 | 3.77 (-12.48, 23.03) | ≥ 14.71 | 5.82 (-10.98, 25.79) | ≥ 13.27 | 6.69 (-8.69, 24.66) |
| RERI |  | -0.03 (-0.29, 0.24) |  | 0.03 (-0.23, 0.29) |  | 0.10 (-0.16, 0.37) |  | 0.06 (-0.26, 0.38) |
| 2.5^th^ (2d) ^a^ |  |  |  |  |  |  |  |  |
| No | < 39.46 | 1.00 | < 58.15 | 1.00 | < 14.71 | 1.00 | < 13.27 | 1.00 |
| No | ≥ 39.46 | 0.85 (-4.56, 6.57) | ≥ 58.15 | 1.45 (-4.05, 7.26) | ≥ 14.71 | 0.43 (-6.71, 8.12) | ≥ 13.27 | 5.58 (-1.00, 12.60) |
| Yes | < 39.46 | 12.30 (-7.35, 36.12) | < 58.15 | 11.04 (-7.76, 33.68) | < 14.71 | -15.73 (-33.33, 6.52) | < 13.27 | 5.75 (-17.89, 36.18) |
| Yes | ≥ 39.46 | 0.94 (-16.58, 22.14) | ≥ 58.15 | 1.89 (-16.98, 25.05) | ≥ 14.71 | 20.62 (1.56, 43.26) | ≥ 13.27 | 11.91 (-4.87, 31.66) |
| RERI |  | -0.12 (-0.41, 0.16) |  | -0.11 (-0.40, 0.19) |  | 0.36 (0.08, 0.63) |  | 0.01 (-0.31, 0.32) |
| 2.5^th^ (3d) ^a^ |  |  |  |  |  |  |  |  |
| No | < 39.46 | 1.00 | < 58.15 | 1.00 | < 14.71 | 1.00 | < 13.27 | 1.00 |
| No | ≥ 39.46 | 1.65 (-3.75, 7.35) | ≥ 58.15 | 2.02 (-3.46, 7.80) | ≥ 14.71 | 0.83 (-6.31, 8.51) | ≥ 13.27 | 5.91 (-0.64, 12.88) |
| Yes | < 39.46 | 5.22 (-18.15, 35.26) | < 58.15 | 2.38 (-18.69, 28.90) | < 14.71 | -13.54 (-33.39, 12.22) | < 13.27 | 31.21 (-8.73, 88.63) |
| Yes | ≥ 39.46 | -2.49 (-20.40, 19.45) | ≥ 58.15 | -0.75 (-20.10, 23.29) | ≥ 14.71 | 10.59 (-10.22, 36.22) | ≥ 13.27 | -1.28 (-17.56, 18.23) |
| RERI |  | -0.09 (-0.42, 0.24) |  | -0.05 (-0.37, 0.27) |  | 0.23 (-0.09, 0.55) |  | -0.38 (-0.89, 0.12) |
| 2.5^th^ (4d) ^a^ |  |  |  |  |  |  |  |  |
| No | < 39.46 | 1.00 | < 58.15 | 1.00 | < 14.71 | 1.00 | < 13.27 | 1.00 |
| No | ≥ 39.46 | 1.71 (-3.68, 7.40) | ≥ 58.15 | 2.32 (-3.16, 8.11) | ≥ 14.71 | 1.16 (-5.94, 8.79) | ≥ 13.27 | 5.85 (-0.68, 12.81) |
| Yes | < 39.46 | 3.39 (-25.56, 43.59) | < 58.15 | 7.32 (-20.82, 45.47) | < 14.71 | -15.62 (-38.12, 15.07) | < 13.27 | 49.15 (-17.70, 170.29) |
| Yes | ≥ 39.46 | -6.61 (-25.20, 16.60) | ≥ 58.15 | -8.60 (-27.45, 15.15) | ≥ 14.71 | 4.21 (-17.64, 31.86) | ≥ 13.27 | -4.16 (-21.42, 16.89) |
| RERI |  | -0.12 (-0.52, 0.28) |  | -0.18 (-0.57, 0.21) |  | 0.19 (-0.17, 0.55) |  | -0.59 (-1.50, 0.32) |
| Zhaoqing |  |  |  |  |  |  |  |  |
| 10^th^ (2d) ^b^ |  |  |  |  |  |  |  |  |
| No | < 44.50 | 1.00 | < 59.50 | 1.00 | < 13.41 | 1.00 | < 14.58 | 1.00 |
| No | ≥ 44.50 | 0.71 (-4.22, 5.91) | ≥ 59.50 | 0.39 (-4.58, 5.62) | ≥ 13.41 | 0.27 (-5.25, 6.12) | ≥ 14.58 | 2.08 (-3.32, 7.78) |
| Yes | < 44.50 | -12.47 (-21.20, -2.77) | < 59.50 | -13.15 (-21.91, -3.42) | < 13.41 | -9.29 (-18.49, 0.95) | < 14.58 | -11.92 (-21.57, -1.10) |
| Yes | ≥ 44.50 | 4.76 (-7.92, 19.17) | ≥ 59.50 | 7.36 (-6.39, 23.14) | ≥ 13.41 | -3.43 (-15.49, 10.36) | ≥ 14.58 | 0.14 (-10.58, 12.15) |
| RERI |  | 0.17 (0.01, 0.32) |  | 0.20 (0.04, 0.37) |  | 0.06 (-0.09, 0.21) |  | 0.10 (-0.04, 0.24) |
| 10^th^ (3d) ^b^ |  |  |  |  |  |  |  |  |
| No | < 44.50 | 1.00 | < 59.50 | 1.00 | < 13.41 | 1.00 | < 14.58 | 1.00 |
| No | ≥ 44.50 | 0.90 (-3.94, 5.98) | ≥ 59.50 | 0.55 (-4.28, 5.63) | ≥ 13.41 | -0.30 (-5.73, 5.44) | ≥ 14.58 | 2.33 (-3.05, 8.00) |
| Yes | < 44.50 | -13.97 (-23.33, -3.46) | < 59.50 | -14.83 (-24.01, -4.54) | < 13.41 | -12.52 (-22.24, -1.57) | < 14.58 | -13.10 (-24.14, -0.45) |
| Yes | ≥ 44.50 | 6.94 (-6.31, 22.07) | ≥ 59.50 | 11.40 (-3.39, 28.47) | ≥ 13.41 | 1.43 (-11.54, 16.30) | ≥ 14.58 | 0.42 (-10.44, 12.59) |
| RERI |  | 0.20 (0.04, 0.36) |  | 0.26 (0.08, 0.43) |  | 0.14 (-0.02, 0.30) |  | 0.11 (-0.04, 0.27) |
| 10^th^ (4d) ^b^ |  |  |  |  |  |  |  |  |
| No | < 44.50 | 1.00 | < 59.50 | 1.00 | < 13.41 | 1.00 | < 14.58 | 1.00 |
| No | ≥ 44.50 | 1.48 (-3.29, 6.49) | ≥ 59.50 | 1.34 (-3.42, 6.35) | ≥ 13.41 | 0.64 (-4.77, 6.37) | ≥ 14.58 | 1.92 (-3.42, 7.56) |
| Yes | < 44.50 | -6.94 (-18.28, 5.98) | < 59.50 | -7.92 (-18.90, 4.56) | < 13.41 | -5.60 (-16.66, 6.92) | < 14.58 | -7.73 (-20.74, 7.43) |
| Yes | ≥ 44.50 | 15.88 (1.24, 32.64) | ≥ 59.50 | 21.32 (4.84, 40.40) | ≥ 13.41 | 17.35 (0.77, 36.66) | ≥ 14.58 | 10.99 (-1.63, 25.22) |
| RERI |  | 0.21 (0.02, 0.40) |  | 0.28 (0.07, 0.48) |  | 0.22 (0.02, 0.43) |  | 0.17 (-0.02, 0.35) |
| 7.5^th^ (2d) ^b^ |  |  |  |  |  |  |  |  |
| No | < 44.50 | 1.00 | < 59.50 | 1.00 | < 13.41 | 1.00 | < 14.58 | 1.00 |
| No | ≥ 44.50 | 1.49 (-3.31, 6.52) | ≥ 59.50 | 1.64 (-3.19, 6.72) | ≥ 13.41 | 0.77 (-4.67, 6.51) | ≥ 14.58 | 3.14 (-2.19, 8.76) |
| Yes | < 44.50 | -11.12 (-20.78, -0.29) | < 59.50 | -10.06 (-19.67, 0.69) | < 13.41 | -7.16 (-17.21, 4.12) | < 14.58 | -7.50 (-18.88, 5.48) |
| Yes | ≥ 44.50 | 11.92 (-2.84, 28.93) | ≥ 59.50 | 14.15 (-2.20, 33.25) | ≥ 13.41 | 3.06 (-11.60, 20.15) | ≥ 14.58 | 2.66 (-9.33, 16.23) |
| RERI |  | 0.22 (0.04, 0.39) |  | 0.23 (0.03, 0.42) |  | 0.09 (-0.09, 0.28) |  | 0.07 (-0.10, 0.24) |
| 7.5^th^ (3d) ^b^ |  |  |  |  |  |  |  |  |
| No | < 44.50 | 1.00 | < 59.50 | 1.00 | < 13.41 | 1.00 | < 14.58 | 1.00 |
| No | ≥ 44.50 | 1.19 (-3.48, 6.08) | ≥ 59.50 | 1.39 (-3.29, 6.30) | ≥ 13.41 | 0.40 (-4.87, 5.96) | ≥ 14.58 | 2.41 (-2.77, 7.87) |
| Yes | < 44.50 | -21.89 (-32.43, -9.70) | < 59.50 | -18.64 (-28.97, -6.80) | < 13.41 | -14.96 (-26.01, -2.26) | < 14.58 | -22.50 (-35.67, -6.63) |
| Yes | ≥ 44.50 | 13.73 (-1.91, 31.87) | ≥ 59.50 | 16.26 (-1.33, 36.98) | ≥ 13.41 | 4.49 (-11.14, 22.86) | ≥ 14.58 | 2.84 (-9.53, 16.90) |
| RERI |  | 0.34 (0.15, 0.54) |  | 0.34 (0.12, 0.55) |  | 0.19 (-0.01, 0.39) |  | 0.23 (0.04, 0.42) |
| 7.5^th^ (4d) ^b^ |  |  |  |  |  |  |  |  |
| No | < 44.50 | 1.00 | < 59.50 | 1.00 | < 13.41 | 1.00 | < 14.58 | 1.00 |
| No | ≥ 44.50 | 1.91 (-2.75, 6.80) | ≥ 59.50 | 1.90 (-2.77, 6.80) | ≥ 13.41 | 1.20 (-4.09, 6.79) | ≥ 14.58 | 2.59 (-2.58, 8.04) |
| Yes | < 44.50 | -13.71 (-26.55, 1.37) | < 59.50 | -12.03 (-24.31, 2.23) | < 13.41 | -8.03 (-20.30, 6.13) | < 14.58 | -14.93 (-30.73, 4.49) |
| Yes | ≥ 44.50 | 12.83 (-3.97, 32.56) | ≥ 59.50 | 16.62 (-2.51, 39.51) | ≥ 13.41 | 11.18 (-8.66, 35.33) | ≥ 14.58 | 6.30 (-7.65, 22.36) |
| RERI |  | 0.25 (0.02, 0.47) |  | 0.27 (0.02, 0.51) |  | 0.18 (-0.07, 0.43) |  | 0.19 (-0.04, 0.42) |
| 5^th^ (2d) ^b^ |  |  |  |  |  |  |  |  |
| No | < 44.50 | 1.00 | < 59.50 | 1.00 | < 13.41 | 1.00 | < 14.58 | 1.00 |
| No | ≥ 44.50 | 1.62 (-3.06, 6.53) | ≥ 59.50 | 1.64 (-3.04, 6.55) | ≥ 13.41 | 1.01 (-4.31, 6.64) | ≥ 14.58 | 2.67 (-2.54, 8.16) |
| Yes | < 44.50 | -20.36 (-31.71, -7.13) | < 59.50 | -19.36 (-30.41, -6.56) | < 13.41 | -11.12 (-23.33, 3.03) | < 14.58 | -19.62 (-33.81, -2.38) |
| Yes | ≥ 44.50 | 13.21 (-3.74, 33.15) | ≥ 59.50 | 17.83 (-1.26, 40.60) | ≥ 13.41 | -0.53 (-16.77, 18.87) | ≥ 14.58 | 2.05 (-11.17, 17.24) |
| RERI |  | 0.32 (0.10, 0.54) |  | 0.36 (0.12, 0.59) |  | 0.10 (-0.12, 0.32) |  | 0.19 (-0.02, 0.40) |
| 5^th^ (3d) ^b^ |  |  |  |  |  |  |  |  |
| No | < 44.50 | 1.00 | < 59.50 | 1.00 | < 13.41 | 1.00 | < 14.58 | 1.00 |
| No | ≥ 44.50 | 2.30 (-2.35, 7.17) | ≥ 59.50 | 2.17 (-2.48, 7.03) | ≥ 13.41 | 1.09 (-4.17, 6.63) | ≥ 14.58 | 2.97 (-2.18, 8.38) |
| Yes | < 44.50 | -22.99 (-35.93, -7.45) | < 59.50 | -21.90 (-34.20, -7.30) | < 13.41 | -12.90 (-25.77, 2.20) | < 14.58 | -26.36 (-42.42, -5.82) |
| Yes | ≥ 44.50 | 9.80 (-7.81, 30.77) | ≥ 59.50 | 15.13 (-4.81, 39.25) | ≥ 13.41 | 0.42 (-18.67, 23.99) | ≥ 14.58 | 1.57 (-12.60, 18.03) |
| RERI |  | 0.30 (0.07, 0.54) |  | 0.35 (0.09, 0.60) |  | 0.12 (-0.13, 0.37) |  | 0.25 (0.01, 0.49) |
| 5^th^ (4d) ^b^ |  |  |  |  |  |  |  |  |
| No | < 44.50 | 1.00 | < 59.50 | 1.00 | < 13.41 | 1.00 | < 14.58 | 1.00 |
| No | ≥ 44.50 | 2.55 (-2.10, 7.43) | ≥ 59.50 | 2.22 (-2.43, 7.08) | ≥ 13.41 | 1.07 (-4.18, 6.61) | ≥ 14.58 | 2.93 (-2.20, 8.32) |
| Yes | < 44.50 | -13.36 (-28.47, 4.95) | < 59.50 | -16.17 (-30.15, 0.62) | < 13.41 | -12.98 (-26.31, 2.75) | < 14.58 | -19.90 (-38.12, 3.68) |
| Yes | ≥ 44.50 | 3.90 (-14.26, 25.91) | ≥ 59.50 | 11.11 (-9.43, 36.31) | ≥ 13.41 | 12.11 (-11.94, 42.73) | ≥ 14.58 | 2.24 (-13.09, 20.26) |
| RERI |  | 0.15 (-0.11, 0.41) |  | 0.25 (-0.02, 0.52) |  | 0.24 (-0.06, 0.54) |  | 0.19 (-0.07, 0.46) |

Note: PM, particulate matter; SO_2_, sulfur dioxide; NO_2_, nitrogen dioxide.

^a^ Cold spells were defined by percentile temperature thresholds (10^th^, 7.5^th^, 5^th^ and 2.5^th^) and by the number of consecutive days below the thresholds (2-4 d).

^b^ Nine definitions [10^th^ (2d), 10^th^ (3d), 10^th^ (4d), 7.5^th^ (2d), 7.5^th^ (3d), 7.5^th^ (4d), 5^th^ (2d), 5^th^ (3d), and 5^th^ (4d)] were used in Zhaoqing.

^c^ Air pollutants were classified as binary variables using the median of air pollutant (lag03) concentrations as a cut off.


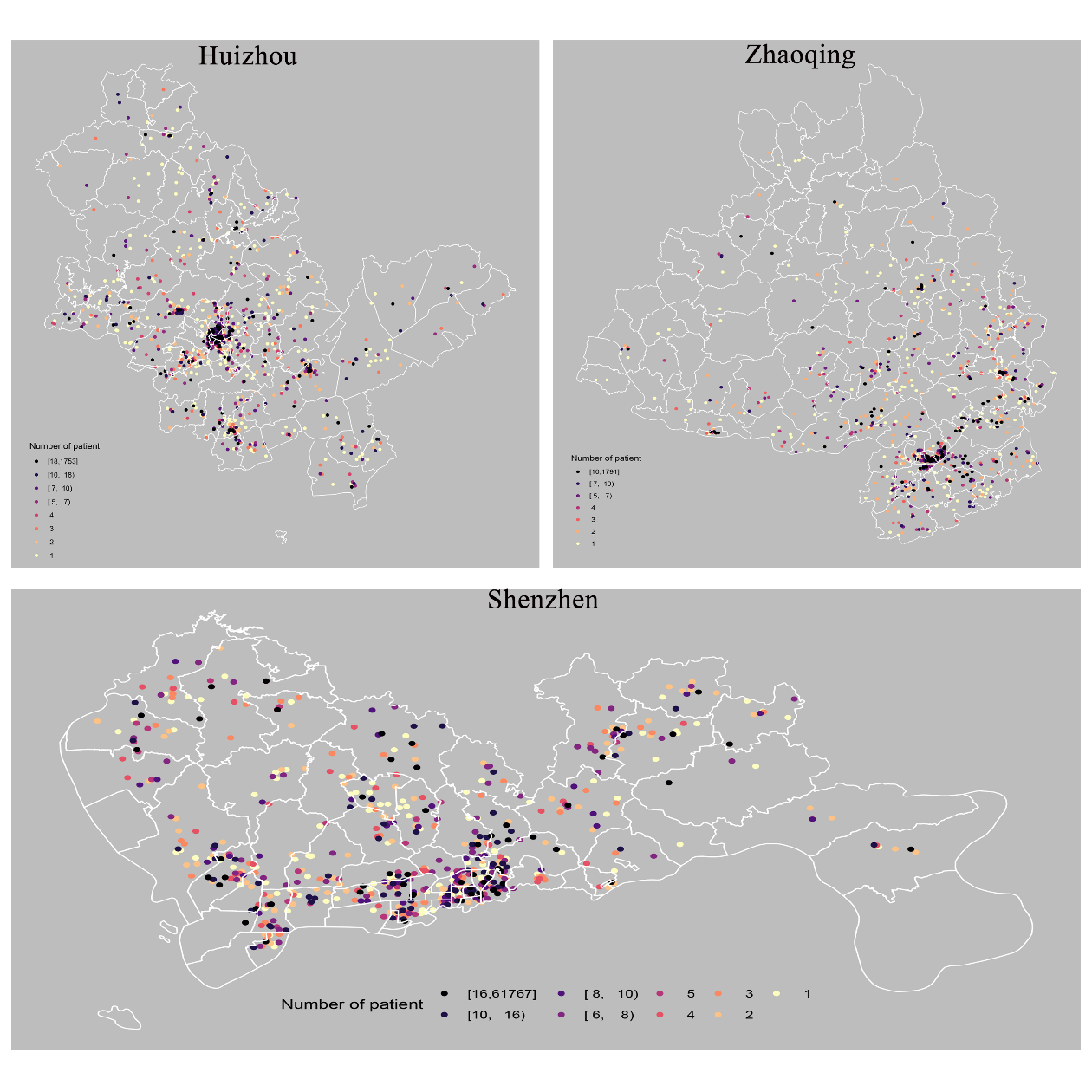


## Figure S1. Spatial distribution of the study population addresses (dots) in three subtropical Chinese cities.


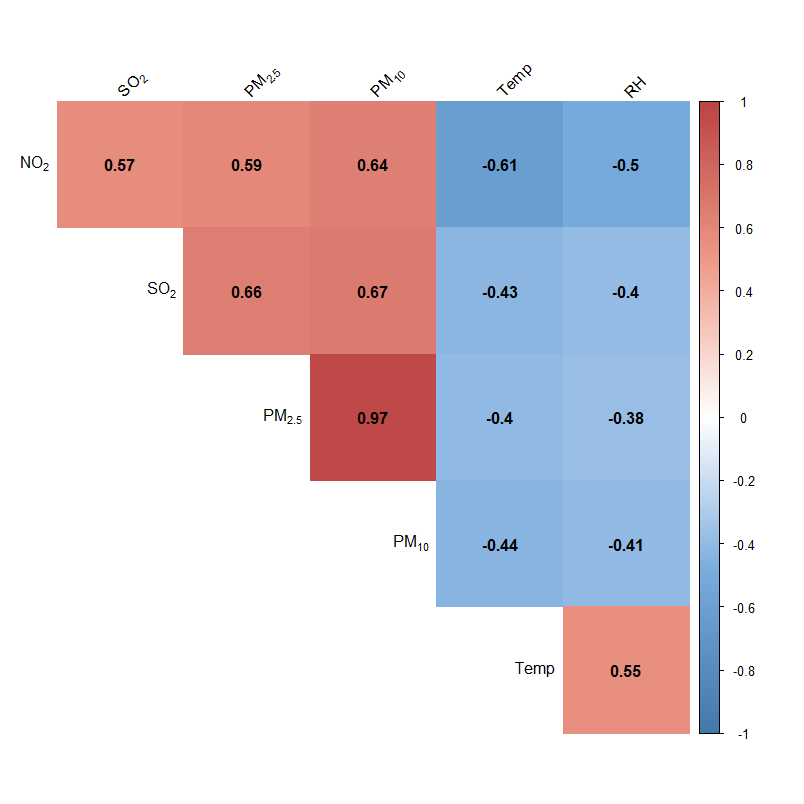


## Figure S2. Spearman’s correlation coefficients between air pollutants and meteorological conditions.

*P* for all pairwise correlations < 0.05.

Note: PM, particulate matter; SO_2_, sulfur dioxide; NO_2_, nitrogen dioxide; Temp: daily mean temperature; RH: daily mean relative humidity.
